# Pathogenic *Leptospira* Evolved a Unique Gene Family Comprised of Ricin B-Like Lectin Domain-Containing Cytotoxins

**DOI:** 10.3389/fmicb.2022.859680

**Published:** 2022-03-29

**Authors:** Reetika Chaurasia, Alan S. Marroquin, Joseph M. Vinetz, Michael A. Matthias

**Affiliations:** Section of Infectious Diseases, Department of Internal Medicine, Yale University School of Medicine, New Haven, CT, United States

**Keywords:** genotoxin, cytotoxin, lectin, cytopathic effect, pathogenesis, virulence factor, evolution, comparative genomics

## Abstract

Leptospirosis is a globally important neglected zoonotic disease. Previous data suggest that a family of virulence-modifying (VM) proteins (PF07598) is a distinctive feature of group I pathogenic *Leptospira* that evolved as important virulence determinants. Here, we show that one such VM protein, LA3490 (also known as Q8F0K3), is expressed by *Leptospira interrogans* serovar Lai, as a secreted genotoxin that is potently cytotoxic to human cells. Structural homology searches using Phyre2 suggested that VM proteins are novel R-type lectins containing tandem N-terminal ricin B-chain-like β-trefoil domains. Recombinant LA3490 (rLA3490) and an N-terminal fragment, t3490, containing only the predicted ricin B domain, bound to the terminal galactose and N-acetyl-galactosamine residues, asialofetuin, and directly competed for asialofetuin-binding sites with recombinant ricin B chain. t3490 alone was sufficient for binding, both to immobilized asialofetuin and to the HeLa cell surface but was neither internalized nor cytotoxic. Treatment of HeLa cells with rLA3490 led to cytoskeleton disassembly, caspase-3 activation, and nuclear fragmentation, and was rapidly cytolethal. rLA3490 had DNase activity on mammalian and bacterial plasmid DNA. The combination of cell surface binding, internalization, nuclear translocation, and DNase functions indicate that LA3490 and other VM proteins evolved as novel forms of the bacterial AB domain-containing toxin paradigm.

## Introduction

Leptospirosis is a globally important neglected zoonotic disease that causes weather-driven, large-scale epidemics, small-scale outbreaks, and sporadic disease, all of which have a substantial impact on human and veterinary public health. Conservative estimates suggest that the global burden of human disease due to leptospirosis is comparable with cholera and typhoid fever (Costa et al., 2015; Torgerson et al., 2015; Rudd et al., 2020). Annually, more than 1 million cases and ∼58,900 deaths are estimated to occur globally with case fatality rates ranging from 5 to 20% (Costa et al., 2015; Jimenez et al., 2018). Humans become infected after exposure to freshwater or wet soil contaminated by the urine of mammalian reservoir hosts. Clinical presentation varies from an undifferentiated fever to jaundice, renal failure, pulmonary hemorrhage, shock, and fulminant death (Ko et al., 2009; Adler and de la Pena Moctezuma, 2010; Cai et al., 2010; Evangelista and Coburn, 2010; Ellis, 2015; Haake and Levett, 2015).

Severe leptospirosis presents clinically with vascular instability, liver and renal dysfunction, and pulmonary hemorrhage, which have been postulated to be caused by circulating toxins produced by pathogenic *Leptospira*. No specific molecule, toxin, or other pathogenetic mechanisms mediating such effects *in vivo* have been delineated to date. Older reports describe general cytotoxic effects present in spent growth medium of pathogenic *Leptospira* (Levett, 2001), and previously described hemolysins (Alexander et al., 1956; Lee et al., 2002; Zhang et al., 2005), sphingomyelinases (Bernheimer and Bey, 1986; Zhang et al., 2008; Narayanavari et al., 2012; Narayanavari et al., 2015), and phospholipase activities (Yanagihara et al., 1982; Samaha, 2019) remain of unclear pathogenetic importance. Despite informative *in vitro* and small animal models that recapitulate human disease, the molecular, cellular, and immunological mechanisms of disease pathogenesis remain unclear (Ko et al., 2009; Picardeau, 2017).

Previously published genomic, pathogenomic, and gene expression data suggest that the so-called virulence-modifying (VM) proteins−defined as containing a domain of unknown function (DUF1561), PF07598 protein family−contribute to the pathogenesis of leptospirosis (Lehmann et al., 2013; Lehmann et al., 2014; Fouts et al., 2016). Predicted to have secretory signal peptides (Fouts et al., 2016) suggesting transportation extracellularly, expression of genes encoding VM proteins are upregulated *in vitro* under conditions mimicking the internal host environment (Matsunaga et al., 2007) and *in vivo* in small animal models of acute infection (Lehmann et al., 2013). Among the *Leptospira* spp., genes encoding VM proteins are restricted to group I pathogenic *Leptospira*. The cosmopolitan and lethal *L. interrogans* serovars Copenhageni and Canicola (Lehmann et al., 2013; Fouts et al., 2016) have an expanded repertoire of ≥ 12 distinct paralogs, further suggesting that they are involved in disease pathogenesis.

**FIGURE 1:**
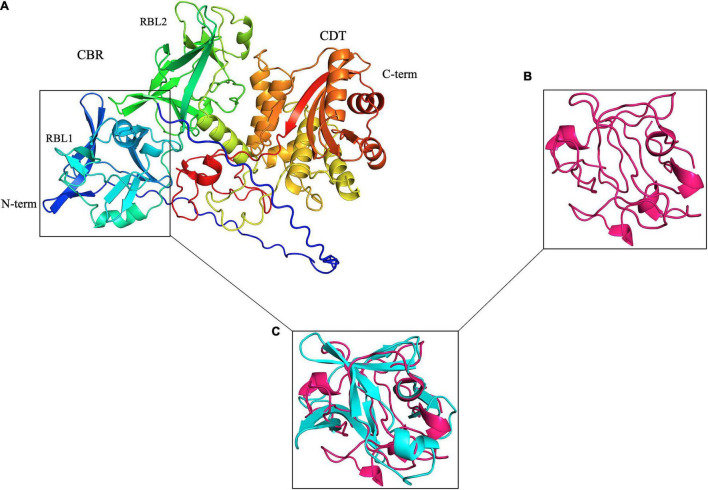
*Leptospira* PF07598 gene family members, represented by LA3490 here, are predicted with high confidence to have two tandemly repeated, N-terminal ricin B-like (RBL) lectin domains. **(A)** Visualization of an AlphaFold 3D-generated model of full-length LA3490 (Callaway, 2020; Jumper et al., 2020; Senior et al., 2020) showing four globular domains N-terminal to C-terminal (blue to red color) residues visualized in PyMOL 2.4.0 https://pymol.org/2/. Phyre2 (Protein Fold Prediction Server; http://www.sbg.bio.ic.ac.uk/phyre2/html/page.cgi?id=index) had first predicted, with high (> 94%) confidence, that LA3490, as well as all other virulence-modifying (VM) proteins encoded by the PF07598 gene family, contains N-terminal β-trefoil folds identified as ricin B domains. **(B)** Ricin B domain (PBD; 2AAI-B, 7 aa to 129 aa). **(C)** Superimposition of 2AAI-B and N-terminal region of LA3490 (i.e., amino acid positions 40–150) was performed using PyMOL (TM) 2.4.0 showing structural conservation of RBL1 and the B chain of ricin (RMSD = 1.796 Å).

Initially, using Phyre2 to carry out a structure-based remote homology search (Kelley et al., 2015), we detected a statistically well-supported ricin B-like lectin (RBL) subdomain in the N-terminus of the otherwise anonymous PF07598 protein family (Figure 1). On this basis, we hypothesized that leptospiral VM proteins were carbohydrate-binding cytotoxins (i.e., cytotoxic lectins). Here we demonstrate the pathogenic potential and mechanisms of the highly upregulated *L. interrogans* Lai paralog encoded by LA3490 (Lehmann et al., 2013), an exemplar of the VM protein family. Here we show that the LA3490 gene product mediates cytotoxicity *in vitro* and demonstrate mechanisms by which this protein does so. These results are the first rigorous demonstration of a *Leptospira*-secreted exotoxin, which has novel and direct relevance in understanding the mechanisms of leptospirosis pathogenesis and will guide new approaches to vaccine and therapeutics development.

## Materials and Methods

### Computational Analysis

To identify functional subdomains, the amino acid sequences of Q8F0K3 and its closest paralog Q8F8D7—encoded by LA3490 and LA0620 in *L. interrogans* serovar Lai, respectively—were submitted separately to the Phyre2 remote homology search portal^1^ (Kelley et al., 2015). Short functional regions and motifs, including amphipathic potentially membrane-binding α-helices, putative eukaryotic protein-sorting signals, proteolytic cleavage, and phosphorylation sites, and binding/docking motifs, were identified *via* HeliQuest^2^ (Gautier et al., 2008) and the Eukaryotic Linear Motif (ELM) resource^3^ (Kumar et al., 2020).

About 3,000 PF07598| VM proteins representing all clinically relevant *Leptospira* species—as well as *L. alexanderi* and *L. alstonii*, were aligned against a custom-built HMM model, which was based upon the full PF07598 reference alignment. Following visual inspection of the aligned amino acid residues, any (VM protein) sequence that contained ambiguous amino acids was annotated as partial—if derived from draft genomes; those that did not span at least one presumed functional subdomain [i.e., any of RBL1/RBL2/CTD (carboxy terminal domain)] were excluded. For clustering analysis, subalignments encompassing the CBR (carbohydrate-binding region) and CTD (i.e., aa positions 23–343 and 344–639, respectively, with respect to Q8F0K3) were removed from the curated global alignment and used as input for computation of discrete all vs. all pairwise distance matrices using the R package, bio2mds (Pele et al., 2012), excluding “gappy” columns (i.e., containing > 50% gaps). Pairwise distance matrices demonstrating close amino acid relatedness of the full-length alignment and CBR- and CTD-subalignments are provided for six leptospiral serovars of public health importance (*L. interrogans* serovars Copenhageni, Canicola, Hardjo, Lai, Manilae, and Pomona and *L. kirschneri* Pomona) in Supplementary Tables 1–3). Poorly aligned columns were improved manually *via* visual inspection in Jalview v2.11.4. Fragmented VM proteins (i.e., that did not span at least one functional subdomain) were removed. The curated multiple sequence alignment was used as input for HMM profile-based remote homology searches against PDB, SCOPe70, SMARTv6, and UniProt-SissProt-viral70 databases *via* HHpred^4^. A consensus secondary structure was predicted using Ali2D^5^ and the 3D (protein) structure predicted using AlphaFold (Callaway, 2020; Jumper et al., 2020; Senior et al., 2020). Separate distance matrices for the amino terminal half, encompassing the predicted lectin domain, and the C-terminal half containing the putative toxin subdomain were generated to infer their evolutionary relatedness and clustering relationships.

### *Leptospira* Culture and Virulence Gene Expression *in vitro*

Low-passage, virulent *L. interrogans* serovar Lai strain 56,601 that had been passaged through hamsters to recover high virulence (LD_50_ < 100) (Lehmann et al., 2013) were maintained at 30°C in semisolid Ellinghausen, McCullough, Johnson, and Harris medium (EMJH, BD Biosciences, United States) (Ellinghausen and McCullough, 1965). Because our published data showed that VM proteins are transcriptionally upregulated *in vivo* in a hamster model of acute leptospirosis (Lehmann et al., 2013) and to maximize their expression *in vitro*, *Leptospira* were grown under conditions mimicking the *in vivo* host environment known to induce virulence gene expression *in vitro* (Matsunaga et al., 2005). Mid-logarithmic cultures (2 × 10^8^ leptospires/ml) in EMJH medium were harvested by centrifugation at 18,514 *g*. Pelleted cells were washed twice with 1 × PBS, resuspended in liquid EMJH medium supplemented with 120 mM NaCl, and then incubated at 37°C for 4 h (Sigma Aldrich, United States).

Following virulence gene induction, culture supernatants were collected by centrifugation at 18,514 × *g* for 20 min, clarified by filtration through a 0.22-μm membrane filter (Merck Millipore, Germany), and then concentrated *via* a 30-kDa Amicon^®^ Ultra centrifugal filter (Merck Millipore, Germany). Induced and uninduced (corresponding to baseline *in vitro* expression) culture supernatants were analyzed by Western blot probed with rabbit anti-LA3490 polyclonal antiserum. Total protein was estimated by BCA assay (Pierce™ BCA Protein Assay Kit, Thermo Fisher Scientific, United States).

### Mammalian Cell Culture

HeLa cells obtained from the American Type Culture Collection (ATCC, United States) were grown as monolayers in tissue culture plates in Dulbecco’s modified Eagle medium (DMEM; Sigma-Aldrich, United States) supplemented with 10% fetal bovine serum and 1% antibiotic–antimycotic solution (penicillin, 100 units/ml; streptomycin, 100 μg/ml; and amphotericin, 25 μg/ml; Invitrogen, United States) at 37°C in a humidified incubator containing 5% CO2. Antibiotic-containing medium was replaced with fresh, antibiotic-free medium prior to each experiment.

### Plasmid Constructs and Cloning

*Escherichia coli* codon-optimized gene fusions consisting of either the complete LA3490 coding sequence (NP_713670.1) minus the predicted signal peptide (i.e., corresponding to nucleotide positions 57–1,917 bp) or an N-terminal truncation inclusive of positions 40–174 bp and encompassing the Phyre2-predicted ricin B-like lectin subdomain linked to mCherry (AST15061.1) *via* a glycine–serine hinge (GGGGSGGGGSGGGGS) were synthesized and cloned into pET32b (+) (Gene Universal Inc., United States). Prior to use, constructs were verified by sequencing.

### Recombinant Protein Expression and Purification

Because PF07598 proteins are cysteine-rich [LA3490 encode 12 cysteine, (Supplementary Table 4)], recombinant proteins were expressed in SHuffle^®^T7-competent *E. coli* cells (New England Biolabs, United States) owing to their capacity to promote disulfide bonds in the cytoplasm ensuring proper protein folding. Transformants were subcultured into Luria–Bertani (LB) medium containing 100 μg/ml of ampicillin. When cultures had reached an OD of 0.6, expression was induced at 16°C and 250 rpm for 24 h *via* addition of 1 mM isopropyl-β-D-thiogalactoside (IPTG; Sigma-Aldrich, United States).

Following induction, cells were pelleted by centrifugation and then lysed in CelLytic™ B (Cell Lysis Reagent; Sigma-Aldrich, United States) containing 50 U of benzonase nuclease (Sigma-Aldrich, United States), 0.2 μg/ml of lysozyme, non-EDTA protease inhibitor cocktail (Roche, United States) plus 1 mM PMSF (Sigma-Aldrich, United States) for 30 min at 37°C. Lysates were centrifuged at 4°C and 18,514 × *g* for 10 min. Supernatants and pellets were separated, and then analyzed by 4–12% bis-*tris* sodium dodecyl sulfate-polyacrylamide gel electrophoresis (SDS-PAGE). As above, protein concentrations were determined by BCA assay.

Recombinant thioredoxin (TRX)-His_6_-VM protein-(GGGGSGGGGSGGGGS)-mCherry-His_6_ fusion proteins were isolated using a 5-ml pre-packed Ni-Sepharose AKTA Hi-TRAP column (GE Healthcare, United States) pre-equilibrated with a buffer containing 100 mM NaH_2_PO_4_, 10 mM Tris–HCl, and 25 mM imidazole, pH 8.0. Bound fusion protein was then eluted from the column in the presence of 500 mM imidazole, pH 8.0. Eluates were pooled, concentrated *via* a 30 kDa Amicon^®^ Ultra centrifugal filter, and then centrifuged using a high-capacity endotoxin-removal spin column (Thermo Fisher Scientific, United States) to eliminate lipopolysaccharide contamination. Recombinant protein preparations were dialyzed overnight against 1 × PBS (pH 7.4) with gentle stirring (350 rpm) at 4°C (30-kDa cutoff, Slide-A-Lyzer, Thermo Scientific™, United States), followed by size exclusion *via* a 40-kDa Zeba™ desalting spin column (Thermo Fisher Scientific, United States) to remove imidazole, and then stored at −80°C until use.

### Sodium Dodecyl Sulfate-Polyacrylamide Gel Electrophoresis and Western Immunoblot Analysis

Sodium dodecyl sulfate-polyacrylamide gel electrophoresis (SDS-PAGE) was done according to the method of Laemmli (Laemmli, 1970). Proteins were transferred to nitrocellulose membranes, which were then blocked for 2 h with 5% non-fat dry milk dissolved in 1 × TBST buffer (AmericanBio, United States), and then probed with either mouse anti-His monoclonal antibody (1:2,000 dilution; Santa Cruz Biotechnology, United States) or mouse anti-LA3490 polyclonal antibodies (1:2,000 dilution). After washing thrice with TBST, membranes were incubated for 21/2 h with alkaline phosphatase-conjugated goat anti-mouse IgG (H + L) as the secondary antibody (KPL, United States) at a dilution of 1:5,000 in TBST. Blots were developed in 5-bromo-4-chloro-3-indolyl phosphate and nitroblue tetrazolium solution (BCIP/NBT; KPL, United States).

### Asialofetuin Binding and Ricin B-Chain Competitive Binding Assays

To confirm binding specificity of the Phyre2 predicted ricin B-like lectin domain, we tested whether *E. coli*-produced, recombinant full-length, and truncated Q8F0K3 (i.e., rLA3490 and t3490, respectively) and *Leptospira* secreted VM proteins bound to immobilized asialofetuin as does ricin B.

Binding assays using rLA3490/t3490 were done using Immulon^®^ 2HB flat-bottom microtiter plates (Thermo Fisher Scientific, United States). Plates were precoated with asialofetuin (5 ng/μl in carbonate–bicarbonate buffer, pH 9.4), incubated at 4°C overnight. Prior to use, plates were blocked for 2 h at 37°C with 5% non-fat dry milk in 1 × TBST. After blocking, rLA3490, t3490, and recombinant ricin B chain (Vector Laboratories, Inc., United States) were added separately and in triplicate at molar concentrations of 0.9, 4.50, and 9.05 nM in 1 × TBST. Plates were incubated for 2 h, washed thrice with 1 × TBST, and then incubated for 1 h with anti-LA3490 polyclonal antibodies or anti-ricin B-chain monoclonal antibody, 1:1,000 in TBST (Invitrogen, United States). To quantify bound rLA3490/t3490, plates were incubated with goat anti-mouse IgG (1:5,000; KPL, United States) for 1 h, washed thrice with TBST, and developed with p-nitrophenyl phosphate (1-Step™ PNPP Substrate Solution; KPL, United States). The reaction was stopped with 2 M NaOH, and absorbance was read at 405 nm using a SpectraMax^®^ M2e Microplate Reader (Molecular Devices, United States). For competitive binding assays, precoated with asialofetuin (2.5 ng/μl) plates were preincubated with either 25 or 50 nM recombinant ricin B chain (Vector Laboratories, United States) for 2 h prior to the addition of 50 nM of rLA3490/t3490 and a final 2-h incubation. Bound recombinant protein was quantified using anti-LA3490 polyclonal antibodies.

The capacity of *Leptospira*-secreted VM proteins to bind asialofetuin was evaluated using coated Sepharose^®^ beads. Commercially available asialofetuin (1 mg/ml), (Sigma-Aldrich, United States) dissolved in 0.1 M NaHCO_3_, was coupled with PBS-washed, NHS-activated Sepharose beads (GE Healthcare, United States). The suspension was agitated slowly at room temperature for 1 h, and unoccupied NHS groups blocked with 1 M ethanolamine, pH 9 for 1 h. Washed beads were incubated with 250 μg of clarified *Leptospira* culture supernatant containing secreted proteins for 1 h, and then washed twice with MEPBS (4 mM β-mercaptoethanol, 2 mM EDTA, and 20 mM Na-phosphate, pH 7.2) buffer at 200 × *g* for 1 min. Bound proteins were eluted with 0.5 M lactose and analyzed by 4–10% bis-tris SDS-PAGE followed by Western blot with mouse anti-LA3490 polyclonal antibodies (1:2,000 dilution) as above.

### rLA3490-Mediated HeLa Cell Cytotoxicity

HeLa cells (35,000 cells/200 μl) were seeded in eight-well chamber slides (LabTek, United States) and incubated at 37°C in a humidified atmosphere containing 5% CO_2_ for 24 h. Cells were treated with a pre-optimized concentration of 45 nM rLA3490; t3490- and BSA-treated and untreated HeLa cells served as controls. Slides were incubated for up to 4 h, and timelapse images were taken at × 40 objective lens using a Leica DMi8 inverted microscope (Leica Microsystems, Germany). Adherent cells, before and after exposure to either rLA3490, t3490, or BSA or untreated HeLa cells were captured *via* a × 10 objective, and the cells were counted using LAS AF 2D quantitative image analysis software (Leica Application Suite X, LAS X; Leica Microsystems, Germany). A grayscale prefilter was applied to improve image clarity. Detachment was quantified as 100 × (# cells at 4 h/# cells at 0 h) and is reported as the average of two or more replicate experiments.

### Live/dead and LDH Assay, and F-Actin Staining

HeLa cells were exposed to 45 nM of rLA3490 or t3490 for 4 h. Monolayers were then washed twice with 1 × PBS, pH 7.4; 200 μl of 2 μM calcein AM/4 μM ethidium homodimer-1 dissolved in PBS (Live/Dead^®^ Viability Kit, Invitrogen, United States) was added to each well, and plates were incubated for 30 min in the dark. Monolayers were washed with PBS pH 7.4 to mitigate non-specific, background fluorescence. BSA and untreated HeLa cells were used as controls. Images were taken using a Leica DMi8 microscope *via* a 10 × objective with appropriate excitation and emission filters for green (live cell) and for red (dead) fluorescence. Cell lysis was quantified by assaying the concentration of lactate dehydrogenase in culture supernatants (CyQUANT™ LDH Cytotoxicity Assay, Invitrogen, United States).

For F-actin staining, cell monolayers were exposed for up to 1 h, washed twice with PBS, pH 7.4, and then fixed with 4% paraformaldehyde (Sigma-Aldrich, United States) for 30 min at room temperature. Following aspiration of the fixative, monolayers were washed twice with PBS, then 0.1% Triton X-100 in PBS was added to each well for 5 min prior to repeat washes with PBS. Monolayers were incubated with phalloidin Alexa_488 nm conjugate (Invitrogen, United States) at room temperature for 30 min in the dark per manufacturer’s directions. Nuclei were stained with 0.1 μg/ml of ProLong™ Gold Antifade Mount with DAPI for 10 min. All images were taken using a Leica DMi8 microscope with appropriate filters [Alexa_488 nm (green), DAPI (blue)] at × 40 objective lens.

### Internalization of rLA3490 by HeLa Cells

HeLa cells were seeded in eight-well chamber slides (LabTek, United States) and incubated as described above. Monolayers were treated with 45 nM of rLA3490 or t3490 for up to 60 min, washed twice with PBS, and then stained with CellMask™ Green Plasma Membrane Stain (Invitrogen, United States) per manufacturer’s directions; nuclei were stained with 0.1 μg/ml of ProLong™ Gold Antifade Mount with DAPI for 10 min. Images were taken using a Leica SP8 Gated STED 3 × super-resolution confocal microscope (Leica Microsystems, Germany) *via* a × 100 objective lens with oil immersion. Three-dimensional z projections were obtained from 24–42 cross-sectional images (depth, 6.87 μm; separation, 298.5 nm) *via* the LAS X software.

### DNA Fragmentation/Laddering Assays

DNase assays were done in a final volume of 15 μl of 1 × Tris-magnesium chloride (TM) sample buffer containing 10 mM Tris and 3 mM MgCl_2_ (pH 7.5) (Nakamura and Wisnieski, 1990) using isolated HeLa cell genomic DNA (QiAmp DNesay Blood and Tissue kit; Qiagen, Invitrogen, United States); monolayers were trypsinized when 90% confluent, and genomic DNA isolated per manufacturer-recommended protocol for nucleated mammalian cells. Recombinant proteins rLA3490 or t3490 (as negative control) were diluted in TM and allowed to equilibrate to 22°C prior to use. Triplicate reactions containing either 3, 10, 30, or 100 nM of recombinant protein were initiated with 150 ng of genomic DNA (pre-equilibrated to 22°C), terminated upon addition of premixed loading buffer, gel loading dye, purple (6×) (New England Biolabs, United States), at different time points and then analyzed by 1% agarose gel electrophoresis; gels were stained with ethidium bromide (0.5 μl/ml). Gel images were taken using Gel Doc UV illumination (Gel Logic 212 Pro, Carestream Molecular imaging, United States). Endonuclease activity was assessed using 400 ng of undigested (i.e., supercoiled) pET plasmid vector or *Hin*dIII-digested pET plasmid vector as input.

These assays were repeated using a complementary, fluorescence-based approach utilizing a dual-labeled oligonucleotide probe, labeled with fluorescein at its 5′ end and black hole quencher, BHQ-1^®^, at its 3′ end, that fluoresces intensely (excitation/emission 495/520 nm) in the presence of DNase. As before, recombinant proteins were diluted in TM (3, 10, or 30 nM), and allowed to equilibrate to 22°C for 10 min prior to use. Twenty-microliter reactions containing master mix, oligonucleotide probe, detection buffer, ROX reference dye, and 10 μl of pre-equilibrated recombinant protein (in TM buffer) were subjected to a two-step thermocycling protocol consisting of 36°C, 10 s and 37°C, 50 s for 30 cycles using a CFX96 Touch Real-Time PCR Detection System (Bio-Rad, United States). Fluorescence was recorded in 5-min increments. DNase I (0.02 units/μl) was used as positive control, and PCR grade water as negative control.

### Statistical Analysis and Image Editing

All experiments were performed in triplicate and repeated at least twice to assess reproducibility. Results are expressed as mean and standard deviation. An unpaired, two-tailed student’s *t*-test was used to assess statistical significance. Data were visualized *via* Graph Prism 8. All figures were produced using Adobe Illustrator.

## Results

### Unique Architecture of Leptospiral Virulence-Modifying Proteins

Virulent *Leptospira* spp. encode 10 to 12 multidomain VM proteins of ∼640 amino acids (aa), each containing tandem N-terminal ricin B-like lectin (RBL) subdomains: RBL1 and RBL2—collectively referred to as the carbohydrate-binding region (CBR) or lectin domain (Figure 1). The presence of these tandemly repeated RBLs is similar to the *Mycoplasma pneumoniae* community-acquired respiratory distress syndrome (i.e., CARDS) toxin (Becker et al., 2015)—although in reverse orientation (Figure 1), but the C-terminal regions of the *Leptospira* VM proteins and the CARDS toxin are not related. *Leptospira* VM proteins are contained in a single polypeptide transcribed from a single genetic locus, distinct from most other bacterial AB toxins, which are typically encoded by two or more genes—hence the A–B designation—and assembled into multimeric protein complexes (Brown et al., 1980; Nakamura and Wisnieski, 1990; McSweeney and Dreyfus, 2004; Coutte and Locht, 2015; Cherubin et al., 2018). Unique to *L. interrogans*, natural CBR deletion variants (∼313 aa) containing a predicted signal sequence are present (see below).

### Distribution and Evolution of *Leptospira* Virulence-Modifying Protein Variants

**FIGURE 2:**
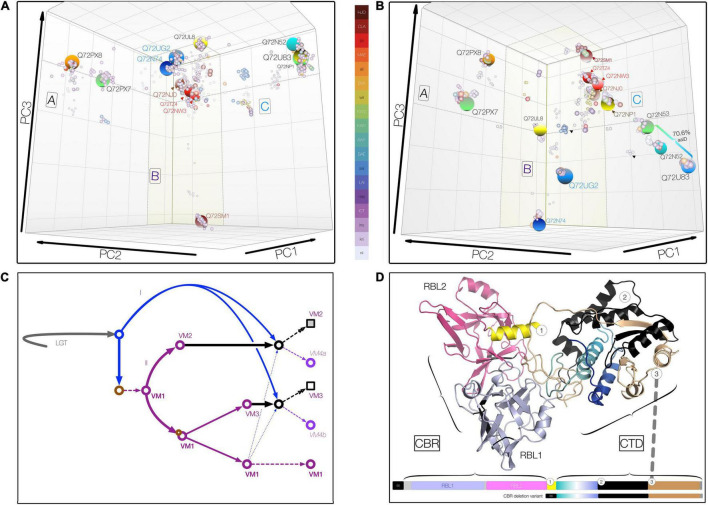
Three-dimensional metric multidimensional scaling (3DMMDS/Galaxy) plots depicting (orthologous) VM protein clusters. Clusters were identified among 940 PF07598 family VM proteins analyzed using *bios2mds* (Pele et al., 2012) and visualized using principal component analysis in R. In addition to typical PF07598 paralogs, 42 natural deletion mutants lacking both ricin B-like lectin, RBL, subdomains (i.e., containing amino terminal signal sequence and toxin domain only) were included. **(A)** Carbohydrate-binding region (CBR), containing two unidentical tandem RBL subdomains. **(B)** Carboxy terminal toxin domain (CTD) encompassing discrete trafficking and DNase subdomains. For both, initial renderings were edited (cosmetic changes only) to aid visualization by enhancing the 3D effect. No coordinates were altered. Clusters containing VM protein variants found in *Leptospira interrogans* are highlighted (large spheres) and named using the reference *L. interrogans* serovar Copenhageni strain (PMID 15028702), L1-130 (UniProtKB) protein IDs. Orthologous clusters were grouped into three superclusters comprising VM protein paralogs (A, *n* = 2; B, *n* = 7; and C, *n* = 4) based upon percent identity (PID) (Supplementary Tables 1–4). The color key uses the following convention: for species, *L. interrogans* (ins), *L. kirschneri* (kri), *L. noguchii* (nii), etc.; for serovar, e.g., Canicola (CLA), Lai (LAI), Hardjo (HJO), etc.; and for strains originating from Sri Lanka, e.g., *L. interrogans* serovar Unknown strain KW1 (KW1), etc. **(C)** Schematic showing a theoretical evolutionary history of the VM protein family, involving lateral transfer (LGT), gene duplication (purple arrows, II) and erosion (solid black arrows), and recombination (blue arrows = donor acquired *via* lateral gene transfer, I; broken arrows indicate intragenomic donor from closely related paralog). Circles represent theoretical evolving VM proteins over time; squares represent final evolved form at the current time. **(D)** Domain organization and junctions of chimeric *Leptospira* VM proteins resulting from CBR and CTD domain fusions of paralogs belonging to closely related CBR clusters, such as those related to Q72NW3 (e.g., WP.017856587.1) and Q72TZ4 (e.g., QHH71994.1) (∼99.1% PID, Supplementary Tables 1, 4). These natural VM protein variants occur infrequently (∼2%) in *L. interrogans* and its sister species, *L. kirschneri* and *L. noguchii.* Chimeric VM proteins generally share a common junction regardless of the paralogs represented.

Comparative whole-genome analyses of all (at the time) recognized pathogenic *Leptospira* spp. suggested that gene duplications and decay produced an uneven distribution of VM protein variants among the virulent group I *Leptospira*, which radiated further only in *L. interrogans*, *L. kirschneri*, and *L. noguchii* (Fouts et al., 2016). Following distance matrix computation, three-dimensional (3D) metric multidimensional scaling (MMDS) was used to define and visualize orthologous clusters using customized R scripts and bio2mds (Figures 2A,B). For consistency, clusters are named using the relevant Copenhageni UniProtKB IDs. VM protein paralogs (and their derivative CBRs and CTDs) are referenced using assigned (orthologous) cluster ID, e.g., Q8F0K3 (cluster ID, Q72UG2) (Figures 2A,B and Supplementary Tables 1–3).

To further explore the evolutionary implications of these observations, phylogenomic analysis of ∼3,000 leptospiral VM proteins derived from pathogenic *Leptospira* species was carried out. Amino acid sequences were aligned *via* hmmalign against a custom-built HMMER v3 (Potter et al., 2018) profile based upon a comprehensive full PF07598 reference alignment (Supplementary Table 4). Within the pathogenic clade of the genus *Leptospira*, VM proteins are extraordinarily diverse, comprising at least 36 discrete orthologous clusters but with important amino acid similarities (Figures 2A,B and Supplementary Tables 1–3). Clusters limited to a few leptospiral species are moderately sized containing between 5 and 50 individual VM proteins. Clusters containing *L. interrogans*-, *L. kirschneri*-, and *L. noguchii*-specific proteins are larger, ranging in size from 80 to 375 member proteins, probably reflecting a bias toward these species in accessible genomic databases.

Inspection of the pairwise distance matrices and MMDS cluster membership allows us to draw important conclusions. First, among paralogs, the N-terminal segment encompassing the CBR is more conserved ∼78% pairwise amino acid identity, Lai intragenomic range 66–99% in comparison with the CTD at ∼63 pairwise amino acid identity, Lai range, 62–72%. By contrast, the CTD is more conserved among orthologs with a minimum amino acid identity > 75%, even between distantly related species allowing easy discernment of paralogs and orthologs. Second, of the many orthologous clusters defined (Figure 2B), only one—Q72PX8—contains proteins (*N* = 375) originating from all medically important species (as well as *L. alexanderi* and *L. alstonii*). The clustering pattern around Q72PX8 likely indicates that this protein is an ancestral VM protein in Group I pathogenic *Leptospira*. Conversely, most species contain at least one defining cluster, e.g., the CBR deletion variants of *L. interrogans* (*n* = 139). Third, the scope of carbohydrate binding and toxin functionalities may vary with serovar, even among those belonging to the same species. For instance, both CBRUG2 and CTDUG2 corresponding to the CBR and CTD of Q8F0K3[UG2], respectively, are present—at one instance per genome—in *L. interrogans* serovars Copenhageni (represented by 8 strains and 98 proteins), Canicola (8 and 95), Hardjo (2 and 21), Lai (3 and 28), Manilae (2 and 26), and Pomona (5 and 62), each with > 99% amino acid identity. Whereas Lai and Copenhageni orthologs feature the expected CBRUG2//CTDUG2 combination, some strains of Canicola contain the conserved chimeric variants CBRUG2//CTDTZ4 and CBRUL8//CTDUG2. While the cognate Q8F0K3[UG2] CBR and CTD occur in alternate tandem pairings, to be considered a genuine chimera, a variant must occur in multiple genomes, and CBRs and CTDs must each share ≥ 99% amino acid identity within a designated cluster center. Such variants are more common in serovars possessing paralogs with identical or nearly identical CBRs (e.g., Q72U83 and Q72NP1 in Copenhageni) (Figures 2A,B and Supplementary Tables 2, 3). Taken together, these observations suggest that evolution of both CBR and CTD domains and their reassortment in certain lineages—likely *via* recombination—have contributed to the diversity of leptospiral VM proteins and, specifically to understanding group I pathogen adaptation to the mammalian niche (Figure 2C).

### Locating the Fusion Junction of Chimeric Virulence-Modifying Proteins: Functional Consequences

Having demonstrated that leptospiral VM proteins radiated and diversified within virulent species *via* gene duplication and lineage-dependent reassortment of CBR and CTD (Figure 2), we sought evidence to indicate whether fusion junctions of the VM protein domains were functionally conserved. To test whether all or parts of the CTD are replaced, we first identified chimeric VM proteins using complementary sequence-based approaches. Chimeric variants occur in medically important *Leptospira*, but occur only among recently diverged paralog pairs belonging to the same supercluster (Figure 2D). Only two types of chimeric variants were detected regardless of the paralogs involved, suggesting that chimeric variant development depends on some specific structural features critical to *in vivo* function. Inspection of the chimeric junctions revealed that fusion junctions are remarkably consistent (corroborated *via* comparison of multiple strains), suggesting that these replacements are functionally constrained. Accordingly, chimeric VM proteins are novel subdomain haplotypes wherein trafficking motifs present in the amino terminal CTD segment of one paralog become paired with DNase activity located in the second CTD segment of a related paralog (Figure 2D). These observations suggest that these chimeric proteins likely have altered cell-targeting competencies (relative to their cognate donors and recipients), i.e., may reflect leptospiral adaption to different hosts.

### The First Ricin B-Like Lectin Subdomain of Leptospiral Virulence-Modifying Proteins (RBL1) Has Carbohydrate-Binding Specificity Similar to That of Ricin B Chain

Because remote homology searches identified tandem ricin B-like lectin subdomains in the amino–terminal regions of leptospiral VM proteins (Figure 1), we tested the hypothesis that, like ricin B chain, leptospiral VM proteins bind to terminal galactose and N-acetyl-galactosamine residues, such as asialofetuin, a model protein (Dawson et al., 1999; Blome et al., 2010; Falach et al., 2020). Initial experiments focused on recombinant Q8F0K3—referred to here by its locus tag rLA3490—because previous studies showed that LA3490 is both highly transcriptionally upregulated *in vivo* and implicated in virulence (Lehmann et al., 2013). For consistency, all full-length recombinant VM proteins [i.e., complete CDS minus SS (Signal Sequence)] will be referred to similarly. Recombinant N-terminal truncations, e.g., of Q8F0K3, containing RBL1 alone, will be referred to as follows: t3490 for truncated LA3490. For experiments using native VM proteins, *Leptospira* cells were grown under conditions mimicking the internal host environment to promote virulence gene expression *in vitro* (Matsunaga et al., 2005; Lo et al., 2006; Matsunaga et al., 2007).

**FIGURE 3:**
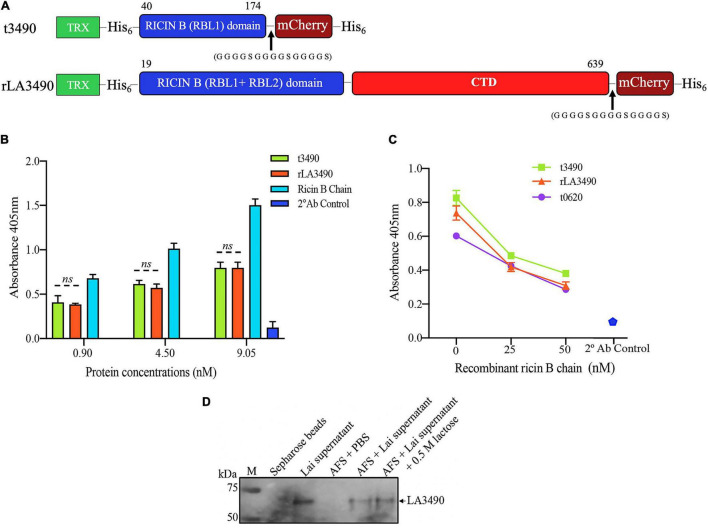
VM Protein LA3490 is a *bona fide* R-type lectin. **(A)** Schematic depicting the organization of the recombinant mCherry (mC) fusion proteins used in the current study; t3490, amino acid positions 40–147 aa (minus SS, signal sequence); and rLA3490, 19–639 aa, also lacking SS. Recombinant fusions also include a glycine–serine (GGGGSGGGGSGGGGS) linker and C-terminal His_6_ tag (purification), and N-terminal thioredoxin. RBL and CTD denominates ricin B-like lectin and carboxy terminal domain, respectively. **(B)** Asialofetuin-binding assay demonstrating that truncated (t3490) and full-length (rLA3490) VM proteins bind to asialofetuin in a dose-dependent manner similar to commercially available ricin B chain. **(C)** Competition assay showing that truncated (t3490), the ricin B domain of another VM protein, LA0620 (t0620), and full-length (rLA3490) compete for the same binding site as recombinant ricin B chain (25 nM and 50 nM). Assays were performed in microtiter plates using an ELISA format. Mouse polyclonal anti-LA3490 and anti-LA0620 antibodies (1:1,000 dilution) were used as primary and anti-mouse IgG as secondary antibody (used alone as a specificity control, labeled as 2 Ab control). **(D)** Native LA3490 (70.29 kDa) secreted by *L. interrogans* serovar Lai into EMJH culture supernatant in the presence of 120 mM NaCl binds to asialofetuin-coupled Sepharose beads (AFS). Proteins were eluted with 0.5 M lactose. Unconjugated Sepharose beads incubated with *L. interrogans* serovar Lai-conditioned medium, and AFS beads with PBS served as controls. Assays were run in triplicate, and experiments were repeated twice. The mean absorbance (± SEM) was visualized in GraphPad Prism 8 and considered statistically significant at *p* < *0.05.* The blot shown in panel **(D)** was cropped from the full blot shown in Supplementary Figure 3.

Recombinant VM proteins were expressed in *E. coli* as N-terminal fusions with thioredoxin-His_6_ (TRX) to improve solubility. The C-terminal were fused with mCherry-His_6_ to facilitate affinity purification and visualization of the protein using fluorescence microscopy (Figure 3A). Western immunoblot, which was shown to be endotoxin-free by a limulus amebocyte lysate assay, ruling out the potential cytotoxicity of contaminating lipopolysaccharide (Supplementary Figure 1). LA3490 and t3490 both bound to asialofetuin (Figure 3B). VM proteins have similar carbohydrate-binding specificity to ricin B chain as determined by competitive asialofetuin-binding assays (Sehnke et al., 1994; Dawson et al., 1999; Blome et al., 2010; Figure 3C). As predicted based upon the presence of a predicted secretory signal, Q8F0K3[rLA3490]—and likely other VM proteins—was found as a soluble protein in *Leptospira*-conditioned medium, with such secretion inducible by physiologic osmolarity and temperature. Solid phase-binding assays using asialofetuin-conjugated Sepharose beads confirmed that Q8F0K3 bound to asialofetuin (Figure 3D). Like recombinant ricin B, asialofetuin-bound Q8F0K3 could be eluted using 0.5 M lactose, further supporting the conclusion that *Leptospira* VM proteins have *bona fide* ricin B-like lectin carbohydrate-binding activity.

These observations indicate that the CBD (RBL1) of LA3490 and, by analogy, CBDs of other VM proteins, are *bona fide* R-type lectins.

### Dose-Dependent Cytotoxicity of rLA3490, on HeLa Cell Monolayers

Having confirmed that the RBL1 is an R-type lectin, we hypothesized that VM proteins are cytotoxins with effects mediated by the as-yet uncharacterized C-terminal region.

**FIGURE 4:**
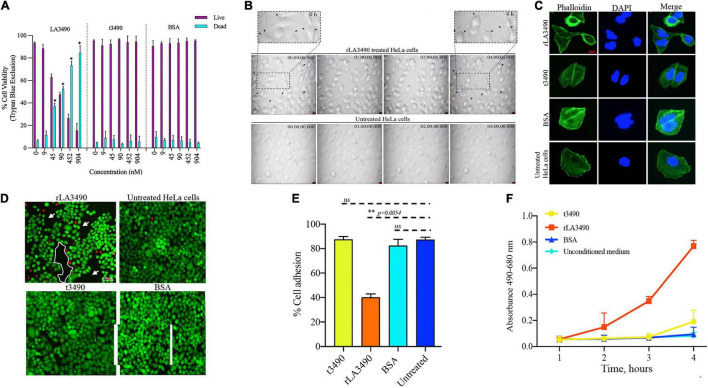
Cytopathic effect of rLA3490. **(A)** Dose-dependent HeLa cell death induced by r3490 as assessed by trypan blue dye exclusion. Negative controls, t3490, BSA, and no treatment, had no such effect. Cell monolayers were treated with graded molar ratio doses (0 to 904 nM) of LA3490, t3490, and BSA for 4 h. Data represent mean ± SD of two independent experiments done with each condition in triplicate (paired *t*-test, *p < *0.005*). **(B)** Time-lapse phase-contrast microscopy images (40 frames, 5 s intervals) showing HeLa cell cytopathic effect following exposure to 45 nM of rLA3490 and controls. Only with rLA3490 was cell blebbing evident from 1 h onward [seen in zoom view (top left and right panel, black arrow)]. Time-lapse imaging was captured using a × 40 objective lens using a Leica DMi8 inverted microscope. Scale bar, 10 μm. **(C)** Actin depolymerization occurs early after rLA3490 treatment. HeLa cell monolayers were incubated with 45 nM of rLA3490, t3490, and BSA up to 1 h. Monolayers were fixed with 4% paraformaldehyde followed by 0.1% Triton X-100 in PBS permeabilization. The monolayer was incubated with phalloidin-Alexafluor-488 nm conjugate, washed, and then mounted with ProLong™ Gold Antifade Mountant with DAPI. Images were captured using a Leica DMi8 confocal microscope [Alexa_488 nm (green), DAPI (blue)] at × 40 magnification. Untreated HeLa cells served as control. Scale bar, 20 μm. **(D)** HeLa cell death induced by rLA3490 as assessed by fluorescent live/dead staining. Negative controls (t3490, BSA, and no treatment) had no such effect. Live/dead staining of HeLa cell monolayers was carried out after 4-h exposure to 45 nM rLA3490 (top left panel) and t3490 (bottom left). A dramatic decrease in adherent cells and concomitant accumulation of dead cells upon treatment with rLA3490, but not t3490 or BSA, was observed. Images were captured at × 10 magnification using a Leica DMi8 inverted microscope. Scale bar, 100 μm. **(E)** Quantification of LA3490–induced detachment of HeLa cells from the monolayer following 4-h exposure, compared with negative control exposure (t3490, BSA, and no treatment). Cells were visibly dissociating from the monolayer after 1 h of rLA3490 exposure. **(F)** Quantification of time-dependent HeLa cell death by lactate dehydrogenase release after treatment with rLA3490 in comparison with negative controls. Groups were compared using the one-way *t*-test in GraphPad Prism 8 and considered statistically significant at *p* < *0.05*; ns, non-significant. **means statistically significant with *p* = 0.0054.

Exposure of HeLa cells to rLA3490 induced dose-dependent cytopathic effect and HeLa cell monolayer destruction (Figure 4), as demonstrated by trypan blue exclusion, phase-contrast microscopy (including time-lapse microscopy video, Supplementary Movie 1), fluorescent live/dead staining, and release of lactate dehydrogenase. Actin depolymerization (Figure 4C) and caspase activation (Supplementary Figure 2) were observed. No such changes were observed with negative controls, including t3490, bovine serum albumin (BSA), and no treatment, confirming that cell death was a direct result of rLA3490 treatment and not to fusion protein affinity/epitope tags or an artifact of the culture conditions.

### Ricin B-Like Lectin 1 Alone Is Sufficient for Attachment to the HeLa Cell Surface but Not for Internalization

**FIGURE 5:**
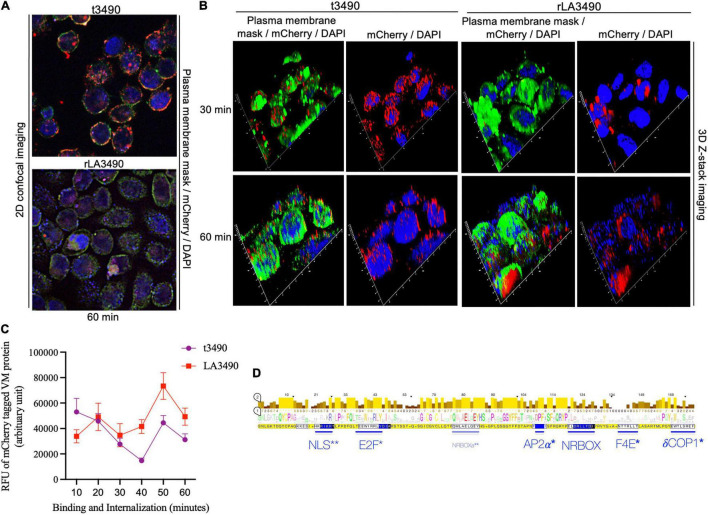
Surface binding and nuclear localization of rLA3490 in HeLa cells. Fluorescent confocal microscopy demonstrating kinetics of binding of mCherry–rLA3490 and mCherry–t3490 fusion proteins binding to HeLa cells. **(A)** Two-dimensional view, at 60 min, t3490 is visible only on the cell surface (red); rLA3490 is internalized by 60 min (red/pink). **(B)** Three-dimensional Z-stack and orthogonal images obtained by high-resolution fluorescent confocal microscopy showing internalization of mCherry–rLA3490 fusion from 30 min onward, with nuclear translocation and chromosomal degradation (shown by patchy DAPI staining, lower right) evident within 60 min. t3490 remained on the cell surface at 30 and 60 min. Visualization of treated cells was done after staining with CellMask™ green plasma membrane stain mounting with ProLong™ Gold Antifade Mountant + DAPI nuclear stain. Images were captured using an oil immersion × 100 objective using appropriate filters (blue, DAPI; green, plasma membrane; red, mCherry fusions). **(C)** Time-dependent interactions of mCherry-tagged rLA3490 and t3490 proteins with HeLa cells (surface binding plus internalization). Fluorescent confocal microscopy (using ImageJ version 1.53 software) was used to quantify recombinant fusion proteins with HeLa cell monolayers. Monolayers were exposed to 45 nM recombinant fusion proteins or controls up to 60 min. Fluorescence intensities of mCherry–t3490 and –LA3490 fusion proteins were measured in 10-min intervals from 0 to 60 min. Data were visualized in GraphPrism 8. **(D)** Eukaryotic trafficking and protein–protein interaction motifs found in *Leptospira* VM proteins. Consensus amino acids in larger letters indicate fully conserved residues. Statistically well-supported motif mimics (*p* < 10^–3^) were identified *via* the Eukaryotic Linear Motif resource (http://elm.eu.org). Those predicted to be involved in intracellular trafficking (*) and nuclear translocation (**) are shown in blue. Bars indicate location and length, and consensus sequences are boxed. NLS, nuclear localization signal. NRBOX, nuclear receptor box (or LxxLL) motif confers binding to nuclear receptors and is present in the C-terminal domain of all VM protein paralogs [including the natural *L. interrogans* deletion variant, Q72N53 (LA0591 cluster ID)]. Q72UG2 (LA3490) contains an additional LxxLL motif depicted as NRBOXa. E2F, LxxLFD motif characteristic of E2F family transcription factor. AP2*a*, DPF/W motif binds alpha and beta subunits of AP2 adaptor complex. F4E, variant YxxxxL motif mediates binding to the dorsal surface of eukaryotic translation initiation factor, eIF4E. *d*COP, di-tryptophan motif predicted to mediate retrograde trafficking from Golgi to the endoplasmic reticulum.

Internalization and/or intracellular trafficking of rLA3490– and t3490–mCherry fusion proteins were monitored using super-resolution flouresent confocal microscopy (Figure 5). Both fusion proteins bound to the cell surface, but only rLA3490 internalized and localized to the cell nucleus (Figures 5A,B). While RBL1 alone was sufficient for binding—both to immobilized asialofetuin (above) and at the HeLa cell surface—internalization depended on protein folds beyond the RBL domains. Binding of both rLA3490 and t3490 at the cell surface occurred 30–60 min after treatment, with internalization, translocation, and nuclear fragmentation evident from 30 min onward (Figure 5B, right panels). Maximal accumulation of rLA3490 was dependent on the full-length protein-dependent internalization; maximum binding and accumulation of t3490 was at 10 min, while rLA3490 continued to accumulate in HeLa cells (Figure 5C). t3490 bound to the surface of HeLa cells but was not internalized (Figure 5B, left panels), and was not cytotoxic. Animated orthogonal (Supplementary Movies 2–5) and z-stacks (Supplementary Movies 6–9) showing binding and internalization of rLA3490 and/or t3490 by HeLa cells at 30 and 60 min.

### Leptospiral Virulence-Modifying Proteins Have *in vitro* Endo- and Exo-DNase Activity

**FIGURE 6:**
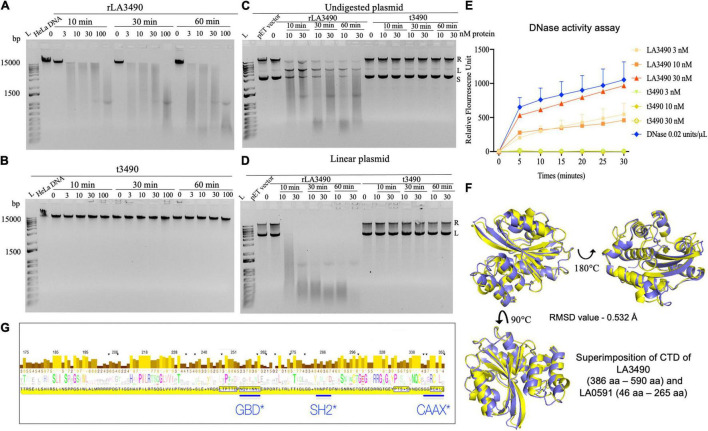
DNase activity of leptospiral VM proteins. **(A)** DNase activity of rLA3490 observed upon incubation of 150 ng of DNA from HeLa cells in TM buffer containing 3 mM Mg^2+^ for indicated dose and time (absence of Mg^+2^ in reaction yielded no DNA degradation). Samples were subjected to 1% agarose gel electrophoresis. The DNase activity rLA3490 is indicated by smearing and disappearance of DNA; t3490 had no such effect. **(B)** Other recombinant VM proteins (LA0620, LA1400, LA1402, and LA0591) all had similar DNase activity (not shown). **(C)** DNase activity of rLA3490 on 400 ng of undigested plasmid pET28 shows partial degradation with uncoiling, linearizing, and partial degradation, and unaffected by t3490, shown by the white arrow. **(D)** DNase activity of rLA3490 on linearized plasmid shows complete disappearance of linear and relaxed plasmid, with dose- and time-dependent smearing. L, DNA ladder. **(E)** Quantification of rLA3490 DNase activity using real-time PCR and a FAM fluorescence probe. Bovine DNase, 0.02 U/μl, was used as positive control. Data represent the mean ± SD of three independent experiments. **(F)** Superimposition of AlphaFold generated CTD of LA3490 and LA0591, respectively. While LA3490 represents the vast majority of VM proteins with two RBLs, a CTD, and intervening functional sequences as visualized in Figure 1A, LA0591 lacks RBL1 and RBL2 but contains the rest of the functional sequences. This paralog represented by LA0591 is only fully present in *L. interrogans* species but not in other pathogenic Group I *Leptospira.* The CTDs of LA3490 and LA0591 are predicted to be highly conserved at the structural level despite amino acid sequence divergence, as shown by the RMSD values of 0.532 Å. **(G)** Eukaryotic trafficking motifs are present in the DNase-containing CTDs of leptospiral VM protein paralogs. GBD, GTPase-binding domain ligand, which is an amphipathic α-helix found in the C-terminal VCA segment of WASP/N-WASP proteins. SH2, Src Homology 2 domain, which is a broadly conserved protein interaction module central to tyrosine kinase signaling, and found in ubiquitin ligases, transcription factors, and guanine nucleotide exchange factors. The canonical CAAX motif is found in most *Leptospira* VM protein paralogs (but neither Q72UG2 nor Q72N53).

Having demonstrated that rLA3490 localizes to HeLa cell nuclei leading to chromosomal fragmentation, we hypothesized that Q8F0K3 (and, by extension, all VM proteins) possesses DNase activity that might contribute to the mechanism of cell death. Cell-free assays demonstrated that rLA3490 has potent, dose-dependent DNase activity on purified HeLa cell genomic DNA (Figure 6A), and dose-dependent nicking, endo-, and exonuclease activity on supercoiled plasmid DNA producing relaxed and linearized plasmid (Figures 6C,D). Linearized plasmids were completely digested (Figure 6D) confirming exo-DNase activity comparable with that of recombinant bovine DNase I (Figure 6E). Other recombinant VM proteins, LA0591 [serovar Lai CBR deletion variant (Figure 6F)], rLA0620, rLA1400, and rLA1402 also exhibited exo-DNase activity (data not shown). Negative controls, t3490 (and t0620, not shown), had no detectable DNase activity (Figure 6).

### Sequence, Structural Similarity of Leptospiral Carboxy Terminal Domains of LA3490 With Bovine DNase I

**FIGURE 7:**
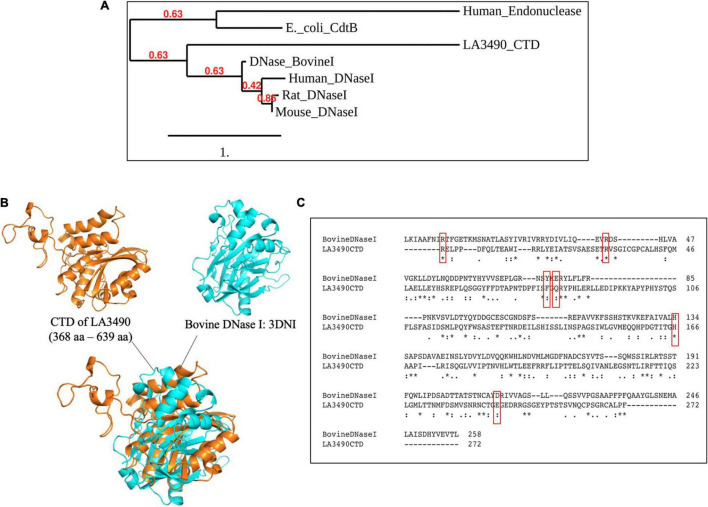
CTD of LA3490 possess conserved active site residues identical to bovine DNase I. **(A)** Phylogenetic tree based on amino acid sequence alignment from CTD of LA3490, bovine DNaseI (uniport ID: P00639), mouse DNase1 (P49183), rat DNase1 P21704, human DNaseI (P24855), *E. coli*_CdtB (Q46669), and human endonuclease (P27695) was generated using https://www.phylogeny.fr. Scale bar, one substitution per amino acid site. Numerals indicate the statistical reliability of the branching order as determined by bootstrap analysis of 100 alternative trees. **(B)** Superimposition of CTD of LA3490 (368–639 aa) and bovine DNase (PDB: 3DNI) are predicted structural similarity in the active sites of bovine DNaseI with RMSD of 9.012 Å. **(C)** Clustal Omega-based multiple sequence alignment of CTD of LA3490 and bovine DNaseI (P00639) showing percentage identity of 20.18%. Red boxes represent the catalytic active sites in bovine DNaseI, which are highly conserved in CTD of LA3490. The ‘*’ indicates identical amino acids in the multiple sequence alignment.

After experimental demonstration of DNase activity in recombinant full length VM proteins, we performed a new sequence alignment and phylogenetic relation analysis focused on comparing the CTD of VM protein amino acid sequences with various mammalian DNases and the *E. coli* cytolethal distending toxin (CdtB) sequences. These analyses were consistent with the *Leptospira* VM proteins having identifiable C-terminal DNase domains. In attempts to superimpose the CTD of LA3490 with bovine DNase I, there was structural resemblance specifically in the active site, but only an RMSD value of 9.012 Å was obtained because the overall structural similarity was low outside of the predicted active site (Figure 7).

### Leptospiral Virulence-Modifying Protein Structure–Function Relationships

Based on the demonstration of VM protein cytotoxic and DNase activity, we sought to better understand the diversity of these proteins and their radiation among virulent *Leptospira* spp. HHpred homology searches indicated that RBL1 and RBL2 share high sequence and structural homology with D2 and D3 of CARDS (Becker et al., 2015) (E = 2.7*e*^–96^) and appear to be functionally analogous. AlphaFold structural modeling suggests that VM protein CTDs appear to contain functionally distinct subdomains that explain experimentally demonstrated intracellular trafficking, cytotoxicity, and DNase activity (Figure 1).

All VM CTDs, including CBR deletion variants, contain several statistically well supported (i.e., *p* < 1e^–4^) human Short Linear Motif (SLiM) mimics (Samano-Sanchez and Gibson, 2020), identified using the Eukaryotic Linear Motif (ELM) resource, that likely facilitate VM protein trafficking and nuclear translocation (Figure 5D) including a nuclear localization signal, NLS. Of 13 *L. interrogans* paralogs, only CBR deletion variants share a common NLS with another paralog, likely reflecting their evolutionary origins. Most members of a given ortholog cluster belonging to the same species contain an invariant NLS. Compared with its flanks, the NLS-containing subdomain (14 aa) is especially variable suggesting strong selection. Following this subdomain are consecutive amphipathic α-helices, containing a putative LxxLL nuclear receptor motif [LIG_NRBOX(ELME000045): ^451^HLERLLE^457^, and a SH2-binding motif a LIG_SH2_SRC(ELME000474): YxxxΦ (^385^YEIAT^389^)]. Also, present is an LxxLFD motif characteristic of EF2 transcription factors (absent from Q8F0K3) and a single LIG_AP2alpha_2 mimic (^440^DPF^442^) for binding accessory endocytic proteins (ELME000190). Q72UG2 cluster members, including Q8F0K3, contain multiple distinct SLiM mimics, including a second LxxLL motif (^413^MLAELLE^419^).

A pLxIS motif (^536^PILRIS^541^) may mediate immune escape *via* interference with IRF-3-dependent signaling and an adaptin-binding endosome–lysosome–basolateral sorting signal (*p* < 8.4*e*^–5^): TRG_DiLeu_BaEn_3[ELME000525] and ^557^EEFRRFL^563^ (Figure 6G).

VM protein C-termini appear to be disordered and are usually terminated by a CAAX motif (i.e., MOD_CAAXbox[ELME000059], *p* < 2.4*e*^–6^) reminiscent of the F-box proteins produced by the intracellular bacterium *Legionella pneumophila* (Perpich et al., 2017). These motifs act as prenylation substrates and usually mediate membrane attachment. Q8F0K3[UG2] and Q8F6G6[CBR deletion variant] lack a canonical CAAX box but are terminated by a LIG_PDZ_Class_2[ELME000091] motif: ^634^RCALPF^639^ (*p* < 7.9*e*^–5^), for binding of PDZ domains, which are globular protein modules found in eukaryotic regulatory proteins.

## Discussion

Here we demonstrate that *Leptospira* virulence-modifying (VM) proteins, epitomized by LA3490 (Q8F0K3), are *bona fide* R-type lectin domain-containing cytotoxins—the first experimentally validated *Leptospira* exotoxins. rLA3490 binds to, and is quickly internalized by, HeLa cells *via* an N-terminal R-type lectin domain with specificity for terminal galactosyl residues. After binding/internalization, it is translocated to the HeLa cell nucleus *via* a nuclear targeting signal and twin LxxLL motifs for nuclear receptor binding. Cell surface binding and internalization were shown to be rapid, occurring within 30 min after exposure. rLA3490 produced pleiotropic effects on HeLa cells, including actin depolymerization, caspase-3 activation, nuclear fragmentation, and ultimately blebbing and cell death. One mechanism of cell death appears to be originated with genomic DNA degradation, which occurs after nuclear localization of the VM protein. Corroborating *in vitro* experiments using purified HeLa cell genomic DNA, and supercoiled and linearized bacterial plasmid DNA indicates that rLA3490, and at least four other VM proteins tested so far, possess endo- and exo-DNase activities.

Most VM proteins, with the exception of the CBR deletion variants, fit the classical AB toxin paradigm (Odumosu et al., 2010). The entire leptospiral VM protein gene contains domains that are commonly encoded by two or three separate genes in other bacteria. VM proteins have at least two functionally distinct regions, with the N-terminal partly responsible for host cell targeting (binding and internalization) and the C-terminal partly mediating cytotoxicity (intracellular trafficking/enzymatic activity). The N-terminal segment is reasonably well conserved among *Leptospira* serovars (∼78% average pairwise amino acid identity) and contains a confirmed R-type lectin domain (amino acid positions 40–174) that shares binding specificity with ricin B chain for terminal galactosyl residues of glycoproteins. In contrast, the C-terminal segment is less conserved (∼63% average pairwise amino acid identity) and appears to mediate cytotoxicity. This sequence diversity is thought to influence VM protein cell targeting specificity (i.e., successful binding/internalization and intracellular trafficking) rather than catalytic activity, as other VM proteins tested so far exhibit DNase activity *in vitro*. Despite the evidence of expanded family clusters of paralogs (Figure 2), we cannot yet speculate on the reasons for the diversification of the PF07598 gene family, although one leading hypothesis is that paralog expansion has enabled adaption of different *Leptospira* to different hosts. Further experimentation and *in silico* analysis to compare VM protein structure and function is needed to determine any possible sequence motifs that might indicate virulence differences among PF07598 family members.

Comparison of intragenomic distances has revealed that the expanded VM proteins repertoire in virulent group I pathogenic *Leptospira* arose from a series of gene duplication events followed by autonomous evolution of N- and C-terminal segments, occurring more rapidly in the latter. Based upon available data, it appears that an initial duplication event produced LA1402//LA1400, which constitute the only *L. interrogans* VM proteins identified so far with close orthologs in less virulent group I pathogenic species (Fouts et al., 2016). This initial event was followed by successive duplications likely originating from LA1400 that formed three discrete gene clusters [A, B, and C (Fouts et al., 2016)], the largest comprising seven VM protein-encoding genes, including LA3490 and LA0620. Some serovars have seemingly lost specific VM protein genes, e.g., LICRS03300 from serovar Lai and LA3271 from Hardjo, whereas others contain various CBR deletion variants. As far as we know, this uneven distribution of VM proteins among *Leptospira* serovars and their implied differences in host cell-targeting specificity is the first definitive evidence that some are intrinsically more virulent than others and, therefore, of heightened clinical and public health significance. Most VM protein-encoding genes are found only in *L. interrogans* and its sister species. As some VM proteins, e.g., LA3490 (Q8F0K3) have proven to be particularly toxic to human cells, their presence in serum could foreshadow severe disease complications, providing prognostic information—a cornerstone for effective clinical risk assessment.

Like ricin, for which toxicity and pathology are clearly linked and route dependent (inhalation and severe respiratory compromise being most lethal), site-specific expression of certain VM proteins coupled with their presumed differences in host cell specificity (i.e., host cell exposure and susceptibility) might explain the variable clinical presentation of severe leptospirosis. Indeed, the effort to understand the molecular and cellular pathogenesis of leptospirosis remains in its infancy, and approaches to prevent leptospirosis or ameliorate its pathogenesis are predicated on mechanistic understandings of the biology of *Leptospira*–host interactions. For example, pulmonary hemorrhage and refractory shock are particularly important clinical manifestation of leptospirosis (Sehgal et al., 1995; Marotto et al., 1999; Segura et al., 2005; Gouveia et al., 2008; Truong and Coburn, 2011; Helmerhorst et al., 2012; Ruwanpura et al., 2012). Indirect evidence—that these serious manifestations are ameliorated by hemodialysis/hemofiltration (Andrade et al., 2007; Cleto et al., 2016)—suggests that there may be a circulating soluble toxin or toxins in leptospirosis. Histopathological analysis of lung tissues in severe pulmonary leptospirosis syndrome do not find intact *Leptospira* (Nicodemo et al., 1997), but rather damage to alveolar epithelial and activation of endothelial cells, with deposition of immunoglobulin and complement as secondary events (Nally et al., 2004; Croda et al., 2010; De Brito et al., 2013). Nonetheless, apart from various sphingomyelinases/hemolysins (Narayanavari et al., 2015; Chaurasia and Sritharan, 2020) and a collagenase (Kassegne et al., 2014), a few potential leptospiral toxins have been identified, and none adequately explain the pathogenetic features of the diverse clinical spectrum of leptospirosis. Nonetheless, hemodialysis and hemofiltration remain life-saving interventions but exceed the clinical resources and/or capabilities in the vast majority of leptospirosis-endemic regions. By contrast, as with other AB toxins (Odumosu et al., 2010), e.g., CARDS (Somarajan et al., 2014; Becker et al., 2015) and ricin (Yermakova et al., 2014; Gal et al., 2017), mitigating VM protein toxicity using monoclonal antibody (mAb)-based biologics or small molecule inhibitors (Benz and Barth, 2017) that perturb VM protein cell-surface binding/cell entry and/or intracellular trafficking/toxicity would constitute more generally accessible alternatives.

The data we present here build upon our previously published observations (Matsunaga et al., 2007; Lehmann et al., 2013; Fouts et al., 2016) indicating that leptospiral VM proteins are major virulence factors presumably involved in the molecular and cellular pathogenesis of leptospirosis. Transposon mutagenesis screens have since shown that multiple VM proteins, particularly Q8F6G8 (gene ID, LA0589), contribute to lethal disease in hamsters (Murray et al., 2009; Truong and Coburn, 2011). Nonetheless, until now, VM proteins were uninformatively classified as PF07598, a protein family of unknown function. Here, we confirm Phyre2-based predictions that these proteins belong to a superfamily of proteins, the R-type lectins, which possess carbohydrate-binding activity named for and structurally similar to ricin B chain, and are found in plants, animals, and bacteria (Cummings and Schnaar, 2017). Ricin and its B chain (and other R-type lectins) bind to terminal galactoses or other related glycans of a diverse range of host cell surface glycoconjugates, which facilitates translocation and internalization of the ricin A chain into target cells, resulting in cell death *via* inhibition of protein synthesis (Montanaro et al., 1973; Sperti et al., 1973; Lord et al., 2003; Sowa-Rogozinska et al., 2019). Likewise, bacterial AB toxins, such as Shiga, pertussis toxins, and diphtheria toxins, mediate cell death either by ADP-ribosylation of 28S rRNA, or α_i_ subunits of the heterotrimeric G protein or by inactivation of elongation factor 2 (Brown et al., 1980; Cemal, 1999; Coutte and Locht, 2015; Cherubin et al., 2018). Genotoxins like *Leptospira* VM proteins, e.g., cytolethal distending toxin (CDT), are less well studied. With our characterization of *Leptospira* VM proteins, some general features of bacterial genotoxins have come into focus. For example, they exhibit DNase activity, are translocated to the nucleus *via* a nuclear localization signal (McSweeney and Dreyfus, 2004), and have pleiotropic effects on target cells, including causing cell death *via* caspase 3-dependent and -independent mechanisms (Ohara et al., 2008).

By recontextualizing the data presented here within the classical AB toxin paradigm, we propose that following cell surface binding—possibly *via* mannose receptor, as has been reported for ricin B (Simmons et al., 1986; Lord et al., 1992; Newton et al., 1992) given overlapping VM protein–ricin B carbohydrate-binding specificities, VM proteins are endocytosed—as are ricin and most AB toxins, e.g., Shiga toxin (Arfilli et al., 2010); released to the cytoplasm then ferried to the nucleus *via* an internal nuclear localization signal gaining entry after binding at the nuclear pore complex *via* one or more LxxLL motif-containing amphipathic α-helices; and then actively translocated through the pore into the nucleoplasm, leading to nuclear fragmentation *via* intrinsic exonuclease activity; possibly inducing caspase-3 activation and cytoskeleton disassembly *via* yet unknown mechanisms during transit, when free in the cytosol.

While we demonstrated that ricin B-like lectin domains of distinct VM proteins (rLA3490, t3490, and t0620) bound to immobilized asialofetuin (Wales et al., 1994; Frankel et al., 1996; Dawson et al., 1999), native target ligands and cellular targets are yet to be defined. Second, differences in cytopathic potential among VM proteins, their host cell targeting specificities, and molecular pathways by which they exert their pleiotropic effects remain to be fully explored, although these initial experiments indicate that they are likely AB-type cytotoxic genotoxins. Third, while it is becoming increasingly evident that leptospiral VM proteins arose from successive gene duplication events, we do not yet fully understand the reasons for the expanded repertoire in *L. interrogans*, *L. kirschneri*, and *L. noguchii* (Lehmann et al., 2013) compared with other pathogenic *Leptospira* species (Lehmann et al., 2013; Fouts et al., 2016), nor for the uneven distribution of VM paralogs among *Leptospira* serovars types, although yet unknown ecological niche specialization is quite plausible, presumably as a defense against eukaryotic predation in soil/surface water, akin to Shiga toxin production in *E. coli* (Lainhart et al., 2009).

## Data Availability Statement

The datasets presented in this study can be found in online repositories. The names of the repository/repositories and accession number(s) can be found in the article/Supplementary Material.

## Author Contributions

RC, MM, and JV conceptualized and designed various, respective contributions to the study. JV was the overall conceptualizer. RC performed the experiments and wrote the first draft of the manuscript. AM analyzed and organized the confocal microscopy data. MM performed the computational analysis, generated the data, and wrote sections of the manuscript. JV supervised and by mentoring RC, contributed to the experimental studies, obtained funding, and wrote the manuscript. All authors contributed to the manuscript revision, read, and approved the submitted version.

## Conflict of Interest

JV and spouse have an equity interest in LeptoX, which may have a future interest in licensing this work. The work reported here has been filed in patent applications from Yale University. The remaining authors declare that the research was conducted in the absence of any commercial or financial relationships that could be construed as a potential conflict of interest.

## Publisher’s Note

All claims expressed in this article are solely those of the authors and do not necessarily represent those of their affiliated organizations, or those of the publisher, the editors and the reviewers. Any product that may be evaluated in this article, or claim that may be made by its manufacturer, is not guaranteed or endorsed by the publisher.

## Footnotes

1
http://www.sbg.bio.ic.ac.uk/phyre2

2
https://heliquest.ipmc.cnrs.fr

3
http://elm.eu.org

4
https://toolkit.tuebingen.mpg.de/tools/hhpred

5
https://toolkit.tuebingen.mpg.de/tools/ali2d
