# Supplementary figures and images for: Pathogenic Leptospira Evolved a Unique Gene Family Comprised of Ricin B-Like Lectin Domain-Containing Cytotoxins
Source: Front Microbiol. 2022 Mar 29;13:859680. doi: 10.3389/fmicb.2022.859680 (PMC9002632; doi:10.3389/fmicb.2022.859680)

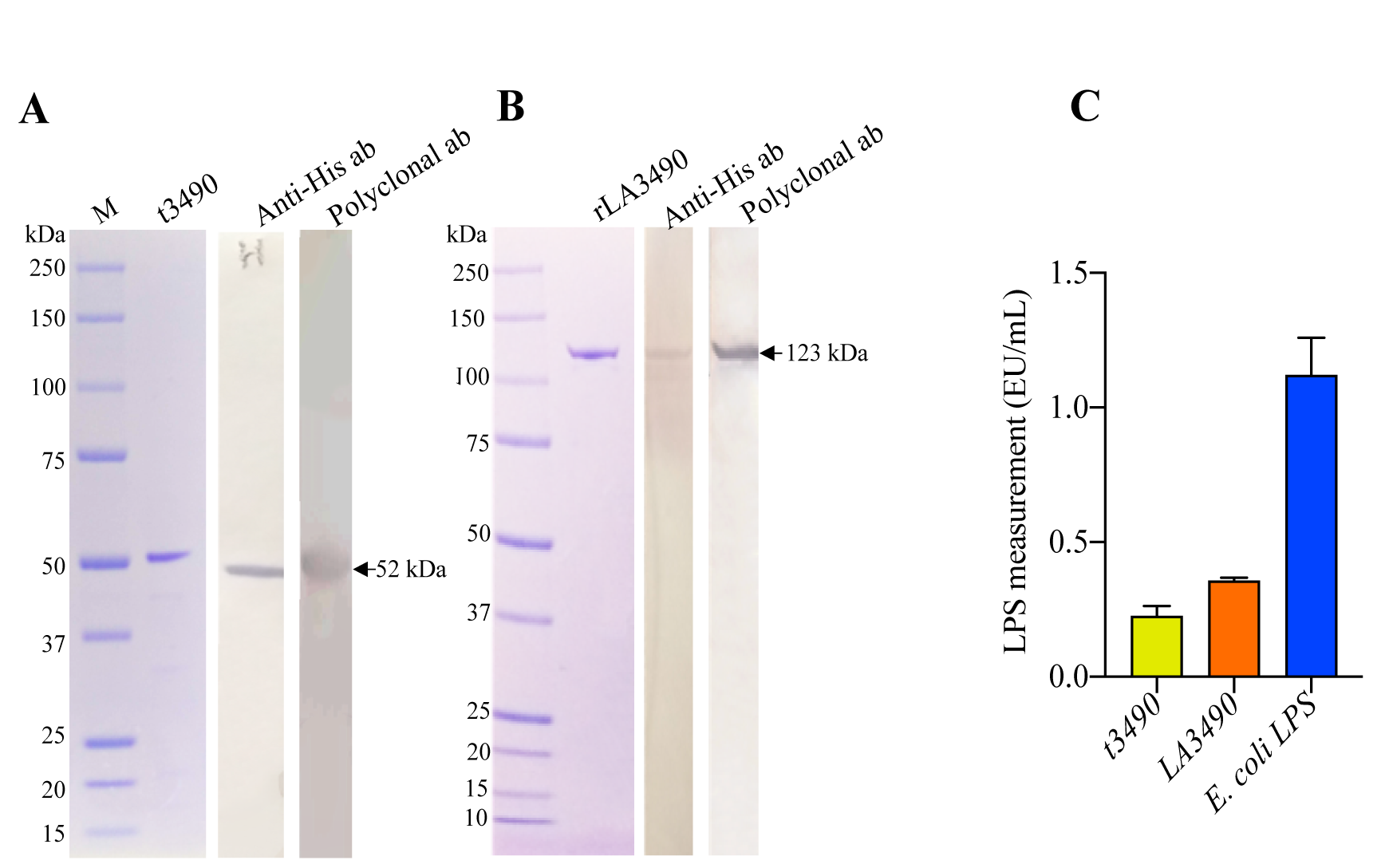

Supplement: Supplementary file 1 [file Image_1.TIF]

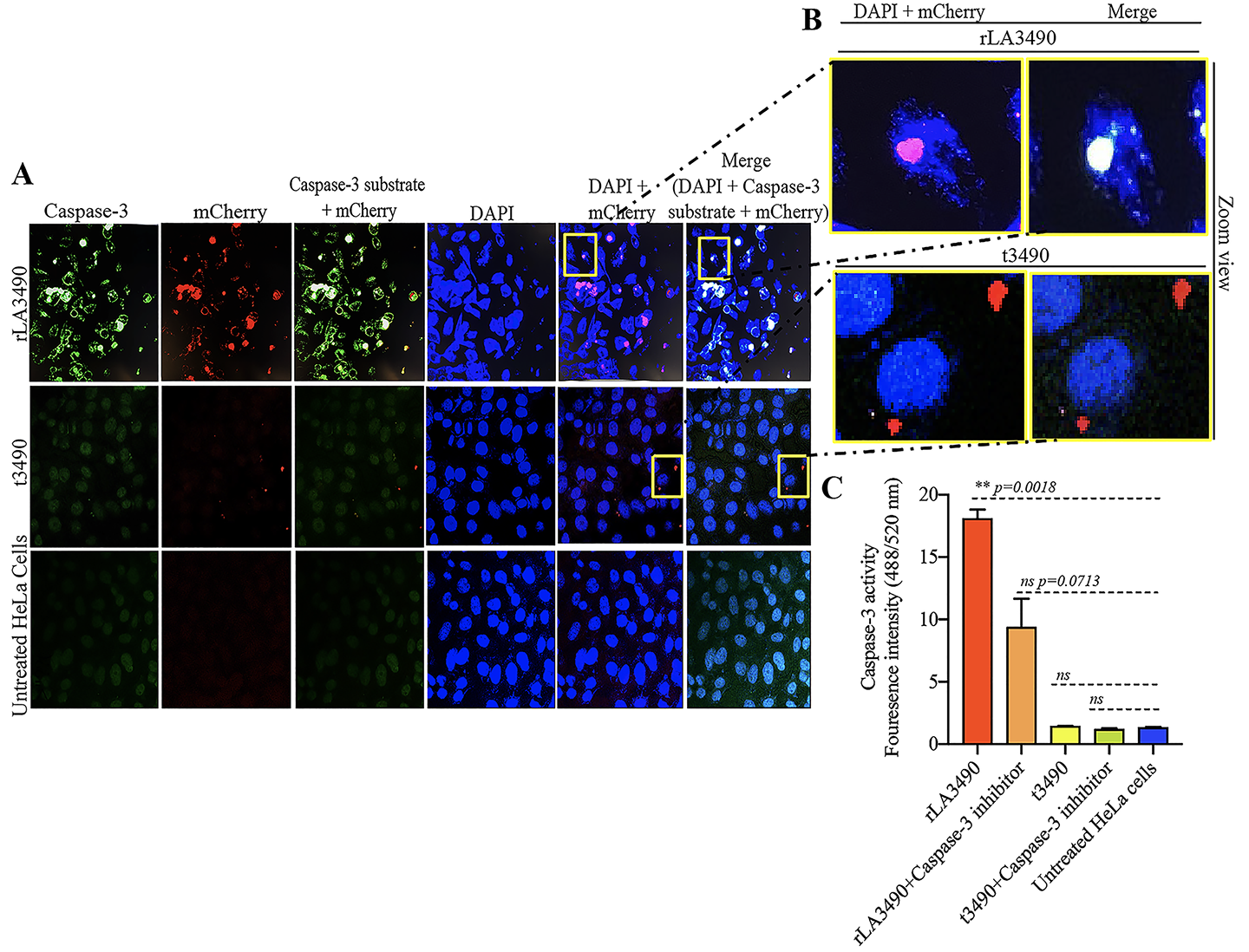

Supplement: Supplementary file 2 [file Image_2.TIF]

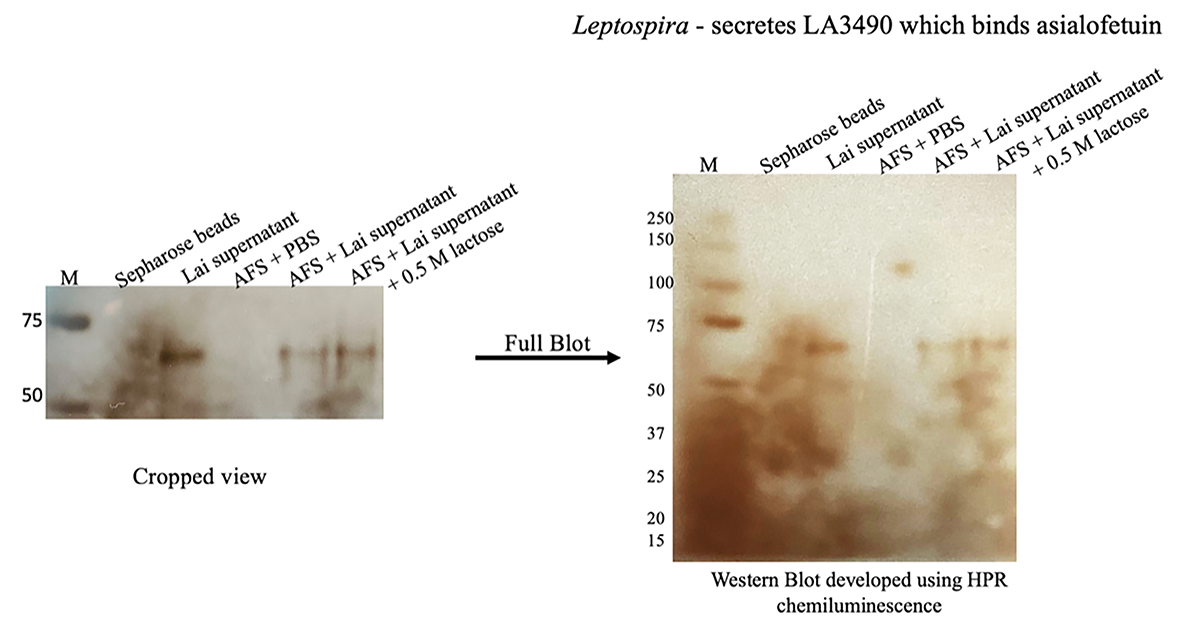

Supplement: Supplementary file 3 [file Image_3.TIF]
