# Supplementary material for: Pathogenic Leptospira Evolved a Unique Gene Family Comprised of Ricin B-Like Lectin Domain-Containing Cytotoxins
Source: Front Microbiol. 2022 Mar 29;13:859680. doi: 10.3389/fmicb.2022.859680 (PMC9002632; doi:10.3389/fmicb.2022.859680)
Supplement: Supplementary file 4 [file Presentation_1.pdf]

SUPPORTING INFORMATION APPENDIX

Pathogenic *Leptospira* Evolved a Unique Gene Family Comprised of Ricin B-like Lectin Domain-Containing Cytotoxins

This PDF file includes Legends for the following:

Supplementary Figures S1-S3

Supplementary Tables S1-S4

Supplementary Movies S1-S9

Supplementary Figures (S1, S2 and S3)

Supplementary Figure 1. Western immunoblot and Limulus Amebocyte Lysate assay confirming identity and purity of recombinant protein preparations. (A-B) Western blot of recombinant protein preparations confirming the presence of a single band of the expected size. *A*, t3490, *B*. rLA3490. Membranes were probed with anti-His_6 (lane 2) and polyclonal anti-LA3490 antibodies (lane 3). M - molecular weight marker. (**C**) Limulus Amebocyte Lysate assay *with E. coli* LPS as positive control indicating no appreciable endotoxin contamination. Data were visualized in GraphPad Prism v8.

Supplementary Figure 2. Caspase activation after rLA3490 treatment of HeLa cells. Super-resolution confocal fluorescence microscopy showed that adding the rLA3490-mCherry fusion protein to HeLa cells results in caspase-3 activation associated with internalization of the recombinant protein, as evidenced by cleavage of the caspase-3 recognition sequence/substrate (DEVD) producing green fluorescence. This co-localization also showed significant morphological changes in the nucleus, unlike the negative control t3490, or untreated HeLa cells **(A-B)**. Briefly, cell monolayer was treated with 45 nM recombinant fusion proteins for 4 h. Cells were washed and stained with PBS containing 10 μM NucView® 488 substrate and then mounted with ProLong™ Gold Antifade Mount plus DAPI. Images were captured using a 63x oil immersion objective using appropriate filters (blue, DAPI; green, caspase-3 active cells; and red, mCherry fusions). **(B)** Zoomed view indicating the co-localization of rLA3490 in nucleus and caspase-3 activation leading cell apoptosis, unlike t3490. **(C)** The impact of caspase-3 inhibitor and active caspase-3 fluorescence was read on spectrophotometer plate reader at 488/520 nm (excitation/emission). Prior treatment of HeLa cells with a caspase-3 inhibitor moderated the effect of rLA3490 on apoptotic cells**.** The various treatments

were evaluated via t-test in GraphPad Prism 8 and considered significant when $p < 0.05$, **ns** = non-significant.

Supplementary Figure 3. ***Leptospira interrogans*** **serovar Lai secretes VM proteins under** ***in vitro*** **high salt (120 mM NaCl) mimicking** ***in vivo*** **environmental conditions.** This figure depicts the original Western blot from which the cropped panel of Figure 3D was made.

Supplementary Tables (S1-S4)

Supplementary Tables S1-S3. **Pairwise distance matrices of full-length (Table S1), N-terminal carbohydrate binding region (i.e., positions 40 – 337 WRT Q8F0K3) containing tandem ricin B-like lectin domains (Table S2), and C-terminal segment (i.e., amino acid positions 375 – 639 WRT (Q8F0K3) encompassing the presumed C-terminal toxin domain (Table S2).** Pairwise distances of serovars Lai, Copenhageni and Canicola have been conditionally formatted, and all others de-emphasized for easier viewing. Cells colored in shades of green indicate low pair wise amino acid identities—darker shades = lowest; whereas those colored in shades of blue indicate higher pair wise amino acid identities—darker shades = highest. Orthologs have been indicated in boldface.

Supplementary Tables 4. Amino acid multiple sequence alignment (MSA) of all full-length PF07598 proteins found in serovars Lai, Copenhageni, Canicola, Hardjo and Pomona (only 12 Lai orthologs shown, 91 VM proteins in total), including schematic depiction of VM protein domain organization. Reference, Q8F0K3[rLA3490], appears first in the MSA, with every 10th amino acid residue from alanine at position nine onwards indicated. **(A)** The N-terminal segment containing tandem ricin B-like lectin domains, RBL1 and RBL2, including consensus logo and amino acid conservation. Gaps are mostly omitted, unless they occur in the reference, Q8F0K3, or required to indicate large gaps in the alignment, e.g., the complete deletion of the N-terminal segment of [Q8F8G6, rLA0591] (i.e., CBR deletion variants). Truncated, recombinant Q8F0K3 (i.e., t3490) contains a complete RBL1 domain, which has been indicated. Q8F0K3 amino acid residues matching HHpred-generated CARDS (27) toxin structure-consensus are

indicated using blue/white stars immediately above the consensus logo, with blue stars indicating residues that are 100% conserved in VM proteins and D2/D3 of CARDS toxin. **(B)** C-terminal segment presumed to mediate cytotoxicity, including a schematic depiction of a common *L. interrogans* CBR deletion VM protein variant. Statistically well-supported (i.e., $p < 10^{-3}$) Short Linear Motif (SLiM) mimics found in leptospiral VM proteins found in Q8F0K3 [rLA3490] (top sequence) and Q8F8D7 [rLA0620] (bottom sequence) are shown, with divergent residues in red. Predicted structural elements are also indicated, β-sheets (green) and α-helices (faded salmon). Amino acid residues predicted to form the hydrophobic face of amphipathic α-helices ($\alpha 1 - \alpha 7$) are indicated—enclosed in red ovals, with Q8F0K3 as guide. *Abbreviations*: DO*1-3*: Intrinsically disordered regions, one – three, numbered in order of appearance, N-terminal first. RBL1/2, segments identified as containing ricin B-like lectin domains, in order of appearance. NLS, nuclear localization signal. LIG_NRBOX1/2, dual LxxLL motifs conferring nuclear receptor binding. CTE, disordered C-terminal end, invariably terminated by CAAX motif.

Supplementary Movies (S1-S9)

Supplementary Movie S1. Time-lapse video showing morphological changes in rLA3490-treated HeLa cells (40 frames, 5s intervals). Cytopathic effect is apparent as early as 50 min post-exposure. Monolayers were treated with rLA3490 at a concentration of 45 nM. Images were captured at 40x objective via a Leica DMi8 inverted microscope. Scale bar 10 μm.

Supplementary Movie S2. Orthogonal projections of rLA3490-treated HeLa cells monolayers 30 min post-exposure showing binding/internalization of rLA3490-mCherry fusion protein at HeLa cell surface. Internalization of the rLA3490-mCherry fusion protein is evident with red fluorescence (top left, runtime 3 s; and bottom right, runtime 10 s). Plasma membrane and nuclei were stained with CellMask^{TM} Green Plasma Membrane Stain and ProLong™ Gold Antifade Mount with DAPI, respectively. Images were captured using a Leica SP8 Gated STED 3X super-resolution confocal microscope using a 100x oil immersion objective.

Supplementary Movie S3. Orthogonal projections of rLA3490-treated HeLa cells at 60 min post-exposure. Internalization of mCherry-rLA3490 fusion evident in all four images. Plasma membrane and nuclei were stained with CellMask^{TM} Green Plasma Membrane Stain and ProLong™ Gold Antifade Mount with DAPI, respectively. Images were captured using a Leica SP8 Gated STED 3X super-resolution confocal microscope using a 100x oil immersion objective.

Supplementary Movie S4. Orthogonal projections of t3490-treated HeLa cells at 30 min post-exposure. Top left and bottom right panels show binding of t3490-mCherry fusion protein (red) at HeLa cell surface 30 minutes post-exposure. Plasma membrane and nuclei stained with CellMask™ Green Plasma Membrane Stain and ProLong™ Gold Antifade Mount with DAPI, respectively. Images captured using a Leica SP8 Gated STED 3X super-resolution confocal microscope using a 100x oil immersion objective.

Supplementary Movie S5. Orthogonal projections of t3490-treated HeLa cells at 60 min post-exposure. Top left and bottom right panels show binding of mCherry-t3490 fusion at HeLa cell surface 60 minutes post-exposure. Plasma membrane and nuclei stained with CellMask™ Green Plasma Membrane Stain and ProLong™ Gold Antifade Mount with DAPI, respectively. Images captured using a Leica SP8 Gated STED 3X super-resolution confocal microscope using a 100x oil immersion objective.

Supplementary Movie S6. Z-stacks of rLA3490-treated HeLa cells at 30 min post-exposure. Animation of 35 cross-sectional images (total depth, 10.15 μm; separation, 298.5 nm) showing binding of mCherry-rLA3490 fusion to HeLa cell surface. Plasma membrane and nuclei were stained with CellMask™ Green Plasma Membrane Stain and ProLong™ Gold Antifade Mount with DAPI, respectively. Images were captured using a Leica SP8 Gated STED 3X super-resolution confocal microscope using a 100x oil immersion objective.

Supplementary Movie S7. Z-stacks of rLA3490-treated HeLa cells at 60 min post-exposure. Animation of 42 cross-sectional images (total depth, 12.24 μm; separation, 298.5 nm) showing cell membrane binding and internalization of mCherry-rLA3490

fusion. Plasma membrane and nuclei were stained with CellMask™ Green Plasma Membrane Stain and ProLong™ Gold Antifade Mount with DAPI, respectively. Images captured using a Leica SP8 Gated STED 3X super-resolution confocal microscope using a 100x oil immersion objective.

Supplementary Movie S8. Z-stacks of t3490-treated HeLa cells at 30 min post-exposure. Animation of 24 cross-sectional images (total depth, 6.87 μm; separation, 298.5 nm), with binding of mCherry-t3490 fusion evident at HeLa cell surface. Cells were washed twice with PBS. Plasma membrane and nuclei were stained with CellMask™ Green Plasma Membrane Stain and ProLong™ Gold Antifade Mount with DAPI, respectively. Images captured using a Leica SP8 Gated STED 3X super-resolution confocal microscope using a 100x oil immersion objective.

Supplementary Movie S9. Z-stacks of t3490-treated HeLa cells at 60 min post-exposure. Animation of 32 cross-sectional images (total depth, 9.25 μm; separation, 298.5 nm), with binding of mCherry-t3490 fusion still evident on HeLa cell surface (i.e. no internalization). Cells were washed twice with PBS. Plasma membrane and nuclei were stained with CellMask™ Green Plasma Membrane Stain and ProLong™ Gold Antifade Mount with DAPI, respectively. Images captured using a Leica SP8 Gated STED 3X super-resolution confocal microscope using a 100x oil immersion objective.
