# Supplementary material for: Pathogenic Leptospira Evolved a Unique Gene Family Comprised of Ricin B-Like Lectin Domain-Containing Cytotoxins
Source: Front Microbiol. 2022 Mar 29;13:859680. doi: 10.3389/fmicb.2022.859680 (PMC9002632; doi:10.3389/fmicb.2022.859680)
Supplement: Supplementary file 8 [file Table_4.pdf]

```
                                           10        20        30        40        50        60        70        80        90        100
EMG12588.1iGSA_sLT2186/1-601           ------------------------------------------------------mVIQKPTDPPKDKPIKVIVSGGGKFCYGPDFSGGESYIIIEQCWQMHVMNAR
AW3X1B_AW3.3968_AG10.iAW3/1-227        ----------------------------------------------------------------------------------------------------------
AP6X1B_AP6.3613_AG3.iAP6-/1-227        ----------------------------------------------------------------------------------------------------------
AP5X1B_AP5.3535_AG10.iAP5/1-227        ----------------------------------------------------------------------------------------------------------
WP.193824661.1iUNK/1-313               ---------------mgrw-ivlllillislgvgysygv-------------------------------------------------------------------
WP.163693602.1iUNK/1-313               ---------------mgrw-ivlllvllislgvgysygv-------------------------------------------------------------------
WP.061278819.1iUNK/1-313               ---------------mgrw-ivlllvllislgvgysygv-------------------------------------------------------------------
WP.061243963.1iUNK/1-313               ---------------mgrw-ivlllvllislgvgysygv-------------------------------------------------------------------
WP.061234208.1iUNK/1-313               ---------------mgrw-ivlllvllislgvgysygv-------------------------------------------------------------------
WP.010679749.1iUNK/1-313               ---------------mgrw-ivlllvllislgvgysygv-------------------------------------------------------------------
WP.002189487.1iUNK/1-313               ---------------mgrw-ivlllvllislgvgysygv-------------------------------------------------------------------
WP.002165058.1iUNK/1-313               ---------------mgrw-ivlllvllislgvgysygv-------------------------------------------------------------------
WP.000534003.1iUNK/1-313               ---------------mgrw-ivlllvllislgvgysygv-------------------------------------------------------------------
WP.000533986.1iUNK/1-313               ---------------mgrw-ivlllvllislgvgysygv-------------------------------------------------------------------
WP.000533985.1iUNK/1-313               ---------------mgrw-ivlllvllislgvgysygv-------------------------------------------------------------------
WP.000533980.1iUNK/1-313               ---------------mgrw-ivlllvllislgvgysygv-------------------------------------------------------------------
WP.000533956.1iUNK/1-313               ---------------mgrw-ivlllillislgvgysygv-------------------------------------------------------------------
WP.000533955.1iUNK/1-313               ---------------mgrw-ivlllillislgvgysygv-------------------------------------------------------------------
WP.000533954.1iUNK/1-313               ---------------mgrw-ivlllillislgvgysygv-------------------------------------------------------------------
WP.000533935.1iUNK/1-314               ---------------mgrw-iilllvllisfgvgysygv-------------------------------------------------------------------
UNK.iRCA_4042425/1-313                 ---------------mgrw-ivlllvllislgvgysygv-------------------------------------------------------------------
UNK.iR7_3573507/1-313                  ---------------mgrw-ivlllvllislgvgysygv-------------------------------------------------------------------
UNK.iR22_3583212/1-313                 ---------------mgrw-ivlllvllislgvgysygv-------------------------------------------------------------------
UNK.iR21_3584403/1-313                 ---------------mgrw-ivlllvllislgvgysygv-------------------------------------------------------------------
UNK.iR19_3583045/1-313                 ---------------mgrw-ivlllvllislgvgysygv-------------------------------------------------------------------
UNK.iR17_3583854/1-313                 ---------------mgrw-ivlllvllislgvgysygv-------------------------------------------------------------------
UNK.iR16_3574049/1-313                 ---------------mgrw-ivlllvllislgvgysygv-------------------------------------------------------------------
UNK.iR13_3574221/1-313                 ---------------mgrw-ivlllvllislgvgysygv-------------------------------------------------------------------
UNK.iR13L_3592842/1-313                ---------------mgrw-ivlllvllislgvgysygv-------------------------------------------------------------------
UNK.iR12_3583044/1-313                 ---------------msrw-ivlllvllislgvgysygv-------------------------------------------------------------------
UNK.iR11_3574690/1-313                 ---------------mgrw-ivlllvllislgvgysygv-------------------------------------------------------------------
QHH72245.1iUNK/1-313                   ---------------msrw-ivlllvllislgvgysygv-------------------------------------------------------------------
PN2Q12_PN2.1571_AG14.iPN2/1-313        ---------------mgrw-ivlllillislgvgysygv-------------------------------------------------------------------
PD1Q1D_PD1.4349_AG3.iPD1-/1-313        ---------------mgrw-ivlllvllislgvgysygv-------------------------------------------------------------------
MAE.iUPMMCNIIDLP_617623/1-314          ---------------mgrw-iilllvllisfgvgysygv-------------------------------------------------------------------
MAE.iUPMMCNIIDHP_617624/1-314          ---------------mgrw-iilllvllisfgvgysygv-------------------------------------------------------------------
KG2Q12_KG2.1465_AG3.uKG2-/1-313        ---------------mgrw-ivlllvllislgvgysygv-------------------------------------------------------------------
KG1Q12_KG1.14_AG14.uKG1-1/1-313        ---------------mgrw-ivlllillislgvgysygv-------------------------------------------------------------------
ICT.iS898_3627304/1-313                ---------------mgrw-ivlllvllislgvgysygv-------------------------------------------------------------------
ICT.iPiscina_3580676/1-313             ---------------mgrw-ivlllvllislgvgysygv-------------------------------------------------------------------
ICT.iLangkawi_3707575/1-313            ---------------mgrw-ivlllvllislgvgysygv-------------------------------------------------------------------
HJO.iNorma_3759514/1-313               ---------------mgrw-ivlllvllislgvgysygv-------------------------------------------------------------------
HJO.iL53_3705248/1-313                 ---------------mgrw-ivlllvllislgvgysygv-------------------------------------------------------------------
HJO.iHjtno_3686582/1-313               ---------------mgrw-ivlllvllislgvgysygv-------------------------------------------------------------------
EMN48154.1_PF07598_domain/1-313        ---------------mgrw-ivlllvllislgvgysygv-------------------------------------------------------------------
COP.iSK1_3627332/1-313                 ---------------mgrw-ivlllvllislgvgysygv-------------------------------------------------------------------
COP.iL1130_Q72N53.cl3624/1-313         ---------------mgrw-ivlllvllislgvgysygv-------------------------------------------------------------------
COP.iFDAARGOS203_1636889/1-313         ---------------mgrw-ivlllvllislgvgysygv-------------------------------------------------------------------
CLA.iS611_3608246/1-313                ---------------mgrw-ivlllillislgvgysygv-------------------------------------------------------------------
CLA.iS114_2632356/1-313                ---------------mgrw-ivlllillislgvgysygv-------------------------------------------------------------------
CLA.iRUFN_3138222/1-313                ---------------mgrw-ivlllillislgvgysygv-------------------------------------------------------------------
CLA.iLJ178_3613237/1-313               ---------------mgrw-ivlllillislgvgysygv-------------------------------------------------------------------
BVA.iPigK151_3742948/1-313             ---------------mgrw-ivlllvllislgvgysygv-------------------------------------------------------------------
AW3Q1B_AW3.4147_AG10.iAW3/1-313        ---------------mgrw-ivlllvllislgvgysygv-------------------------------------------------------------------
AP6Q1B_AP6.3788_AG3.iAP6-/1-313        ---------------mgrw-ivlllvllislgvgysygv-------------------------------------------------------------------
AP5Q1B_AP5.3710_AG10.iAP5/1-313        ---------------mgrw-ivlllvllislgvgysygv-------------------------------------------------------------------
WP.061241223.1iUNK/1-313               ---------------mgrw-ivlllvllislgvgysygv-------------------------------------------------------------------
WP.061234213.1iUNK/1-313               ---------------mgrw-ivlllvllislgvgysygv-------------------------------------------------------------------
WP.017857982.1iUNK/1-313               ---------------mgrw-ivlllvllislgvgysygv-------------------------------------------------------------------
WP.017854484.1iUNK/1-313               ---------------mgrw-ivlllvllislgvgysygv-------------------------------------------------------------------
WP.000533984.1iUNK/1-313               ---------------mgrw-ivlllvllislgvgysygv-------------------------------------------------------------------
WP.000533983.1iUNK/1-313               ---------------mgrw-ivlllvllislgvgysygv-------------------------------------------------------------------
WP.000533981.1iUNK/1-313               ---------------mgrw-ivlllvllislgvgysygv-------------------------------------------------------------------
WP.000533979.1iUNK/1-313               ---------------mgrw-ivlllvllislgvgysygv-------------------------------------------------------------------
WP.000533975.1iUNK/1-313               ---------------mgrw-ivlllvllislgvgysygv-------------------------------------------------------------------
WP.000533952.1iUNK/1-313               ---------------mgrw-ivlllillislgvgysygv-------------------------------------------------------------------
RT2Q12_RT2.598_AG3.iRT2-1/1-313        ---------------mgrw-ivlllvllislgvgysygv-------------------------------------------------------------------
PN3Q12_SLPN3.697_AG3.iPN3/1-313        ---------------mgrw-ivlllvllislgvgysygv-------------------------------------------------------------------
LHI.iS56609_3691353/1-313              ---------------mgrw-ivlllvllislgvgysygv-------------------------------------------------------------------
KW2Q12_KW2.608_AG3.iKW2-1/1-313        ---------------mgrw-ivlllvllislgvgysygv-------------------------------------------------------------------
KW2Q12_KW2.596_AG3.iKW2-1/1-313        ---------------mgrw-ivlllvllislgvgysygv-------------------------------------------------------------------
KW1Q1C_KW1.3531_AG3.iKW1-/1-313        ---------------mgrw-ivlllvllislgvgysygv-------------------------------------------------------------------
CLA.iS782_3902791/1-313                ---------------mgrw-ivlllvllislgvgysygv-------------------------------------------------------------------
BAE.iS1548_3700587/1-313               ---------------mgrw-ivlllvllislgvgysygv-------------------------------------------------------------------
BAE.iS1489_3766124/1-313               ---------------mgrw-ivlllvllislgvgysygv-------------------------------------------------------------------
AW1Q12_AW1.408_AG3.iAW1-1/1-313        ---------------mgrw-ivlllvllislgvgysygv-------------------------------------------------------------------
AP7Q12_AP7.594_AG3.iAP7-1/1-313        ---------------mgrw-ivlllvllislgvgysygv-------------------------------------------------------------------
AP3Q12_AP3.697_AG10.iAP3-/1-313        ---------------mgrw-ivlllvllislgvgysygv-------------------------------------------------------------------
AP1Q12_AP1.1119_AG3.iAP1-/1-313        ---------------mgrw-ivlllvllislgvgysygv-------------------------------------------------------------------
AG3Q12_U1.697_AG3.iO73718/1-313        ---------------mgrw-ivlllvllislgvgysygv-------------------------------------------------------------------
WP.192486091.1iUNK/1-313               ---------------mgrw-ivlllvllislgvgysygv-------------------------------------------------------------------
WP.002120557.1iUNK/1-313               ---------------mgrw-ivlllvllislgvgysygv-------------------------------------------------------------------
WP.000533982.1iUNK/1-313               ---------------mgrw-ivlllvllislgvgysygv-------------------------------------------------------------------
LAI.iS56601_599890/1-313               ---------------mgrw-ivlllvllislgvgysygv-------------------------------------------------------------------
LAI.iIPAV_599534/1-313                 ---------------mgrw-ivlllvllislgvgysygv-------------------------------------------------------------------
AW1X1C_AW1.3438_AG3.iAW1-/1-226        ----------------------------------------------------------------------------------------------------------
PD1Q12_PD1.1337_AG3.iPD1-/1-303        ----------------------------------------------------------------------------------------------------------
KG2Q1C_KG2.5021_AG3.uKG2-/1-303        ----------------------------------------------------------------------------------------------------------
KG2Q19_KG2.3779_AG3.uKG2-/1-324        ----------------------------------------------------------------------------------------------------------
AW1X1A_AW1.3436_AG3.iAW1-/1-334        ---------------MYNWKKILIVVLLASIMVYLEYEMDHTLVHAASSSKTTNSIVQKPTDPPKDKPIKVNVSGGGTFCYGPDFSGGESYIIIEQCWQMHVMNAR
PD1Q13_PD1.1338_AG3.iPD1-/1-321        ---------------MYKW-KVLTAFFLISIGSGFEYGVNHTHIHA--LSKIEYSVIQKPTDPPKDKPIKVIVSDGGKFCYGPNFSGGESYIIIEQCWQMHVMNAR
KG2Q1D_KG2.5022_AG3.uKG2-/1-321        ---------------MYKW-KVLTAFFLISIGSGFEYGVNHTHIHA--LSKIEYSVIQKPTDPPKDKPIKVIVSDGGKFCYGPNFSGGESYIIIEQCWQMHVMNAR
UNK.yVS2413_411205/1-638               ---------------MGRW-IVLTVVLLVSIGVSFTYGGNS----I-SSNGISGSIIQKPTDKPNDKSIKIVAHNGRNYCYSPMFSHNESYIQIQQCGDR-TPKAR
UNK.yS200901116_756730/1-638           ---------------MGRW-IVLTVVLLVSIGVSFTYGGNS----I-SSNGISGSIIQKPTDKPNDKSIKIVAHNGRNYCYSPMFSHNESYIQIEQCGDR-TPKAR
UNK.yMDI272_108563/1-638               ---------------MGRW-IVLTVVLLVSIGVSFTYGGNS----I-SSNGISGSIIQKPTDKPNDKSIKIVAHNGRNYCYSPMFSHNESYIQIEQCGDR-TPKAR
UNK.yMDI222_446693/1-638               ---------------MGRW-IVLTVVLLVSIGVSFTYGGNS----I-SSNGISGSIIQKPTDKPNDKSIKIVAHNGRNYCYSPMFSHNESYIQIEQCGDR-TPKAR
UNK.wCUD13_3157649/1-638               ---------------MGRW-IVLTVVLLVSIGVSFTYGGNS----I-SSSGIPGSIVQKPTDKPNDKPIKIIAHNGKNYCYSPMFGSNESYIQLQGCGDK-TPKAR
PD2X13_PD2.4149_GTG6.wPD2/1-613        ---------------MGRW-IVLTVVLLVSIGVSFTYGGNS----I-SSSGIPGSIVQKPTDKPNDKPIKIIAHNGKNYCYSPMFGSNESYIQLQGCGDK-TPKAR
RT1X14_RT1.4105_GCG2.wRT1/1-614        ---------------MGRW-IVLTVVLLVSIGVSFTYGGNS----I-SSSGIPGSIVQKPTDKPNDKPIKIIAHNGKNYCYSPMFGSNESYIQLQGCGDK-TPKAR
UNK.wCUDO6_1427232/1-638               ---------------MGRW-IVLTVVLLVSIGVSFTYGGNS----I-SSSGIPGSIVQKPTDKPNDKPIKIIAHNGKNYCYSPMFTIDGSFIQLQGCGDK-TPKAR
UNK.iR14-L_1959517/1-638               ---------------MGRW-IVLTVILLISIGVSFTYGGSS----I-SSSGIPGSVAQKPTDKPNDQPIKIVAHNGKNYCYSPMFGRNESYIRLEQCGDK-TLKAR
UNK.bR6_1833791/1-638                  ---------------MGRW-IVLTVILLISIGVSFTYGGSS----I-SSSGIPGSVAQKPTDKPNDQPIKIVAHNGKNYCYSPMFGRNESYIRLEQCGDK-TLKAR
UNK.bR6L_1899418/1-638                 ---------------MGRW-IVLTVILLISIGVSFTYGGSS----I-SSSGIPGSVAQKPTDKPNDQPIKIVAHNGKNYCYSPMFGRNESYIRLEQCGDK-TLKAR
UNK.bR29_1897969/1-638                 ---------------MGRW-IVLTVILLISIGVSFTYGGSS----I-SSSGIPGSVAQKPTDKPNDQPIKIVAHNGKNYCYSPMFGRNESYIRLEQCGDK-TLKAR
```

UNK.bR28_1898050/1-638	---------------MGRW-IVLTVILLISIGVSFTYGGSS----I-SSSGIPGSVAQKPTDKPNDQPIKIVAHNGKNYCYSPMFGRNESYIRLEQCGDK-TLKAR
UNK.bR23_1899648/1-638	---------------MGRW-IVLTVILLISIGVSFTYGGSS----I-SSSGIPGSVAQKPTDKPNDQPIKIVAHNGKNYCYSPMFGRNESYIRLEQCGDK-TLKAR
UNK.bR14_2010228/1-638	---------------MGRW-IVLTVILLISIGVSFTYGGSS----I-SSSGIPGSVAQKPTDKPNDQPIKIVAHNGKNYCYSPMFGRNESYIRLEQCGDK-TLKAR
UNK.bMo4_1958287/1-638	---------------MGRW-IVLTVILLISIGVSFTYGGSS----I-SSSGIPGSVAQKPTDKPNDQPIKIVAHNGKNYCYSPMFGRNESYIRLEQCGDK-TLKAR
PN4Z11_PN4.784_GTG3.bPN4-/1-638	---------------MGRW-IVLTVILLISIGVSFTYGGSS----I-SSSGIPGSVAQKPTDKPNDQPIKIVAHNGKNYCYSPMFGRNESYIRLEQCGDK-TLKAR
PN3Z11_SLPN3.794_GTG3.bPN/1-638	---------------MGRW-IVLTVILLISIGVSFTYGGSS----I-SSSGIPGSVAQKPTDKPNDQPIKIVAHNGKNYCYSPMFGRNESYIRLEQCGDK-TLKAR
PN1Z11_PN1.789_GTG3.bPN1-/1-638	---------------MGRW-IVLTVILLISIGVSFTYGGSS----I-SSSGIPGSVAQKPTDKPNDQPIKIVAHNGKNYCYSPMFGRNESYIRLEQCGDK-TLKAR
HJO.bS4E_1930400/1-638	---------------MGRW-IVLTVILLISIGVSFTYGGSS----I-SSSGIPGSVAQKPTDKPNDQPIKIVAHNGKNYCYSPMFGRNESYIRLEQCGDK-TLKAR
CCA.bPiyasena_725629/1-638	---------------MGRW-IVLTVILLISIGVSFTYGGSS----I-SSSGIPGSVAQKPTDKPNDQPIKIVAHNGKNYCYSPMFGRNESYIRLEQCGDK-TLKAR
BUM.iS56604_1930400/1-638	---------------MGRW-IVLTVILLISIGVSFTYGGSS----I-SSSGIPGSVAQKPTDKPNDQPIKIVAHNGKNYCYSPMFGRNESYIRLEQCGDK-TLKAR
AP9Z11_AP9.2731_GTG3.bAP9/1-638	---------------MGRW-IVLTVILLISIGVSFTYGGSS----I-SSSGIPGSVAQKPTDKPNDQPIKIVAHNGKNYCYSPMFGRNESYIRLEQCGDK-TLKAR
AP8Z11_AP8.788_GTG3.bAP8-/1-638	---------------MGRW-IVLTVILLISIGVSFTYGGSS----I-SSSGIPGSVAQKPTDKPNDQPIKIVAHNGKNYCYSPMFGRNESYIRLEQCGDK-TLKAR
AP5Z11_AP5.794_GTG3.bAP5-/1-638	---------------MGRW-IVLTVILLISIGVSFTYGGSS----I-SSSGIPGSVAQKPTDKPNDQPIKIVAHNGKNYCYSPMFGRNESYIRLEQCGDK-TLKAR
AP4Z11_AP4.788_GTG3.bAP4-/1-638	---------------MGRW-IVLTVILLISIGVSFTYGGSS----I-SSSGIPGSVAQKPTDKPNDQPIKIVAHNGKNYCYSPMFGRNESYIRLEQCGDK-TLKAR
AP2Z11_AP2.786_GTG3.bAP2-/1-638	---------------MGRW-IVLTVILLISIGVSFTYGGSS----I-SSSGIPGSVAQKPTDKPNDQPIKIVAHNGKNYCYSPMFGRNESYIRLEQCGDK-TLKAR
HJO.bS203_2936772/1-638	---------------MGRW-IVLTVILLISIGVSFTYGGSS----I-SSSGIPGSVVQKPTDKPNDKPIKIVAHNGKNYCYSPMFGRNESYIQLEQCGDK-TLKAR
HJO.bNVSLs13433_2969566/1-638	---------------MGRW-IVLTVILLISIGVSFTYGGSS----I-SSSGIPGSVVQKPTDKPNDKPIKIVAHNGKNYCYSPMFGRNESYIQLEQCGDK-TLKAR
HJO.bL550_2966011/1-638	---------------MGRW-IVLTVILLISIGVSFTYGGSS----I-SSSGIPGSVVQKPTDKPNDKPIKIVAHNGKNYCYSPMFGRNESYIQLEQCGDK-TLKAR
HJO.bL49_2981788/1-638	---------------MGRW-IVLTVILLISIGVSFTYGGSS----I-SSSGIPGSVVQKPTDKPNDKPIKIVAHNGKNYCYSPMFGRNESYIQLEQCGDK-TLKAR
HJO.bBK3006005/1-638	---------------MGRW-IVLTVILLISIGVSFTYGGSS----I-SSSGIPGSVVQKPTDKPNDKPIKIVAHNGKNYCYSPMFGRNESYIQLEQCGDK-TLKAR
HJO.bBK2988145/1-638	---------------MGRW-IVLTVILLISIGVSFTYGGSS----I-SSSGIPGSVVQKPTDKPNDKPIKIVAHNGKNYCYSPMFGRNESYIQLEQCGDK-TLKAR
HJO.bBK2985692/1-638	---------------MGRW-IVLTVILLISIGVSFTYGGSS----I-SSSGIPGSVVQKPTDKPNDKPIKIVAHNGKNYCYSPMFGRNESYIQLEQCGDK-TLKAR
HJO.bNVSLs818_703087/1-638	---------------MGRW-IVLTVILLISIGVSFTYGGSS----I-SSSGIPGSVVQKPTDKPNDKPIKIVAHNGKNYCYSPMFGRNESYIQLEQCGDK-TLKAR
HJO.bJB197_696223/1-638	---------------MGRW-IVLTVILLISIGVSFTYGGSS----I-SSSGIPGSVVQKPTDKPNDKPIKIVAHNGKNYCYSPMFGRNESYIQLEQCGDK-TLKAR
WP.081100479.1nUNK/1-634	---------------marC-----IVLLVLIGISFTYGWSS----I-SSSILPGSIVQRPTDKPKDKPIKIVVHDGGKYCYAPVFIRGESYIQIGGCGEQEVNNAR
WP.020980218.1nUNK/1-634	---------------marC-----IVLLVLIGISFTYGWSS----I-SSSILPGSIVQRPTDKPKDKPIKIVVHDGGKYCYAPVFIRGESYIQIGGCGEQEVNNAR
WP.002179060.1nUNK/1-634	---------------marC-----IVLLVLIGISFTYGWSS----I-SSSILPGSIVQRPTDKPKDKPIKIVVHDGGKYCYAPVFIRGESYIQIGGCGEQEVNNAR
WP.004440471.1nUNK/1-630	---------------marC-----IVLLVLIGISFTYGWNS----I-SSSALPGSIVQRPTDKPKDKPIKILVHDGGKYCYTPVFIRGESYIQIGRCGEQEVNNAR
WP.061251457.1nUNK/1-630	---------------marC-----IVLLVLIGISFTYGWNS----I-SSSALPGSIVQRPTDKPKDKPIKILVHDGGKYCYTPVFIRGESYIQIGRCGEQEVNNAR
WP.004426631.1nUNK/1-630	---------------marC-----IVLLVLIGISFTYGWNS----I-SSSALPGSIVQRPTDKPKDKPIKILVHDGGKYCYTPVFIRGESYIQIGRCGEQEVNNAR
WP.061245665.1nUNK/1-629	---------------marC-----IVLLVLIGISFTYGWNS----I-SSSALPGSIVQRPTDKPKDKPIKIVVHDGGKYCYTPVFIRGESYIQIGRCGEQEVNNAR
WP.004450195.1nUNK/1-629	---------------marC-----IVLLVLIGISFTYGWNS----I-SSSALPGSIVQRPTDKPKDKPIKIVVHDGGKYCYTPVFIRGESYIQIGRCGEQEVNNAR
WP.002154169.1nUNK/1-629	---------------marC-----IVLLVLIGISFTYGWNS----I-SSSALPGSIVQRPTDKPKDKPIKIVVHDGGKYCYTPVFIRGESYIQIGRCGEQEVNNAR
WP.061526997.1kUNK/1-632	---------------MGRW-IVLLLVLLASIGVGYSYGVNPESVPI-PSNKIPGSIIQRPTDKPKDKPIKIVTHDGGTFCYAPVFIGGKSYIQIEQCWEQEVSNAR
WP.082293468.1kUNK/1-627	---------------MNRW-IVLLLVLLASIGVGYSYGVNPESVPI-PSNKIPGSIIQRPTDKPKDKPIKIVIHDGGTFCYAPVFSGGKSYIQIEQCGEQEVSNAR
WP.082293269.1kUNK/1-632	---------------MGRW-IVLLLVLLASIGVGYSYGVNPESVPI-PSNKIPGSIIQRPTDKPKDKPIKIVTHDGGTFCYAPVFSGGKSYIQIEQCGEQEVSNAR
PN5K1B_PN5.3372_AG15.kPN5/1-407	---------------MGRW-IVLLLVLLVSIGVGYSYGVNPESVPI-LSNKIPGSIIQIPTDKPKDKPIKIVTHDGGTFCYAPVFSGGKSYIQIEQRWEQEVSNAR
WP.082286384.1kUNK/1-630	---------------MNRW-IVLLLVLLVSIGVGYSYGVNPESVPI-PSNKIPGSIIQRPTDKPKDKPIKIVTHDGGSFCYAPVFIGGKSYIQIEQCWEQEVSNAR
WP.016560633.1kUNK/1-630	---------------MGRW-IVLLLVLLASIGVGYSYGVNPESVPI-PSNKIPGSIIQRPTDKPKDKPIKIVIHDGGSFCYAPVFIGGKSYIQIEQCWEQEVSNAR
WP.053388525.1kUNK/1-630	---------------MNRW-IVLLLVLLVSIGVGYSYGVNPESVPI-PSNKIPGSIIQRPTDKPKDKPIKIVIHDGGSFCYAPVFIGGKSYIQIEQCWEQEVSNAR
WP.057162247.1kUNK/1-630	---------------MNRW-IVLLLVLLASIGVGYSYGVNPESVPI-PSNKIPGSIIQRPTDKPKDKPIKIVIHDGGTFCYAPVFIGGKSYIQIEQCWEQEVSNAR
WP.057162290.1kUNK/1-632	---------------MNRW-IVLLLVLLASIGVGYSYGVNPESVPI-PSNKIPGSIIQRPTDKPKDKPIKIVTHDGGSFCYAPVFIGGKSYIQIEQCWEQEVSNAR
WP.016560669.1kUNK/1-632	---------------MNRW-IVLLLVLLASIGVGYSYGVNPESVPI-PSNKIPGSIIQRPTDKPKDKPIKIVIHDGGSFCYAPVFIGGKSYIQIEQCWEQEVSNAR
WP.004777426.1kUNK/1-632	---------------MNRW-IVLLLVLLVSIGVGYSYGVNPESVPI-PSNKIPGSIIQRPTDKPKDKPIKIVTHDGGSFCYAPVFIGGKSYIQIEQCWEQEVSNAR
WP.004770809.1kUNK/1-632	---------------MNRW-IVLLLVLLVSIGVGYSYGVNPESVPI-PSNKIPGSIIQRPTDKPKDKPIKIVIHDGGAFCYAPVFIGGKSYIQIEQCWEQEVSNAR
EMO75296.1kUNK_s200801925/1-632	---------------MNRW-IVLLLVLLVSIGVGYSYGVNPESVPI-PSNKIPGSIIQRPTDKPKDKPIKIVTHDGGSFCYAPVFIGGKSYIQIEQCWEQEVSNAR
WP.004768128.1kUNK/1-632	---------------MNRW-IVLLLVLLVSIGVGYSYGVNPESVPI-PSNKIPGSIIQRPTDKPKDKPIKIVTHDGGSFCYAPVFIGGKSYIQIKQCWEQEVSNAR
WP.004758578.1kUNK/1-632	---------------MNRW-IVLLLVLLASIGVGYSYGVNPESVPI-PSNKIPGSIIQRPTDKPKDKPIKIVTHDGGSFCYAPVFIGGKSYIQIEQCWEQEVSNAR
WP.082286398.1kUNK/1-632	---------------MNRW-IVLLLVLLASIGVGYSYGVNPESVPI-PSNKIPGSIIQRPTDKPKDKPIKIVTHDGGSFCYAPVFIGGKSYIQIEQCWEQEVSNAR
WP.016750515.1kUNK/1-630	---------------MGRW-IILLLVLLVSIGVGYSYGVNPESVPI-LSNKIPGSIIQRPTDKPKDKPIKIVIHDGGTFCYAPVFIGGKSYIQIEQCWEQEVSNAR
WP.016560547.1kUNK/1-630	---------------MGRW-IVLLLVLLVSIGVGYSYGVNPEIVPI-PSNKIPGSTIQRPTDKPKDKPIKIVIHDGGSFCYAPVFIGGKSYIQIEQCWEQEVSNAR
WP.057162246.1kUNK/1-630	---------------MNRW-IVLLLVLLVSIGVGYSYGVNPESVPI-PSNKIPGSIIQRPTDKPKDKPIKIVIHDGGSFCYAPVFIGGKSYIQIEQCWEQEVSNAR
WP.082293154.1kUNK/1-631	---------------MGRW-IVLLLVLLASIGVGYSYGVNPESVPI-LSNKIPGSIIQRPTDKPKDKPIKIVIHDGGTFCYAPVFSGGKSYIQIEQCWEQEVSNAR
WP.004781573.1kUNK/1-631	---------------MGRW-IVLLLVLLVSIGVGYSYGVNPESVPI-LSNKIPGSIIQRPTDKPKDKPIKIVIHDGGTFCYAPVFSGGKSYIQIEQCWEQEVSNAR
EMO77328.1kUNK_s200801925/1-631	---------------MGRW-IVLLLVLLVSIGVGYSYGVNPESVPI-LSNKIPGSIIQRPTDKPKDKPIKIVIHDGGAFCYAPVFSGGKSYIQIEQCWEQEVSNAR
WP.004767287.1kUNK/1-631	---------------MGRW-IILLLVLLVSIGVGYSYGVNPESVPI-LSNKIPGSIIQRPTDKPKNKPIKIVIHDGGTFCYAPVFIGGKSYIQIEQCWEQEVSNAR
WP.057162236.1kUNK/1-631	---------------MGRW-IILLLVLLVSIGVGYSYGVNPESVPI-LSNKIPGSIIQRPTDKPKDKPIKIVIHDGGTFCYAPVFIGGKSYIQIEQCWEQEVSNAR
WP.025180626.1kUNK/1-631	---------------MNRW-IVLLLVLLASIGVGYSYGVNPESVPI-PSNKIPGSIIQRPTDKPKDKPIKIVIHDGGSFCYAPVFIGGKSYIQIEQCWEQEVSNAR
WP.017858927.1kUNK/1-631	---------------MNRW-IVLLLVLLASIGVGYSYGVNPESVPI-PSNKIPGSIIQRPTDKPKDKPIKIVIHDGGTFCYAPVFIGGKSYIQIEQCWEQEVSNAR
WP.020764625.1kUNK/1-631	---------------MNRW-IVLLLVLLVSIGVGYSYGVNPESVPI-PSNKIPGSIIQRPTDKPKDKPIKIVTHDGGSFCYAPVFIGGKSYIQIEQCWEQEVSNAR
WP.016560855.1kUNK/1-631	---------------MGRW-IILLLVLLVSIGVGYSYGVNPESVPI-LSNKIPGSIIQRPTDKPKDKPIKIVIHDGGTFCYAPVFIGGKSYIQIEQCWEQEVSNAR
WP.004759267.1kUNK/1-631	---------------MGRW-IVLLLVLLVSIGVGYSYGVNPESVPI-LSNKIPGSIIQRPTDKPKDKPIKIVIHDGGTFCYAPVFIGGKSYIQIEQCWEQEVSNAR
WP.004776922.1kUNK/1-631	---------------MGRW-IVLLLVLLVSIGVGYSYGVNPESVPI-LSNKIPGSIIQRPTDKPKDKPIKIVIHDGGTFCYAPVFIGGKSYIQIEQCWEQEVSNAR
WP.004768686.1kUNK/1-631	---------------MGRW-IVLLLVLLVSIGVGYSYGVNPESVPI-LSNKIPGSIIQRPTDKPKDKPIKIVIHDGGTFCYAPVFIGGKSYIQIEQCWEQEVSNAR
EMO74331.1kUNK_s200801925/1-632	---------------MGRW-IVLLLVLLVSIGVGYSYGVNPESVPI-LSNKIPGSIIQRPTDKPKDKPIKIVIHDGGTFCYAPVFIGGKSYIQIEQCWEQEVSNAI
WP.004771699.1kUNK/1-632	---------------MGRW-IVLLLVLLVSIGVGYSYGVNPESVPI-LSNKIPGSIIQRPTDKPKDKPIKIVIHDGGAFCYAPVFIGGKSYIQIEQCWEQEVSNAR
WP.004750469.1kUNK/1-631	---------------MGRW-IILLLVLLVSIGVGYSYGVNPESVPI-LSNKIPGSIIQRPTDKPKDKPIKIVIHDGGTFCYAPVFIGGKSYIQIEQCWEQEVSNAR
WP.061220051.1sUNK/1-632	---------------MSRR-IVLIVVLLVSVGVDFSYGVNPALIDI-ISNAVPPSIIQRPTDKPKDKPIKVVVS-GGTYCYAPVFSGGESYIHIRQCWEQDVANAR
WP.016757225.1sUNK/1-632	---------------MSRR-IVLIVVLLVSVGVDFSYGVNPALIDI-ISNAVPPSIIQRPTDKPKDKPIKVVVS-GGTYCYAPVFSGGESYIHIRQCWEQDVANAR
WP.004461679.1sUNK/1-632	---------------MSRR-IVLIVILLVSVGVDFSYGVNPALIDI-ISNAVPPSIIQRPTDKPKDKPIKVVVS-GGTYCYAPVFSGGESYIHIRQCWEQDVANAR
SNI.sLT821_2635648/1-632	---------------MSRR-IVLIVILLVSVGVDFSYGVNPALIDI-ISNAVPPSIIQRPTDKPKDKPIKVVVS-GGTYCYAPVFSGGESYIHIRQCWEQDVANAR
WP.004465983.1sUNK/1-632	---------------MSRR-IVLIVVLLVSVGVDFSYGVNPALIDI-ISNAVPPSIIQRPTDKPKDKPIKVVVS-GGTYCYAPVFSGGESYIHIRQCWEQDVANAR
WP.004481144.1sUNK/1-632	---------------MSRR-IVLIVILLVSVGVDFSYGVNPALIDI-ISNAVPPSIIQRPTDKPKDEPIKVVVS-GGTYCYAPVFSGGESYINIRQCWEQDVANAR
WP.017863276.1sUNK/1-632	---------------MSRR-IVLIVILLVSVGVDFSYGVNPALIDI-ISNAVPPSIIQRPTDKPKDEPIKVVVS-GGTYCYAPVFSGGESYINIRQCWEQDVANAR
WP.046943672.1sUNK/1-632	---------------MSRL-IVLIVVLLVSVGVDFSYGVNPALIDI-ISNAVPPSIIQRPTDKPKDEPIKVVVS-GGTYCYAPVFSGGESYINIRQCWEQEVANAR
WP.032916236.1sUNK/1-632	---------------MSRR-IVLIVVLLVSVGVDFSYGVNPALIDI-ISNAVPPSIIQRPTDKPKDEPIKVVVS-GGTYCYAPVFSGGESYINIRQCWEQEVANAR
WP.004492935.1sUNK/1-632	---------------MSRR-IVLIVVLLVSVGVDFSYGVNPALIDI-ISNAVPPSIIQRPTDKPKDEPIKVVVS-GGTYCYAPVFSGGESYINIRQCWEQEVANAR
EMO71697.1sUNK_s200403458/1-647	mylpyltfQKKRGKKMSRR-IVLIVVLLVSVGVDFSYGVNPALIDI-ISNAVPPSIIQRPTDKPKDEPIKVVVS-GGTYCYAPVFSGGESYINIRQCWEQEVANAR
WP.004478293.1sUNK/1-632	---------------MSRR-IVLIVILLVSVGVDFSYGVNPALIDI-ISNAVPPSIIQRPTDKPKDEPIKVVVS-GGTYCYAPVFSGGESYINIRQCWEQDVANAR
WP.004475697.1sUNK/1-632	---------------MSRR-IVLIVILLVSVGVDFSYGVNPALIDI-ISNAVPPSIIQRPTDKPKDEPIKVVVS-GGTYCYAPVFSGGESYINIRQCWEQDVANAR
WP.004468786.1sUNK/1-632	---------------MSRR-IVLIVILLVSVGVDFSYGVNPALIDI-ISNAVPPSIIQRPTDKPKDEPIKVVVS-GGTYCYAPVFSGGESYINIRQCWEQDVANAR
WP.016755627.1sUNK/1-632	---------------MSRR-IVLIVILLVSVGVDFSYGVNPALIDI-ISNAVPPSIIQRPTDKPKDEPIKVVVS-GGTYCYAPVFSGGESYIHIRQCWEQDVANAR
WP.046691934.1sUNK/1-632	---------------MSRL-IVLIVVLLVSVGVDFSYGVNPALIDI-ISNAVPPSIIQRPTDKPKDEPIKVVVS-GGTYCYAPVFSGGESYIHIRQCWEQDVANAR
WP.046951602.1sUNK/1-632	---------------MSRL-IVLIVVLLVSVGVDFSYGVNPALIDI-ISNAVPPSIIQRPTDKPKDEPIKVVVS-GGTYCYAPVFSGGESYINIRQCWEQEVANAR
WP.075979500.1sUNK/1-632	---------------MSRL-IVLIVILLVSVGVDFSYGVNPALIDI-ISNAVPPSIIQRPTDKPKDEPIKVVVS-GGTYCYAPVFSGGESYINIRQCWEQEVANAR
WP.008396482.1mUNK/1-632	---------------MSRL-IVLIVVLLVSVGVDFSYGVNPALIDI-ISNAVPPSIIQRPTDKPKDEPIKVVVS-GGTYCYAPVFSGGESYINIRQCWEQEVANAR
WP.134788057.1sUNK/1-635	-------------msrlivlIVLIVVLLVSVGVDFSYGVNPALIDI-ISNAVPPSIIQRPTDKPKDEPIKVVVS-GGTYCYAPVFSGGESYINIRQCWEQDVANAR
WP.061525945.1sUNK/1-632	---------------MSRR-IVLIVILLVSVGVDFSYGVNPALIDI-ISNAVPPSIIQRPTDKPKDKPIKVVVS-GGTYCYAPVFSGGESYIHIRQCWEQDVANAR
WP.004482757.1sUNK/1-632	---------------MSRR-IVLIVILLVSVGVDFSYGVNPALIDI-ISNAVPPSIIQRPTDKPKDEPIKVVVS-GGTYCYAPVFSGGESYINIRQCWEQDVANAR
WP.004491224.1sUNK/1-632	---------------MSRR-IVLIVVLLVSVGVDFSYGVNPALIDI-ISNAVPPSIIQRPTDKPKDEPIKVVVS-GGTYCYAPVFSGGESYIHIRQCWEQDVANAR
WP.004472422.1sUNK/1-632	---------------MSRR-IVLIVILLVSVGVDFSYGVNPALIDI-ISNAVPPSIIQRPTDKPKDEPIKVVVS-GGTYCYAPVFSGGESYIHIRQCWEQDVANAR
WP.075918231.1sUNK/1-632	---------------MSRR-IVLIVILLVSVGVDFSYGMNPALIDI-ISNAIPPSIIQRPTDKPKDEPIKVVVS-GGTYCYAPVFSGGESYINIRQCWEQGVANAR
WP.193811390.1bUNK/1-629	---------------MIRL-IVLIVVLLVSIGVDFSYGVNSALIDI-VSRSIPGSIIQKPTDTPQDRAIKIVIHDGGKFCYAPVFSGGESYIRILQCWEQGVSNAR
WP.016757603.1bUNK/1-629	---------------MIRL-IVLIVVLLVSIGVDFSYGVNSALIDI-VSRSIPGSIIQKPTDTPQDRAIKIVIHDGGKFCYAPVFSGGESYIRILQCWEQGVSNAR
WP.193824231.1bUNK/1-632	---------------MIRL-IVLIVVLLVSIGVDFSYGVNSALIDI-VSRSIPGPIIQKPTDTPQDRAIKIVIHDGGKFCYAPVFSGGESYIRILQCWEQGVSNAR
WP.193824181.1bUNK/1-632	---------------MIRL-IVLIVVLLVSIGVDFSYGVNSALIDI-VSRSIPGPIIQKPTDTPQDRAIKIVIHDGGKFCYAPVFSGGESYIRILQCWEQGVSNAR
GIM18248.1bUNK_pKHM09.069/1-632	---------------MIRL-IVLIVVLLVSIGVNFSYGVNSALIDI-VSRSIPGSIIQKPTDTPQDRAIKIVLHDGGKFCYAPVFSGGESYIRILQCWEQGVSNAR
WP.002734644.1bUNK/1-629	---------------MIRL-IVLIVVLLVSIGVNFSYGVNSALIDI-VSRSIPGSIIQKPTDTPQDRAIKIVIHDGGKFCYAPVFSGGESYIRILQCWEQGVSNAR
WP.118883531.1bUNK/1-632	---------------MIRL-IVLIVVLLVSIGVNFSYGVNSALIDI-VSRSIPGSIIQKPTDTPQDRAIKIVIHDGGKFCYAPVFSGGESYIRILQCWEQGVSNAR
GIM18247.1bUNK_pKHM09.069/1-629	---------------MIRL-IVLIVVLLVSIGVNFSYGVNSALIDI-VSRSIPGSIIQKPTDTPQDRAIKIVIHDGGKFCYAPVFSGGESYIRILQCWEQGVSNAR
ABJ80524.1bHJO_sL550/1-629	-----------------mIRLIIVLLVSIGVDFSYGVNSALIDI-VSRSIPGSIIQKPTDTPQDRAIKIVIHDRGKFCYAPVFSGGESYIRILQCWEQDVSNAR
ABJ77580.1bHJO_sJB107/1-629	-----------------mIRLIIVLLVSIGVDFSYGVNSALIDI-VSRSIPGSIIQKPTDTPQDRAIKIVIHDRGKFCYAPVFSGGESYIRILQCWEQDVSDAR
WP.002757815.1bUNK/1-629	---------------MIRL-IVLIVVLLVSIGVDFSYGVNSALIDI-VSRSIPGSIIQKPTDTPQDRAIKIVIHDGGKFCYAPVFSGGESYIRILQCWEQGVSNAR
WP.002741326.1bUNK/1-629	---------------MIRL-IVLIVVLLVSIGVDFSYGVNSALIDI-VSRSIPGSIIQKPTDTPQDRAIKIVIHDGGKFCYAPVFSGGESYIRILQCWEQGVSNAR
WP.193822882.1bUNK/1-632	---------------MIRL-IVLIVVLLVSIGVDFSYGVNSALIDI-VSRSIPGPIIQKPTDTPQDRAIKIVIHDGGKFCYAPVFSGGESYIRILQCWEQGVSNAR
WP.193822870.1bUNK/1-632	---------------MIRL-IVLIVVLLVSIGVDFSYGVNSALIDI-VSRSIPGPIIQKPTDTPQDRAIKIVLHDGGKFCYAPVFSGGESYIRILQCWEQGVSNAR

WP.193813123.1bUNK/1-632	---------------MIRL-IVLIVVLLVSIGVNFSYGVNSALINI-VSRSIPGPIIQKPTDTPQDRAIKIVLHDGGKFCYAPVFSGGESYIRILQCWEQGVSNAR
WP.193810541.1bUNK/1-632	---------------MIRL-IVLIVVLLVSIGVDFSYGVNSALINI-VSRSIPGPIIQKPTDTPQDRAIKIVLHDGGKFCYAPVFSGGESYIRILQCWEQGVSNAR
WP.193810402.1bUNK/1-632	---------------MIRL-IVLIVVLLVSIGVDFSYGVNSALINI-VSRSIPGPIIQKPTDTPQDRAIKIVLHDGGKFCYAPVFSGGESYIRILQCWEQGVSNAR
WP.078313332.1bUNK/1-632	---------------MIRL-IVLIVVLLVSIGVDFSYGVNSALIDI-VSRSIPGSIIQKPTDTPQDRAIKIVIHDGGKFCYAPVFSGGESYIRILQCWEQGVSNAR
WP.193824035.1bUNK/1-632	---------------MIRL-IVLIVVLLVSIGVDFSYGVNSALIDI-VSRSIPGSIIQKPTDTPQDRAIKIVIHDGGKFCYAPVFSGGESYIRILQCWEQGVSNAR
WP.002758129.1bUNK/1-632	---------------MIRL-IVLIVVLLVSIGVDFSYGVNSALINI-VSRSIPGPIIQKPTDTPQDRAIKIVLHDGGKFCYAPVFSGGESYIRILQCWEQGVSNAR
WP.002744075.1bUNK/1-632	---------------MIRL-IVLIVVLLVSIGVNFSYGVNSALINI-VSRSIPGPIIQKPTDTPQDRAIKIVLHDGGKFCYAPVFSGGESYIRILQCWEQGVSNAR
WP.016561445.1bUNK/1-632	---------------MIRL-IVLIVVLLVSIGVDFSYGVNSALINI-VSRSIPGPIIQKPTDTPQDRAIKIVLHDGGKFCYAPVFSGGESYIRILQCWEQGVSNAR
GIM25926.1bUNK_pKHM25.185/1-632	---------------MIRL-IVLIVVLLVSIGVDFSYGVNSALINI-VSRSIPGPIIQKPTDTPQDRAIKIVLHDGGKFCYAPVFSGGESYIRILQCWEQGVSNAR
WP.002737714.1bUNK/1-632	---------------MIRL-IVLIVVLLVSIGVDFSYGVNSALINI-VSRSIPGPIIQKPTDTPQDRAIKIVLHDGGKFCYAPVFSGGESYIRILQCWEQGVSNAR
PN4Z12_PN4.3935_GTG3.bPN4/1-536	---------------MIRL-IVLIVVLLVSIGVDFSYGVNSALINI-VSRSIPGPIIQKPTDTPQDRAIKIVLHDGGKFCYAPVFSGGESYIRILQCWEQGVSNAR
PN3Z12_SLPN3.3988_GTG3.bP/1-536	---------------MIRL-IVLIVVLLVSIGVDFSYGVNSALINI-VSRSIPGPIIQKPTDTPQDRAIKIVLHDGGKFCYAPVFSGGESYIRILQCWEQGVSNAR
PN1Z12_PN1.3997_GTG3.bPN1/1-536	---------------MIRL-IVLIVVLLVSIGVDFSYGVNSALINI-VSRSIPGPIIQKPTDTPQDRAIKIVLHDGGKFCYAPVFSGGESYIRILQCWEQGVSNAR
AP9Z12_AP9.4025_GTG3.bAP9/1-536	---------------MIRL-IVLIVVLLVSIGVDFSYGVNSALINI-VSRSIPGPIIQKPTDTPQDRAIKIVLHDGGKFCYAPVFSGGESYIRILQCWEQGVSNAR
AP8Z12_AP8.3908_GTG3.bAP8/1-536	---------------MIRL-IVLIVVLLVSIGVDFSYGVNSALINI-VSRSIPGPIIQKPTDTPQDRAIKIVLHDGGKFCYAPVFSGGESYIRILQCWEQGVSNAR
AP5Z12_AP5.3988_GTG3.bAP5/1-536	---------------MIRL-IVLIVVLLVSIGVDFSYGVNSALINI-VSRSIPGPIIQKPTDTPQDRAIKIVLHDGGKFCYAPVFSGGESYIRILQCWEQGVSNAR
AP4Z12_AP4.4117_GTG3.bAP4/1-536	---------------MIRL-IVLIVVLLVSIGVDFSYGVNSALINI-VSRSIPGPIIQKPTDTPQDRAIKIVLHDGGKFCYAPVFSGGESYIRILQCWEQGVSNAR
AP2Z12_AP2.4003_GTG3.bAP2/1-536	---------------MIRL-IVLIVVLLVSIGVDFSYGVNSALINI-VSRSIPGPIIQKPTDTPQDRAIKIVLHDGGKFCYAPVFSGGESYIRILQCWEQGVSNAR
UNK.yVS2413_I319370/1-633	---------------MNRF-IVLIVVLLISIGVDFSYGVSPALVHI-LSSVVSDSIVQKPTDNPKDKAIKIVIHDGGKFCYTPVFSGGESYIQLEQCWEQDVTSAR
UNK.yVS2413_I19165/1-633	---------------MNRF-IVLIVVLLISIGVDFSYGVSPALVHI-LSSVVSDSIVQKPTDNPKDKAIKIVIHDGGKFCYTPVFSGGESYIQLEQCWEQDVTSAR
UNK.yS200901116_I220189/1-633	---------------MNRF-IVLIVVLLISIGVDFSYGVSPALVHI-LSSVVSDSIVQKPTDNPKDKAIKIVIHDGGKFCYAPVFSGGESYIQLEQCWEQDVTSAR
UNK.yMDI272_Ic186914/1-633	---------------MNRF-IVLIVVLLISIGVDFSYGVSPALVHI-LSSVVSDSIVQKPTDNPKDKAIKIVIHDGGKFCYAPVFSGGESYIQLEQCWEQDVTSAR
UNK.yMDI222_I149743/1-633	---------------MNRF-IVLIVVLLISIGVDFSYGVSPALVHI-LSSVVSDSIVQKPTDNPKDKAIKIVIHDGGKFCYTPVFSGGESYIQLEQCWEQDVTSAR
WP.129533444.1yUNK/1-633	---------------MNRF-IVLIVVLLISIGVDFSYGVSPALVHI-LSSVVSDSIVQKPTDNPKDKAIKIVIHDGGKFCYAPVFSGGESYIQLEQCWEQDVTSAR
UNK.yMDI222_353992/1-633	---------------MNRF-IVLIVVLLISIGVDFSYGVSPALVHI-LSSVVSDSIVQKPTDNPKDKAIKIVIHDGGKFCYAPVFSGGESYIQLEQCWEQDVTSAR
WP.117344699.1yUNK/1-633	---------------MNRF-IVLIVVLLISIGVDFSYGVSPALVHI-LSSVVSGSIVQKPTDNPKDKAIKIVIHDGGKFCYAPVFSGGESYIQLEQCWEQDVTSAR
UNK.yS200901116_859304/1-633	---------------MNRF-IVLIVVLLISIGVDFSYGVSPALVHI-LSSVVSGSIVQKPTDNPKDKAIKIVIHDGGKFCYAPVFSGGESYIQLEQCWEQDVTSAR
UNK.yMDI272_3850663/1-633	---------------MNRF-IVLIVVLLISIGVDFSYGVSPALVHI-LSSVVSGSIVQKPTDNPKDKAIKIVIHDGGKFCYAPVFSGGESYIQLEQCWEQDVTSAR
UNK.yS200901116_855377/1-633	---------------MNRF-IVLIVVLLISIGVDFSYGVSPALVHI-LSSVVSGSIVQKPTDNPKDKAIKIVIHDGGKFCYAPVFSGGESYIQLEQCWEQDVTSAR
UNK.yMDI272_3854590/1-633	---------------MNRF-IVLIVVLLISIGVDFSYGVSPALVHI-LSSVVSGSIVQKPTDNPKDKAIKIVIHDGGKFCYAPVFSGGESYIQLEQCWEQDVTSAR
UNK.yMDI272_11174/1-633	---------------MNRF-IVLIVVLLISIGVDFSYGVSPALVHI-LSSVVSGSIVQKPTDNPKDKAIKIVIHDGGKFCYAPVFSGGESYIQLEQCWEQDVTSAR
WP.117339054.1yUNK/1-633	---------------MNRF-IVLIVVLLISIGVDFSYGVSPALVHI-LSSVVSDSIVQKPTDNPKDKAIKIVIHDGGKFCYAPVFSGGESYIQLEQCWEQDVSSAR
UNK.yVS2413_347663/1-633	---------------MNRF-IVLIVVLLISIGVDFSYGVSPALVHI-LSSVVSDSIVQKPTDNPKDKAIKIVIHDGGKFCYAPVFSGGESYIQLEQCWEQDVSSAR
UNK.wCUDO6_944011/1-632	---------------MIRL-IVPIVVLSVSIGVDLSYGVSPALIHI-LSNAIPGSIVQKPADNPKDKAIKIVIHDGGKFCYAPVFSGGESYIQIEQCWEQGVANAR
UNK.wCUD13_917673/1-632	---------------MIRL-IVPIVVLSVSIGVDLSYGVSPALIHI-LSNAIPGSIVQKPADNPKDKAIKIVIHDGGKFCYAPVFSGGESYIQIEQCWEQGVANAR
WP.004500844.1wUNK/1-634	---------------MSRL-IVPIVVLSVSIGVDLSYGVSPALIHI-LSNAIPGSIVQKPADNPKDKAIKIVIHDGGKFCYVPVFSGGESYIQIEQCWEQGVANAR
RT1X12_RT1.459_GCG2.wRT1-/1-634	---------------MIRL-IVPIVVLSVSIGVDLSYGVSPALIHI-LSNAIPGSIVQKPADNPKDKAIKIVIHDGGKFCYVPVFSGGESYIQIEQCWEQGVANAR
RT1X11_RT1.228_GCG2.wRT1-/1-634	---------------MIRL-IVPIVVLSVSIGVDLSYGVSPALIHI-LSNAIPGSIVQKPADNPKDKAIKIVIHDGGKFCYVPVFSGGESYIQIEQCWEQGVANAR
PD2X12_PD2.1568_GTG6.wPD2/1-632	---------------MIRL-IVPIVVLSVSIGVDLSYGVSPALIHI-LSNAIPGSIVQKPADNPKDKAIKIVIHDGGKFCYAPVFSGGESYIQIEQCWEQGVANAR
RT1X13_RT1.1572_GCG2.wRT1/1-632	---------------MIRL-IVPIVVLSVSIGVDLSYGVSPALIHI-LSNAIPGSIVQKPADNPKDKAIKIVIHDGGKFCYAPVFSGGESYIQIEQCWEQGVANAR
PD2X11_PD2.494_GTG6.wPD2-/1-633	---------------MIRL-IVPIVVLSVSIGVDLSYGVSPALIHI-LSNAIPGSIVQKPADNPKDKAIKIVIHDGGKFCYVPVFSGGESYIQIEQCWEQGVANAR
WP.017850947.1iUNK/1-636	---------------MCNW-KILIVILLVSIGVGFEYGMDHTLVHA-SSSKSDYSIVQKSTDQPKDKPIQVVVSDKGKFCYGPTFSGGESYIQIEQCWERHVMNAR
WP.141598375.1nUNK/1-637	---------------MYNWKKVFIVVLLVSIGVGFEYGESHTRVHAASSSKSNYSIVQRPTDKPKDKPIQVVVSDGGKFCYGPIFSGGESYIQIEQCWERHVKNAR
WP.004445236.1nUNK/1-634	---------------MRNWKKMLIVVLLVSIGVGFEYSINPTPVHA--SSKANDSIVQKPTDPPKDKPIKIVVSNKGKYCYSPVFTKGEGYVWIDKCTDK-TAKAR
WP.016559986.1nUNK/1-634	---------------MRCW-KVLVVVLLVSILLSFRYGINV---HA--SSKANDSIVQKPTDPPKDKPIKIVVSNKGKYCYSPVFTKGEGYVWIDKCTDK-TAKAR
WP.004433833.1nUNK/1-633	---------------------MLIVVLLVSIGVGFEYSINPAPVHA-SSSKVNSSIVQKPTDKPKDKPIKIVVHDGGKYCYSPVFTNGEGYVWIDKCTDK-TAKAR
WP.020980207.1nUNK/1-634	---------------MRNWKKMLIVVLLVSIGVGFEYSINPTPVHA--SSKANNSIVQKPTDPPKDKPIKVVVSNKKAYCYSPAFTKGEGYVWIDTCTDK-IAKAR
WP.004440492.1nUNK/1-633	---------------MRNWKKMLIVVLLVSIGVGFEYSINPTPVHA--SSKANNSIVQKPTDPPKDKPIKVVVSNKKAYCYSPAFTKGEGYVWIDTCTDK-IAKAR
WP.004422402.1nUNK/1-634	---------------MCNWKKELLVVLLVSVGTILEYGINYTFVHA-SSSKVNSSIVQKPTDPPKDKPIKVVVSNKKAYCYSPVFTKGEGYVWIDNCTDK-TAKAR
WP.053522774.1nUNK/1-628	---------------MSRC-IVLRAFLLVLIGVGFEYGINHTSVNA--SPKSDYSIVQKPTDPPKDKPIKVVVSNKKAYCYSPAFTKGEGYVWIDKCTDK-TPKAR
WP.203368386.1iUNK/1-627	---------------MGRW-IVLRVSLLVLIGIGFEYGINHTSINA--SSKSDYSIAQKPADQPKDKSIKVVMHGGSNYCYSPVFTKGEGYIWIDYCSDN-TAKAR
WP.137119391.1iUNK/1-633	---------------MGRW-IVLRVSLLVLIGIGFEYGINHTSINA--SSKSDYSIAQKPADQPKDKSIQVVMHGGSKYCYSPVFTKGEGYIWIDYCRDN-TAKAR
WP.061270981.1iUNK/1-627	---------------MGRW-IVLRVSLLVLIGIGFEYGINHTSINA--SSKSDYSIAQKPADQPKDKSIQVVMHGGSNYCYSPVFTKGEGYIWIDYCSDN-TAKAR
WP.061269786.1iUNK/1-627	---------------MGRW-IVLRVSLLVLIGIGFEYGINHTSINA--SSKSDYSIAQKPADQPKDKSIKVVMHGGSNYCYSPVFTKGEGYIWIDYCRDN-TAKAR
WP.061243274.1iUNK/1-627	---------------MGRW-IVLRVSLLVLIGIGFEYGINHTSINA--SSKSDYSIAQKPADQPKDKSIKVVMHGGSNYCYSPVFTKGEGYIWIDYCRDN-TAKAR
WP.025179862.1iUNK/1-625	---------------MGNW-KNLVVVLLVSIGVGFGY---HTLIHA-SSSKANYSIAQKPTDPPKDKPINIVTHDGKTYCYSPVFSKGEGYVWIEKCGDN-TAKAR
WP.025179748.1iUNK/1-627	---------------MGRW-IVLRVSLLVLIGIGFEYGINHTSINA--SSKSDYSIAQKPADQPKDKSIKVVMHGGSNYCYSPVFTKGEGYIWIDYCRDN-TAKAR
WP.002127288.1iUNK/1-627	---------------MGRW-IVLRVSLLVLIGIGFEYGINHTSINA--SSKSDYSIAQKPADQPKDKSIKVVMHGGSNYCYSPVFTKGEGYIWIDYCRDN-TAKAR
WP.000534009.1iUNK/1-627	---------------MGRW-IVLRVSLLVLIGIGFEYGINHTSINA--SSKSDYSIAQKPADQPKDKSIKVVMHGGSNYCYSPVFTKGEGYIWIDYCRDN-TAKAR
PN3Q19_SLPN3.2805_AG3.iPN/1-627	---------------MGRW-IVLRVSLLVLIGIGFEYGINHTSINA--SSKSDYSIAQKPADQPKDKSIQVVMHGGSNYCYSPVFTKGEGYIWIDYCSDN-TAKAR
LAI.iS56601_1395688/1-627	---------------MGRW-IVLRVSLLVLIGIGFEYGINHTSINA--SSKSDYSIAQKPADQPKDKSIKVVMHGGSNYCYSPVFTKGEGYIWIDYCRDN-TAKAR
LAI.iIPAV_1394759/1-627	---------------MGRW-IVLRVSLLVLIGIGFEYGINHTSINA--SSKSDYSIAQKPADQPKDKSIKVVMHGGSNYCYSPVFTKGEGYIWIDYCRDN-TAKAR
KG1Q1A_KG1.4082_AG14.uKG1/1-627	---------------MGRW-IVLRVSLLVLIGIGFEYGINHTSINA--SSKSDYSIAQKPADQPKDKSIKVVMHGGSNYCYSPVFTKGEGYIWIDYCRDN-TAKAR
ICT.iLangkawi_2889455/1-627	---------------MGRW-IVLRVSLLVLIGIGFEYGINHTSINA--SSKSDYSIAQKPADQPKDKSIKVVMHGGSNYCYSPVFTKGEGYIWIDYCRDN-TAKAR
AW1Q19_AW1.2739_AG3.iAW1-/1-633	---------------MGRW-IVLRVSLLVLIGIGFEYGINHTSINA--SSKSDYSIAQKPADQPKDKSIQVVMHGGSKYCYSPVFTKGEGYIWIDYCRDN-TAKAR
AP7Q19_AP7.2707_AG3.iAP7-/1-627	---------------MGRW-IVLRVSLLVLIGIGFEYGINHTSINA--SSKSDYSIAQKPADQPKDKSIQVVMHGGSNYCYSPVFTKGEGYIWIDYCSDN-TAKAR
AP3Q19_AP3.2805_AG10.iAP3/1-627	---------------MGRW-IVLRVSLLVLIGIGFEYGINHTSINA--SSKSDYSIAQKPADQPKDKSIQVVMHGGSNYCYSPVFTKGEGYIWIDYCSDN-TAKAR
AP1Q18_AP1.3259_AG3.iAP1-/1-627	---------------MGRW-IVLRVSLLVLIGIGFEYGINHTSINA--SSKSDYSIAQKPADQPKDKSIQVVMHGGSNYCYSPVFTKGEGYIWIDYCSDN-TAKAR
AG3Q19_U1.2805_AG3.iO7371/1-627	---------------MGRW-IVLRVSLLVLIGIGFEYGINHTSINA--SSKSDYSIAQKPADQPKDKSIQVVMHGGSNYCYSPVFTKGEGYIWIDYCSDN-TAKAR
WP.192505596.1iUNK/1-627	---------------MGRW-IVLRVSLLVLIGIGFEYGINHTSINA--SSKSDYSIAQKPADQPKDKSIKVVMHGGSNYCYSPVFTKGEGYIWIDYCRDN-TAKAR
WP.061261833.1iUNK/1-627	---------------MGRW-IVLRVSLLVLIGIGFEYGINHTSINA--SSKSDYSIAQKPADQPKDKSIQVVMHGGSNYCYSPVFTKGEGYIWIDYCSDN-TAKAR
CLA.iS782_1564345/1-627	---------------MGRW-IVLRVSLLVLIGIGFEYGINHTSINA--SSKSDYSIAQKPADQPKDKSIKVVMHGGSNYCYSPVFTKGEGYIWIDYCRDN-TAKAR
WP.061270460.1iUNK/1-627	---------------MGRW-IVLRVSLLVLIGIGFEYGINHTSINA--SSKSDYSIAQKPADQPKDKSIQVVMHGGSNYCYSPVFTKGEGYIWIDYCSDN-TAKAR
WP.061266364.1iUNK/1-627	---------------MGRW-IVLRVSLLVLIGIGFEYGINHTSINA--SSKSDYSIAQKPADQPKDKSIQVVMHDGSNYCYSPVFTKGEGYIWIDYCSDN-TAKAR
WP.061260456.1iUNK/1-627	---------------MGRW-IVLRVSLLVLIGIGFEYGINHTSINA--SSKSDYSIAQKPADQPKDKSIQVVMYGGSNYCYSPVFTKGEGYIWIDYCSDN-TAKAR
WP.061234571.1iUNK/1-627	---------------MGRW-IVLRVSLLVLIGIGFEYGINHTSINA--SSKSDYSIAQKPTDQPKDKSIQVVMHDGSKYCYSPVFTKGEGYVWIDYCRDN-TAKAR
WP.061226807.1iUNK/1-629	---------------MGRW-IVLRVSLLVLIGIGFEYGINHTSINA--SSKSDYSIAQKPADQPKDKSIQVVMHGGSNYCYSPVFTKGEGYIWIDYCSDN-TAKAR
WP.061226106.1iUNK/1-627	---------------MGRW-IVLRVSLLVLIGIGFEYGINHTSINA--SSKSDYSIAQKPADQPKDKSIQVVMHGGSNYCYSPVFTKGEGYIWIDYCSDN-TAKAR
WP.025185498.1iUNK/1-627	---------------MGRW-IVLRVSLLVLIGIGFEYGINHTSINA--SSKSDYSIAQKPADQPKDKSIQVVMHGGSNYCYSPVFTKGEGYIWIDYCSDN-TAKAR
WP.025177736.1iUNK/1-627	---------------MGRW-IVLRVSLLVLIGIGFEYGINHTSINA--SSKSDYSIAQKPADQPKDKSIKVVMHGGSNYCYSPVFTKGEGYIWIDYCRDN-TAKAR
WP.025177478.1iUNK/1-627	---------------MGRW-IVLRVSLLVLIGIGFEYGINHTSINA--SSKSDYSIAQKPADQPKDKSIKVVMHGGSNYCYSPVFTKGEGYIWIDYCRDN-TAKAR
WP.000534023.1iUNK/1-627	---------------MGRW-IVLRVSLLVLIGIGFEYGINHTSINA--SSKSDYSIAQKPADQPKDKSIQVVMHGGSNYCYSPVFTKGEGYIWIDYCSDN-TAKAR
WP.000534015.1iUNK/1-627	---------------MGRW-IVLRVSLLVLIGIGFEYGINHTSINA--SSKSDYSIAQKPADQPKDKSIQVVMHGGSNYCYSPVFTKGEGYIWIDYCSDN-TAKAR
WP.000534014.1iUNK/1-627	---------------MGRW-IVLRVSLLVLIGIGFEYGINHTSINA--SSKSDYSIAQKPADQPKDKSIQVVMHGGSNYCYSPVFTKGEGYIWIDYCSDN-TAKAR
WP.000534013.1iUNK/1-627	---------------MGRW-IVLRVSLLVLIGIGFEYGINHTSINA--SSKSDYSIAQKPADQPKDKSIQVVMHGGSNYCYSPVFTKGEGYIWIDYCSDN-TAKAR
UNK.iR7_2787093/1-627	---------------MGRW-IVLRVSLLVLIGIGFEYGINHTSINA--SSKSDYSIAQKPADQPKDKSIQVVMHGGSNYCYSPVFTKGEGYIWIDYCSDN-TAKAR
UNK.iR22_2796934/1-627	---------------MGRW-IVLRVSLLVLIGIGFEYGINHTSINA--SSKSDYSIAQKPADQPKDKSIQVVMHGGSNYCYSPVFTKGEGYIWIDYCSDN-TAKAR
UNK.iR21_2797605/1-627	---------------MGRW-IVLRVSLLVLIGIGFEYGINHTSINA--SSKSDYSIAQKPADQPKDKSIQVVMHGGSNYCYSPVFTKGEGYIWIDYCSDN-TAKAR
UNK.iR19_2796919/1-627	---------------MGRW-IVLRVSLLVLIGIGFEYGINHTSINA--SSKSDYSIAQKPADQPKDKSIQVVMHGGSNYCYSPVFTKGEGYIWIDYCSDN-TAKAR
UNK.iR17_2797850/1-627	---------------MGRW-IVLRVSLLVLIGIGFEYGINHTSINA--SSKSDYSIAQKPADQPKDKSIQVVMHGGSNYCYSPVFTKGEGYIWIDYCSDN-TAKAR
UNK.iR16_2788045/1-627	---------------MGRW-IVLRVSLLVLIGIGFEYGINHTSINA--SSKSDYSIAQKPADQPKDKSIQVVMHGGSNYCYSPVFTKGEGYIWIDYCSDN-TAKAR
UNK.iR13_2788309/1-627	---------------MGRW-IVLRVSLLVLIGIGFEYGINHTSINA--SSKSDYSIAQKPADQPKDKSIQVVMHGGSNYCYSPVFTKGEGYIWIDYCSDN-TAKAR
UNK.iR13L_2805755/1-627	---------------MGRW-IVLRVSLLVLIGIGFEYGINHTSINA--SSKSDYSIAQKPADQPKDKSIQVVMHGGSNYCYSPVFTKGEGYIWIDYCSDN-TAKAR
UNK.iR12_2795657/1-627	---------------MGRW-IVLRVSLLVLIGIGFEYGINHTSINA--SSKSDYSIAQKPADQPKDKSIQVVMHGGSNYCYSPVFTKGEGYIWIDYCSDN-TAKAR
UNK.iR11_2788484/1-627	---------------MGRW-IVLRVSLLVLIGIGFEYGINHTSINA--SSKSDYSIAQKPADQPKDKSIQVVMHGGSNYCYSPVFTKGEGYIWIDYCSDN-TAKAR
PD1Q17_PD1.3305_AG3.iPD1-/1-627	---------------MGRW-IVLRVSLLVLIGIGFEYGINHTSINA--SSKSDYSIAQKPADQPKDKSIQVVMHGGSNYCYSPVFTKGEGYIWIDYCSDN-TAKAR
LHI.iS56609_1486974/1-627	---------------MGRW-IVLRVSLLVLIGIGFEYGINHTSINA--SSKSDYSIAQKPADQPKDKSIQVVMHGGSNYCYSPVFTKGEGYIWIDYCSDN-TAKAR
ICT.iS898_2832150/1-627	---------------MGRW-IVLRVSLLVLIGIGFEYGINHTSINA--SSKSDYSIAQKPADQPKDKSIQVVMHGGSNYCYSPVFTKGEGYIWIDYCSDN-TAKAR
HJO.iNorma_1491902/1-627	---------------MGRW-IVLRVSLLVLIGIGFEYGINHTSINA--SSKSDYSIAQKPADQPKDKSIKVVMHGGSNYCYSPVFTKGEGYIWIDYCRDN-TAKAR
HJO.iL53_1519604/1-627	---------------MGRW-IVLRVSLLVLIGIGFEYGINHTSINA--SSKSDYSIAQKPADQPKDKSIKVVMHGGSNYCYSPVFTKGEGYIWIDYCRDN-TAKAR
HJO.iHjtno_1514705/1-627	---------------MGRW-IVLRVSLLVLIGIGFEYGINHTSINA--SSKSDYSIAQKPADQPKDKSIKVVMHGGSNYCYSPVFTKGEGYIWIDYCRDN-TAKAR
COP.iSK1_2832190/1-627	---------------MGRW-IVLRVSLLVLIGIGFEYGINHTSINA--SSKSDYSIAQKPADQPKDKSIQVVMHGGSNYCYSPVFTKGEGYIWIDYCSDN-TAKAR
COP.iL1130_Q72PX7.cl2831/1-627	---------------MGRW-IVLRVSLLVLIGIGFEYGINHTSINA--SSKSDYSIAQKPADQPKDKSIQVVMHGGSNYCYSPVFTKGEGYIWIDYCSDN-TAKAR
COP.iFDAARGOS203_841506/1-627	---------------MGRW-IVLRVSLLVLIGIGFEYGINHTSINA--SSKSDYSIAQKPADQPKDKSIQVVMHGGSNYCYSPVFTKGEGYIWIDYCSDN-TAKAR
BAE.iS1489_2918822/1-627	---------------MGRW-IVLRVSLLVLIGIGFEYGINHTSINA--SSKSDYSIAQKPADQPKDKSIKVVMHGGSNYCYSPVFTKGEGYIWIDYCRDN-TAKAR
[illegible]	[illegible]

```
AW3Q15_AW3.1878_AG10.iAW3/1-627 ---------------MGRW-IVLRVSLLVLIGIGFEYGINHTSINA--SSKSDYSIAQKPADQPKDKSIQVVMHGGSNYCYSPVFTKGEGYIWIDYCSDN-TAKAR
AP6Q15_AP6.1583_AG3.iAP6-/1-627 ---------------MGRW-IVLRVSLLVLIGIGFEYGINHTSINA--SSKSDYSIAQKPADQPKDKSIQVVMHGGSNYCYSPVFTKGEGYIWIDYCSDN-TAKAR
AP5Q15_AP5.1531_AG10.iAP5/1-627 ---------------MGRW-IVLRVSLLVLIGIGFEYGINHTSINA--SSKSDYSIAQKPADQPKDKSIQVVMHGGSNYCYSPVFTKGEGYIWIDYCSDN-TAKAR
WP.061257066.1iUNK/1-627 ---------------MGRW-IVLRVSLLVLIGIGFEYGINHTSINA--SSKSDYSIAQKPADQPKDKSIQVVMHGGSNYCYSPVFTKGEGYIWIDYCSDN-TAKAR
WP.000534016.1iUNK/1-627 ---------------MGRW-IVLRVSLLVLIGIGFEYGINHTSINA--SSKSDYSIAQKPADQPKDKSIQVVMHGGSNYCYSPVFTKGEGYIWIDYCSDN-TAKAR
BAE.iS1548_1507940/1-627 ---------------MGRW-IVLRVSLLVLIGIGFEYGINHTSINA--SSKSDYSIAQKPADQPKDKSIQVVMHGGSNYCYSPVFTKGEGYIWIDYCSDN-TAKAR
ICT.iPiscina_2790690/1-627 ---------------MGRW-IVLRVSLLVLIGIGFEYGINHTSINA--SSKSDYSIAQKPADQPKDKSIQVVMHGGSNYCYSPVFTKGEGYIWIDYCSDN-TAKAR
APH42156.1iICT_pA9P81.250/1-627 ---------------MGRW-IVLRVSLLVLIGIGFEYGINHTSINA--SSKSDYSIAQKPADQPKDKSIQVVMHGGSNYCYSPVFTKGEGYIWIDYCSDN-TAKAR
WP.025177425.1iUNK/1-627 ---------------MGRW-IVLRVSLLVLIGIGFEYGINHTSINA--SSKSDYSIAQKPADQPKDKSIQVVMHGGSNYCYSPVFTKGEGYIWIDYCSDN-TAKAR
WP.061241258.1iUNK/1-627 ---------------MGRW-IVLRVSLLVLIGIGFEYGINHTPINA--SSKSDYSIAQKPADQPKDKSIQVVMHGGSKYCYSPVFTKGEGYIWIDYCRDN-TAKAR
WP.010678767.1iUNK/1-627 ---------------MGRW-IVLRVSLLVLIGIGFEYGINHTSINA--SSKSNYSIAQKPTDQPKDKSIKVVMYGGSKYCYSPVFTKGEGYVWIDNCRDN-TAKAR
WP.000534024.1iUNK/1-627 ---------------MGRW-IVLRVSLLVLIGIGFEYGINHTSINA--SSKSNYSIAQKPTDQPKDKSIKVVMYGGSKYCYSPVFTKGEGYVWIDNCRDN-TAKAR
MAE.iUPMMCNIIDLP_2775300/1-627 ---------------MGRW-IVLRVSLLVLIGIGFEYGINHTSINA--SSKSNYSIAQKPTDQPKDKSIKVVMYGGSKYCYSPVFTKGEGYVWIDNCRDN-TAKAR
MAE.iUPMMCNIIDHP_2775301/1-627 ---------------MGRW-IVLRVSLLVLIGIGFEYGINHTSINA--SSKSNYSIAQKPTDQPKDKSIKVVMYGGSKYCYSPVFTKGEGYVWIDNCRDN-TAKAR
WP.025184547.1iUNK/1-649 ---------------MGRW-IVLRVSLLVLIGIGFEYGINHTSINA--SSKSDYSIAQKPADQPKDKSIQVVMHGGSNYCYSPVFTKGEGYIWIDYCSDN-TAKAR
WP.017862335.1iUNK/1-629 ---------------MGRW-IVLRVSLLVLIGIGFEYGINHTSINA--SSKSDYSIAQKPADQPKDKSIQVVMHGGSNYCYSPVFTKGEGYIWIDYCSDN-TAKAR
EMF44677.1iLRA_sTE1992/1-627 ---------------MGRW-IVLRVSLLVLIGIGFEYGINHTSINA--SSKSDYSIAQKPADQPKDKSIQVVMHGGSNYCYSPVFTKGEGYIWIDYCSDN-TAKAR
BVA.iPigK151_2926905/1-629 ---------------MGRW-IVLRVSLLVLIGIGFEYGINHTSINA--SSKSDYSIAQKPADQPKDKSIQVVMHGGSNYCYSPVFTKGEGYIWIDYCSDN-TAKAR
WP.137108219.1iUNK/1-627 ---------------MGRW-IVLRVSLLVLIGIGFEYGINHTSINA--SSKSDYSIAQKPADQPKDKSIQVVMHGGSKYCYSPVFTKGEGYIWIDYCRDN-TAKAR
RT2Q19_RT2.2773_AG3.iRT2-/1-627 ---------------MGRW-IVLRVSLLVLIGIGFEYGINHTSINA--SSKSDYSIAQKPADQPKDKSIQVVMHGGSKYCYSPVFTKGEGYIWIDYCRDN-TAKAR
KW2Q19_KW2.2742_AG3.iKW2-/1-627 ---------------MGRW-IVLRVSLLVLIGIGFEYGINHTSINA--SSKSDYSIAQKPADQPKDKSIQVVMHGGSKYCYSPVFTKGEGYIWIDYCRDN-TAKAR
KW2Q19_KW2.2726_AG3.iKW2-/1-627 ---------------MGRW-IVLRVSLLVLIGIGFEYGINHTSINA--SSKSDYSIAQKPADQPKDKSIQVVMHGGSKYCYSPVFTKGEGYIWIDYCRDN-TAKAR
KW1Q15_KW1.1407_AG3.iKW1-/1-627 ---------------MGRW-IVLRVSLLVLIGIGFEYGINHTSINA--SSKSDYSIAQKPADQPKDKSIQVVMHGGSKYCYSPVFTKGEGYIWIDYCRDN-TAKAR
WP.061286125.1iUNK/1-627 ---------------MGHW-IVLRVFLLVLIGIGFEYGINDTSINA--SSKSDYSIAQKPADQPKDKSIQVVMHGGSNYCYSPVFTKGEGYIWIDYCSDN-TAKAR
WP.025181059.1iUNK/1-627 ---------------MGHW-IVLRVFLLVLIGIGFEYGINDTSINA--SSKSNYSIAQKPTDQPKDKSIQVVMYDGSKYCYSPVFTKGEGYVWIDYCSDK-TAKAR
WP.061245522.1iUNK/1-627 ---------------MGRW-IVLRVFLLVLIGIGFEYGINHTSINA--SSKSNYSIAQKPTDQPKDKSIQVVMHDGSKYCYSPVFTKGEGYVWIDYCRDK-TAKAR
WP.025176071.1iUNK/1-629 ---------------MGRW-IVLRVFLLVLIGIGFEYGINHTSINA--SSKSNYSIAQKPTDQPKDKSIQVVMHDGSKYCYSPVFTKGEGYVWIDYCRDK-TAKAR
WP.017861638.1iUNK/1-627 ---------------MGRW-IVLRVFLLVLIGIGFEYGINHTSINA--SSKSNYSIAQKPTDQPKDKSIQVVMHDGSKYCYSPVFTKGEGYVWIDYCRDK-TAKAR
WP.000534007.1iUNK/1-627 ---------------MGRW-IVLRVFLLVLIGIGFEYGINHTSINA--SSKSNYSIAQKPTDQPKDKSIQVVMHDGSKYCYSPVFTKGEGYVWIDYCRDK-TAKAR
PN2Q19_PN2.3727_AG14.iPN2/1-627 ---------------MGRW-IVLRVFLLVLIGIGFEYGINHTSINA--SSKSNYSIAQKPTDQPKDKSIQVVMHDGSKYCYSPVFTKGEGYVWIDYCRDK-TAKAR
CLA.iS611_1427947/1-627 ---------------MGRW-IVLRVFLLVLIGIGFEYGINHTSINA--SSKSNYSIAQKPTDQPKDKSIQVVMHDGSKYCYSPVFTKGEGYVWIDYCRDK-TAKAR
CLA.iS114_3541578/1-627 ---------------MGRW-IVLRVFLLVLIGIGFEYGINHTSINA--SSKSNYSIAQKPTDQPKDKSIQVVMHDGSKYCYSPVFTKGEGYVWIDYCRDK-TAKAR
CLA.iRUFN_675389/1-627 ---------------MGRW-IVLRVFLLVLIGIGFEYGINHTSINA--SSKSNYSIAQKPTDQPKDKSIQVVMHDGSKYCYSPVFTKGEGYVWIDYCRDK-TAKAR
CLA.iLJ178_1432584/1-625 ---------------MGRW-IVLRVFLLVLIGIGFEYGINHTSINA--SSKSNYSIAQKPTDQPKDKSIQVVMHDGSKYCYSPVFTKGEGYVWIDYCRDK-TAKAR
WP.061246552.1iUNK/1-627 ---------------MGRW-IVLRVFLLVLIGIGFEYGINHTSINA--SSKSNYSIAQKPTDQPKDKSIQVVMHDGSKYCYSPVFTKGEGYVWIDYCRDK-IAKAR
WP.061230616.1iUNK/1-627 ---------------MGRW-IVLRVFLLVLIGIGFEYGINHTSINA--SSKSNYSIAQKPTDQPKDKSIQVVMHDGSKYCYSPVFTKGEGYVWIDYCSDK-TAKAR
WP.000534006.1iUNK/1-627 ---------------MGRW-IVLRVFLLVLIGIGFEYGINHTSINA--SSKSNYSIAQKPTDQPKDKSIQVVMHDGSKYCYSPVFTKGEGYVWIDYCRDK-TAKAR
WP.061250250.1iUNK/1-627 ---------------MGRW-IVLRVFLLVLIGIGFEYGINHTSINA--SSKSNYSIAQKPTDQPKDKSIQVVMHDGSKYCYSPVFTKGEGYVWIDYCRDK-TAKAR
WP.192486374.1iUNK/1-641 ---------------MGNW-KNLVVVLLVSIGVGFGY---HTLIHA-SSSKANYSIAQKPTDPPKDKPINIVTHDGKTYCYSPVFSKGEGYVWIEKCGDN-TAKAR
WP.061286809.1iUNK/1-641 ---------------MGNW-KNLVVVLLVSIGVGFGY---HTLIHA-SSSKANYSIAQKPTDPPKDKPINIVTHDGKTYCYSPVFSKGEGYVWIEKCGDN-TAKAR
WP.045191789.1iUNK/1-641 ---------------MGNW-KNLVVVLLVSIGVGFGY---HTLIHA-SSSKANYSIAQKPTDPPKDKPINIVTHDGKTYCYSPVFSKGEGYVWIEKCGDN-TAKAR
WP.000528831.1iUNK/1-641 ---------------MGNW-KNLVVVLLVSIGVGFGY---HTLIHA-SSSKANYSIAQKPTDPPKDKPINIVTHDGKTYCYSPVFSKGEGYVWIEKCGDN-TAKAR
LHI.iS56609_1489841/1-641 ---------------MGNW-KNLVVVLLVSIGVGFGY---HTLIHA-SSSKANYSIAQKPTDPPKDKPINIVTHDGKTYCYSPVFSKGEGYVWIEKCGDN-TAKAR
ICT.iLangkawi_2886588/1-641 ---------------MGNW-KNLVVVLLVSIGVGFGY---HTLIHA-SSSKANYSIAQKPTDPPKDKPINIVTHDGKTYCYSPVFSKGEGYVWIEKCGDN-TAKAR
CLA.iS782_1567213/1-641 ---------------MGNW-KNLVVVLLVSIGVGFGY---HTLIHA-SSSKANYSIAQKPTDPPKDKPINIVTHDGKTYCYSPVFSKGEGYVWIEKCGDN-TAKAR
WP.061266365.1iUNK/1-641 ---------------MGNW-KNLVVVLLVSIGVGFGY---HTLIHA-SSSKANYSIAQKPTDPPKDKPINIVTHDGKTYCYSPVFSKGEGYVWIEKCGDN-TAKAR
WP.061226109.1iUNK/1-641 ---------------MGNW-KNLVVVLLVSIGVGFGY---HTLIHA-SSSKANYSIAQKPTDPPKDKPINIVTHDGKTYCYSPVFSKGEGYVWIEKCGDN-TAKAR
WP.017855795.1iUNK/1-643 ---------------MGRW-IVLRVSLLVLIGIGFEYGINHTSINA--SSKSDYSIAQKPADQPKDKSIQVVMHGGSNYCYSPVFTKGEGYIWIDYCSDN-TAKAR
WP.002126984.1iUNK/1-641 ---------------MGNW-KNLVVVLLVSIGVGFGY---HTLIHA-SSSKANYSIAQKPTDPPKDKPINIVTHDGKTYCYSPVFSKGEGYVWIEKCGDN-TAKAR
WP.002089922.1iUNK/1-641 ---------------MGNW-KNLVVVLLVSIGVGFGY---HTLIHA-SSSKANYSIAQKPTDPPKDKPINIVTHDGKTYCYSPVFSKGEGYVWIEKCGDN-TAKAR
WP.001974454.1iUNK/1-641 ---------------MGNW-KNLVVVLLVSIGIGFGY---HTLIHA-SSSKANYSIAQKPTDPPKDKPINIVTHDGKTYCYSPVFSKGEGYVWIEKCGDN-TAKAR
WP.000528832.1iUNK/1-641 ---------------MGNW-KNLVVVLLVSIGVGFGY---HTLIHA-SSSKANYSIAQKPTDPPKDKPINIVTHDGKTYCYSPVFSKGEGYVWIEKCGDN-TAKAR
WP.000528829.1iUNK/1-641 ---------------MGNW-KNLVVVLLVSIGVGFGY---HTLIHA-SSSKANYSIAQKPTDPPKDKPINIVTHDGKTYCYSPVFSKGEGYVWIEKCGDN-TAKAR
UNK.iRCA_3247964/1-641 ---------------MGNW-KNLVVVLLVSIGVGFGY---HTLIHA-SSSKANYSIAQKPTDPPKDKPINIVTHDGKTYCYSPVFSKGEGYVWIEKCGDN-TAKAR
UNK.iR7_2784225/1-641 ---------------MGNW-KNLVVVLLVSIGVGFGY---HTLIHA-SSSKANYSIAQKPTDPPKDKPINIVTHDGKTYCYSPVFSKGEGYVWIEKCGDN-TAKAR
UNK.iR22_2794066/1-641 ---------------MGNW-KNLVVVLLVSIGVGFGY---HTLIHA-SSSKANYSIAQKPTDPPKDKPINIVTHDGKTYCYSPVFSKGEGYVWIEKCGDN-TAKAR
UNK.iR21_2794737/1-641 ---------------MGNW-KNLVVVLLVSIGVGFGY---HTLIHA-SSSKANYSIAQKPTDPPKDKPINIVTHDGKTYCYSPVFSKGEGYVWIEKCGDN-TAKAR
UNK.iR19_2794051/1-641 ---------------MGNW-KNLVVVLLVSIGVGFGY---HTLIHA-SSSKANYSIAQKPTDPPKDKPINIVTHDGKTYCYSPVFSKGEGYVWIEKCGDN-TAKAR
UNK.iR17_2794982/1-641 ---------------MGNW-KNLVVVLLVSIGVGFGY---HTLIHA-SSSKANYSIAQKPTDPPKDKPINIVTHDGKTYCYSPVFSKGEGYVWIEKCGDN-TAKAR
UNK.iR16_2785177/1-641 ---------------MGNW-KNLVVVLLVSIGVGFGY---HTLIHA-SSSKANYSIAQKPTDPPKDKPINIVTHDGKTYCYSPVFSKGEGYVWIEKCGDN-TAKAR
UNK.iR13_2785441/1-641 ---------------MGNW-KNLVVVLLVSIGVGFGY---HTLIHA-SSSKANYSIAQKPTDPPKDKPINIVTHDGKTYCYSPVFSKGEGYVWIEKCGDN-TAKAR
UNK.iR13L_2802887/1-641 ---------------MGNW-KNLVVVLLVSIGVGFGY---HTLIHA-SSSKANYSIAQKPTDPPKDKPINIVTHDGKTYCYSPVFSKGEGYVWIEKCGDN-TAKAR
UNK.iR12_2792789/1-641 ---------------MGNW-KNLVVVLLVSIGVGFGY---HTLIHA-SSSKANYSIAQKPTDPPKDKPINIVTHDGKTYCYSPVFSKGEGYVWIEKCGDN-TAKAR
UNK.iR11_2785616/1-641 ---------------MGNW-KNLVVVLLVSIGVGFGY---HTLIHA-SSSKANYSIAQKPTDPPKDKPINIVTHDGKTYCYSPVFSKGEGYVWIEKCGDN-TAKAR
PN3Q18_SLPN3.2803_AG3.iPN/1-641 ---------------MGNW-KNLVVVLLVSIGVGFGY---HTLIHA-SSSKANYSIAQKPTDPPKDKPINIVTHDGKTYCYSPVFSKGEGYVWIEKCGDN-TAKAR
PD1Q16_PD1.3303_AG3.iPD1-/1-641 ---------------MGNW-KNLVVVLLVSIGVGFGY---HTLIHA-SSSKANYSIAQKPTDPPKDKPINIVTHDGKTYCYSPVFSKGEGYVWIEKCGDN-TAKAR
LAI.iS56601_1398555/1-641 ---------------MGNW-KNLVVVLLVSIGVGFGY---HTLIHA-SSSKANYSIAQKPTDPPKDKPINIVTHDGKTYCYSPVFSKGEGYVWIEKCGDN-TAKAR
LAI.iIPAV_1397626/1-641 ---------------MGNW-KNLVVVLLVSIGVGFGY---HTLIHA-SSSKANYSIAQKPTDPPKDKPINIVTHDGKTYCYSPVFSKGEGYVWIEKCGDN-TAKAR
KG2Q18_KG2.3777_AG3.uKG2-/1-641 ---------------MGNW-KNLVVVLLVSIGVGFGY---HTLIHA-SSSKANYSIAQKPTDPPKDKPINIVTHDGKTYCYSPVFSKGEGYVWIEKCGDN-TAKAR
ICT.iS898_2829282/1-641 ---------------MGNW-KNLVVVLLVSIGVGFGY---HTLIHA-SSSKANYSIAQKPTDPPKDKPINIVTHDGKTYCYSPVFSKGEGYVWIEKCGDN-TAKAR
HJO.iNorma_1494769/1-641 ---------------MGNW-KNLVVVLLVSIGVGFGY---HTLIHA-SSSKANYSIAQKPTDPPKDKPINIVTHDGKTYCYSPVFSKGEGYVWIEKCGDN-TAKAR
HJO.iL53_1522471/1-641 ---------------MGNW-KNLVVVLLVSIGVGFGY---HTLIHA-SSSKANYSIAQKPTDPPKDKPINIVTHDGKTYCYSPVFSKGEGYVWIEKCGDN-TAKAR
HJO.iHjtno_1517572/1-641 ---------------MGNW-KNLVVVLLVSIGVGFGY---HTLIHA-SSSKANYSIAQKPTDPPKDKPINIVTHDGKTYCYSPVFSKGEGYVWIEKCGDN-TAKAR
EMY24429.1iAUS_s200703203/1-641 ---------------MGNW-KNLVVVLLVSIGIGFGY---HTLIHA-SSSKANYSIAQKPTDPPKDKPINIVTHDGKTYCYSPVFSKGEGYVWIEKCGDN-TAKAR
EMF44680.1iLRA_sTE1992/1-641 ---------------MGNW-KNLVVVLLVSIGVGFGY---HTLIHA-SSSKANYSIAQKPTDPPKDKPINIVTHDGKTYCYSPVFSKGEGYVWIEKCGDN-TAKAR
COP.iSK1_2829322/1-641 ---------------MGNW-KNLVVVLLVSIGVGFGY---HTLIHA-SSSKANYSIAQKPTDPPKDKPINIVTHDGKTYCYSPVFSKGEGYVWIEKCGDN-TAKAR
COP.iL1130_Q72PX8.cl2829/1-641 ---------------MGNW-KNLVVVLLVSIGVGFGY---HTLIHA-SSSKANYSIAQKPTDPPKDKPINIVTHDGKTYCYSPVFSKGEGYVWIEKCGDN-TAKAR
COP.iFDAARGOS203_838638/1-641 ---------------MGNW-KNLVVVLLVSIGVGFGY---HTLIHA-SSSKANYSIAQKPTDPPKDKPINIVTHDGKTYCYSPVFSKGEGYVWIEKCGDN-TAKAR
BVA.iPigK151_2924032/1-641 ---------------MGNW-KNLVVVLLVSIGVGFGY---HTLIHA-SSSKANYSIAQKPTDPPKDKPINIVTHDGKTYCYSPVFSKGEGYVWIEKCGDN-TAKAR
BAE.iS1548_1510806/1-641 ---------------MGNW-KNLVVVLLVSIGVGFGY---HTLIHA-SSSKANYSIAQKPTDPPKDKPINIVTHDGKTYCYSPVFSKGEGYVWIEKCGDN-TAKAR
BAE.iS1489_2915957/1-641 ---------------MGNW-KNLVVVLLVSIGVGFGY---HTLIHA-SSSKANYSIAQKPTDPPKDKPINIVTHDGKTYCYSPVFSKGEGYVWIEKCGDN-TAKAR
AW3Q16_AW3.1880_AG10.iAW3/1-641 ---------------MGNW-KNLVVVLLVSIGVGFGY---HTLIHA-SSSKANYSIAQKPTDPPKDKPINIVTHDGKTYCYSPVFSKGEGYVWIEKCGDN-TAKAR
AW1Q18_AW1.2737_AG3.iAW1-/1-641 ---------------MGNW-KNLVVVLLVSIGVGFGY---HTLIHA-SSSKANYSIAQKPTDPPKDKPINIVTHDGKTYCYSPVFSKGEGYVWIEKCGDN-TAKAR
AP7Q18_AP7.2705_AG3.iAP7-/1-641 ---------------MGNW-KNLVVVLLVSIGVGFGY---HTLIHA-SSSKANYSIAQKPTDPPKDKPINIVTHDGKTYCYSPVFSKGEGYVWIEKCGDN-TAKAR
AP6Q16_AP6.1585_AG3.iAP6-/1-641 ---------------MGNW-KNLVVVLLVSIGVGFGY---HTLIHA-SSSKANYSIAQKPTDPPKDKPINIVTHDGKTYCYSPVFSKGEGYVWIEKCGDN-TAKAR
AP5Q16_AP5.1533_AG10.iAP5/1-641 ---------------MGNW-KNLVVVLLVSIGVGFGY---HTLIHA-SSSKANYSIAQKPTDPPKDKPINIVTHDGKTYCYSPVFSKGEGYVWIEKCGDN-TAKAR
AP3Q18_AP3.2803_AG10.iAP3/1-641 ---------------MGNW-KNLVVVLLVSIGVGFGY---HTLIHA-SSSKANYSIAQKPTDPPKDKPINIVTHDGKTYCYSPVFSKGEGYVWIEKCGDN-TAKAR
AP1Q17_AP1.3257_AG3.iAP1-/1-641 ---------------MGNW-KNLVVVLLVSIGVGFGY---HTLIHA-SSSKANYSIAQKPTDPPKDKPINIVTHDGKTYCYSPVFSKGEGYVWIEKCGDN-TAKAR
AG3Q18_U1.2803_AG3.iO7371/1-641 ---------------MGNW-KNLVVVLLVSIGVGFGY---HTLIHA-SSSKANYSIAQKPTDPPKDKPINIVTHDGKTYCYSPVFSKGEGYVWIEKCGDN-TAKAR
AAS70908.1iCOP_sL1130/1-641 ---------------MGNW-KNLVVVLLVSIGVGFGY---HTLIHA-SSSKANYSIAQKPTDPPKDKPINIVTHDGKTYCYSPVFSKGEGYVWIEKCGDN-TAKAR
WP.061272590.1iUNK/1-641 ---------------MGNW-KNLVVVLLVSIGVGFGY---HTLIHA-SSSKANYSIAQKPTDPPKDKPINIVTHDGKTYCYSPVFSKGEGYVWIEKCGDN-IAKAR
KG1Q19_KG1.4080_AG14.uKG1/1-541 ---------------MGNW-KNLVVVLLVSIGVGFGY---HTLIHA-SSSKANYSIAQKPTDPPKDKPINIVTHDGKTYCYSPVFSKGEGYVWIEKCGDN-TAKAR
ICT.iPiscina_2787822/1-641 ---------------MGNW-KNLVVVLLVSIGVGFGY---HTLIHA-SSSKANYSIAQKPTDPPKDKPINIVTHDGKTYCYSPVFSKGEGYVWIEKCGDN-TAKAR
APH42155.1iICT/1-641 ---------------MGNW-KNLVVVLLVSIGVGFGY---HTLIHA-SSSKANYSIAQKPTDPPKDKPINIVTHDGKTYCYSPVFSKGEGYVWIEKCGDN-TAKAR
WP.025185840.1iUNK/1-641 ---------------MGNW-KNLVVVLLVSIGVGFGY---HTLIHA-SSSKANYSIAQKPTDPPKDKPINIVTHDGKTYCYSPVFSKGEGYVWIEKCGDN-TAKAR
WP.082271907.1iUNK/1-641 ---------------MGNW-KNLVVVLLVSIGVGFGY---HTLVHA-SSSKANYSIVQKPTDPPKDKPINIIAFDGKKYCYSPVFTKGEGYVWIDNCGDK-TAKAR
WP.061230617.1iUNK/1-641 ---------------MGNW-KNLVVVLLVSIGVGFGY---HTLVHA-SSSKANYSIVQKPTDPPKDKPINIVTHDGKKYCYSPVFTKGEGYVWIDNCGDK-TAKAR
WP.002072005.1iUNK/1-641 ---------------MGNW-KNLVVVLLVSIGVGFGY---HTLVHA-SSSKANYSIVQKPTDPPKDKPINIIAFDGKKYCYSPVFTKGEGYVWIDNCGDK-TAKAR
WP.000528834.1iUNK/1-641 ---------------MGNW-KNLVVVLLVSIGVGFGY---HTLVHA-SSSKANYSIVQKPADPPKDKPINIIAFDGKKYCYSPVFTKGEGYVWIDNCGDK-TAKAR
PN2Q18_PN2.3725_AG14.iPN2/1-641 ---------------MGNW-KNLVVVLLVSIGVGFGY---HTLVHA-SSSKANYSIVQKPTDPPKDKPINIIAFDGKKYCYSPVFTKGEGYVWIDNCGDK-TAKAR
CLA.iS611_1431139/1-641 ---------------MGNW-KNLVVVLLVSIGVGFGY---HTLVHA-SSSKANYSIVQKPTDPPKDKPINIIAFDGKKYCYSPVFTKGEGYVWIDNCGDK-TAKAR
CLA.iS114_3544770/1-641 ---------------MGNW-KNLVVVLLVSIGVGFGY---HTLVHA-SSSKANYSIVQKPTDPPKDKPINIIAFDGKKYCYSPVFTKGEGYVWIDNCGDK-TAKAR
CLA.iRUFN_678581/1-641 ---------------MGNW-KNLVVVLLVSIGVGFGY---HTLVHA-SSSKANYSIVQKPTDPPKDKPINIIAFDGKKYCYSPVFTKGEGYVWIDNCGDK-TAKAR
CLA.iLJ178_1435770/1-641 ---------------MGNW-KNLVVVLLVSIGVGFGY---HTLVHA-SSSKANYSIVQKPTDPPKDKPINIIAFDGKKYCYSPVFTKGEGYVWIDNCGDK-TAKAR
WP.061270220.1iUNK/1-641 ---------------MGNW-KNLVVVLLVSIGVGFGY---HTLIHA-SSSKANYSIAQKPTDPPKDKPINIVTHDGKTYCYSPVFSKGEGYVWIEKCGDN-IAKAR
WP.025183115.1iUNK/1-635 ---------------MGRW-IVLRVSLLVLIGIGFEYGINHTSINA--SSKSDYSIAQKPADQPKDKSIQVVMHGGSNYCYSPVFTKGEGYIWIDYCSDN-TAKAR
```

WP.017860601.1iUNK/1-635	---------------MGRW-IVLRVSLLVLIGIGFEYGINHTSINA--SSKSDYSIAQKPADQPKDKSIQVVMHGGSNYCYSPVFTKGEGYIWIDYCSDN-TAKAR
WP.017858080.1iUNK/1-635	---------------MGRW-IVLRVSLLVLIGIGFEYGINHTSINA--SSKSDYSIAQKPADQPKDKSIQVVMHGGSKYCYSPVFTKGEGYIWIDYCRDN-TAKAR
WP.017854665.1iUNK/1-635	---------------MGRW-IVLRVSLLVLIGIGFEYGINHTSINA--SSKSDYSIAQKPADQPKDKSIQVVMHGGSNYCYSPVFTKGEGYIWIDYCSDN-TAKAR
WP.017862508.1iUNK/1-635	---------------MGRW-IVLRVSLLVLIGIGFEYGINHTSINA--SSKSDYSIAQKPADQPKDKSIQVVMHGGSNYCYSPVFTKGEGYIWIDYCSDN-TAKAR
WP.137108220.1iUNK/1-640	---------------MGNW-KNLVVVLLVSIGVGFGY---HTLIHA-SSSKANYSIAQKPTDPPKDKPINIVTHNGKTYCYSPVFSKGEGYVWIEKCGDN-TAKAR
RT2Q18_RT2.2771_AG3.iRT2-/1-640	---------------MGNW-KNLVVVLLVSIGVGFGY---HTLIHA-SSSKANYSIAQKPTDPPKDKPINIVTHNGKTYCYSPVFSKGEGYVWIEKCGDN-TAKAR
KW2Q18_KW2.2740_AG3.iKW2-/1-640	---------------MGNW-KNLVVVLLVSIGVGFGY---HTLIHA-SSSKANYSIAQKPTDPPKDKPINIVTHNGKTYCYSPVFSKGEGYVWIEKCGDN-TAKAR
KW2Q18_KW2.2724_AG3.iKW2-/1-640	---------------MGNW-KNLVVVLLVSIGVGFGY---HTLIHA-SSSKANYSIAQKPTDPPKDKPINIVTHNGKTYCYSPVFSKGEGYVWIEKCGDN-TAKAR
KW1Q16_KW1.1409_AG3.iKW1-/1-640	---------------MGNW-KNLVVVLLVSIGVGFGY---HTLIHA-SSSKANYSIAQKPTDPPKDKPINIVTHNGKTYCYSPVFSKGEGYVWIEKCGDN-TAKAR
WP.002159113.1iUNK/1-640	---------------MGNW-KNLVVVLLVSIGVGFGY---HTLIHA-SSSKANYSIAQKPTDPPKDKPINIVTHDGKKYCYSPVFSKGEGYVWIEKCGDN-TAKAR
WP.000528833.1iUNK/1-640	---------------MGNW-KNLVVVLLVSIGVGFGY---HTLVHA-SSSKANYSIAQKPTDPPKDKPINIVTHDGKKYCYSPVFSKGEGYVWIEKCGDN-TAKAR
MAE.iUPMMCNIIDLP_2772434/1-640	---------------MGNW-KNLVVVLLVSIGVGFGY---HTLIHA-SSSKANYSIAQKPTDPPKDKPINIVTHDGKKYCYSPVFSKGEGYVWIEKCGDN-TAKAR
MAE.iUPMMCNIIDHP_2772435-/1-640	---------------MGNW-KNLVVVLLVSIGVGFGY---HTLIHA-SSSKANYSIAQKPTDPPKDKPINIVTHDGKKYCYSPVFSKGEGYVWIEKCGDN-TAKAR
WP.061241270.1iUNK/1-640	---------------MGNW-KNLVVVLLVSIGVGFGY---HTLIHA-SSSKANYSIAQKPTDPPKDKPINIVTHDGKTYCYSPVFSKGEGYVWIEKCGDN-TAKAR
EMN50459.1iUNK_sL1207/1-640	---------------MGNW-KNLVVVLLVSIGVGFGY---HTLIHA-SASKANYSIAQKPTDPPKDKPINIVTHDGKTYCYSPVFSKGEGYVWIEKCGDN-TAKAR
WP.020766753.1mUNK/1-643	---------------MGNW-KNFVVVLLVSIGVGFGYGMDHTLVYA-SSSETNYSIFQKPTDPPKDKPINIVAHDGKTYCYSPVFSKGEGYVWIEKCGDK-TAKAR
WP.004768396.1kUNK/1-643	---------------MGNW-KNLVVVLLVSIGVGFGYGMDHTLVYA-SSSETNYSIFQKPTDPPKDKPINIVAHDGKTYCYSPVFSKGEGYVWIEKCGDK-TAKAR
WP.016751772.1kUNK/1-642	---------------MGRW-IALKVVLLVLIGIGFEYGINHTSIDA--SSKSGYSIAQKPTDQPKDKPINIVTHDGKTYCYSPVFSKGEGYVWINKCGDK-TAKAR
WP.078131700.1kUNK/1-642	---------------MGRW-IALKVVLLVLIGIGFEYGINHTSIDA--SSKSGYSIAQKPTDQPKDKPINIVTHDGKTYCYSPVFTKGEGYVWINKCEDK-TAKAR
WP.004782942.1kUNK/1-642	---------------MGRW-IALKVVLLVLIGIGFEYGINHTSIDA--SSKSGYSIAQKPTDQPKDKPINIVTHDGKTYCYSPVFTKGEGYVWIEKCGDK-TAKAR
WP.004771056.1kUNK/1-642	---------------MGRW-IALKVVLLVLIGIGFEYGINHTSIDA--SSKSGYSIAQKPTDQPKDKPINIVTHDGKTYCYSPVFSKGEGYVWINKCGDK-TAKAR
WP.004762947.1kUNK/1-642	---------------MGRW-IALKVVLLVLIGIGFEYGINHTSIDA--SSKSGYSIAQKPTDQPKDKPINIVTHDGKTYCYSPVFTKGEGYVWIEKCGDK-TAKAR
WP.004776693.1kUNK/1-642	---------------MGRW-IALKVVLLVLIGIGFEYGINHTSIDA--SSKSGYSIAQKPTDQPKDKPINIVTHDGKTYCYSPVFTKGEGYVWIEKCGDK-TAKAR
WP.016753063.1kUNK/1-642	---------------MGRW-IALKVVLLVLIGIGFEYGINHTSIDA--SSKSGYSIAQKPTDQPKDKPINIVTHDGKTYCYSPVFTKGEGYVWINKCEDK-TAKAR
WP.061248835.1nUNK/1-642	---------------MGRW-IALKVVLLVLIGIGFEYGINHTSIDA--SSKSGYSIAQKPTDQPKDKPIRIIGYGDEKYCYSPVFTKGEGYVWIDKCEDK-TAKAR
WP.004452138.1nUNK/1-642	---------------MGRW-IALKVVLLVLIGIGFEYGINHTSIDA--SSKSDYSIAQKPTDQPKDKPIRIIGYGDEKYCYSPVFTKGEGYVWIDKCEDK-TAKAR
WP.061233679.1nUNK/1-642	---------------MGRC-IALKVVLLVLIGIGFEYGINHTSIDA--SSKSGYSIAQKPTDQPKDKPIRIIGYGDEKYCYSPVFTKGEGYVWIDKCEDK-TAKAR
WP.053522773.1nUNK/1-642	---------------MGRW-IALKVVLLVLIGIGFEYGINHTSIDA--SSKSGYSIAQKPTDQPKDKPIRIIGYGDEKYCYSPVFTKGEGYVWIDKCEDK-TAKAR
EMO24871.1nBAE_sHAI135/1-642	---------------MGRW-IALKVVLLVLIGIGFEYGINHTSIDA--SSKSGYSIAQKPTDQPKDKPIRIIGYGDEKYCYSPVFTKGEGYVWIDKCEDK-TAKAR
WP.061245737.1nUNK/1-642	---------------MGRW-IALKVVLLVLIGIGFEYGINHTSIDA--SSKSGYSIAQKPTDQPKDKPIRIIGYGDEKYCYSPVFTKGEGYVWIDKCEDK-TAKAR
WP.004433931.1nUNK/1-642	---------------MGRW-IALKVVLLVLIGIGFEYGINHTSIDA--SSKSGYSIAQKPTDQPKDKPIRIIGYGDEKYCYSPVFTKGEGYVWIDKCEDK-TAKAR
WP.002177372.1nUNK/1-643	---------------MGRW-IALKVVLLVLIGIGFEYGINHTSVDA--SSKSAYSIAQKPTDQPKDKPISIIGYGEEKYCYSPVFTKGEGYVWIDKCGDK-TAKAR
UNK.sU233_1198243/1-639	---------------MGRW-SVLIIVLLVSSGVGFAYGGNSQIVSS--------SIVQKPTDQPKDQPIKVVLHTGKEYCYSPVFSKGEGYVWIDKCGNQ-TAKAR
UNK.sU1604_2753918/1-639	---------------MGRW-SVLIIVLLVSSGVGFAYGGNSQIIS--------SSIVQKPTDQPKDQPIKVVLHTGKEYCYSPVFSKGEGYVWIDKCGNQ-TAKAR
UNK.sAH2_650502/1-641	---------------MGRW-SVLIIVLLVSSGVGFAYGGNSQIVSS--------SIVQKPTDQPKDQPIKVVLHTGKEYCYSPVFSKGEGYVWIDKCGNQ-TAKAR
SNI.sLT821_2460912/1-641	---------------MGRW-SVLIIVLLVLSGVGFAYGGNSQIVSS--------SIVQKPTDQPKDQPIKVVLHTGKEYCYSPVFSKGEGYVWIDKCGNQ-TAKAR
UNK.sU160_420515/1-639	---------------MGRW-SVLIIVLLVSSGVGFAYGGNSQIVSS--------SIVQKPTDQPKDQPIKVVLHTGKEYCYSPVFSKGEGYVWIDKCGNK-TAKAR
WP.021064468.1aUNK/1-638	---------------MGRW-IVLLVFLSVSVGVGFTYGVSPAIIDL-LSKVSPSSIVQKPTDKPKDKPIKVVVHDGGKFCYAPTFSGGESYIQLRQCWERGVTNAR
WP.194099171.1kUNK/1-637	---------------MYNWKKILIVILLTSIMVYLEYEMDHTLVHA-ASSKATGSIVQKPTNPPKDKPIKVNVSGGGTFCYGPNFSGGESYIVIEQCWQMHVMNAR
WP.025182393.1kUNK/1-637	---------------MYNWKKILIVILLTSIMVYLEYEMDHTLVHA-ASSKATGSIVQKPTNPPKDKPIKVNVSGGGTFCYGPNFSGGESYIVIEQCWQMHVMNAR
EMJ87808.1kUNK_sJB/1-614	--------------------------------------MDHTLVHA-ASSKATGSIVQKPTNPPKDKPIKVNVSGGGTFCYGPNFSGGESYIVIEQCWQMHVMNAR
WP.020765622.1mUNK/1-637	---------------MYNWKKILIVILLTSIMVYLEYEMDHTLVHA-ASSKATGSIVQKPTDPPKDKPIKVNVSGGGTFCYGPNFSGGESYIVIEQCWQMHVMNAR
WP.020767664.1kUNK/1-635	---------------MGRW-IVLRVFLLVLIGIGFEYGINHTSINA--SSKSDYSIVQKPTDQPKDKPVQVVVSDRGKFCYGPTFSGGESYIVIEQCWQMHVMSAR
EMN23823.1kSNE_sRM1/1-637	---------------MYNWKKILIVILLTSIMVYLEYEMDHTLVHA-AASKATGSIVQKPTDPPKDKPIKVNVSGGGTFCYGPTFSGGESYILIEQCWQMHVMNAR
EKQ83184.1iGSA_sMoskva/1-637	---------------MYNWKKILIVILLTSIMVYLEYEMDHTLVHA-AASKATGSIVQKPTDPPKDKPIKVNVSGGGTFCYGPTFSGGESYILIEQCWQMHVMNAR
EJO70052.1kGSA_sRM52/1-637	---------------MYNWKKILIVILLTSIMVYLEYEMDHTLVHA-AASKATGSIVQKPTDPPKDKPIKVNVSGGGTFCYGPTFSGGESYILIEQCWQMHVMNAR
EMO79484.1kUNK_s200801774/1-637	---------------MYNWKKILIVILLTSIMVYLEYEMDHTLVHA-AASKATGSIVQKPTDPPKDKPIKVNVSGGGTFCYGPTFSGGESYIVIEQCWQMHVMNAR
EMN06418.1kBIM_s1051/1-637	---------------MYNWKKILIVILLTSIMVYLEYEMDHTLVHA-AASKATGSIVQKPTDPPKDKPIKVNVSGGGTFCYGPTFSGGESYIVIEQCWQMHVMNAR
EKO51942.1kUNK_s200802841/1-637	---------------MYNWKKILIVILLTSIMVYLEYEMDHTLVHA-AASKATGSIVQKPTDPPKDKPIKVNVSGGGTFCYGPTFSGGESYIVIEQCWQMHVKNAR
WP.004769005.1kUNK/1-637	---------------MYNWKKILIVILLTSIMVYLEYEMNHTLVHA-AASKATGSIVQKPTDPPKDKPIKVNVSGGGTFCYGPTFSGGESYIVIEQCWQMHVKNAR
PN5K14_PN5.1932_AG15.kPN5/1-637	---------------MYNWKKILIVILLTSIMVYLEYEMNHTLVHA-AASKATGSIVQKPTDPPKDKPIKVNVSDGGKFCYGPIFSGGESYIIIEQCWERHVKNAR
EKO13416.1kUNK_sH1/1-637	---------------MYNWKKILIVILLTSIMVYLEYEMNHTLVHA-AASKATGSIVQKPTDPPKDKPIKVNVSDGGKFCYGPIFSGGESYIIIEQCWERHVKNAR
WP.192503323.1iUNK/1-638	---------------MYNWKKILIVVLLASIMVYLEYEMDHTLVHAASSSKTTNSIVQKPTDPPKDKPIKVNVSGGGTFCYGPNFSGGESYIIIEQCWQMHVMNAR
WP.096637690.1iUNK/1-638	---------------MYNWKKILIVVLLASIMVYLEYEMDHTLVHAASSSKTANSIVQKPTDPPKDKPIKVNVSGGGTFCYGPNFSGGESYIIIEQCWQMHVTNAR
WP.082272092.1iUNK/1-624	---------------m-------------SFGVGYSYGVNPDVVPI-SSSDIPGSIIQRPTDKPKDKPIKVNVSGGGTFCYGPNFSGGESYIIIEQCWQMHVMNAR
WP.061286898.1iUNK/1-638	---------------MYNWKKILIVVLLASIMVYLEYEMDHTLVHAASSSKTTNSIVQKPTDPPKDKPIKVNVSGGGTFCYGPNFSGGESYIIIEQCWQMHVTNAR
WP.025185094.1iUNK/1-638	---------------MYNWKKILIVVLLASIMVYLEYEMDHTLVHAASSSKTANSIVQKPTDPPKDKPIKVNVSGGGTFCYGPNFSGGESYIIIEQCWQMHVTNAR
WP.025180333.1iUNK/1-638	---------------MYNWKKILIVVLLASIMVYLEYEMDHTLVHAASSSKTTNSIVQKPTDPPKDKPIKVNVSGGGTFCYGPDFSGGESYIIIEQCWQMHVMNAR
WP.025176823.1iUNK/1-636	---------------MCNWKKIFIVVLLVLIGFGFEYGINPTPVDA--SSKIEYLVIQKPTDPPKDQPIKVIVSGEGKFCYGPDFSGGESYIIIEQCWQMHVMNAR
WP.017861903.1iUNK/1-635	---------------MRHS-IVFIVVLLVLVTTSFEYSINNIHIHA--SSNTSNSIIQKPTDQPKDKPIKIVIHDGGKFCYGPFVSGGESYIIIEQCWQMHVMNAR
WP.017861199.1iUNK/1-638	---------------MYNWKKILIVVLLASIMVYLEYEMDHTLVHAASSSKTTNSIVQKPTDPPKDKPIKVNVSGGGTFCYGPNFSGGESYIIIEQCWQMHVMNAR
WP.017854888.1iUNK/1-638	---------------MYNWKKILIVVLLASIMVYLEYEMDHTLVHAASSSKTTNSIVQKPTDPPKDKPIKVNVSGGGTFCYGPNFSGGESYIVIEQCWQMHVMNAR
WP.017854782.1iUNK/1-638	---------------MYNWKKILIVVLLASIMVYLEYEMDHTLVHAASSSKTTNSIVQKPTDPPKDKPIKVNVSGGGTFCYGPNFSGGESYIIIEQCWQMHVMNAR
WP.017853778.1iUNK/1-638	---------------MYNWKKILIVVLLASIMVYLEYEMDHTLVHAASSSKTTNSIVQKPTDPPKDKPIKVNVSGGGTFCYGPDFSGGESYIIIEQCWQMHVMNAR
WP.000283007.1iUNK/1-638	---------------MYNWKKILIVVLLASIMVYLEYEMDHTLVHAASSSKTTNSIVQKPTDPPKDKPIKVNVSGGGTFCYGPNFSGGESYIIIEQCWQMHVMNAR
WP.000283005.1iUNK/1-638	---------------MYNWKKILIVVLLASIMVYLEYEMDHTLVHAASSSKTTNSIVQKPTDPPKDKPIKVNVSGGGTFCYGPNFSGGESYIIIEQCWQMHVMNAR
WP.000283003.1iUNK/1-638	---------------MYNWKKILIVVLLASIMVYLEYEMDHTLVHAASSSKTTNSIVQKPTDPPKDKPIKVNVSGGGTFCYGPNFSGGESYIIIEQCWQMHVMNAR
WP.000283002.1iUNK/1-638	---------------MYNWKKILIVVLLASIMVYLEYEMDHTLVHAASSSKTTNSIVQKPTDPPKDKPIKVNVSGGGTFCYGPNFSGGESYIIIEQCWQMHVMNAR
WP.000283000.1iUNK/1-638	---------------MYNWKKILIVVLLASIMVYLEYEMDHTLVHAASSSKTTNSIVQKPTDPPKDKPIKVNVSGGGTFCYGPNFSGGESYIIIEQCWQMHVMNAR
WP.000282990.1iUNK/1-638	---------------MYNWKKILIVVLLASIMVYLEYEMDHTLVHAASSSKTTNSIVQKPTDPPKDKPIKVNVSGGGTFCYGPDFSGGESYIIIEQCWQMHVMNAR
WP.000282989.1iUNK/1-638	---------------MYNWKKILIVVLLASIMVYLEYEMDHTLVHAASSSKTTNSIVQKPTDPPKDKPIKVNVSGGGTFCYGPDFSGGESYIIIEQCWQMHVMNAR
WP.000282982.1iUNK/1-638	---------------MYNWKKILIVVLLASIMVYLEYEMDHTLVHAASSSKTANSIVQKPTDPPKDKPIKVNVSGGGTFCYGPNFSGGESYIIIEQCWQMHVTNAR
UNK.iRCA_1249851/1-638	---------------MYNWKKILIVVLLASIMVYLEYEMDHTLVHAASSSKTTNSIVQKPTDPPKDKPIKVNVSGGGTFCYGPNFSGGESYIIIEQCWQMHVMNAR
UNK.iR7_771161/1-638	---------------MYNWKKILIVVLLASIMVYLEYEMDHTLVHAASSSKTTNSIVQKPTDPPKDKPIKVNVSGGGTFCYGPNFSGGESYIIIEQCWQMHVMNAR
UNK.iR22_779356/1-638	---------------MYNWKKILIVVLLASIMVYLEYEMDHTLVHAASSSKTTNSIVQKPTDPPKDKPIKVNVSGGGTFCYGPNFSGGESYIIIEQCWQMHVMNAR
UNK.iR21_780144/1-638	---------------MYNWKKILIVVLLASIMVYLEYEMDHTLVHAASSSKTTNSIVQKPTDPPKDKPIKVNVSGGGTFCYGPNFSGGESYIIIEQCWQMHVMNAR
UNK.iR19_780726/1-638	---------------MYNWKKILIVVLLASIMVYLEYEMDHTLVHAASSSKTTNSIVQKPTDPPKDKPIKVNVSGGGTFCYGPNFSGGESYIIIEQCWQMHVMNAR
UNK.iR17_779148/1-638	---------------MYNWKKILIVVLLASIMVYLEYEMDHTLVHAASSSKTTNSIVQKPTDPPKDKPIKVNVSGGGTFCYGPNFSGGESYIIIEQCWQMHVMNAR
UNK.iR16_770337/1-638	---------------MYNWKKILIVVLLASIMVYLEYEMDHTLVHAASSSKTTNSIVQKPTDPPKDKPIKVNVSGGGTFCYGPNFSGGESYIIIEQCWQMHVMNAR
UNK.iR13_770337/1-638	---------------MYNWKKILIVVLLASIMVYLEYEMDHTLVHAASSSKTTNSIVQKPTDPPKDKPIKVNVSGGGTFCYGPNFSGGESYIIIEQCWQMHVMNAR
UNK.iR13L_770735/1-638	---------------MYNWKKILIVVLLASIMVYLEYEMDHTLVHAASSSKTTNSIVQKPTDPPKDKPIKVNVSGGGTFCYGPDFSGGESYIIIEQCWQMHVMNAR
UNK.iR12_770911/1-638	---------------MYNWKKILIVVLLASIMVYLEYEMDHTLVHAASSSKTTNSIVQKPTDPPKDKPIKVNVSGGGTFCYGPNFSGGESYIIIEQCWQMHVMNAR
UNK.iR11_769408/1-638	---------------MYNWKKILIVVLLASIMVYLEYEMDHTLVHAASSSKTTNSIVQKPTDPPKDKPIKVNVSGGGTFCYGPNFSGGESYIIIEQCWQMHVMNAR
RT2Q1D_RT2.3449_AG3.iRT2-/1-638	---------------MYNWKKILIVVLLASIMVYLEYEMDHTLVHAASSSKTTNSIVQKPTDPPKDKPIKVNVSGGGTFCYGPNFSGGESYIIIEQCWQMHVMNAR
PN3Q1D_SLPN3.3479_AG3.iPN/1-638	---------------MYNWKKILIVVLLASIMVYLEYEMDHTLVHAASSSKTTNSIVQKPTDPPKDKPIKVNVSGGGTFCYGPNFSGGESYIIIEQCWQMHVMNAR
PN2Q1D_PN2.4411_AG14.iPN2/1-638	---------------MYNWKKILIVVLLASIMVYLEYEMDHTLVHAASSSKTANSIVQKPTDPPKDKPIKVNVSGGGTFCYGPNFSGGESYIIIEQCWQMHVMNAR
PD1Q11_PD1.1258_AG3.iPD1-/1-638	---------------MYNWKKILIVVLLASIMVYLEYEMDHTLVHAASSSKTTNSIVQKPTDPPKDKPIKVNVSGGGTFCYGPDFSGGESYIIIEQCWQMHVMNAR
LAI.iIPAV_3526676/1-638	---------------MYNWKKILIVVLLASIMVYLEYEMDHTLVHAASSSKTTNSIVQKPTDPPKDKPIKVNVSGGGTFCYGPDFSGGESYIIIEQCWQMHVMNAR
KW2Q1D_KW2.3409_AG3.iKW2-/1-638	---------------MYNWKKILIVVLLASIMVYLEYEMDHTLVHAASSSKTTNSIVQKPTDPPKDKPIKVNVSGGGTFCYGPNFSGGESYIIIEQCWQMHVMNAR
KW2Q1D_KW2.3389_AG3.iKW2-/1-638	---------------MYNWKKILIVVLLASIMVYLEYEMDHTLVHAASSSKTTNSIVQKPTDPPKDKPIKVNVSGGGTFCYGPNFSGGESYIIIEQCWQMHVMNAR
KW1Q11_KW1.750_AG3.iKW1-1/1-638	---------------MYNWKKILIVVLLASIMVYLEYEMDHTLVHAASSSKTTNSIVQKPTDPPKDKPIKVNVSGGGTFCYGPNFSGGESYIIIEQCWQMHVMNAR
KG2Q1B_KG2.4879_AG3.uKG2-/1-638	---------------MYNWKKILIVVLLASIMVYLEYEMDHTLVHAASSSKTTNSIVQKPTDPPKDKPIKVNVSGGGTFCYGPDFSGGESYIIIEQCWQMHVMNAR
ICT.iS898_780339/1-638	---------------MYNWKKILIVVLLASIMVYLEYEMDHTLVHAASSSKTTNSIVQKPTDPPKDKPIKVNVSGGGTFCYGPNFSGGESYIIIEQCWQMHVMNAR
ICT.iPiscina_793266/1-638	---------------MYNWKKILIVVLLASIMVYLEYEMDHTLVHAASSSKTTNSIVQKPTDPPKDKPIKVNVSGGGTFCYGPNFSGGESYIIIEQCWQMHVMNAR
ICT.iLangkawi_792609/1-638	---------------MYNWKKILIVVLLASIMVYLEYEMDHTLVHAASSSKTTNSIVQKPTDPPKDKPIKVNVSGGGTFCYGPNFSGGESYIIIEQCWQMHVMNAR
HJO.iNorma_645204/1-638	---------------MYNWKKILIVVLLASIMVYLEYEMDHTLVHAASSSKTTNSIVQKPTDPPKDKPIKVNVSGGGTFCYGPNFSGGESYIIIEQCWQMHVMNAR
HJO.iL53_641621/1-638	---------------MYNWKKILIVVLLASIMVYLEYEMDHTLVHAASSSKTTNSIVQKPTDPPKDKPIKVNVSGGGTFCYGPDFSGGESYIIIEQCWQMHVMNAR
HJO.iHjtno_776148/1-638	---------------MYNWKKILIVVLLASIMVYLEYEMDHTLVHAASSSKTTNSIVQKPTDPPKDKPIKVNVSGGGTFCYGPDFSGGESYIIIEQCWQMHVMNAR
EMN70415.1iBAE_sUI08561/1-615	--------------------------------------MDHTLVHAASSSKTTNSIVQKPTDPPKDKPIKVNVSGGGTFCYGPNFSGGESYIIIEQCWQMHVMNAR
EMN55391.1iAIS_sLP101/1-615	--------------------------------------MDHTLVHAASSSKTANSIVQKPTDPPKDKPIKVNVSGGGTFCYGPNFSGGESYIIIEQCWQMHVTNAR
EKR26445.1iBAE_sL1111/1-615	--------------------------------------MDHTLVHAASSSKTTNSIVQKPTDPPKDKPIKVNVSGGGTFCYGPNFSGGESYIIIEQCWQMHVMNAR
EKR19078.1iPES_s200600696/1-615	--------------------------------------MDHTLVHAASSSKTTNSIVQKPTDPPKDKPIKVNVSGGGTFCYGPDFSGGESYIIIEQCWQMHVMNAR
EKO89603.1iGSA_sAndaman/1-615	--------------------------------------MDHTLVHAASSSKTTNSIVQKPTDPPKDKPIKVNVSGGGTFCYGPNFSGGESYIIIEQCWQMHVMNAR
EKN86468.1iUNK_s200200062/1-615	--------------------------------------MDHTLVHAASSSKTTNSIVQKPTDPPKDKPIKVNVSGGGTFCYGPNFSGGESYIIIEQCWQMHVMNAR
EJP18090.1iUNK_sFPW2026/1-615	--------------------------------------MDHTLVHAASSSKTANSIVQKPTDPPKDKPIKVNVSGGGTFCYGPNFSGGESYIIIEQCWQMHVTNAR
[illegible]	[illegible]

EJP03263.1iBRA_sMallika/1-615	--------------------------------------MDHTLVHAASSSKTTNSIVQKPTDPPKDKPIKVNVSGGGTFCYGPNFSGGESYIIIEQCWQMHVMNAR
COP.iSK1_780466/1-638	---------------MYNWKKILIVVLLASIMVYLEYEMDHTLVHAASSSKTTNSIVQKPTDPPKDKPIKVNVSGGGTFCYGPNFSGGESYIIIEQCWQMHVMNARY
COP.iL1130_Q72UL8.cl7806/1-638	---------------MYNWKKILIVVLLASIMVYLEYEMDHTLVHAASSSKTTNSIVQKPTDPPKDKPIKVNVSGGGTFCYGPNFSGGESYIIIEQCWQMHVMNARY
COP.iFDAARGOS203_3070416/1-638	---------------MYNWKKILIVVLLASIMVYLEYEMDHTLVHAASSSKTTNSIVQKPTDPPKDKPIKVNVSGGGTFCYGPNFSGGESYIIIEQCWQMHVMNARY
CLA.iS782_748545/1-638	---------------MYNWKKILIVVLLASIMVYLEYEMDHTLVHAASSSKTTNSIVQKPTDPPKDKPIKVNVSGGGTFCYGPNFSGGESYIIIEQCWQMHVMNARY
BVA.iPigK151_800903/1-638	---------------MYNWKKILIVVLLASIMVYLEYEMDHTLVHAASSSKTTNSIVQKPTDPPKDKPIKVNVSGGGTFCYGPNFSGGESYIIIEQCWQMHVMNARY
BAE.iS1548_835652/1-638	---------------MYNWKKILIVVLLASIMVYLEYEMDHTLVHAASSSKTTNSIVQKPTDPPKDKPIKVNVSGGGTFCYGPNFSGGESYIIIEQCWQMHVMNARY
BAE.iS1489_771332/1-638	---------------MYNWKKILIVVLLASIMVYLEYEMDHTLVHAASSSKTTNSIVQKPTDPPKDKPIKVNVSGGGTFCYGPNFSGGESYIIIEQCWQMHVMNARY
AW3Q11_AW3.3712_AG10.iAW3/1-638	---------------MYNWKKILIVVLLASIMVYLEYEMDHTLVHAASSSKTTNSIVQKPTDPPKDKPIKVNVSGGGTFCYGPDFSGGESYIIIEQCWQMHVMNARY
APH40614.1iICT_pA9P81.068/1-638	---------------MYNWKKILIVVLLASIMVYLEYEMDHTLVHAASSSKTTNSIVQKPTDPPKDKPIKVNVSGGGTFCYGPNFSGGESYIIIEQCWQMHVMNARY
AP7Q1D_AP7.3372_AG3.iAP7-/1-638	---------------MYNWKKILIVVLLASIMVYLEYEMDHTLVHAASSSKTTNSIVQKPTDPPKDKPIKVNVSGGGTFCYGPNFSGGESYIIIEQCWQMHVMNARY
AP6Q11_AP6.802_AG3.iAP6-1/1-638	---------------MYNWKKILIVVLLASIMVYLEYEMDHTLVHAASSSKTTNSIVQKPTDPPKDKPIKVNVSGGGTFCYGPDFSGGESYIIIEQCWQMHVMNARY
AP5Q11_AP5.748_AG10.iAP5-/1-638	---------------MYNWKKILIVVLLASIMVYLEYEMDHTLVHAASSSKTTNSIVQKPTDPPKDKPIKVNVSGGGTFCYGPDFSGGESYIIIEQCWQMHVMNARY
AP3Q1D_AP3.3479_AG10.iAP3/1-638	---------------MYNWKKILIVVLLASIMVYLEYEMDHTLVHAASSSKTTNSIVQKPTDPPKDKPIKVNVSGGGTFCYGPNFSGGESYIIIEQCWQMHVMNARY
AP1Q1C_AP1.3935_AG3.iAP1-/1-638	---------------MYNWKKILIVVLLASIMVYLEYEMDHTLVHAASSSKTTNSIVQKPTDPPKDKPIKVNVSGGGTFCYGPNFSGGESYIIIEQCWQMHVMNARY
ALE37874.iHJO_pG436.0655/1-615	--------------------------------------MDHTLVHAASSSKTTNSIVQKPTDPPKDKPIKVNVSGGGTFCYGPDFSGGESYIIIEQCWQMHVMNARY
AG3Q1D_U1.3479_AG3.iO7371/1-638	---------------MYNWKKILIVVLLASIMVYLEYEMDHTLVHAASSSKTTNSIVQKPTDPPKDKPIKVNVSGGGTFCYGPNFSGGESYIIIEQCWQMHVMNARY
AER03630.1iLAI_sIPAV/1-615	--------------------------------------MDHTLVHAASSSKTTNSIVQKPTDPPKDKPIKVNVSGGGTFCYGPDFSGGESYIIIEQCWQMHVMNARY
AAS69260.1iCOP_sL1130/1-640	-------------mKMYNWKKILIVVLLASIMVYLEYEMDHTLVHAASSSKTTNSIVQKPTDPPKDKPIKVNVSGGGTFCYGPNFSGGESYIIIEQCWQMHVMNARY
WP.000283006.1iUNK/1-638	---------------MYNWKKILIVVLLASIMVYLEYEMDHTLVHAASSSKTTNSIVQKPTDPPKDKPIKVNVSGGGTFCYGPNFSGGESYIIIEQCWQMHVMNARY
WP.000283001.1iUNK/1-638	---------------MYNWKKILIVVLLASIMVYLEYEMDHTLVHAASSSKTTNSIVQKPTDPPKDKPIKVNVSGGGTFCYGPNFSGGESYIIIEQCWQMHVMNARY
EMN81166.1iGSA_sUI12764/1-615	--------------------------------------MDHTLVHAASSSKTTNSIVQKPTDPPKDKPIKVNVSGGGTFCYGPNFSGGESYIIIEQCWQMHVMNARY
EKR56005.1iUNK_sUI12758/1-615	--------------------------------------MDHTLVHAASSSKTTNSIVQKPTDPPKDKPIKVNVSGGGTFCYGPNFSGGESYIIIEQCWQMHVMNARY
WP.000283004.1iUNK/1-638	---------------MYNWKKILIVVLLASIMVYLEYEMDHTLVHAASSSKTTNSIVQKPTDPPKDKPIKVNVSGGGTFCYGPNFSGGESYIIIEQCWQMHVMNARY
EMN49661.1iUNK_sL1207/1-615	--------------------------------------MDHTLVHAASSSKTTNSIVQKPTDPPKDKPIKVNASGGGTFCYGPNFSGGESYIIIEQCWQMHVMNARY
WP.061250510.1iUNK/1-638	---------------MYNWKKILIVVLLASIMVYLEYEMDHTLVHAASSSKTTNSIVQKPTDPPKDKPIKVNVSGGGTFCYGPNFSGGESYIIIEQCWQMHVTNARY
WP.000282984.1iUNK/1-638	---------------MYNWKKILIVVLLASIMVYLEYEMDHTLVHAASSSKTANSIVQKPTDPPKDKPIKVNVSGGGTFCYGPNFSGGESYIIIEQCWQMHVTNARY
WP.000283008.1iUNK/1-638	---------------MYNWKKILIVVLLASIMVYLEYEMDHTLVHAASSSKTTNSIVQKPTDPPKDKPIKVNVSGGGTFCYGPNFSGGESYIIIEQCWQMHVMNARY
WP.000282985.1iUNK/1-638	---------------MYNWKKILIVVLLASIMVYLEYEMDHTLVHAASSSKTANSIVQKPTDPPKDKPIKVNVSGGGTFCYGPNFSGGESYIIIEQCWQMHVTNARY
WP.000282983.1iUNK/1-638	---------------MYNWKKILIVVLLASIMVYLEYEMDHTLVHAASSSKTANSIVQKPTDPPKDKPIKVNVSGGGTFCYGPNFSGGESYIIIEQCWQMHVTNARY
WP.000282980.1iUNK/1-638	---------------MYNWKKILIVVLLASIMVYLEYEMDHTLVHAASSSKTANSIVQKPTDPPKDKPIKVNVSGGGTFCYGPDFSGGESYIIIEQCWQMHVMNARY
LHI.iS56609_771906/1-638	---------------MYNWKKILIVVLLASIMVYLEYEMDHTLVHAASSSKTANSIVQKPTDPPKDKPIKVNVSGGGTFCYGPNFSGGESYIIIEQCWQMHVTNARY
KG1Q14_KG1.1896_AG14.uKG1/1-638	---------------MYNWKKILIVVLLASIMVYLEYEMDHTLVHAASSSKTANSIVQKPTDPPKDKPIKVNVSGGGTFCYGPNFSGGESYIIIEQCWQMHVTNARY
EMJ65036.1iPNA_sCSL4002/1-615	--------------------------------------MDHTLVHAASSSKTANSIVQKPTDPPKDKPIKVNVSGGGTFCYGPDFSGGESYIIIEQCWQMHVMNARY
EKR45959.1iGSA_sUI08368/1-615	--------------------------------------MDHTLVHAASSSKTTNSIVQKPTDPPKDKPIKVNVSGGGTFCYGPNFSGGESYIIIEQCWQMHVMNARY
EKR38279.1iHIS_sR499/1-615	--------------------------------------MDHTLVHAASSSKTANSIVQKPTDPPKDKPIKVNVSGGGTFCYGPNFSGGESYIIIEQCWQMHVTNARY
EKO71150.1iCLA_sLV133/1-615	--------------------------------------MDHTLVHAASSSKTANSIVQKPTDPPKDKPIKVNVSGGGTFCYGPNFSGGESYIIIEQCWQMHVTNARY
CLA.iS611_779183/1-638	---------------MYNWKKILIVVLLASIMVYLEYEMDHTLVHAASSSKTANSIVQKPTDPPKDKPIKVNVSGGGTFCYGPNFSGGESYIIIEQCWQMHVTNARY
CLA.iS114_538868/1-638	---------------MYNWKKILIVVLLASIMVYLEYEMDHTLVHAASSSKTANSIVQKPTDPPKDKPIKVNVSGGGTFCYGPNFSGGESYIIIEQCWQMHVTNARY
CLA.iLJ178_784208/1-638	---------------MYNWKKILIVVLLASIMVYLEYEMDHTLVHAASSSKTANSIVQKPTDPPKDKPIKVNVSGGGTFCYGPNFSGGESYIIIEQCWQMHVTNARY
WP.033109409.1iUNK/1-638	---------------MYNWKKILIVVLLASIMVYLEYEMDHTLVHAASSSKTANSIVQKPTDPPKDKPIKVIVSGEGKFCYGPNFSGGESYIIIEQCWQMHVMNARY
WP.000282975.1iUNK/1-638	---------------MYNWKKIFIVVLLASIMVYLEYEMDHTLVHAASSSKTANSIVQKPTDPPKDKPIKVIVSGEGKFCYGPNFSGGESYIIIEQCWQMHVTNARY
MAE.iUPMMCNIIDLP_3448557/1-638	---------------MYNWKKILIVVLLASIMVYLEYEMDHTLVHAASSSKTANSIVQKPTDPPKDKPIKVIVSGEGKFCYGPNFSGGESYIIIEQCWQMHVMNARY
MAE.iUPMMCNIIDHP_3448509/1-638	---------------MYNWKKILIVVLLASIMVYLEYEMDHTLVHAASSSKTANSIVQKPTDPPKDKPIKVIVSGEGKFCYGPNFSGGESYIIIEQCWQMHVMNARY
ENO71652.1iVZI_sValbuzzi/1-615	--------------------------------------MDHTLVHAASSSKTANSIVQKPTDPPKDKPIKVIVSGEGKFCYGPNFSGGESYIIIEQCWQMHVTNARY
WP.025181231.1iUNK/1-636	---------------MYNWKKILIVVLLAFIMVYLEYEMDHTLVHAAHASKVASSIVQKPTDPPKDKPIKVNVS-GGTFCYGPTFSGGESYIIIEQCWEMHVKNARY
WP.017215611.1nUNK/1-636	---------------MRYW-KVLVVVLLVSISLSFKYGVNNTPVHA--SSKANNSIVQKPTDPPKDKPIQVVVHDGGKFCYGPTFSGGESYIVIEQCWQMHVKNARY
WP.017215878.1nUNK/1-630	---------------MGRC-IVLIVVLLVSIGVSYSYGGNS----I-SSNGIPGSIVQKPTDPPKDKPIQVVVHDGGKFCYGPTFSGGESYIQIEQCWQMHVKNARY
WP.002179577.1nUNK/1-629	---------------MGRC-IVLIVVLFVSIGVSYSYGGNS----I-SSNGIPGSIVQKPTDPPKDKPIQVVVHDGGKFCYGPTFSGGESYIQIEQCWEQHVKNARY
WP.002177906.1nUNK/1-635	---------------MRYW-KVLVVVLLVSISLSFKYGVNNTPVHA--SSKANNSIVQKPTDPPKDKPIQVVVHDGGKFCYGPTFSGGESYIVIEQCWQMHVKNARY
WP.004427110.1nUNK/1-636	---------------MRNWKKMLIVVLLVSIGVGFEYGESHTSVHAASSSKVNYSITQKPTDKPKDKPIQVVVSDGGKFCYGPIFSGGESYIQIEQCWERHVMNARY
WP.004425726.1nUNK/1-636	---------------MRNWKKMLIVVLLVSIGVGFEYGESHTRVHAASSSKSNYSIVQRPTDKPKDKPIQVVVSDGGKFCYGPIFSGGESYIQIEQCWERHVMNARY
WP.053522368.1nUNK/1-640	---------------MYNWKKMLIVVLLISIGVGFEYGESHTRVHAASSSKSNYSIVQRPTDKPKDKPIQVVVSDGGKFCYGPIFSGGESYIQIEQCWEQHVKNARY
EKR74112.1nUNK_s200600187/1-640	---------------MYNWKKMLIVVLLVSIGVGFEYGESHTRVHAASSSKSNYSIVQRPTDKPKDKPIQVVVSDGGKFCYGPIFSGGESYIQIEQCWERHVKNARY
WP.004446289.1nUNK/1-640	---------------MYNWKKVFIVVLLVSIGVGFEYGKSHTRVHAASSSKVNYSITQRPTDKPKDKPIKVVVSDGGKFCYAPIFSGGESYIQIQQCWEQHVKNARY
WP.004452491.1nUNK/1-634	---------------MRCW-KVLVVVLLVSILLSFRYGINV---HA--SSKANDSIVQKPTDPPKDKPIQVVVSDGGKFCYGPIFSGGESYIIIEQCWEQHVKNARY
WP.004450773.1nUNK/1-640	---------------MYNWKKVFIVVLLVSIMIYLEYKIDHTRVYAASSSKSNYSIVQKPTDKPKDKPIQVVVSDGGKFCYGPIFSGGESYIIIEQCWEQHVKNARY
WP.061248232.1nUNK/1-636	---------------MRNWKKVFIVVLLVSIGVGFEYGESHTRVHAASSSKANYSIAQKPTDKPKDKPIKVVVSDGGKFCYGPIFSGGESYIQIEQCWERHVMNARY
WP.002180375.1nUNK/1-636	---------------MRNWKKVFIVVLLVSIGVGFEYGESHTRVHAASSSKANYSIAQKPTDKPKDKPIKVVVSDGGKFCYGPIFSGGESYIQIEQCWERHVMNARY
WP.004446400.1nUNK/1-636	---------------MRNWKKMLIVVLLVSIGVGFEYGESHTSVHAASSSKVNYSITQKPTDKPKDKPIQVVVSDGGKFCYGPIFSGGESYIQIEQCWERHVMNARY
WP.193825843.1iUNK/1-632	---------------MGRW-IVLLLVLLISLGVGYSYGVNPDVVPI-SSSDIPGSIIQRPTDKPKDKPIKVIVHDGGTFCYGPTFSGGESYIVIEQCWEQDVRNARY
WP.002131229.1iUNK/1-622	--------------------------MLISLGVGYSYGVNPDVVPI-SSSDIPGSIIQRPTDKPKDKPIKVNVSSGGTFCYGPTFSGGESYIVIEQCWEQDVRNARY
WP.002124393.1iUNK/1-632	---------------MGRW-IVLLLVLLISLGVGYSYGVNPDVVPI-SSSDIPGSIIQRPTDKPKDKPIKVNVSSGGTFCYGPTFSGGESYIVIEQCWEQDVRNARY
WP.000533995.1iUNK/1-632	---------------MGRW-IVLLLVLLISLGVGYSYGVNPDVVPI-SSSDIPGSIIQRPTDKPKDKPIKVNVSSGGTFCYGPTFSGGESYIVIEQCWEQDVRNARY
WP.000533994.1iUNK/1-632	---------------MGRW-IVLLLVLLISLGVGYSYGVNPDVVPI-SSSDIPGSIIQRPTDKPKDKPIKVNVSSGGTFCYGPTFSGGESYIVIEQCWEQDVRNARY
PN2Q11_PN2.1569_AG14.iPN2/1-632	---------------MGRW-IVLLLVLLISLGVGYSYGVNPDVVPI-SSSDIPGSIIQRPTDKPKDKPIKVNVSSGGTFCYGPTFSGGESYIVIEQCWEQDVRNARY
WP.096693913.1iUNK/1-632	---------------MGRW-IVLLLVLLISLGVGYSYGVNPDVVPI-SSSDIPGSIIQRPTDKPKDKPIKVNVSSGGTFCYGPTFSGGESYIVIEQCWEQDVRNARY
WP.002107538.1iUNK/1-631	---------------MSRW-IVLLLVLLISLGVGYSYGVNPDVVPI-SSSDIPGAIIQRPTDKPKDKPIKIIVHDGGTFCYGPTFSGGESYIVIEQCWEQDVRNARY
WP.000534001.1iUNK/1-631	---------------MGRW-IVLLLVLLISLGVGYSYGVNPDVVPI-SSSNIPGSIIQRPTDKPKDKPIKVNVSSGGTFCYGPTFSGGESYIVIEQCWEQDVRNARY
KG1Q16_KG1.2075_AG14.uKG1/1-631	---------------MGRW-IILMTVLLVSIGVGYSYGVNPDVVPI-SSNDIPSSIIQRPTDKPKDKPIKIVIHDGGTFCYGPTFSGGESYIVIEQCWQMNVMNARY
EMN70372.1iBAE_sUI08561/1-631	---------------MGRW-IVLLLVLLISLGVGYSYGVNPDVVPI-SSSNIPGSIIQRPTDKPKDKPIKVNVSSGGTFCYGPTFSGGESYIVIEQCWEQDVRNARY
WP.061233745.1iUNK/1-623	--------------------------MLISLGVGYSYGVNPDVVPI-SSNDIPSSIIQRPTDKPKDKPIKVNVSSGGTFCYGPTFSGGESYIVIEQCWEQDVRNARY
WP.002102505.1iUNK/1-632	---------------MGRW-IVLLLVLLISLGVGYSYGVNPDVVPI-SSSDIPGSIIQRPTDKPKDKPIKVNVSSGGTFCYGPTFSGGESYIVIEQCWEQDVRNARY
CLA.iS611_3610805/1-632	---------------MGRW-IVLLLVLLISLGVGYSYGVNPDVVPI-SSSDIPGSIIQRPTDKPKDKPIKVNVSSGGTFCYGPTFSGGESYIVIEQCWEQDVRNARY
CLA.iLJ178_3615796/1-632	---------------MGRW-IVLLLVLLISLGVGYSYGVNPDVVPI-SSSDIPGSIIQRPTDKPKDKPIKVNVSSGGTFCYGPTFSGGESYIVIEQCWEQDVRNARY
WP.002117534.1iUNK/1-631	---------------MGRW-IVLLLVLLISLGVGYSYGVNPDVVPI-SSSDIPGAIIQRPTDKPKDKPIKVNVSSGGTFCYGPTFSGGESYIVIEQCWEQDVRNARY
WP.000534002.1iUNK/1-631	---------------MGRW-IVLLLVLLISLGVGYSYGVNPDVVPI-SSSNIPGSIIQRPTDKPKDKPIKVNVSSGGTFCYGPTFSGGESYIVIEQCWEQDVRNARY
CLA.iS611_943404/1-631	---------------MGRW-IVLLLVLLISLGVGYSYGVNPDVVPI-SSSNIPGSIIQRPTDKPKDKPIKVNVSSGGTFCYGPTFSGGESYIVIEQCWEQDVRNARY
CLA.iLJ178_948647/1-631	---------------MGRW-IVLLLVLLISLGVGYSYGVNPDVVPI-SSSNIPGSIIQRPTDKPKDKPIKVNVSSGGTFCYGPTFSGGESYIVIEQCWEQDVRNARY
WP.002184994.1iUNK/1-631	---------------MSRW-IVLLLVLLISLGVGYSYGVNPDVVPI-SSSDIPGAIIQRPTDKPKDKPIKIIVHDGGTFCYGPTFSGGESYIVIEQCWEQDVRNARY
WP.057140033.1iUNK/1-631	---------------MGRW-IVLLLVLLISLGVGYSYGVNPDVVPI-SSSNIPGSIIQRPTDKPKDKPIKVNVSSGGTFCYGPTFSGGESYIVIEQCWEQDVRNARY
CLA.iRUFN_3140961/1-632	---------------MGRW-IVLLLVLLISLGVGYSYGVNPDVVPI-SSSDIPGSIIQRPTDKPKDKPIKVNVSSGGTFCYGPTFSGGESYIVIEQCWEQDVRNARY
ASV09570.1iCLA_pB2G50.147/1-632	---------------MGRW-IVLLLVLLISLGVGYSYGVNPDVVPI-SSSDIPGSIIQRPTDKPKDKPIKVNVSSGGTFCYGPTFSGGESYIVIEQCWEQDVRNARY
WP.193824755.1iUNK/1-632	---------------MGRW-IVLLLVLLISLGVGYSYGVNPDVVPI-SSSDIPGSIIQRPTDKPKDKPIKVNVSSGGTFCYGPTFSGGESYIVIEQCWEQDVRNARY
WP.071183207.1iUNK/1-631	---------------MGRW-IVLLLVLLISLGVGYSYGVNPDVVPI-SSSDIPGSIIQRPTDKPKDKPIKVNVSSGGTFCYGPTFSGGESYIVIEQCWEQDVRNARY
WP.000533987.1iUNK/1-631	---------------MGRW-IVLLLVLLISLGVGYSYGVNPDVVPI-SSSDIPGAIIQRPTDKPKDKPIKIIVHDGGTFCYGPTFSGGESYIVIEQCWEQDVRNARY
PN2Q1B_PN2.4236_AG14.iPN2/1-631	---------------MGRW-IVLLLVLLISLGVGYSYGVNPDVVPI-SSSDIPGAIIQRPTDKPKDKPIKIIVHDGGTFCYGPTFSGGESYIVIEQCWEQDVRNARY
PN2Q15_PN2.1818_AG14.iPN2/1-631	---------------MSRW-IVLLLVLLISLGVGYSYGVNPDVVPI-SSSDIPGSIIQRPTDKPKDKPIKIIVHDGGTFCYGPTFSGGESYIVIEQCWEQDVRNARY
WP.002072400.1iUNK/1-631	---------------MSRW-IVLLLVLLISLGVGYSYGVNPDVVPI-SSSDIPGAIIQRPTDKPKDKPIKIIVHDGGTFCYGPTFSGGESYIVIEQCWEQDVRNARY
WP.000092393.1iUNK/1-631	---------------MSRW-IVLLLVLLISLGVGYSYGVNPDVVPI-SSSDIPGAIIQRPTDKPKDKPIKIIVHDGGTFCYGPTFSGGESYIVIEQCWEQDVRNARY
WP.061272931.1iUNK/1-631	---------------MSRW-IVLLLVLLISLGVGYSYGVNPDVVPI-SSNDIPSSIIQRPTDKPKDKPIKIVIHDGGTFCYGPTFSGGESYIVIEQCWQMNVMNARY
WP.061237015.1iUNK/1-631	---------------MSRW-IVLLLVLLISLGVGYSYGVNPDVVPI-SSSDIPGSIIQRPTDKPKDKPIKIIVHDGGTFCYGPTFSGGESYIVIEQCWEQDVRNARY
WP.002111374.1iUNK/1-631	---------------MSRW-IVLLLVLLISLGVGYSYGVNPDVVPI-SSSDIPGSIIQRPTDKPKDKPIKIIVHDGGTFCYGPTFSGGESYIVIEQCWEQDVRNARY
WP.002102541.1iUNK/1-631	---------------MSRW-IVLLLVLLISLGVGYSYGVNPDVVPI-SSSDIPGAIIQRPTDKPKDKPIKIIVHDGGTFCYGPTFSGGESYIVIEQCWEQDVRNARY
WP.002100284.1iUNK/1-631	---------------MGRW-IVLLLVLLISLGVGYSYGVNPDVVPI-SSSDIPGSIIQRPTDKPKDKPIKIIVHDGGAFCYGPTFSGGESYIVIEQCWEQDVRNARY
WP.000092395.1iUNK/1-631	---------------MSRW-IVLLLVLLISLGVGYSYGVNPDVVPI-SSSDIPGAIIQRPTDKPKDKPIKIIVHDGGTFCYGPTFSGGESYIVIEQCWEQDVRNARY
CLA.iS611_3367796/1-631	---------------MSRW-IVLLLVLLISLGVGYSYGVNPDVVPI-SSSDIPGAIIQRPTDKPKDKPIKIIVHDGGTFCYGPTFSGGESYIVIEQCWEQDVRNARY
CLA.iS114_916419/1-631	---------------MSRW-IVLLLVLLISLGVGYSYGVNPDVVPI-SSSDIPGAIIQRPTDKPKDKPIKIIVHDGGTFCYGPTFSGGESYIVIEQCWEQDVRNARY
CLA.iLJ178_3372683/1-631	---------------MSRW-IVLLLVLLISLGVGYSYGVNPDVVPI-SSSDIPGAIIQRPTDKPKDKPIKIIVHDGGTFCYGPTFSGGESYIVIEQCWEQDVRNARY
KG1Q1C_KG1.4672_AG14.uKG1/1-631	---------------MGRW-IVLLLVLLISLGVGYSYGVNPDVVPI-SSSDIPGAIIQRPTDKPKDKPIKIIVHDGGTFCYGPTFSGGESYIVIEQCWEQDVRNARY
CLA.iRUFN_64013p/1-631	---------------MSRW-IVLLLVLLISLGVGYSYGVNPDVVPI-SSSDIPGAIIQRPTDKPKDKPIKIIVHDGGTFCYGPTFSGGESYIVIEQCWEQDVRNARY
ASV08116.1iCLA_pB2G50.003/1-631	---------------MSRW-IVLLLVLLISLGVGYSYGVNPDVVPI-SSSDIPGAIIQRPTDKPKDKPIKIIVHDGGTFCYGPTFSGGESYIVIEQCWEQDVRNARY
WP.002183548.1iUNK/1-631	---------------MGRW-IVLLLVLLISLGVGYSYGVNPDVVPI-SSSDIPGAIIQRPTDKPKDKPIKIIVHDGGTFCYGPTFSGGESYIVIEQCWEQDVRNARY
EMN47452.1iUNK_sL1207/1-631	---------------MSRW-IILMTVLLVSIGVGYSYGVNPDVVPI-SSSDIPSSIIQRPTDKPKDKPIKIVIHDGGIFCYGPTFSGGESYIVIEQCWEQDVRNARY
WP.002183832.1iUNK/1-632	---------------MGRL-IVLLLVLLISLGVGYSYGVNPDVVPI-SSSDIPGSIIQRPTDKPKDKPIKVNVSSGGTFCYGPTFSGGESYIVIEQCWQMNVMNARY
WP.002080339.1iUNK/1-632	---------------MGRW-IILMTVLLVSIGVGYSYGVNPDVVPI-SSSDIPGSIIQRPTDKPKDKPIKVNVSSGGTFCYGPNFSGGESYIVIEQCWQMNVMNARY

```
KG1Q11_KG1.12_AG14.uKG1-1/1-632 ---------------MGRW-IVLLLVLLISFGVGYSYGVNPDVVPI-SSSDIPGSIIQRPTDKPKDKPIKVNVSSGGTFCYGPNFSGGESYIVIEQCWQMHVMNAR
WP.048657660.1iUNK/1-631 ---------------MGRW-IVLLLVLLISFGVGYSYGVNPDVVPI-SSSDIPGSIIQRPTDKPKDKPIKVNVSSGGTFCYGPTFSGGESYIVIEQCWQMHVMNAR
MAE.iUPMMCNIIDLP_615075/1-631 ---------------MGRW-IVLLLVLLISFGVGYSYGVNPDVVPI-SSSDIPGSIIQRPTDKPKDKPIKVNVSSGGTFCYGPTFSGGESYIVIEQCWQMHVMNAR
MAE.iUPMMCNIIDHP_615076/1-631 ---------------MGRW-IVLLLVLLISFGVGYSYGVNPDVVPI-SSSDIPGSIIQRPTDKPKDKPIKVNVSSGGTFCYGPTFSGGESYIVIEQCWQMHVMNAR
WP.004486750.1iUNK/1-631 ---------------MSRW-IVLLLVLLISLGVGYSYGVNPDVVPI-SSNDIPSSIIQRPTDKPKDKPIKIVIHDGGTFCYGPTFSGGESYIVIEQCWQMNVMNAR
WP.000533969.1iUNK/1-631 ---------------MGRW-IVLLLVLLISFGVGYSYGVNPDVVPI-SSSDIPGSIIQRPTDKPKDKPIKVNVSSGGTFCYGPNFSGGESYIVIEQCWQMHVMNAR
WP.192505423.1iUNK/1-632 ---------------MGRW-IVLLLVLLISFGVGYSYGVNPDVVPI-SSSDIPGSIIQRPTDKPKDKPIKVNVSSGGTFCYGPNFSGGESYIVIEQCWQMHVMNAR
WP.082279640.1iUNK/1-632 ---------------MSRW-IVLLLVLLISLGVGYSYGVNPDVVPI-SSNDIPSSIIQRPTDKPKDKPIKVNVSSGGTFCYGPNFSGGESYIVIEQCWQMHVMNAR
WP.060762739.1iUNK/1-632 ---------------MGRW-IVLLLVLLISLGVGYSYGVNPDVVPI-SSSDIPGSIIQRPTDKPKDKPIKVNVSSGGTFCYGPTFSGGESYIVIEQCWQMHVMNAR
WP.060589296.1iUNK/1-632 ---------------MGRW-IVLLLVLLISFGVGYSYGVNPDVVPI-SSSDIPGSIIQRPTDKPKDKPIKVNVSSGGTFCYGPNFSGGESYIVIEQCWQMHVMNAR
WP.033110993.1iUNK/1-622 --------------------------MLISFGVGYSYGVNPDVVPI-SSSDIPGSIIQRPTDKPKDKPIKVNVSSGGTFCYGPNFSGGESYIVIEQCWQMHVMNAR
WP.002189465.1iUNK/1-632 ---------------MGRW-IVLLLVLLISFGVGYSYGVNPDVVPI-SSSDIPGSIIQRPTDKPKDKPIKVNVSSGGTFCYGPNFSGGESYIVIEQCWQMHVMNAR
WP.001216430.1iUNK/1-631 ---------------MRHS-IVFIVVLLVLVTTSFEYSINNIHIHA--SSNTSNSIIQKPTDQPKDKPIKIVIHDGKKFCYAPVFSGGESYIVIEQCWQMHVMNAR
WP.000533998.1iUNK/1-632 ---------------MGRW-IVLLLVLLISLGVGYSYGVNPDVVPI-SSSDIPGSIIQRPTDKPKDKPIKVNVSSGGTFCYGPTFSGGESYIVIEQCWQMNVMNAR
WP.000533997.1iUNK/1-632 ---------------MGRW-IVLLLVLLISLGVGYSYGVNPDVVPI-SSSDIPGSIIQRPTDKPKDKPIKVNVSSGGTFCYGPTFSGGESYIVIEQCWQMHVMNAR
WP.000533996.1iUNK/1-632 ---------------MGRW-IVLLLVLLISLGVGYSYGVNPDVVPI-SSSDIPGSIIQRPTDKPKDKPIKVNVSSGGTFCYGPTFSGGESYIVIEQCWEQDVRNAR
WP.000533992.1iUNK/1-632 ---------------MGRW-IVLLLVLLISLGVGYSYGVNPDVVPI-SSSDIPGSIIQRPTDKPKDKPIKVNVSSGGTFCYGPNFSGGESYIVIEQCWQMHVMNAR
WP.000533970.1iUNK/1-632 ---------------MGRW-IVLLLVLLISFGVGYSYGVNPDVVPI-SSSDIPGSIIQRPTDKPKDKPIKVNVSSGGTFCYGPNFSGGESYIVIEQCWQMHVMNAR
WP.000533964.1iUNK/1-632 ---------------MGRW-IVLLLVLLISFGVGYSYGVNPDVVPI-SSSDIPGSIIQRPTDKPKDKPIKVNVSSGGTFCYGPNFSGGESYIVIEQCWQMHVMNAR
WP.000533950.1iUNK/1-632 ---------------MGRW-IVLLLALLISFGVGYSYGVNPDVVPI-SSSDIPGSIIQRPTDKPKDKPIKVNVSSGGTFCYGPNFSGGESYIVIEQCWQMHVMNAR
UNK.iRCA_4044988/1-632 ---------------MGRW-IVLLLVLLISFGVGYSYGVNPDVVPI-SSSDIPGSIIQRPTDKPKDKPIKVNVSSGGTFCYGPNFSGGESYIVIEQCWQMHVMNAR
UNK.iR7_3576070/1-632 ---------------MGRW-IVLLLVLLISFGVGYSYGVNPDVVPI-SSSDIPGSIIQRPTDKPKDKPIKVNVSSGGTFCYGPNFSGGESYIVIEQCWQMNVMNAR
UNK.iR22_3585775/1-632 ---------------MGRW-IVLLLVLLISFGVGYSYGVNPDVVPI-SSSDIPGSIIQRPTDKPKDKPIKVNVSSGGTFCYGPNFSGGESYIVIEQCWQMHVMNAR
UNK.iR21_3586966/1-632 ---------------MGRW-IVLLLVLLISFGVGYSYGVNPDVVPI-SSSDIPGSIIQRPTDKPKDKPIKVNVSSGGTFCYGPNFSGGESYIVIEQCWQMHVMNAR
UNK.iR19_3585608/1-632 ---------------MGRW-IVLLLVLLISFGVGYSYGVNPDVVPI-SSSDIPGSIIQRPTDKPKDKPIKVNVSSGGTFCYGPNFSGGESYIVIEQCWQMHVMNAR
UNK.iR17_3586417/1-632 ---------------MGRW-IVLLLVLLISFGVGYSYGVNPDVVPI-SSSDIPGSIIQRPTDKPKDKPIKVNVSSGGTFCYGPNFSGGESYIVIEQCWQMHVMNAR
UNK.iR16_3576612/1-632 ---------------MGRW-IVLLLVLLISFGVGYSYGVNPDVVPI-SSSDIPGSIIQRPTDKPKDKPIKVNVSSGGTFCYGPNFSGGESYIVIEQCWQMHVMNAR
UNK.iR13_3576784/1-632 ---------------MGRW-IVLLLVLLISFGVGYSYGVNPDVVPI-SSSDIPGSIIQRPTDKPKDKPIKVNVSSGGTFCYGPNFSGGESYIVIEQCWQMHVMNAR
UNK.iR11_3577253/1-632 ---------------MGRW-IVLLLVLLISFGVGYSYGVNPDVVPI-SSSDIPGSIIQRPTDKPKDKPIKVNVSSGGTFCYGPNFSGGESYIVIEQCWQMNVMNAR
QHH54692.1iUNK/1-632 ---------------MGRW-IVLLLVLLISFGVGYSYGVNPDVVPI-SSSDIPGSIIQRPTDKPKDKPIKVNVSSGGTFCYGPNFSGGESYIVIEQCWQMNVMNAR
ICT.iS898_3629867/1-632 ---------------MGRW-IVLLLVLLISFGVGYSYGVNPDVVPI-SSSDIPGSIIQRPTDKPKDKPIKVNVSSGGTFCYGPNFSGGESYIVIEQCWQMHVMNAR
ICT.iPiscina_3583239/1-632 ---------------MGRW-IVLLLVLLISFGVGYSYGVNPDVVPI-SSSDIPGSIIQRPTDKPKDKPIKVNVSSGGTFCYGPNFSGGESYIVIEQCWQMHVMNAR
ICT.iLangkawi_3710243/1-632 ---------------MGRW-IVLLLVLLISFGVGYSYGVNPDVVPI-SSSDIPGSIIQRPTDKPKDKPIKVNVSSGGTFCYGPNFSGGESYIVIEQCWQMHVMNAR
HJO.iNorma_3762078/1-632 ---------------MGRW-IVLLLVLLISFGVGYSYGVNPDVVPI-SSSDIPGSIIQRPTDKPKDKPIKVNVSSGGTFCYGPNFSGGESYIVIEQCWQMHVMNAR
HJO.iL53_3707782/1-632 ---------------MGRW-IVLLLVLLISFGVGYSYGVNPDVVPI-SSSDIPGSIIQRPTDKPKDKPIKVNVSSGGTFCYGPNFSGGESYIVIEQCWQMHVMNAR
HJO.iHjtno_3689146/1-632 ---------------MGRW-IVLLLVLLISFGVGYSYGVNPDVVPI-SSSDIPGSIIQRPTDKPKDKPIKVNVSSGGTFCYGPNFSGGESYIVIEQCWQMHVMNAR
EMM94407.1iZNI_sLT2156/1-632 ---------------MGRW-IVLLLVLLISFGVGYSYGVNPDVVPI-SSSDIPGSIIQRPTDKPKDKPIKVNVSSGGTFCYGPNFSGGESYIVIEQCWQMHVMNAR
COP.iSK1_3629895/1-632 ---------------MGRW-IVLLLVLLISFGVGYSYGVNPDVVPI-SSSDIPGSIIQRPTDKPKDKPIKVNVSSGGTFCYGPNFSGGESYIVIEQCWQMHVMNAR
COP.iL1130_Q72N52.cl3626/1-632 ---------------MGRW-IVLLLVLLISFGVGYSYGVNPDVVPI-SSSDIPGSIIQRPTDKPKDKPIKVNVSSGGTFCYGPNFSGGESYIVIEQCWQMHVMNAR
COP.iFDAARGOS203_1639452/1-632 ---------------MGRW-IVLLLVLLISFGVGYSYGVNPDVVPI-SSSDIPGSIIQRPTDKPKDKPIKVNVSSGGTFCYGPNFSGGESYIVIEQCWQMHVMNAR
CLA.iS782_3905355/1-632 ---------------MGRW-IVLLLVLLISFGVGYSYGVNPDVVPI-SSSDIPGSIIQRPTDKPKDKPIKVNVSSGGTFCYGPNFSGGESYIVIEQCWQMHVMNAR
BVA.iPigK151_3745512/1-632 ---------------MGRW-IVLLLVLLISLGVGYSYGVNPDVVPI-SSSDIPGSIIQRPTDKPKDKPIKVNVSSGGTFCYGPTFSGGESYIVIEQCWQMHVMNAR
BAE.iS1548_3703152/1-632 ---------------MGRW-IVLLLVLLISFGVGYSYGVNPDVVPI-SSSDIPGSIIQRPTDKPKDKPIKVNVSSGGTFCYGPNFSGGESYIVIEQCWQMHVMNAR
BAE.iS1489_3768689/1-632 ---------------MGRW-IVLLLVLLISFGVGYSYGVNPDVVPI-SSSDIPGSIIQRPTDKPKDKPIKVNVSSGGTFCYGPNFSGGESYIVIEQCWQMHVMNAR
AW3Q1C_AW3.4149_AG10.iAW3/1-632 ---------------MGRW-IVLLLVLLISFGVGYSYGVNPDVVPI-SSSDIPGSIIQRPTDKPKDKPIKVNVSSGGTFCYGPNFSGGESYIVIEQCWQMHVMNAR
AP6Q1C_AP6.3790_AG3.iAP6-/1-632 ---------------MGRW-IVLLLALLISFGVGYSYGVNPDVVPI-SSSDIPGSIIQRPTDKPKDKPIKVNVSSGGTFCYGPNFSGGESYIVIEQCWQMHVMNAR
AP5Q1C_AP5.3712_AG10.iAP5/1-632 ---------------MGRW-IVLLLALLISFGVGYSYGVNPDVVPI-SSSDIPGSIIQRPTDKPKDKPIKVNVSSGGTFCYGPNFSGGESYIVIEQCWQMHVMNAR
WP.192486093.1iUNK/1-632 ---------------MGRW-IVLLLVLLISFGVGYSYGVNPDVVPI-SSSDIPGSIIQRPTDKPKDKPIKVNVSSGGTFCYGPNFSGGESYIVIEQCWQMHVMNAR
EMN48101.1iUNK_sL1207/1-632 ---------------MGRW-IVLLLVLLILLGVGYSYGVNPDVVPI-SSSDIPGSIIQRPTDKPKDKPIKVNVSSGGTFCYGPTFSGGESYIVIEQCWQMHVMNAR
WP.002125847.1iUNK/1-632 ---------------MGRW-IVLLLVLLISFGVGYSYGVNPDVVPI-SSSDIPGSIIQRPTDKPKDKPIKVNVSSGGTFCYGPNFSGGESYIVIEQCWQMHVMNAR
WP.061243961.1iUNK/1-632 ---------------MGRW-IVLLLALLISFGVGYSYGVNPDVVPI-SSSDIPGSIIQRPTDKPKDKPIKVNVSSGGTFCYGPNFSGGESYIVIEQCWQMHVMNAR
WP.002109284.1iUNK/1-622 --------------------------MLISFGVGYSYGVNPDVVPI-SSSDIPGSIIQRPTDKPKDKPIKVNVSSGGTFCYGPNFSGGESYIVIEQCWQMHVMNAR
WP.000533966.1iUNK/1-632 ---------------MGRW-IVLLLVLLISFGVGYSYGVNPDVVPI-SSSDIPGSIIQRPTDKPKDKPIKVNVSSGGTFCYGPNFSGGESYIVIEQCWQMHVMNAR
LHI.iS56609_3694022/1-632 ---------------MGRW-IVLLLVLLISFGVGYSYGVNPDVVPI-SSSDIPGSIIQRPTDKPKDKPIKVNVSSGGTFCYGPNFSGGESYIVIEQCWQMHVMNAR
WP.048657713.1iUNK/1-631 ---------------MGRW-IVLLLVLLVSLGVGYSYGVNPDVVPI-SSSDIPGSIIQRPTDKPKDKPIKVNVSSGGTFCYGPTFSGGESYIVIEQCWQMHVMNAR
MAE.iUPMMCNIIDLP_843910/1-631 ---------------MGRW-IVLLLVLLVSLGVGYSYGVNPDVVPI-SSSDIPGSIIQRPTDKPKDKPIKVNVSSGGTFCYGPTFSGGESYIVIEQCWQMHVMNAR
MAE.iUPMMCNIIDHP_843912/1-631 ---------------MGRW-IVLLLVLLVSLGVGYSYGVNPDVVPI-SSSDIPGSIIQRPTDKPKDKPIKVNVSSGGTFCYGPTFSGGESYIVIEQCWQMHVMNAR
WP.061296659.1iUNK/1-632 ---------------MGRW-IVLLLALLISFGVGYSYGVNPDVVPI-SSSDIPGSIIQRPTDKPKDKPIKVNVSSGGTFCYGPNFSGGESYIVIEQCWQMHVMNAR
WP.061284025.1iUNK/1-632 ---------------MGRW-IVLLLALLISFGVGYSYGVNPDVVPI-SSSDIPGSIIQRPTDKPKDKPIKVNVSSGGTFCYGPNFSGGESYIVIEQCWQMHVMNAR
WP.000533973.1iUNK/1-632 ---------------MGRW-IVLLLVLLISFGVGYSYGVNPDVVPI-SSSDIPGSIIQRPTDKPKDKPIKVNVSSGGTFCYGPNFSGGESYIVIEQCWQMHVMNAR
LAI.iS56601_597327/1-632 ---------------MGRW-IVLLLVLLISFGVGYSYGVNPDVVPI-SSSDIPGSIIQRPTDKPKDKPIKVNVSSGGTFCYGPNFSGGESYIVIEQCWQMHVMNAR
LAI.iIPAV_596971/1-632 ---------------MGRW-IVLLLVLLISFGVGYSYGVNPDVVPI-SSSDIPGSIIQRPTDKPKDKPIKVNVSSGGTFCYGPNFSGGESYIVIEQCWQMHVMNAR
WP.000533972.1iUNK/1-631 ---------------MGRW-IVLLLVLLISFGVGYSYGVNPDVVPI-SSSDIPGSIIQRPTDKPKDKPIKVNVSSGGTFCYGPNFSGGESYIVIEQCWQMHVMNAR
EMF74456.1iCLA_sLT1962/1-632 ---------------MGRW-IVLLLALLISFGVGYSYGVNPDVVPI-SSSDIPGSIIQRPTDKPKDKPIKVNVSSGGTFCYGPNFSGGESYIVIEQCWQMNVMNAR
WP.137119312.1iUNK/1-632 ---------------MGRW-IVLLLVLLISFGVGYSYGVNPDVVPI-SSSDIPGSIIQRPTDKPKDKPIKIVIHDGGTFCYSPTFSGGESYIVIEQCWEQDVRNAR
WP.137108644.1iUNK/1-632 ---------------MGRW-IVLLLVLLISFGVGYSYGVNPDVVPI-SSSDIPGSIIQRPTDKPKDKPIKIVIHDGGTFCYSPTFSGGESYIVIEQCWEQDVRNAR
RT2Q11_RT2.596_AG3.iRT2-1/1-632 ---------------MGRW-IVLLLVLLISFGVGYSYGVNPDVVPI-SSSDIPGSIIQRPTDKPKDKPIKIVIHDGGTFCYSPTFSGGESYIVIEQCWEQDVRNAR
PN3Q11_SLPN3.695_AG3.iPN3/1-632 ---------------MGRW-IVLLLVLLISFGVGYSYGVNPDVVPI-SSSDIPGSIIQRPTDKPKDKPIKIVIHDGGTFCYSPTFSGGESYIVIEQCWEQDVRNAR
KW2Q11_KW2.606_AG3.iKW2-1/1-632 ---------------MGRW-IVLLLVLLISFGVGYSYGVNPDVVPI-SSSDIPGSIIQRPTDKPKDKPIKIVIHDGGTFCYSPTFSGGESYIVIEQCWEQDVRNAR
KW2Q11_KW2.594_AG3.iKW2-1/1-632 ---------------MGRW-IVLLLVLLISFGVGYSYGVNPDVVPI-SSSDIPGSIIQRPTDKPKDKPIKIVIHDGGTFCYSPTFSGGESYIVIEQCWEQDVRNAR
KW1Q1D_KW1.3533_AG3.iKW1-/1-632 ---------------MGRW-IVLLLVLLISFGVGYSYGVNPDVVPI-SSSDIPGSIIQRPTDKPKDKPIKIVIHDGGTFCYSPTFSGGESYIVIEQCWEQDVRNAR
AW1Q11_AW1.406_AG3.iAW1-1/1-632 ---------------MGRW-IVLLLVLLISFGVGYSYGVNPDVVPI-SSSDIPGSIIQRPTDKPKDKPIKIVIHDGGTFCYSPTFSGGESYIVIEQCWEQDVRNAR
AP7Q11_AP7.592_AG3.iAP7-1/1-632 ---------------MGRW-IVLLLVLLISFGVGYSYGVNPDVVPI-SSSDIPGSIIQRPTDKPKDKPIKIVIHDGGTFCYSPTFSGGESYIVIEQCWEQDVRNAR
AP3Q11_AP3.695_AG10.iAP3-/1-632 ---------------MGRW-IVLLLVLLISFGVGYSYGVNPDVVPI-SSSDIPGSIIQRPTDKPKDKPIKIVIHDGGTFCYSPTFSGGESYIVIEQCWEQDVRNAR
AP1Q11_AP1.1117_AG3.iAP1-/1-632 ---------------MGRW-IVLLLVLLISFGVGYSYGVNPDVVPI-SSSDIPGSIIQRPTDKPKDKPIKIVIHDGGTFCYSPTFSGGESYIVIEQCWEQDVRNAR
AG3Q11_U1.695_AG3.iO73718/1-632 ---------------MGRW-IVLLLVLLISFGVGYSYGVNPDVVPI-SSSDIPGSIIQRPTDKPKDKPIKIVIHDGGTFCYSPTFSGGESYIVIEQCWEQDVRNAR
WP.061266343.1iUNK/1-632 ---------------MGRW-IVLLLVLLISLGVGYSYGVNPDVVPI-SSSDIPGSIIQRPTDKPKDKPIKVNVSSGGTFCYGPNFSGGESYIVIEQCWQMHVMNAR
WP.000533959.1iUNK/1-631 ---------------MGRW-IVLLLVLLISFGVGYSYGVNPDVVPI-SSSDIPGSIIQRPTDKPKDKPIKIVVHDGGTFCYAPVFIGGESYIMIEQCWEQDIRNAR
PD1Q1E_PD1.4351_AG3.iPD1-/1-632 ---------------MGRW-IVLLLVLLISFGVGYSYGVNPDVVPI-SSSDIPGSIIQRPTDKPKDKPIKIVIHDGGTFCYSPTFSGGESYIVIEQCWEQDVRNAR
KG2Q11_KG2.1463_AG3.uKG2-/1-632 ---------------MGRW-IVLLLVLLISFGVGYSYGVNPDVVPI-SSSDIPGSIIQRPTDKPKDKPIKIVIHDGGTFCYSPTFSGGESYIVIEQCWEQDVRNAR
WP.061266449.1iUNK/1-631 ---------------MGRW-IILMTVLLVSIGVGYSYGVNPDVVPI-SSNDIPSSIIQRPTDKPKDKPIKIVIHDGGTFCYGPTFSGGESYIVIEQCWQMNVMNAR
WP.175270780.1iUNK/1-632 ---------------MGRW-IVLLLVLLISFGVGYSYGVNPDVVPI-SSSDIPSSIIQRPTDKPKDKPIKIVIHDGGTFCYGPNFSGGESYIVIEQCWQMNVMNAR
WP.033109400.1iUNK/1-631 ---------------MGRW-RVLIAILLISLGVGYSYGVNPDVVPI-SSSDIPGSIIQRPTDKPKDKPIKIVIHDGGTFCYGPTFSGGESYIVIEQCWEQDVRNAR
MAE.iUPMMCNIIDLP_3280773/1-631 ---------------MGRW-RVLIAILLISLGVGYSYGVNPDVVPI-SSSDIPGSIIQRPTDKPKDKPIKIVIHDGGTFCYGPTFSGGESYIVIEQCWEQDVRNAR
MAE.iUPMMCNIIDHP_3280726/1-631 ---------------MGRW-RVLIAILLISLGVGYSYGVNPDVVPI-SSSDIPGSIIQRPTDKPKDKPIKIVIHDGGTFCYGPTFSGGESYIVIEQCWEQDVRNAR
EMN73081.1iBAE_sUI08561/1-631 ---------------MSRW-IVLLLVLLISLGVGYSYGVNPDVVPI-SSNDIPSSIIQRPTDKPKDKPIKIVIHDGGTFCYGPNFSGGESYIVIEQCWQMNVMNAR
WP.148636708.1iUNK/1-629 ---------------MSRW-IVLLLVLLISLGVGYSYGVNPDVVPI-SSNDIPSSIIQRPTDKPKDKPIKIVIHDGGTFCYGPNFSGGESYIVIEQCWQMNVMNAR
WP.082283152.1iUNK/1-631 ---------------MGRW-IILMTVLLVSIGVGYSYGVNPDVVPI-SSNDIPSSIIQRPTDKPKDKPIKIVIHDGGTFCYGPTFSGGESYIVIEQCWQMNVMNAR
WP.060684566.1iUNK/1-631 ---------------MSRW-IVLLLVLLISLGVGYSYGVNPDVVPI-SSNDIPSSIIQRPTDKPKDKPIKIVIHDGGTFCYGPNFSGGESYIVIEQCWQMNVMNAR
WP.000092387.1iUNK/1-631 ---------------MSRW-IVLLLVLLISLGVGYSYGVNPDVVPI-SSNDIPSSIIQRPTDKPKDKPIKIVIHDGGTFCYGPTFSGGESYIVIEQCWQMNVMNAR
HJO.iNorma_940593/1-631 ---------------MSRW-IVLLLVLLISLGVGYSYGVNPDVVPI-SSNDIPSSIIQRPTDKPKDKPIKIVIHDGGTFCYGPNFSGGESYIVIEQCWQMNVMNAR
HJO.iL53_950996/1-629 ---------------MSRW-IVLLLVLLISLGVGYSYGVNPDVVPI-SSNDIPSSIIQRPTDKPKDKPIKIVIHDGGTFCYGPNFSGGESYIVIEQCWQMNVMNAR
ALE38207.1iHJO_sNorma/1-624 -----------------------MLVLLISLGVGYSYGVNPDVVPI-SSNDIPSSIIQRPTDKPKDKPIKIVIHDGGTFCYGPNFSGGESYIVIEQCWQMNVMNAR
WP.137119337.1iUNK/1-631 ---------------MGRW-IVLLLVLLISFGVGYSYGVNPDVVPI-SSNDIPSSIIQRPTDKPKDKPIKIVIHDGGTFCYGPTFSGGESYIVIEQCWQMNVMNAR
WP.002106187.1iUNK/1-631 ---------------MSRW-IVLLLVLLISLGVGYSYGVNPDVVPI-SSNDIPSSIIQRPTDKPKDKPIKIVIHDGGTFCYGPTFSGGESYIVIEQCWQMNVMNAR
PD1Q1A_PD1.4029_AG3.iPD1-/1-631 ---------------MGRW-IVLLLVLLISFGVGYSYGVNPDVVPI-SSNDIPGSIIQRPTDKPKDKPIKIVIHDGGTFCYGPTFSGGESYIVIEQCWQMNVMNAR
LHI.iS56609_3455446/1-631 ---------------MSRW-IVLLLVLLISLGVGYSYGVNPDVVPI-SSNDIPSSIIQRPTDKPKDKPIKIVIHDGGTFCYGPTFSGGESYIVIEQCWQMNVMNAR
KG2Q15_KG2.1789_AG3.uKG2-/1-631 ---------------MGRW-IVLLLVLLISFGVGYSYGVNPDVVPI-SSNDIPGSIIQRPTDKPKDKPIKIVIHDGGTFCYGPTFSGGESYIVIEQCWQMNVMNAR
CLA.iS782_3676212/1-631 ---------------MSRW-IVLLLVLLISLGVGYSYGVNPDVVPI-SSNDIPSSIIQRPTDKPKDKPIKIVIHDGGTFCYGPTFSGGESYIVIEQCWQMNVMNAR
BAE.iS1548_3469488/1-631 ---------------MSRW-IVLLLVLLISLGVGYSYGVNPDVVPI-SSNDIPSSIIQRPTDKPKDKPIKIVIHDGGTFCYGPTFSGGESYIVIEQCWQMNVMNAR
AW1Q15_AW1.720_AG3.iAW1-1/1-631 ---------------MGRW-IVLLLVLLISFGVGYSYGVNPDVVPI-SSNDIPSSIIQRPTDKPKDKPIKIVIHDGGTFCYGPTFSGGESYIVIEQCWQMNVMNAR
WP.000092385.1iUNK/1-631 ---------------MSRW-IVLLLVLLISLGVGYSYGVNPDVVPI-SSNDIPSSIIQRPTDKPKDKPIKIVIHDGGTFCYGPTFSGGESYIVIEQCWQMNVMNAR
ICT.iS898_3396688/1-631 ---------------MSRW-IVLLLVLLISLGVGYSYGVNPDVVPI-SSNDIPSSIIQRPTDKPKDKPIKIVIHDGGTFCYGPTFSGGESYIVIEQCWQMNVMNAR
ICT.iPiscina_3353041/1-631 ---------------MSRW-IVLLLVLLISLGVGYSYGVNPDVVPI-SSNDIPSSIIQRPTDKPKDKPIKIVIHDGGTFCYGPTFSGGESYIVIEQCWQMNVMNAR
COP.iSK1_3396557/1-631 ---------------MSRW-IVLLLVLLISLGVGYSYGVNPDVVPI-SSNDIPSSIIQRPTDKPKDKPIKIVIHDGGTFCYGPTFSGGESYIVIEQCWQMNVMNAR
[illegible]
```

```
COP.iL1130_Q72NF1.cl3396/1-631 ---------------MSRW-IVLLLVLLISLGVGYSYGVNPDVVPI-SSNDIPSSIIQRPTDKPKDKPIKIVIHDGGTFCYGPTFSGGESYIVIEQCWQMNVMNAR
COP.iFDAARGOS203_1406112/1-631 ---------------MSRW-IVLLLVLLISLGVGYSYGVNPDVVPI-SSNDIPSSIIQRPTDKPKDKPIKIVIHDGGTFCYGPTFSGGESYIVIEQCWQMNVMNARY
APH42576.1iICT/1-631 ---------------MSRW-IVLLLVLLISLGVGYSYGVNPDVVPI-SSNDIPSSIIQRPTDKPKDKPIKIVIHDGGTFCYGPTFSGGESYIVIEQCWQMNVMNARY
WP.000092371.1iUNK/1-631 ---------------MSRW-IVLLLVLLISLGVGYSYGVNPDVVPI-SSNDIPSSIIQRPTDKPKDKPIKIVIHDGGTFCYGPNFSGGESYIVIEQCWQMHVMNARY
WP.047020782.1iUNK/1-631 ---------------MSRW-IVLLLVLLISLGVGYSYGVNPDVVPI-SSNDIPSSIIQRPTDKPKDKPIKIVIHDGGTFCYGPTFSGGESYIVIEQCWQMNVMNARY
WP.002097590.1iUNK/1-624 -----------------------MLVLLISLGVGYSYGVNPDVVPI-SSNDIPSSIIQRPTDKPKDKPIKIVIHDGGTFCYGPTFSGGESYIVIEQCWQMNVMNARY
WP.000092386.1iUNK/1-631 ---------------MSRW-IVLLLVLLISLGVGYSYGVNPDVVPI-SSNDIPSSIIQRPTDKPKDKPIKIVIHDGGTFCYGPTFSGGESYIVIEQCWQMNVMNARY
BVA.iPigK151_3515872/1-631 ---------------MSRW-IVLLLVLLISLGVGYSYGVNPDVVPI-SSNDIPSSIIQRPTDKPKDKPIKIVIHDGGTFCYGPTFSGGESYIVIEQCWQMNVMNARY
WP.002109132.1iUNK/1-631 ---------------MSRW-IVLLLVLLISLGVGYSYGVNPDVVPI-SSNDIPSSIIQRPTDKPKDKPIKIVIHDGGTFCYGPTFSGGESYIVIEQCWQMNVMNARY
WP.002079523.1iUNK/1-631 ---------------MSRW-IVLLLVLLISLGVGYSYGVNPDVVPI-SSSDIPGAIIQRPTDKPKDKPIKIVIHDGGTFCYGPTFSGGESYIVIEQCWQMNVMNARY
WP.000092367.1iUNK/1-631 ---------------MSRW-IILMTVLLVSIGVGYSYGVNPDVVPI-SSNDIPSSIIQRPTDKPKDKPIKIVIHDGGTFCYGPTFSGGESYIVIEQCWQMNVMNARY
LAI.iS56601_835852/1-631 ---------------MSRW-IILMTVLLVSIGVGYSYGVNPDVVPI-SSNDIPSSIIQRPTDKPKDKPIKIVIHDGGTFCYGPTFSGGESYIVIEQCWQMNVMNARY
LAI.iIPAV_835244/1-631 ---------------MSRW-IILMTVLLVSIGVGYSYGVNPDVVPI-SSNDIPSSIIQRPTDKPKDKPIKIVIHDGGTFCYGPTFSGGESYIVIEQCWQMNVMNARY
ICT.iLangkawi_3454877/1-631 ---------------MSRW-IVLLLVLLISLGVGYSYGVNPDVVPI-SSNDIPSSIIQRPTDKPKDKPIKIVIHDGGTFCYGPTFSGGESYIVIEQCWQMNVMNARY
EMP08707.1iPES_s200701872/1-631 ---------------MSRW-IVLLLVLLISLGVGYSYGVNPDVVPI-SSSDIPGAIIQRPTDKPKDKPIKIVIHDGGTFCYGPTFSGGESYIVIEQCWQMNVMNARY
EMG10849.1iGSA_sLT2186/1-631 ---------------MSRW-IVLLLVLLISLGVGYSYGVNPDVVPI-SSNDIPSSIIQRPTDKPKDKPIKIVIHDGGTICYGPTFSGGESYIVIEQCWQMNVMNARY
BAE.iS1489_3503508/1-631 ---------------MSRW-IVLLLVLLISLGVGYSYGVNPDVVPI-SSNDIPSSIIQRPTDKPKDKPIKIVIHDGGTFCYGPTFSGGESYIVIEQCWQMNVMNARY
AW3Q19_AW3.3901_AG10.iAW3/1-631 ---------------MSRW-IVLLLVLLISLGVGYSYGVNPDVVPI-SSSDIPGAIIQRPTDKPKDKPIKIVIHDGGTFCYGPTFSGGESYIVIEQCWQMNVMNARY
AP6Q19_AP6.3548_AG3.iAP6-/1-631 ---------------MSRW-IVLLLVLLISLGVGYSYGVNPDVVPI-SSSDIPGAIIQRPTDKPKDKPIKIVIHDGGTFCYGPTFSGGESYIVIEQCWQMNVMNARY
AP5Q19_AP5.3471_AG10.iAP5/1-631 ---------------MSRW-IVLLLVLLISLGVGYSYGVNPDVVPI-SSSDIPGAIIQRPTDKPKDKPIKIVIHDGGTFCYGPTFSGGESYIVIEQCWQMNVMNARY
WP.002095701.1iUNK/1-631 ---------------MSRW-IVLLLVLLISLGVGYSYGVNPDVVPI-SSSDIPGAIIQRPTDKPKDKPIKVNVSSGGTFCYGPNFSGGESYIVIEQCWQMHVMNARY
WP.025185574.1iUNK/1-631 ---------------MGRW-IILMTVLLVSIGVGYSYGVNPDVVPI-SSNDIPSSIIQRPTDKPKDKPIKIVIHDGGTFCYGPTFSGGESYIVIEQCWQMNVMNARY
EMM90655.1iDAN_sLT1649/1-624 -----------------------mTVLLVSIGVGYSYGVNPDVVPI-SSNDIPSSIIQRPTDKPKDKPIKIVIHDGGTFCYGPTFSGGESYIVIEQCWQMNVMNARY
WP.000533942.1iUNK/1-631 ---------------MGRW-IILMTVLLVSIGVGYSYGVNPDVVPI-SSNDIPSSIIQRPTDKPKDKPIKIVIHDGGTFCYGPTFSGGESYIVIEQCWQMNVMNARY
LHI.iS56609_942472/1-631 ---------------MGRW-IILMTVLLVSIGVGYSYGVNPDVVPI-SSNDIPSSIIQRPTDKPKDKPIKIVIHDGGTFCYGPTFSGGESYIVIEQCWQMNVMNARY
WP.192503325.1iUNK/1-631 ---------------MGRW-IILMTVLLVSIGVGYSYGVNPDVVPI-SSNDIPSSIIQRPTDKPKDKPIKIVIHDGGTFCYGPTFSGGESYIVIEQCWQMNVMNARY
ICT.iLangkawi_966139/1-631 ---------------MGRW-IILMTVLLVSIGVGYSYGVNPDVVPI-SSNDIPSSIIQRPTDKPKDKPIKIVIHDGGTFCYGPTFSGGESYIVIEQCWQMNVMNARY
WP.000092389.1iUNK/1-631 ---------------MSRW-IVLLLVLLISLGVGYSYGVNPDVVPI-SSNDIPSSIIQRPTDKPKDKPIKIVIHDGGTFCYGPTFSGGESYIVIEQCWQMNVMNARY
WP.002096150.1iUNK/1-631 ---------------MSRW-IVLLLVLLISLGVGYSYGVNPDVVPI-SSNDIPSSIIQRPTDKPKDKPIKIVIHDGGTFCYGPTFSGGESYIVIEQCWQMNVMNARY
WP.000533940.1iUNK/1-631 ---------------MGRW-IILMTVLLVSIGVGYSYGVNPDVVPI-SSNDIPSSIIQRPTDKPKDKPIKIVIHDGGTFCYGPTFSGGESYIVIEQCWQMNVMNARY
WP.029781545.1iUNK/1-624 -----------------------mTVLLVSIGVGYSYGVNPDVVPI-SSNDIPSSIIQRPTDKPKDKPIKIVIHDGGTFCYGPTFSGGESYIVIEQCWQMNVMNARY
WP.192486201.1iUNK/1-631 ---------------MGRW-IILMTVLLVSIGVGYSYGVNPDVVPI-SSNDIPSSIIQRPTDKPKDKPIKIVIHDGGTFCYGPTFSGGESYIVIEQCWQMNVMNARY
WP.002118986.1iUNK/1-631 ---------------MGRW-IILMTVLLVSIGVGYSYGVNPDVVPI-SSNDIPSSIIQRPTDKPKDKPIKIVIHDGGTFCYGPTFSGGESYIVIEQCWQMNVMNARY
WP.000533943.1iUNK/1-631 ---------------MGRW-IILMTVLLVSIGVGYSYGVNPDVVPI-SSNDIPSSIIQRPTDKPKDKPIKIVIHDGGTFCYGPTFSGGESYIVIEQCWQMNVMNARY
CLA.iS782_917529/1-631 ---------------MGRW-IILMTVLLVSIGVGYSYGVNPDVVPI-SSNDIPSSIIQRPTDKPKDKPIKIVIHDGGTFCYGPTFSGGESYIVIEQCWQMNVMNARY
BAE.iS1489_947479/1-631 ---------------MGRW-IILMTVLLVSIGVGYSYGVNPDVVPI-SSNDIPSSIIQRPTDKPKDKPIKIVIHDGGTFCYGPTFSGGESYIVIEQCWQMNVMNARY
WP.096693068.1iUNK/1-631 ---------------MGRW-IVLLLVLLISLGVGYSYGVNPDVVPI-SSNDIPSSIIQRPTDKPKDKPIKIVIHDGGTFCYGPTFSGGESYIVIEQCWQMNVMNARY
WP.061236865.1iUNK/1-631 ---------------MGRW-IILMTVLLVSIGVGYSYGVNPDVVPI-SSNDIPSSIIQRPTDKPKDKPIKIVIHDGGTFCYGPTFSGGESYIVIEQCWQMNVMNARY
WP.002190754.1iUNK/1-631 ---------------MGRW-IILMTVLLVSIGVGYSYGVNPDVVPI-SSNDIPSSIIQRPTDKPKDKPIKIVIHDGGTFCYGPTFSGGESYIVIEQCWQMNVMNARY
WP.000533945.1iUNK/1-631 ---------------MGRW-IILMTVLLVSIGVGYSYGVNPDVVPI-SSNDIPSSIIQRPTDKPKDKPIKIVIHDGGTFCYGPTFSGGESYIVIEQCWQMNVMNARY
WP.000533941.1iUNK/1-631 ---------------MGRW-IILMTVLLVSIGVGYSYGVNPDVVPI-SSNDIPSSIIQRPTDKPKDKPIKIVIHDGGTFCYGPTFSGGESYIVIEQCWQMNVMNARY
PD1Q14_PD1.1435_AG3.iPD1-/1-631 ---------------MGRW-IILMTVLLVSIGVGYSYGVNPDVVPI-SSSDIPSSIIQRPTDKPKDKPIKIVIHDGGTFCYGPTFSGGESYIVIEQCWQMNVMNARY
LAI.iS56601_3354783/1-631 ---------------MGRW-IILMTVLLVSIGVGYSYGVNPDVVPI-SSNDIPSSIIQRPTDKPKDKPIKIVIHDGGTFCYGPTFSGGESYIVIEQCWQMNVMNARY
LAI.iIPAV_3351883/1-631 ---------------MGRW-IILMTVLLVSIGVGYSYGVNPDVVPI-SSNDIPSSIIQRPTDKPKDKPIKIVIHDGGTFCYGPTFSGGESYIVIEQCWQMNVMNARY
KG2Q1E_KG2.5120_AG3.uKG2-/1-631 ---------------MGRW-IILMTVLLVSIGVGYSYGVNPDVVPI-SSSDIPSSIIQRPTDKPKDKPIKIVIHDGGTFCYGPTFSGGESYIVIEQCWQMNVMNARY
AW3Q13_AW3.1316_AG10.iAW3/1-631 ---------------MGRW-IILMTVLLVSIGVGYSYGVNPDVVPI-SSNDIPSSIIQRPTDKPKDKPIKIVIHDGGTFCYGPTFSGGESYIVIEQCWQMNVMNARY
AP6Q13_AP6.1028_AG3.iAP6-/1-631 ---------------MGRW-IILMTVLLVSIGVGYSYGVNPDVVPI-SSNDIPSSIIQRPTDKPKDKPIKIVIHDGGTFCYGPTFSGGESYIVIEQCWQMNVMNARY
AP5Q13_AP5.973_AG10.iAP5-/1-631 ---------------MGRW-IILMTVLLVSIGVGYSYGVNPDVVPI-SSNDIPSSIIQRPTDKPKDKPIKIVIHDGGTFCYGPTFSGGESYIVIEQCWQMNVMNARY
WP.000533949.1iUNK/1-631 ---------------MGRW-IILMTVLLVSIGVGYSYGVNPDVVPI-SSSDIPSSIIQRPTDKPKDKPIKIVIHDGGTFCYGPTFSGGESYIVIEQCWQMNVMNARY
WP.137119404.1iUNK/1-631 ---------------MGRW-IILMTVLLVSIGVGYSYGVNPDVVPI-SSNDIPSSIIQRPTDKPKDKPIKIVIHDGGTFCYGPTFSGGESYIVIEQCWQMNVMNARY
AW1Q1B_AW1.3258_AG3.iAW1-/1-631 ---------------MGRW-IILMTVLLVSIGVGYSYGVNPDVVPI-SSNDIPSSIIQRPTDKPKDKPIKIVIHDGGTFCYGPTFSGGESYIVIEQCWQMNVMNARY
WP.137171171.1iUNK/1-631 ---------------MGRW-IVLLLVLLISFGVGYSYGVNPDVVPI-SSNDIPSSIIQRPTDKPKDKPIKIVIHDGGTFCYGPTFSGGESYIVIEQCWQMNVMNARY
RT2Q1B_RT2.3273_AG3.iRT2-/1-631 ---------------MGRW-IVLLLVLLISFGVGYSYGVNPDVVPI-SSNDIPSSIIQRPTDKPKDKPIKIVIHDGGTFCYGPTFSGGESYIVIEQCWQMNVMNARY
PN3Q1B_SLPN3.3309_AG3.iPN/1-631 ---------------MGRW-IVLLLVLLISFGVGYSYGVNPDVVPI-SSNDIPSSIIQRPTDKPKDKPIKIVIHDGGTFCYGPTFSGGESYIVIEQCWQMNVMNARY
KW2Q1B_KW2.xxxx_AG3.iKW2-/1-631 ---------------MGRW-IVLLLVLLISFGVGYSYGVNPDVVPI-SSNDIPSSIIQRPTDKPKDKPIKIVIHDGGTFCYGPTFSGGESYIVIEQCWQMNVMNARY
KW2Q1B_KW2.3225_AG3.iKW2-/1-631 ---------------MGRW-IVLLLVLLISFGVGYSYGVNPDVVPI-SSNDIPSSIIQRPTDKPKDKPIKIVIHDGGTFCYGPTFSGGESYIVIEQCWQMNVMNARY
KAA1269349.1iWHE/1-631 ---------------MGRW-IVLLLVLLISFGVGYSYGVNPDVVPI-SSNDIPSSIIQRPTDKPKDKPIKIVIHDGGTFCYGPTFSGGESYIVIEQCWQMNVMNARY
AP7Q1B_AP7.3207_AG3.iAP7-/1-631 ---------------MGRW-IVLLLVLLISFGVGYSYGVNPDVVPI-SSNDIPSSIIQRPTDKPKDKPIKIVIHDGGTFCYGPTFSGGESYIVIEQCWQMNVMNARY
AP3Q1B_AP3.3309_AG10.iAP3/1-631 ---------------MGRW-IVLLLVLLISFGVGYSYGVNPDVVPI-SSNDIPSSIIQRPTDKPKDKPIKIVIHDGGTFCYGPTFSGGESYIVIEQCWQMNVMNARY
AP1Q1A_AP1.3767_AG3.iAP1-/1-631 ---------------MGRW-IVLLLVLLISFGVGYSYGVNPDVVPI-SSNDIPSSIIQRPTDKPKDKPIKIVIHDGGTFCYGPTFSGGESYIVIEQCWQMNVMNARY
AG3Q1B_U1.3309_AG3.iO7371/1-631 ---------------MGRW-IVLLLVLLISFGVGYSYGVNPDVVPI-SSNDIPSSIIQRPTDKPKDKPIKIVIHDGGTFCYGPTFSGGESYIVIEQCWQMNVMNARY
EMM96034.1iZNI_sLT2156/1-631 ---------------MGRW-IILMTVLLVSIGVGYSYGVNPDVVPI-SSNDIPSSIIQRPTDKPKDKPIKIVIHDGGTFCYGPTFSGGESYIVIEQCWQMNVMNARY
WP.061241527.1iUNK/1-631 ---------------MSRW-IVLLLVLLISLGVGYSYGVNPDVVPI-SSNDIPSSIIQRPTDKPKDKPIKIVIHDGGTFCYGPTFSGGESYIVIEQCWQMNVMNARY
WP.002188454.1iUNK/1-631 ---------------MSRW-IVLLLVLLISLGVGYSYGVNPDVVPI-SSNDIPSSIIQRPTDKPKDKPIKIVIHDGGTFCYGPNFSGGESYIVIEQCWQMNVMNARY
WP.010679784.1iUNK/1-631 ---------------MSRW-IVLLLVLLISLGVGYSYGVNPDVVPI-SSNDIPSSIIQRPTDKPKDKPIKIVIHDGGTFCYGPTFSGGESYIVIEQCWQMNVMNARY
EMN50660.1iUNK_sL1207/1-631 ---------------MSRW-IVLLLVLLISLGVGYSYGVNPDVVPT-SSNDIPSSIIQRPTDKPKDKPIKIVIHDGGTFCYGPTFSGGESYIVIEQCWQMNVMNARY
WP.192502083.1iUNK/1-631 ---------------MGRW-IILMTVLLVSIGVGYSYGVNPDVVPI-SSNDIPSSIIQRPTDKPKDKPIKIVIHDGGTFCYGPTFSGGESYIVIEQCWQMNVMNARY
WP.002101706.1iUNK/1-631 ---------------MGRW-IVLLLVLLISLGVGYSYGVNPDVVPI-SSSDIPSSIIQRPTDKPKDKPIKIVIHDGGTFCYGPTFSGGESYIVIEQCWQMNVMNARY
BAE.iS1548_1007699/1-631 ---------------MGRW-IILMTVLLVSIGVGYSYGVNPDVVPI-SSNDIPSSIIQRPTDKPKDKPIKIVIHDGGTFCYGPTFSGGESYIVIEQCWQMNVMNARY
WP.047722115.1iUNK/1-631 ---------------MSRW-IVLLLVLLISLGVGYSYGVNPDVVPI-SSNDIPSSIIQRPTDKPKDKPIKIVIHDGGTFCYGPTFSGGESYIVIEQCWQMNVMNARY
WP.047020761.1iUNK/1-631 ---------------MGRW-IILMTVLLVSIGVGYSYGVNPDVVPI-SSNDIPSSIIQRPTDKPKDKPIKIVIHDGGTFCYGPTFSGGESYIVIEQCWQMNVMNARY
BVA.iPigK151_1044606/1-631 ---------------MGRW-IILMTVLLVSIGVGYSYGVNPDVVPI-SSNDIPSSIIQRPTDKPKDKPIKIVIHDGGTFCYGPTFSGGESYIVIEQCWQMNVMNARY
WP.061272974.1iUNK/1-631 ---------------MGRW-IVLLLVLLISLGVGYSYGVNPDVVPI-SSSNIPGSIIQRPTDKPKDKPIKVNVSSGGTFCYGPTFSGGESYIVIEQCWEQDVRNARY
WP.082283162.1iUNK/1-631 ---------------MSRW-IVLLLVLLISLGVGYSYGVNPDVVPI-SSNDIPSSIIQRPTDKPKDKPIKIVIHDGGTFCYGPNFSGGESYIVIEQCWQMNVMNARY
WP.060684620.1iUNK/1-631 ---------------MGRW-IILMTVLLVSIGVGYSYGVNPDVVPI-SSNDIPSSIIQRPTDKPKDKPIKIVIHDGGTFCYGPTFSGGESYIVIEQCWQMNVMNARY
HJO.iNorma_3517156/1-631 ---------------MGRW-IILMTVLLVSIGVGYSYGVNPDVVPI-SSNDIPSSIIQRPTDKPKDKPIKIVIHDGGTFCYGPTFSGGESYIVIEQCWQMNVMNARY
HJO.iL53_3462900/1-631 ---------------MGRW-IILMTVLLVSIGVGYSYGVNPDVVPI-SSNDIPSSIIQRPTDKPKDKPIKIVIHDGGTFCYGPTFSGGESYIVIEQCWQMNVMNARY
WP.002124047.1iUNK/1-631 ---------------MGRW-IILMTVLLVSIGVGYSYGVNPDVVPI-SSNDIPSSIIQRPTDKPKDKPIKIVIHDGGTFCYGPTFSGGESYIVIEQCWQMNVMNARY
WP.000092384.1iUNK/1-632 ---------------MSRW-IVLLLVLLISLGVGYSYGVNPDVVPI-SSNDIPSSIIQRPTDKPKDKPIKIVIHDGGTFCYGPTFSGGESYIVIEQCWQMNVMNARY
UNK.iR13L_3595405/1-632 ---------------MGRW-IVLLLVLLISFGVGYSYGVNPDVVPI-SSSDIPGSIIQRPTDKPKDKPIKVNVSSGGTFCYGPNFSGGESYIVIEQCWQMHVMNARY
UNK.iR12_3585596/1-632 ---------------MSRW-IVLLLVLLISLGVGYSYGVNPDVVPI-SSSDIPGSIIQRPTDKPKDKPIKVNVSSGGTFCYGPNFSGGESYIVIEQCWQMHVMNARY
QHH72246.1iUNK/1-632 ---------------MSRW-IVLLLVLLISLGVGYSYGVNPDVVPI-SSSDIPGSIIQRPTDKPKDKPIKVNVSSGGTFCYGPNFSGGESYIVIEQCWQMHVMNARY
QHH29538.1iUNK/1-632 ---------------MGRW-IVLLLVLLISFGVGYSYGVNPDVVPI-SSSDIPGSIIQRPTDKPKDKPIKVNVSSGGTFCYGPNFSGGESYIVIEQCWQMHVMNARY
WP.002156240.1iUNK/1-631 ---------------MSRW-IVLLLVLLISLGVGYSYGVNPDVVPI-SSNDIPSSIIQRPTDKPKDKPIKIVIHDGGTFCYGPTFSGGESYIVIEQCWQMNVMNARY
WP.002145782.1iUNK/1-631 ---------------MSRW-IVLLLVLLISLGVGYSYGVNPDVVPI-SSNDIPSSIIQRPTDKPKDKPIKIVTHDGGTFCYGPTFSGGESYIVIEQCWQMNVMNARY
WP.000092383.1iUNK/1-631 ---------------MSRW-IVLLLVLLISLGVGYSYGVNPDVVPI-SSNDIPSSIIQRPTDKPKDKPIKIVIHDGGTFCYGPTFSGGESYIVIEQCWQMNVMNARY
ICT.iS898_948409/1-631 ---------------MSRW-IVLLLVLLISLGVGYSYGVNPDVVPI-SSNDIPSSIIQRPTDKPKDKPIKIVIHDGGTFCYGPTFSGGESYIVIEQCWQMNVMNARY
COP.iSK1_948534/1-631 ---------------MSRW-IVLLLVLLISLGVGYSYGVNPDVVPI-SSNDIPSSIIQRPTDKPKDKPIKIVIHDGGTFCYGPTFSGGESYIVIEQCWQMNVMNARY
COP.iL1130_Q72U83.cl9487/1-631 ---------------MSRW-IVLLLVLLISLGVGYSYGVNPDVVPI-SSNDIPSSIIQRPTDKPKDKPIKIVIHDGGTFCYGPTFSGGESYIVIEQCWQMNVMNARY
COP.iFDAARGOS203_3238485/1-631 ---------------MSRW-IVLLLVLLISLGVGYSYGVNPDVVPI-SSNDIPSSIIQRPTDKPKDKPIKIVIHDGGTFCYGPTFSGGESYIVIEQCWQMNVMNARY
WP.000092374.1iUNK/1-631 ---------------MSRW-IVLLLVLLISLGVGYSYGVNPDVVPI-SSNDIPSSIIQRPTDKPKDKPIKIVIHDGGTFCYGPTFSGGESYIVIEQCWQMHVMNARY
WP.137108109.1iUNK/1-631 ---------------MGRW-IVLLLVLLISFGVGYSYGVNPDVVPI-SSNDIPSSIIQRPTDKPKDKPIKIVIHDGGTFCYGPTFSGGESYIVIEQCWQMNVMNARY
KW1Q13_KW1.911_AG3.iKW1-1/1-631 ---------------MGRW-IVLLLVLLISFGVGYSYGVNPDVVPI-SSNDIPSSIIQRPTDKPKDKPIKIVIHDGGTFCYGPTFSGGESYIVIEQCWQMNVMNARY
WP.137108596.1iUNK/1-631 ---------------MGRW-IVLLLVLLISFGVGYSYGVNPDVVPI-SSNDIPGSIIQRPTDKPKDKPIKIVIHDGGTFCYGPTFSGGESYIVIEQCWQMNVMNARY
RT2Q15_RT2.850_AG3.iRT2-1/1-631 ---------------MGRW-IVLLLVLLISFGVGYSYGVNPDVVPI-SSNDIPSSIIQRPTDKPKDKPIKIVIHDGGTFCYGPTFSGGESYIVIEQCWQMNVMNARY
PN3Q15_SLPN3.937_AG3.iPN3/1-631 ---------------MGRW-IVLLLVLLISFGVGYSYGVNPDVVPI-SSNDIPSSIIQRPTDKPKDKPIKIVIHDGGTFCYGPTFSGGESYIVIEQCWQMNVMNARY
KW2Q15_KW2.860_AG3.iKW2-1/1-631 ---------------MGRW-IVLLLVLLISFGVGYSYGVNPDVVPI-SSNDIPGSIIQRPTDKPKDKPIKIVIHDGGTFCYGPTFSGGESYIVIEQCWQMNVMNARY
KW2Q15_KW2.842_AG3.iKW2-1/1-631 ---------------MGRW-IVLLLVLLISFGVGYSYGVNPDVVPI-SSNDIPGSIIQRPTDKPKDKPIKIVIHDGGTFCYGPTFSGGESYIVIEQCWQMNVMNARY
KW1Q19_KW1.3296_AG3.iKW1-/1-631 ---------------MGRW-IVLLLVLLISFGVGYSYGVNPDVVPI-SSNDIPGSIIQRPTDKPKDKPIKIVIHDGGTFCYGPTFSGGESYIVIEQCWQMNVMNARY
KAA1291344.1iGRA/1-631 ---------------MGRW-IVLLLVLLISFGVGYSYGVNPDVVPI-SSNDIPSSIIQRPTDKPKDKPIKIVIHDGGTFCYGPTFSGGESYIVIEQCWQMNVMNARY
AP7Q15_AP7.843_AG3.iAP7-1/1-631 ---------------MGRW-IVLLLVLLISFGVGYSYGVNPDVVPI-SSNDIPSSIIQRPTDKPKDKPIKIVIHDGGTFCYGPTFSGGESYIVIEQCWQMNVMNARY
AP3Q15_AP3.937_AG10.iAP3-/1-631 ---------------MGRW-IVLLLVLLISFGVGYSYGVNPDVVPI-SSNDIPSSIIQRPTDKPKDKPIKIVIHDGGTFCYGPTFSGGESYIVIEQCWQMNVMNARY
AP1Q14_AP1.1372_AG3.iAP1-/1-631 ---------------MGRW-IVLLLVLLISFGVGYSYGVNPDVVPI-SSNDIPSSIIQRPTDKPKDKPIKIVIHDGGTFCYGPTFSGGESYIVIEQCWQMNVMNARY
AG3Q15_U1.937_AG3.iO73718/1-631 ---------------MGRW-IVLLLVLLISFGVGYSYGVNPDVVPI-SSNDIPSSIIQRPTDKPKDKPIKIVIHDGGTFCYGPTFSGGESYIVIEQCWQMNVMNARY
WP.061272937.1iUNK/1-634 ---------------MYKW-KVLTAFFLISIGSGFEYGVNPTHIHA-----TTYSIVQKPTDPPKDKPIKVIVSGGGKFCYGPTFSGGESYIIIEQCWQMHVKNARY
WP.061233908.1iUNK/1-634 ---------------MYKW-KVLTAFFLISIGSGFEYGVNPTHIHA-----TTYSIVQKPTDPPKDKPIKVIVSGGGKFCYGPTFSGGESYIIIEQCWQMHVKNARY
```

WP.000280011.1iUNK/1-634	---------------MYKW-KVLTAFFLISIGSGFEYGVNPTHIHA-----TTYSIVQKPTDPPKDKPIKVIVSGGGKFCYGPTFSGGESYIIIEQCWQMHVKNAR
PN2Q1C_PN2.4335_AG14.iPN2/1-634	---------------MYKW-KVLTAFFLISIGSGFEYGVNPTHIHA-----TTYSIVQKPTDPPKDKPIKVIVSGGGKFCYGPTFSGGESYIIIEQCWQMHVKNAR
WP.002184428.1iUNK/1-634	---------------MYKW-KVLTAFFLISIGSGFEYGVNPTHIHA-----TTYSIVQKPTDPPKDKPIKVIVSGGGKFCYGPTFSGGESYIIIEQCWQMHVKNAR
WP.002117330.1iUNK/1-634	---------------MYKW-KVLTAFFLISIGSGFEYGVNPTHIHA-----TTYSIVQKPTDPPKDKPIKVIVSGGGKFCYGPTFSGGESYIIIEQCWQMHVKNAR
CLA.iS611_841413/1-634	---------------MYKW-KVLTAFFLISIGSGFEYGVNPTHIHA-----TTYSIVQKPTDPPKDKPIKVIVSGGGKFCYGPTFSGGESYIIIEQCWQMHVKNAR
CLA.iS114_684764/1-634	---------------MYKW-KVLTAFFLISIGSGFEYGVNPTHIHA-----TTYSIVQKPTDPPKDKPIKVIVSGGGKFCYGPTFSGGESYIIIEQCWQMHVKNAR
CLA.iRUFN_1753985/1-634	---------------MYKW-KVLTAFFLISIGSGFEYGVNPTHIHA-----TTYSIVQKPTDPPKDKPIKVIVSGGGKFCYGPTFSGGESYIIIEQCWQMHVKNAR
CLA.iLJ178_846582/1-634	---------------MYKW-KVLTAFFLISIGSGFEYGVNPTHIHA-----TTYSIVQKPTDPPKDKPIKVIVSGGGKFCYGPTFSGGESYIIIEQCWQMHVKNAR
WP.033109407.1iUNK/1-637	---------------MYKW-KVLTAFFLISIGSGFEYGVNHTHIHA--LSKIEYSVIQKPTDPPKDKPIKVIVSDGGKFCYGPNFSGGESYIIIEQCWQMHVMNAR
EMN51001.1iUNK_sL1207/1-638	---------------MRNRKKVIIVILLVATITYLKYGIDHTHIHA--SSKIEYSVIQKPTDPPKDKPIKVIVSDGGKFCYGPNFSGGESYIIIEQCWQMHVMNAR
MAE.iUPMMCNIIDLP_3385730/1-637	---------------MYKW-KVLTAFFLISIGSGFEYGVNHTHIHA--LSKIEYSVIQKPTDPPKDKPIKVIVSDGGKFCYGPNFSGGESYIIIEQCWQMHVMNAR
MAE.iUPMMCNIIDHP_3385682/1-637	---------------MYKW-KVLTAFFLISIGSGFEYGVNHTHIHA--LSKIEYSVIQKPTDPPKDKPIKVIVSDGGKFCYGPNFSGGESYIIIEQCWQMHVMNAR
WP.010678857.1iUNK/1-637	---------------MYKW-KVLTAFFLISIGSGFEYGVNHTHIHA--LSKIEYSVIQKPTDPPKDKPIKVIVSDGGKFCYGPNFSGGESYIIIEQCWQMHVMNAR
WP.141597671.1nUNK/1-632	---------------MRCW-KVLVVVLLVSILLSFRYGINV---HA--SSKANDSIVQKPTDPPKDKPIKIVVSNKGKYCYSPVFTKGEGYVWIDTCTDK-TAKAR
WP.039939994.1nUNK/1-633	---------------MRCW-KVLVVVLLVSILLSFRYGINV---HA--SSKANDSIVQKPTDPPKDKPIQVVVSDGGKFCYGPLFSGGESYIQIQQCWEQHVKNAR
WP.004447041.1nUNK/1-636	---------------MRNWKKMLIVVLLVSIGVGFEYSINPTPVHA--SSKANDSIVQKPTDPPKDKPIKIVVSNKGKYCYSPVFTKGEGYVWIDKCTDK-TAKAR
EKR72314.1nUNK_s200600187/1-623	--------------------------MLVSILLSFRYGINV---HA--SSKANDSIVQKPTDPPKDKPIQVVVSDGGKFCYGPLFSGGESYIQIQQCWEQHVKNAR
WP.004441001.1nUNK/1-633	---------------MRCW-KVLVVVLLVSILLSFRYGINV---HA--SSKANDSIVQKPTDPPKDKPIQVVVSDGGKFCYGPLFSGGESYIQIQQCWEQHVKNAR
EMO26805.1nBAE_sHAI135/1-633	---------------MRCW-KVLVVVLLVSILLSFRYGINV---HA--SSKVNDSIVQKPTDPPKDKPIQVVVSDGGKFCYGPLFSGGESYIQIQQCWEQHVKNAR
WP.061247334.1nUNK/1-639	---------------MRKW-KVLSLVLLIFIRVEHSYGENPTFTPI--SLGVTGSIVQKPTDKPKDKPIQVVVSDGGKFCYGPIFSGGESYIIIEQCWERHVMNAR
WP.002177543.1nUNK/1-639	---------------MRKW-KVLSLVLLIFIRVEHSYGENPTFTPI--SLGVTGSIVQKPTDKPKDKSIQVVVSDGGKFCYGPIFSGGESYIIIEQCWERHVMNAR
WP.004438285.1nUNK/1-635	---------------MYNW-KVLIIIFLVSVGAILEYGINPTQIDA-SSSKVNYSIVQKPTDQPKDKAIKILVHDGGEFCYAPIFSGGESYIQIEQCWERHVMNAR
WP.002179591.1nUNK/1-638	---------------MCNWKKVLIVVLLISIGVGFEYGESHTRVHAASSSKVNYSIIQKPTNKPKDKPIKILVHDGGEFCYGPIFSGGESYIQIEQCWERHVMNAR
WP.061251431.1nUNK/1-632	---------------MCNWKKVLLAVLLISIGVGFEYGTNPTPIDA--SPNSVYSIVQKPTDPPKDKPIQVVVSDGGKFCYGPIFSGGESYIVIEQCWQMHVMNAR
WP.061232986.1nUNK/1-632	---------------MCNWKKVLLAVLLISIGVGFEYGTNPTPIDA--SPNSVYSIVQKPTDPPKDKPIQVIVSDGGKFCYAPIFSGGESYIVIEQCWQMHVKNAR
WP.061245923.1nUNK/1-640	---------------MYNWKKMLIVVLLISIGVGFEYGESHTRVHAASSSKSNYSIVQKPTDKPKDKPIQVVVSDGGKFCYGPIFSGGESYIQIQQCWERHVMKAR
WP.194099263.1kUNK/1-627	---------------MGRW-IVLRVFLLVLIGIGFEYGINHTSVNA--SSKSDYSIVQKPTDPPKDKPVQVVVSDGGKFCYGPTFSGGESYIVIEQCWQMHVMNAR
WP.082293312.1kUNK/1-627	---------------MSRW-IVLRVFLLVLIGIGFEYGINHTSVNA--SSKSDYSIVQKPTDPPKDKPVQVVVSDGGKFCYGPTFSGGESYIVIEQCWQMHVMSAR
WP.020779706.1kUNK/1-627	---------------MGRW-IVLRVFLLVLIGIGFEYGINHTSVNA--SSKSDYSIVQKPTDPPKDKPVQVVVSDGGKFCYGPTFSGGESYIVIEQCWQMHVMSAR
WP.004771042.1kUNK/1-627	---------------MGRW-IVLRVFLLVLIGIGFEYGINHTSVNA--SSKSDYSIVQKPTDQPKDKPVQVVVSDGGKFCYGPTFSGGESYIVIEQCWQMHVMSAR
WP.004759440.1kUNK/1-627	---------------MGRW-IVLRVFLLVLIGIGFEYGINHTSVNA--SSKSDYSIVQKPTDQPKDKPVQVVVSDGGKFCYGPTFSGGESYIVIEQCWQMHVMSAR
WP.004783031.1kUNK/1-627	---------------MGRW-IVLRVFLLVLIGIGFEYGINHTSVNA--SSKSDYSIVQKPTDQPKDKPVQVVVSDGGKFCYGPTFSGGESYIVIEQCWQMHVMSAR
WP.004768482.1kUNK/1-627	---------------MGRW-IVLRVFLLVLIGIGFEYGINHTSVNA--SSKSDYSIVQKPTDPPKDKPVQVVVSDGGKFCYGPTFSGGESYIVIEQCWQMHVMSAR
WP.004767582.1kUNK/1-627	---------------MGRW-IVLRIFLLVLIGIGFEYGINHTSVNA--SSKSDYSIVQKPTDQPKDKPVQVVVSDGGKFCYGPTFSGGESYIVIEQCWQMHVMSAR
WP.004761566.1kUNK/1-627	---------------MGRW-IVLRIFLLVLIGIGFEYGINHTSVNA--SSKSDYSIVQKPTDQPKDKPVQVVVSDGGKFCYGPTFSGGESYIVIEQCWQMHVMSAR
WP.004776691.1kUNK/1-627	---------------MGRW-IVLRIFLLVLIGIGFEYGINHTSVNA--SSKSDYSIVQKPTDQPKDKPVQVVVSDGGKFCYGPTFSGGESYIVIEQCWQMHVMSAR
WP.078131699.1kUNK/1-627	---------------MGRW-IVLRIFLLVLIGIGFEYGINYTSVNA--SSKSDYSIVQKPTDQPKDKPVQVVVSDGGKFCYGPTFSGGESYIVIEQCWQMHVMSAR
WP.016751771.1kUNK/1-627	---------------MGRW-IVLRVFLLVLIGIGFEYGINHTSVNA--SSKSDYSIVQKPTDQPKDKPVQVVVSDGGKFCYGPTFSGGESYIVIEQCWQMHVMSAR
WP.004755425.1kUNK/1-627	---------------MGRW-IVLRVFLLVLIGIGFEYGINHTSINA--SSKSDYSIVQKPTDQPKDKPVQVVVSDRGKFCYGPTFSGGESYIVIEQCWQMHVMSAR
EMO77486.1kUNK_s200801925/1-627	---------------MGRW-IVLRVFLLVLIGIGFEYGINHTSVNA--SSKSDYSIVQKPTDQPKDKPVQVVVSDGGKFCYGPTFSGGESYIVIEQCWQMHVMSAR
WP.016753062.1kUNK/1-627	---------------MGRW-IVLRVFLLVLIGIGFEYGINHTSVNA--SSKSDYSIVQKPTDQPKDKPVQVVVSDGGKFCYGPTFSGGESYIVIEQCWQMHVKNAR
WP.020766761.1mUNK/1-627	---------------MGRW-IVLRVFLLVLIGIGFEYGINHTSVNA--SSKSDYSIVQKPTDQPKDKPVQVVVSDGGKFCYGPTFSGGESYIVIEQCWQMHVMSAR
WP.004767106.1kUNK/1-628	---------------MGNW-KNLIVVLLVSIGVGFEYGMDHTLVHA-SSSKIDYSIVQKPSDQPKDKSIQVVVSDGGKFCYGPIFSGGESYIIIEQCWERHVKNAR
PN5K1C_PN5.3572_AG15.kPN5/1-628	---------------MGNW-KNLIVVLLVSIGVGFEYGMDHTLVHA-SSSKIDYSIVQKPSDQPKDKSIQVVVSDGGKFCYGPIFSGGESYIIIEQCWERHVKNAR
WP.053522530.1nUNK/1-635	---------------MRNWKKMLMVVLLVSIGVGFEYSINPAPVHA--SSKANNSIVQKPTDKPKDKPIQVVVSDGGKFCYGPIFSGGESYIQIEQCWERHVKNAR
WP.004422750.1nUNK/1-634	---------------MRNWKKMLIVVLLVSIGVGFEYSINPTPVHA--SSKANNSIVQKPTDPPKDKPIKVVVSNKGKYCYSPVFTKGEGYVWIDTCTDK-TAKAR
EMO29634.1nBAE_sHAI135/1-634	---------------MYNW-KVLIIIFLVSVGTILEYGINYTFVHA-SSSKVNYSIIQKPTDKPKDKPIKVVVSNKKAYCYSPAFTKGEGYVWIDKCTDK-TPKAR
WP.017215880.1nUNK/1-644	---------------MCNWKKALLVVLLVSIGVGFEYSINPTPVHA--SSKANNSIVQKPTDPPKDKPIKIVVSNKGKYCYSPVFTKGEGYVWIDTCTDK-TAKAR
WP.017214051.1nUNK/1-635	---------------MRNWKKVLIVVLLVSIGVGFEYGESHTRVHAASSSKANYSIAQKPTDPPKDKPIKVVVSNKKAYCYSPAFTNGEGYVWIDKCTDK-TPKAR
WP.033109370.1iUNK/1-638	---------------MRNWKKIFIVVLLVLIGFGLEYGINPTPVDA--SSKIEYLVIQKPTDPPKDQPIKVIVSGEGKFCYGPDFSGGESYIIIEQCWQMHVMNAR
MAE.iUPMMCNIIDLP_3172273/1-638	---------------MRNWKKIFIVVLLVLIGFGLEYGINPTPVDA--SSKIEYLVIQKPTDPPKDQPIKVIVSGEGKFCYGPDFSGGESYIIIEQCWQMHVMNAR
MAE.iUPMMCNIIDHP_3172226/1-638	---------------MRNWKKIFIVVLLVLIGFGLEYGINPTPVDA--SSKIEYLVIQKPTDPPKDQPIKVIVSGEGKFCYGPDFSGGESYIIIEQCWQMHVMNAR
PN3Q16_SLPN3.1039_AG3.iPN/1-640	---------------MCNWKKIFIVVLLVLIGFGFEYGMNPTPVDA--SSKIEYLVIQKPTDPPKDKPIKVIVSGEGKFCYGPDFSGGESYIIIEQCWQMHVMNAR
KAA1267951.1iWHE/1-640	---------------MCNWKKIFIVVLLVLIGFGFEYGMNPTPVDA--SSKIEYLVIQKPTDPPKDKPIKVIVSGEGKFCYGPDFSGGESYIIIEQCWQMHVMNAR
AP7Q16_AP7.946_AG3.iAP7-1/1-640	---------------MCNWKKIFIVVLLVLIGFGFEYGMNPTPVDA--SSKIEYLVIQKPTDPPKDKPIKVIVSGEGKFCYGPDFSGGESYIIIEQCWQMHVMNAR
AP3Q16_AP3.1039_AG10.iAP3/1-640	---------------MCNWKKIFIVVLLVLIGFGFEYGMNPTPVDA--SSKIEYLVIQKPTDPPKDKPIKVIVSGEGKFCYGPDFSGGESYIIIEQCWQMHVMNAR
AP1Q15_AP1.1477_AG3.iAP1-/1-640	---------------MCNWKKIFIVVLLVLIGFGFEYGMNPTPVDA--SSKIEYLVIQKPTDPPKDKPIKVIVSGEGKFCYGPDFSGGESYIIIEQCWQMHVMNAR
AG3Q16_U1.1039_AG3.iO7371/1-640	---------------MCNWKKIFIVVLLVLIGFGFEYGMNPTPVDA--SSKIEYLVIQKPTDPPKDKPIKVIVSGEGKFCYGPDFSGGESYIIIEQCWQMHVMNAR
WP.192503309.1iUNK/1-637	---------------MRNRKKVIIVILLVATITYLKYGIDHTHIHA--SSKIEYSVIQKPTDPPKDKPIKVIVSDGGKFCYGPNFSGGESYIIIEQCWQMHVMNAR
WP.060762755.1iUNK/1-639	---------------MYNWKKILIVVLLASIMVYLEYEMDHTLVHAASSSKTTNSIVQKPTDPPKDKPIKVNVSGGGTFCYGPNFSGGESYIIIEQCWQMHVMNAR
WP.047020784.1iUNK/1-637	---------------MRNRKKVIIVILLVATITYLKYGIDHTHIHA--SSKIEYSVIQKPTDPPKDKPIKVIVSDGGKFCYGPNFSGGESYIIIEQCWQMHVMNAR
WP.045192965.1iUNK/1-637	---------------MRNRKKVIIVILLVATITYLKYGIDHTHIHA--SSKIEYSVIQKPTDPPKDKPIKVIVSDGGKFCYGPNFSGGESYIIIEQCWQMHVMNAR
WP.033109403.1iUNK/1-637	---------------MRNRKKVIIVILLVATITYLKYGIDHTHIHA--SSKIEYSVIQKPTDPPKDKPIKVIVSDGGKFCYGPNFSGGESYIIIEQCWQMHVMNAR
WP.002120625.1iUNK/1-637	---------------MRNRKKVIIVILLVATITYLKYGIDHTHIHA--SSKIEYSVIQKPTDPPKDKPIKVIVSDGGKFCYGPNFSGGESYIIIEQCWQMHVMNAR
WP.002105407.1iUNK/1-637	---------------MRNRKKVIIVILLVATITYLKYGIDHTHIHA--SSKIEYSVIQKPTDPPKDKPIKVIVSDGGKFCYGPNFSGGESYIIIEQCWQMHVMNAR
WP.001245962.1iUNK/1-637	---------------MRNRKKVIIVILLVATITYLKYGIDHTHIHA--SSKIEYSVIQKPTDPPKDKPIKVIVSDGGKFCYGPNFSGGESYIIIEQCWQMHVMNAR
WP.000282999.1iUNK/1-639	---------------MYNWKKILIVVLLASIMVYLEYEMDHTLVHAASSSKTTNSIVQKPTDPPKDKPIKVNVSGGGTFCYGPNFSGGESYIIIEQCWQMHVMNAR
MAE.iUPMMCNIIDLP_643247/1-637	---------------MRNRKKVIIVILLVATITYLKYGIDHTHIHA--SSKIEYSVIQKPTDPPKDKPIKVIVSDGGKFCYGPNFSGGESYIIIEQCWQMHVMNAR
LHI.iS56609_3665602/1-637	---------------MRNRKKVIIVILLVATITYLKYGIDHTHIHA--SSKIEYSVIQKPTDPPKDKPIKVIVSDGGKFCYGPNFSGGESYIIIEQCWQMHVMNAR
KWV23850.1iUNK_pLA702.311/1-633	----------------------MLIVVLLASIMVYLEYEMDHTLVHAASSSKTTNSIVQKPTDPPKDKPIKVNVSGGGTFCYGPNFSGGESYIIIEQCWQMHVMNAR
ICT.iLangkawi_3681673/1-637	---------------MRNRKKVIIVILLVATITYLKYGIDHTHIHA--SSKIEYSVIQKPTDPPKDKPIKVIVSDGGKFCYGPNFSGGESYIIIEQCWQMHVMNAR
EMP07201.1iPES_s200701872/1-618	----------------------------------mKYGIDHTHIHA--SSKIEYSVIQKPTDPPKDKPIKVIVSDGGKFCYGPNFSGGESYIIIEQCWQMHVMNAR
EMN30651.1iPES_sL0374/1-636	---------------MYKW-KVLTAFFLISIGSGFEYGVNHTHIHA--LSKIEYSVIQKPTDPPKDKPIKVIVSDGGKFCYGPNFSGGESYIIIEQCWQMHVMNAR
BVA.iPigK151_3717284/1-637	---------------MRNRKKVIIVILLVATITYLKYGIDHTHIHA--SSKIEYSVIQKPTDPPKDKPIKVIVSDGGKFCYGPNFSGGESYIIIEQCWQMHVMNAR
AW3Q1A_AW3.4120_AG10.iAW3/1-637	---------------MRNRKKVIIVILLVATITYLKYGIDHTHIHA--SSKIEYSVIQKPTDPPKDKPIKVIVSDGGKFCYGPNFSGGESYIIIEQCWQMHVMNAR
AW1Q13_AW1.433_AG3.iAW1-1/1-637	---------------MRNRKKVIIVILLVATITYLKYGIDHTHIHA--SSKIEYSVIQKPTDPPKDKPIKVIVSDGGKFCYGPNFSGGESYIIIEQCWQMHVMNAR
AP6Q1A_AP6.3763_AG3.iAP6-/1-637	---------------MRNRKKVIIVILLVATITYLKYGIDHTHIHA--SSKIEYSVIQKPTDPPKDKPIKVIVSDGGKFCYGPNFSGGESYIIIEQCWQMHVMNAR
AP5Q1A_AP5.3685_AG10.iAP5/1-637	---------------MRNRKKVIIVILLVATITYLKYGIDHTHIHA--SSKIEYSVIQKPTDPPKDKPIKVIVSDGGKFCYGPNFSGGESYIIIEQCWQMHVMNAR
KG1Q1E_KG1.4938_AG14.uKG1/1-637	---------------MRNRKKVIIVILLVATITYLKYGIDHTHIHA--LSKIEYSVIQKPTDPPKDKPIKVIVSDGGKFCYGPNFSGGESYIIIEQCWQMHVMNAR
KG1Q13_KG1.36_AG14.uKG1-1/1-532	---------------myyc---
WP.002192117.1iUNK/1-637	---------------MRNRKKVTIVILLVATITYLKYGIDHTHIHA--SSKIEYSVIQKPTDPPKDKPIKVIVSDGGKFCYGPNFSGGESYIIIEQCWQMHVMNAR
WP.002190629.1iUNK/1-637	---------------MRNRKKVTIVILLVATITYLKYGIDHTHIHA--SSKIEYSVIQKPTDPPKDKPIKVIVSDGGKFCYGPNFSGGESYIIIEQCWQMHVMNAR
WP.001245947.1iUNK/1-637	---------------MRNRKKVIIVILLVATITYLKYGIDHTHIHA--LSKIEYSVIQKPTDPPKDKPIKVIVSDGGKFCYGPNFSGGESYIIIEQCWQMHVMNAR
WP.001245946.1iUNK/1-637	---------------MRNRKKVIIVILLVATITYLKYGIDHTHIHA--LSKIEYSVIQKPTDPPKDKPIKVIVSDGGKFCYGPNFSGGESYIIIEQCWQMHVMNAR
EMM94335.1iZNI_sLT2156/1-637	---------------MRNRKKVIIVILLVATITYLKYGIDHTHIHA--SSKIEYSVIQKPTDPPKDKPIKVIVSDGGKFCYGPNISGGESYIIIEQCWQMHVMNAR
WP.137170865.1iUNK/1-637	---------------MRNRKKVIIVILLVATITYLKYGIDHTHIHA--SSKIEYSVIQKPTDPPKDKPIKVIVSDGGKFCYGPNFSGGESYIIIEQCWQMHVMNAR
WP.137108638.1iUNK/1-637	---------------MRNRKKVIIVILLVATITYLKYGIDHTHIHA--SSKIEYSVIQKPTDPPKDKPIKVIVSDGGKFCYGPNFSGGESYIIIEQCWQMHVMNAR
WP.082279642.1iUNK/1-637	---------------MRNRKKVIIVILLVATITYLKYGIDHTHIHA--SSKIEYSVIQKPTDPPKDKPIKVIVSDGGKFCYGPNFSGGESYIIIEQCWQMHVMNAR
WP.004486777.1iUNK/1-637	---------------MRNRKKVIIVILLVATITYLKYGIDHTHIHA--SSKIEYSVIQKPTDPPKDKPIKVIVSDGGKFCYGPNFSGGESYIIIEQCWQMHVMNAR
WP.002189448.1iUNK/1-637	---------------MRNRKKVIIVILLVATITYLKYGIDHTHIHA--SSKIEYSVIQKPTDPPKDKPIKVIVSDGGKFCYGPNFSGGESYIIIEQCWQMHVMNAR
WP.002095551.1iUNK/1-637	---------------MRNRKKVIIVILLVATITYLKYGIDHTHIHA--SSKIEYSVIQKPTDPPKDKPIKVIVSDGGKFCYGPNFSGGESYIIIEQCWQMHVMNAR
WP.001245966.1iUNK/1-637	---------------MRNRKKVIIVILLVATITYLKYGIDHTHIHA--SSKIEYSVIQKPTDPPKDKPIKVIVSDGGKFCYGPNFSGGESYIIIEQCWQMHVMNAR
WP.001245965.1iUNK/1-637	---------------MRNRKKVIIVILLVATITYLKYGIDHTHIHA--SSKIEYSVIQKPTDPPKDKPIKVIVSDGGKFCYGPNFSGGESYIIIEQCWQMHVMNAR
WP.001245964.1iUNK/1-637	---------------MRNRKKVIIVILLVATITYLKYGIDHTHIHA--SSKIEYSVIQKPTDPPKDKPIKVIVSDGGKFCYGPNFSGGESYIIIEQCWQMHVMNAR
WP.001245959.1iUNK/1-637	---------------MRNRKKVIIVILLVATITYLKYGIDHTHIHA--SSKIEYSVIQKPTDPPKDKPIKVIVSDGGKFCYGPNFSGGESYIIIEQCWQMHVMNAR
UNK.iRCA_4017015/1-637	---------------MRNRKKVIIVNLLVATITYLKYGIDHTHIHA--SSKIEYSVILKPTEPPKDKPIKVIVSDGGKFCYGPNFSGGESYIIIEQCWQMHVMNAR
UNK.iR12_3557543/1-637	---------------MRNRKKVIIVILLVATITYLKYGIDHTHIHA--SSKIEYSVIQKPTDPPKDKPIKVIVSDGGKFCYGPNFSGGESYIIIEQCWQMHVMNAR
RT2Q13_RT2.625_AG3.iRT2-1/1-637	---------------MRNRKKVIIVILLVATITYLKYGIDHTHIHA--SSKIEYSVIQKPTDPPKDKPIKVIVSDGGKFCYGPNFSGGESYIIIEQCWQMHVMNAR
QHH72225.1iUNK/1-637	---------------MRNRKKVIIVILLVATITYLKYGIDHTHIHA--SSKIEYSVIQKPTDPPKDKPIKVIVSDGGKFCYGPNFSGGESYIIIEQCWQMHVMNAR
PN3Q13_SLPN3.724_AG3.iPN3/1-637	---------------MRNRKKVIIVILLVATITYLKYGIDHTHIHA--SSKIEYSVIQKPTDPPKDKPIKVIVSDGGKFCYGPNFSGGESYIIIEQCWQMHVMNAR
PD1Q1C_PD1.4319_AG3.iPD1-/1-637	---------------MRNRKKVIIVILLVATITYLKYGIDHTHIHA--SSKIEYSVIQKPTDPPKDKPIKVIVSDGGKFCYGPNFSGGESYIIIEQCWQMHVMNAR
LAI.iS56601_625656/1-637	---------------MRNRKKVIIVILLVATITYLKYGIDHTHIHA--SSKIEYSVIQKPTDPPKDKPIKVIVSDGGKFCYGPNFSGGESYIIIEQCWQMHVMNAR
LAI.iIPAV_625300/1-637	---------------MRNRKKVIIVILLVATITYLKYGIDHTHIHA--SSKIEYSVIQKPTDPPKDKPIKVIVSDGGKFCYGPNFSGGESYIIIEQCWQMHVMNAR
KW2Q13_KW2.635_AG3.iKW2-1/1-637	---------------MRNRKKVIIVILLVATITYLKYGIDHTHIHA--SSKIEYSVIQKPTDPPKDKPIKVIVSDGGKFCYGPNFSGGESYIIIEQCWQMHVMNAR
KW2Q13_KW2.621_AG3.iKW2-1/1-637	---------------MRNRKKVIIVILLVATITYLKYGIDHTHIHA--SSKIEYSVIQKPTDPPKDKPIKVIVSDGGKFCYGPNFSGGESYIIIEQCWQMHVMNAR
KW1Q1B_KW1.3506_AG3.iKW1-/1-637	---------------MRNRKKVIIVILLVATITYLKYGIDHTHIHA--SSKIEYSVIQKPTDPPKDKPIKVIVSDGGKFCYGPNFSGGESYIIIEQCWQMHVMNAR

KW1Q1B_KW1.3366_AG3.iKW1-/1-637	[illegible]
KG2Q13_KG2.1493_AG3.uKG2-/1-637	---------------MRNRKKVIIVILLVATITYLKYGIDHTHIHA--SSKIEYSVIQKPTDPPKDKPIKVIVSDGGKFCYGPNFSGGESYIIIEQCWQMHVMNARY
KAA1268179.1iWHE/1-637	---------------MRNRKKVIIVILLVATITYLKYGIDHTHIHA--SSKIEYSVIQKPTDPPKDKPIKVIVSDGGKFCYGPNFSGGESYIIIEQCWQMHVMNARY
ICT.iS898_3601803/1-637	---------------MRNRKKVIIVILLVATITYLKYGIDHTHIHA--SSKIEYSVIQKPTDPPKDKPIKVIVSDGGKFCYGPNFSGGESYIIIEQCWQMHVMNARY
HJO.iNorma_3733676/1-637	---------------MRNRKKVIIVILLVATITYLKYGIDHTHIHA--SSKIEYSVIQKPTDPPKDKPIKVIVSDGGKFCYGPNFSGGESYIIIEQCWQMHVMNARY
HJO.iL53_3679409/1-637	---------------MRNRKKVIIVILLVATITYLKYGIDHTHIHA--SSKIEYSVIQKPTDPPKDKPIKVIVSDGGKFCYGPNFSGGESYIIIEQCWQMHVMNARY
HJO.iHjtno_3660743/1-637	---------------MRNRKKVIIVILLVATITYLKYGIDHTHIHA--SSKIEYSVIQKPTDPPKDKPIKVIVSDGGKFCYGPNFSGGESYIIIEQCWQMHVMNARY
COP.iSK1_3601831/1-637	---------------MRNRKKVIIVILLVATITYLKYGIDHTHIHA--SSKIEYSVIQKPTDPPKDKPIKVIVSDGGKFCYGPNFSGGESYIIIEQCWQMHVMNARY
COP.iL1130_Q72N74.cl3598/1-637	---------------MRNRKKVIIVILLVATITYLKYGIDHTHIHA--SSKIEYSVIQKPTDPPKDKPIKVIVSDGGKFCYGPNFSGGESYIIIEQCWQMHVMNARY
COP.iFDAARGOS203_1611388/1-637	---------------MRNRKKVIIVILLVATITYLKYGIDHTHIHA--SSKIEYSVIQKPTDPPKDKPIKVIVSDGGKFCYGPNFSGGESYIIIEQCWQMHVMNARY
CLA.iS782_3875804/1-637	---------------MRNRKKVIIVILLVATITYLKYGIDHTHIHA--SSKIEYSVIQKPTDPPKDKPIKVIVSDGGKFCYGPNFSGGESYIIIEQCWQMHVMNARY
ASP42827.1iUNK_pAMR47.189/1-637	---------------MRNRKKVIIVNLLVATITYLKYGIDHTHIHA--SSKIEYSVILKPTEPPKDKPIKVIVSDGGKFCYGPNFSGGESYIIIEQCWQMHVMNARY
AP7Q13_AP7.620_AG3.iAP7-1/1-637	---------------MRNRKKVIIVILLVATITYLKYGIDHTHIHA--SSKIEYSVIQKPTDPPKDKPIKVIVSDGGKFCYGPNFSGGESYIIIEQCWQMHVMNARY
AP3Q13_AP3.724_AG10.iAP3-/1-637	---------------MRNRKKVIIVILLVATITYLKYGIDHTHIHA--SSKIEYSVIQKPTDPPKDKPIKVIVSDGGKFCYGPNFSGGESYIIIEQCWQMHVMNARY
AP1Q13_AP1.1146_AG3.iAP1-/1-637	---------------MRNRKKVIIVILLVATITYLKYGIDHTHIHA--SSKIEYSVIQKPTDPPKDKPIKVIVSDGGKFCYGPNFSGGESYIIIEQCWQMHVMNARY
AG3Q13_U1.724_AG3.iO73718/1-637	---------------MRNRKKVIIVILLVATITYLKYGIDHTHIHA--SSKIEYSVIQKPTDPPKDKPIKVIVSDGGKFCYGPNFSGGESYIIIEQCWQMHVMNARY
WP.017861134.1iUNK/1-636	---------------MRHS-IVFIVVLLVLVTTSFEYSINNIHIHA--SSNTSNSIIQKPTDQPKDKPIKIVIHDGGKFCYGPVFSGGESYIIIEQCWQMHVMNARY
WP.082271806.1iUNK/1-637	---------------MRNRKKVTIVILLVATITYLKYGIDHTHIHA--SSKIEYSVIQKPTDPPKDKPIKVIVSDGGKFCYGPNFSGGESYIIIEQCWQMHVMNARY
WP.002183516.1iUNK/1-637	---------------MRNRKKVIIVILLVATITYLKYGIDHTHIHA--SSKIEYSVIQKPTDPPKDKPIKVIVSDGGKFCYGPNFSGGESYIIIEQCWQMHVMNARY
WP.002107486.1iUNK/1-637	---------------MRNRKKVTIVILLVATITYLKYGIDHTHIHA--SSKIEYSVIQKPTDPPKDKPIKVIVSDGGKFCYGPNFSGGESYIIIEQCWQMHVMNARY
WP.002100016.1iUNK/1-631	---------------------mFIVILLVLIGFGFEYGMNPTLVDA--SSRIEYSVIQKPTDPPKDKPIKVIVSDGGKFCYGPNFSGGESYIIIEQCWQMHVMNARY
WP.001245973.1iUNK/1-637	---------------MRNRKKVTIVILLVATITYLKYGIDHTHIHA--SSKIEYSVIQKPTDPPKDKPIKVIVSDGGKFCYGPNFSGGESYIIIEQCWQMHVMNARY
PN2Q13_PN2.1596_AG14.iPN2/1-637	---------------MRNRKKVTIVILLVATITYLKYGIDHTHIHA--SSKIEYSVIQKPTDPPKDKPIKVIVSDGGKFCYGPNFSGGESYIIIEQCWQMHVMNARY
CLA.iS611_3581990/1-637	---------------MRNRKKVTIVILLVATITYLKYGIDHTHIHA--SSKIEYSVIQKPTDPPKDKPIKVIVSDGGKFCYGPNFSGGESYIIIEQCWQMHVMNARY
CLA.iS114_2606101/1-637	---------------MRNRKKVTIVILLVATITYLKYGIDHTHIHA--SSKIEYSVIQKPTDPPKDKPIKVIVSDGGKFCYGPNFSGGESYIIIEQCWQMHVMNARY
CLA.iRUFN_3111968/1-637	---------------MRNRKKVTIVILLVATITYLKYGIDHTHIHA--SSKIEYSVIQKPTDPPKDKPIKVIVSDGGKFCYGPNFSGGESYIIIEQCWQMHVMNARY
CLA.iLJ178_3586981/1-637	---------------MRNRKKVTIVILLVATITYLKYGIDHTHIHA--SSKIEYSVIQKPTDPPKDKPIKVIVSDGGKFCYGPNFSGGESYIIIEQCWQMHVMNARY
ASV06528.1iCLA_pB2G47.120/1-637	---------------MRNRKKVTIVILLVATITYLKYGIDHTHIHA--SSKIEYSVIQKPTDPPKDKPIKVIVSDGGKFCYGPNFSGGESYIIIEQCWQMHVMNARY
WP.001245972.1iUNK/1-637	---------------MRNRKKVTIVILLVATITYLKYGIDHTHIHA--SSKIEYSVIQKPTDPPKDKPIKVIVSDGGKFCYGPNFSGGESYIIIEQCWQMHVMNARY
ICT.iPiscina_3555278/1-637	---------------MRNRKKVIIVILLVATITYLKYGIDHTHIHA--SSKIEYSVIQKPTDPPKDKPIKVIVSDGGKFCYGPNFSGGESYIIIEQCWQMHVMNARY
APH42736.1iICT/1-637	---------------MRNRKKVIIVILLVATITYLKYGIDHTHIHA--SSKIEYSVIQKPTDPPKDKPIKVIVSDGGKFCYGPNFSGGESYIIIEQCWQMHVMNARY
WP.192505524.1iUNK/1-639	---------------MYKW-KVLTAFFLISIGSGFEYGVNHTHIHA--LSKIEYSVIQKPTDPPKDKPIKVIVSDGGKFCYGPNFSGGESYIIIEQCWQMHVMNARY
WP.192504923.1iUNK/1-639	---------------MYKW-KVLTAFFLISIGSGFEYGVNHTHIHA--LSKIEYSVIQKPTDPPKDKPIKVIVSDGGKFCYGPNFSGGESYIIIEQCWQMHVMNARY
WP.192486191.1iUNK/1-639	---------------MYKW-KVLTAFFLISIGSGFEYGVNHTHIHA--LSKIEYSVIQKPTDPPKDKPIKVIVSDGGKFCYGPNFSGGESYIIIEQCWQMHVMNARY
WP.137119406.1iUNK/1-639	---------------MYKW-KVLTAFFLISIGSGFEYGVNHTHIHA--LSKIEYSVIQKPTDPPKDKPIKVIVSDGGKFCYGPDFSGGESYIIIEQCWQMHVMNARY
WP.020198506.1iUNK/1-632	-----------------------MIVLLVLIGFGFEYGMNPTPVDA--SSKIEYLVIQKPTDPPKDKPIKVIVSDGGKFCYGPNFSGGESYIIIEQCWQMHVMNARY
WP.002968844.1iUNK/1-640	---------------MRNRKKVIIVILLVATITYLKYGIDHTHIHA--SSKIEYSVIQKPTDPPKDKPIKVIVSDGGKFCYGPNFSGGESYIIIEQCWQMHVMNARY
WP.002182898.1iUNK/1-639	---------------MYKW-KVLTAFFLISIGSGFEYGVNHTHIHA--LSKIEYSVIQKPTDPPKDKPIKVIVSDGGKFCYGPNFSGGESYIIIEQCWQMHVMNARY
WP.002125111.1iUNK/1-639	---------------MYKW-KVLTAFFLISIGSGFEYGVNHTHIHA--LSKIEYSVIQKPTDPPKDKPIKVIVSDGGKFCYGPNFSGGESYIIIEQCWQMHVMNARY
WP.002101726.1iUNK/1-639	---------------MYKW-KVLTAFFLISIGSGFEYGVNHTHIHA--LSKIEYSVIQKPTDPPKDKPIKVIVSDGGKFCYGPNFSGGESYIIIEQCWQMHVMNARY
WP.002094997.1iUNK/1-639	---------------MYKW-KVLTAFFLISIGSGFEYGVNHTHIHA--LSKIEYSVIQKPTDPPKDQPIKVIVSGGGKFCYGPNFSGGESYIIIEQCWQMHVMNARY
WP.002079971.1iUNK/1-639	---------------MYKW-KVLTAFFLISIGSGFEYGVNHTHIHA--LSKIEYSVIQKPTDPPKDKPIKVIVSDGGKFCYGPNFSGGESYIIIEQCWQMHVMNARY
WP.002070260.1iUNK/1-639	---------------MYKW-KVLTAFFLISIGSGFEYGVNHTHIHA--LSKIEYSVIQKPTDPPKDKPIKVIVSDGGKFCYGPNFSGGESYIIIEQCWQMHVMNARY
WP.000280006.1iUNK/1-639	---------------MYKW-KVLTAFFLISIGSGFEYGVNHTHIHA--LSKIEYSVIQKPTDPPKDKPIKVIVSDGGKFCYGPNFSGGESYIIIEQCWQMHVMNARY
WP.000280002.1iUNK/1-639	---------------MYKW-KVLTAFFLISIGSGFEYGVNHTHIHA--LSKIEYSVIQKPTDPPKDKPIKVIVSDGGKFCYGPNFSGGESYIIIEQCWQMHVMNARY
LHI.iS56609_835199/1-639	---------------MYKW-KVLTAFFLISIGSGFEYGVNHTHIHA--LSKIEYSVIQKPTDPPKDKPIKVIVSDGGKFCYGPNFSGGESYIIIEQCWQMHVMNARY
LAI.iS56601_3460643/1-639	---------------MYKW-KVLTAFFLISIGSGFEYGVNHTHIHA--LSKIEYSVIQKPTDPPKDKPIKVIVSDGGKFCYGPNFSGGESYIIIEQCWQMHVMNARY
LAI.iIPAV_3458690/1-639	---------------MYKW-KVLTAFFLISIGSGFEYGVNHTHIHA--LSKIEYSVIQKPTDPPKDKPIKVIVSDGGKFCYGPNFSGGESYIIIEQCWQMHVMNARY
ICT.iS898_843402/1-639	---------------MYKW-KVLTAFFLISIGSGFEYGVNHTHIHA--LSKIEYSVIQKPTDPPKDKPIKVIVSDGGKFCYGPNFSGGESYIIIEQCWQMHVMNARY
HJO.iNorma_3599050/1-639	---------------MYKW-KVLTAFFLISIGSGFEYGVNHTHIHA--LSKIEYSVIQKPTDPPKDKPIKVIVSDGGKFCYGPNFSGGESYIIIEQCWQMHVMNARY
HJO.iL53_3544786/1-639	---------------MYKW-KVLTAFFLISIGSGFEYGVNHTHIHA--LSKIEYSVIQKPTDPPKDKPIKVIVSDGGKFCYGPNFSGGESYIIIEQCWQMHVMNARY
COP.iSK1_843528/1-639	---------------MYKW-KVLTAFFLISIGSGFEYGVNHTHIHA--LSKIEYSVIQKPTDPPKDKPIKVIVSDGGKFCYGPNFSGGESYIIIEQCWQMHVMNARY
COP.iL1130_Q72UG2.cl8437/1-639	---------------MYKW-KVLTAFFLISIGSGFEYGVNHTHIHA--LSKIEYSVIQKPTDPPKDKPIKVIVSDGGKFCYGPNFSGGESYIIIEQCWQMHVMNARY
COP.iFDAARGOS203_3133478/1-639	---------------MYKW-KVLTAFFLISIGSGFEYGVNHTHIHA--LSKIEYSVIQKPTDPPKDKPIKVIVSDGGKFCYGPNFSGGESYIIIEQCWQMHVMNARY
CLA.iS782_811699/1-639	---------------MYKW-KVLTAFFLISIGSGFEYGVNHTHIHA--LSKIEYSVIQKPTDPPKDKPIKVIVSDGGKFCYGPNFSGGESYIIIEQCWQMHVMNARY
BAE.iS1489_842097/1-639	---------------MYKW-KVLTAFFLISIGSGFEYGVNHTHIHA--LSKIEYSVIQKPTDPPKDKPIKVIVSDGGKFCYGPNFSGGESYIIIEQCWQMHVMNARY
AW1Q1C_AW1.3361_AG3.iAW1-/1-639	---------------MYKW-KVLTAFFLISIGSGFEYGVNHTHIHA--LSKIEYSVIQKPTDPPKDKPIKVIVSDGGKFCYGPDFSGGESYIIIEQCWQMHVMNARY
AP6Q12_AP6.872_AG3.iAP6-1/1-639	---------------MYKW-KVLTAFFLISIGSGFEYGVNHTHIHA--LSKIEYSVIQKPTDPPKDKPIKVIVSDGGKFCYGPNFSGGESYIIIEQCWQMHVMNARY
AP5Q12_AP5.817_AG10.iAP5-/1-639	---------------MYKW-KVLTAFFLISIGSGFEYGVNHTHIHA--LSKIEYSVIQKPTDPPKDKPIKVIVSDGGKFCYGPNFSGGESYIIIEQCWQMHVMNARY
ASP41605.1iUNK_pAMR47.061/1-627	----------------------------mSIGSGFEYGVNHTHIHA--LSKIEYSVIQNPTDPPKDKPIKVIVSDGGKFCYGPNFSGGESYIIIEQCWQMHVMNARY
WP.175270795.1iUNK/1-639	---------------MYKW-KVLTAFFLISIGSGFEYGVNHTHIHA--LSKIEYSVIQKPTDPPKDKPIKVIVSDGGKFCYGPNFSGGESYIIIEQCWQMHVMNARY
WP.137108093.1iUNK/1-639	---------------MYKW-KVLTAFFLISIGSGFEYGVNHTHIHA--LSKIEYSVIQKPTDPPKDKPIKVIVSDGGKFCYGPDFSGGESYIIIEQCWQMHVMNARY
WP.002145818.1iUNK/1-639	---------------MYKW-KVLTAFFLISIGSGFEYGVNHTHIHA--LSKIEYSVIQKPTDPPKDKPIKVIVSDGGKFCYGPNFSGGESYIIIEQCWQMHVMNARY
WP.000534021.1iUNK/1-638	---------------MGRW-IVLRVSLLVLIGIGFEYGINHTSINA--SSKSDYSIAQKPADQPKDKSIQVVMHGGSNYCYSPVFTKGEGYIWIDYCSDN-TAKARY
UNK.iR12_833662/1-639	---------------MYKW-KVLTAFFLISIGSGFEYGVNHTHIHA--LSKIEYSVIQKPTDPPKDKPIKVIVSDGGKFCYGPNFSGGESYIIIEQCWQMHVMNARY
RT2Q1C_RT2.3374_AG3.iRT2-/1-639	---------------MYKW-KVLTAFFLISIGSGFEYGVNHTHIHA--LSKIEYSVIQKPTDPPKDKPIKVIVSDGGKFCYGPDFSGGESYIIIEQCWQMHVMNARY
QHH70178.1iUNK/1-639	---------------MYKW-KVLTAFFLISIGSGFEYGVNHTHIHA--LSKIEYSVIQKPTDPPKDKPIKVIVSDGGKFCYGPNFSGGESYIIIEQCWQMHVMNARY
PN3Q1C_SLPN3.3405_AG3.iPN/1-639	---------------MYKW-KVLTAFFLISIGSGFEYGVNHTHIHA--LSKIEYSVIQKPTDPPKDKPIKVIVSDGGKFCYGPDFSGGESYIIIEQCWQMHVMNARY
KW2Q1C_KW2.3337_AG3.iKW2-/1-646	---------------MYKW-KVLTAFFLISIGSGFEYGVNHTHIHA--LSKIEYSVIQKPTDPPKDKPIKVIVSDGGKFCYGPDFSGGESYIIIEQCWQMHVMNARY
KW2Q1C_KW2.3320_AG3.iKW2-/1-639	---------------MYKW-KVLTAFFLISIGSGFEYGVNHTHIHA--LSKIEYSVIQKPTDPPKDKPIKVIVSDGGKFCYGPDFSGGESYIIIEQCWQMHVMNARY
KW1Q12_KW1.817_AG3.iKW1-1/1-639	---------------MYKW-KVLTAFFLISIGSGFEYGVNHTHIHA--LSKIEYSVIQKPTDPPKDKPIKVIVSDGGKFCYGPDFSGGESYIIIEQCWQMHVMNARY
AP7Q1C_AP7.3299_AG3.iAP7-/1-639	---------------MYKW-KVLTAFFLISIGSGFEYGVNHTHIHA--LSKIEYSVIQKPTDPPKDKPIKVIVSDGGKFCYGPDFSGGESYIIIEQCWQMHVMNARY
AP3Q1C_AP3.3405_AG10.iAP3/1-639	---------------MYKW-KVLTAFFLISIGSGFEYGVNHTHIHA--LSKIEYSVIQKPTDPPKDKPIKVIVSDGGKFCYGPDFSGGESYIIIEQCWQMHVMNARY
AP1Q1B_AP1.3869_AG3.iAP1-/1-639	---------------MYKW-KVLTAFFLISIGSGFEYGVNHTHIHA--LSKIEYSVIQKPTDPPKDKPIKVIVSDGGKFCYGPDFSGGESYIIIEQCWQMHVMNARY
AG3Q1C_U1.3405_AG3.iO7371/1-639	---------------MYKW-KVLTAFFLISIGSGFEYGVNHTHIHA--LSKIEYSVIQKPTDPPKDKPIKVIVSDGGKFCYGPDFSGGESYIIIEQCWQMHVMNARY
WP.096637693.1iUNK/1-637	---------------MYKW-KVLTAFFLISIGSGFEYGVNHTHIHA--LSKIEYSVIQKPTDPPKDKPIKVIVSGEGKFCYGPNFSGGESYIIIEQCWQMHVMNARY
WP.064196009.1iUNK/1-637	---------------MYKW-KVLTAFFLISIGSGFEYGVNHTHIHA--LSKIEYSVIQKPTDPPKDKPIKVIVSDGGKFCYGPNFSGGESYIIIEQCWQMHVMNARY
WP.047020759.1iUNK/1-637	---------------MYKW-KVLTAFFLISIGSGFEYGVNHTHIHA--LSKIEYSVIQKPTDPPKDKPIKVIVSDGGKFCYGPNFSGGESYIIIEQCWQMHVMNARY
WP.025184538.1iUNK/1-638	---------------MRNWKKVSMIVLLVLIGFGLEYGINPTPVDA--SSKIEYSVIQKPTDPPKDQPIKVIVSGEGKFCYGPDFSGGESYIIIEQCWQMHVMNARY
WP.002127324.1iUNK/1-632	---------------m------LTAFFLISIGSGFEYGVNHTHIHA--LSKIEYSVIQKPTDPPKDKPIKVIVSDGGKFCYGPNFSGGESYIIIEQCWQMHVMNARY
WP.002109834.1iUNK/1-637	---------------MYKW-KVLTAFFLISIGSGFEYGVNHTHIHA--LSKIEYSVIQKPTDPPKDKPIKVIVSGEGKFCYGPNFSGGESYIIIEQCWQMHVMNARY
WP.002109301.1iUNK/1-637	---------------MYKW-KVLTAFFLISIGSGFEYGVNHTHIHA--LSKIEYSVIQKPTDPPKDKPIKVIVSDGGKFCYGPNFSGGESYIIIEQCWQMHVMNARY
WP.002073548.1iUNK/1-637	---------------MYKW-KVLTAFFLISIGSGFEYGVNHTHIHA--LSKIEYSVIQKPTDPPKDKPIKVIVSDGGKFCYGPNFSGGESYIIIEQCWQMHVMNARY
WP.000280005.1iUNK/1-637	---------------MYKW-KVLTAFFLISIGSGFEYGVNHTHIHA--LSKIEYSVIQKPTDPPKDKPIKVIVSDGGKFCYGPNFSGGESYIIIEQCWQMHVMNARY
KG1Q15_KG1.1967_AG14.uKG1/1-637	---------------MYKW-KVLTAFFLISIGSGFEYGVNHTHIHA--LSKIEYSVIQKPTDPPKDKPIKVIVSDGGKFCYGPNFSGGESYIIIEQCWQMHVMNARY
ICT.iLangkawi_859393/1-637	---------------MYKW-KVLTAFFLISIGSGFEYGVNHTHIHA--LSKIEYSVIQKPTDPPKDKPIKVIVSDGGKFCYGPNFSGGESYIIIEQCWQMHVMNARY
EMN09560.1kMEN_sBrem129/1-630	-----------------------MIVLLVLIGFGLEYGINPTPVDA--SSKIEYSVIQKPTDPPKDQPIKVIVSGEGKFCYGPDFSGGESYIIIEQCWQMHVMNARY
EMM81809.1iUNK_s200600185/1-634	---------------mesv----nSIFLISIGSGFEYGVNHTHIHA--LSKIEYSVIQKPTDPPKDKPIKVIVSDGGKFCYGPNFSGGESYIIIEQCWQMHVMNARY
BVA.iPigK151_939824/1-637	---------------MYKW-KVLTAFFLISIGSGFEYGVNHTHIHA--LSKIEYSVIQKPTDPPKDKPIKVIVSDGGKFCYGPNFSGGESYIIIEQCWQMHVMNARY
BAE.iS1548_903071/1-637	---------------MYKW-KVLTAFFLISIGSGFEYGVNHTHIHA--LSKIEYSVIQKPTDPPKDKPIKVIVSDGGKFCYGPNFSGGESYIIIEQCWQMHVMNARY
AW3Q12_AW3.1159_AG10.iAW3/1-556	---MHVMNARY
EMM96256.1iZNI_sLT2156/1-637	---------------MYKW-KVLTAFFLISIGSGFEYGVNHTHIHA--LSKIEYSVIQKPTDPPKDKPIKVIVSDGGKFCYGPNFSGGESYIIIEQCWQMHVMNARY
EMY03462.1iUNK_s200200062/1-637	---------------MYKW-KVLTAFFLISIGSGFEYGVNHTHIHA--LSKIEYSVIQKPTDPPKDKPIKVIVSDGGKFCYGPNFSGGESYIIIEQCWQMHVMTARY
WP.001245967.1iUNK/1-635	---------------MRNRKKVIIVILLVATITYLKYGIDHTHIHA--SSKIEYSVIQKPTDPPKDKPIKVIVSDGGKFCYGPNFSGGESYIIIEQCWQMHVMNARY
WP.141607948.1iUNK/1-631	---------------MYKW-KVLTAFFLISIGSGFEYGVNPTHIHA----TTYSIVQKPTDPPKDKPIKVIVSGGGKFCYGPTFSGGESYIIIEQCWQMHVKNARY
WP.033109365.1iUNK/1-639	---------------MRHS-IVFIVVLLVLVITSFEYGIDHTHIHA--SSKIEYLVIQKPTDPPKDKPIKVIVSDGGKFCYGPTFSGGESYIIIEQCWQMHVMNARY
MAE.iUPMMCNIIDLP_789627/1-639	---------------MRHS-IVFIVVLLVLVITSFEYGIDHTHIHA--SSKIEYLVIQKPTDPPKDKPIKVIVSDGGKFCYGPTFSGGESYIIIEQCWQMHVMNARY
MAE.iUPMMCNIIDHP_789628/1-639	---------------MRHS-IVFIVVLLVLVITSFEYGIDHTHIHA--SSKIEYLVIQKPTDPPKDKPIKVIVSDGGKFCYGPTFSGGESYIIIEQCWQMHVMNARY
WP.053388493.1kUNK/1-637	---------------MKRS-IVLVIVLLVFVAVSFEYIVNHTHIHA--SSKTVDSIIQKPTDXPKDKPIKVNVSGGGTFCYGPTFSGGESYIVIEQCWQMHVKNARY
WP.047668004.1kUNK/1-637	---------------MKRS-IVLVIVLLVFVAVSFEYIVNHTHIHA--SSKTVDSIIQKPTDSPKDKPIKVNVSGGGTFCYGPTFSGGESYIVIEQCWQMHVKNARY
WP.082292794.1kUNK/1-638	---------------MYNWKKIFIVALLVSIMIYLEHEMDHTLVHASSSLKTTDSIIQKPTDPPKDKPIKVNVSGGGTFCYGPTFSGGESYIIIEQCWQMHVMNARY
WP.082293152.1kUNK/1-637	---------------MYNWKKIFILVLLVSIMIYLEHEMDHTLVHASSSSKTTDSIIQKPTDPPKDKPIKVNVSGGGTFCYGPTFSGGESYIIIEQCWQMHVMNARY
WP.061526998.1kUNK/1-636	---------------MRNW-KVLILILLVSIVVGFEYGINYTFIHASSSSKTTDSIIQKPTDPPKDKPIKVNVSGGGTFCYGPTFSGGESYIIIEQCWQMHVMNARY
WP.196332967.1kUNK/1-638	---------------MXNWKKIFIVVLLXSIMIYLEYEXDHTLVHASSYSKTTDSIIQKPTDPPKDKPIKVNVSGGGTFCYGPTFSGGESYIIIEQCWQMHVMNARY
KPZ77825.1kMOK_pAPS47.090/1-638	---------------MQNWKKIFIVVLLVSIMIYLEYEIDHTLVHASSYSKTTDSIIQKPTDPPKDKPIKVNVSGGGTFCYGPTFSGGESYIIIEQCWQMHVMNARY
WP.162129208.1kUNK/1-637	---------------MYNWKKIFIVALLVSIMIYLEYEMDHTLVHASSYSKTTNSIIQKPTDPPKDKPIKVNVSGGGTFCYGPTFSGGESYIVIEQCWQMHVKNARY

```
WP.057162288.1kUNK/1-637              ---------------MYNWKKIFIVALLVSIMIYLEYEMDHTLVHASSYSKTTNSIIQKPTDPPKDKPIKVNVSGGGTFCYGPTFSGGESYIVIEQCWQMHVKNAR
WP.004760627.1kUNK/1-637              ---------------MYNWKKIFIVALLVSIMIYLEYEMDHTLVHASSYSKTTNSIIQKPTDPPKDKPIKVNVSGGGTFCYGPTFSGGESYIVIEQCWQMHVKNAR
WP.004752888.1kUNK/1-636              ---------------MRNW-KVLILILLVSIVVGFEYGINYTLVHASSYSKTTDSIIQKPTDPPKDKPIKVNVSGGGTFCYGPTFSGGESYIIIEQCQQMHVMNAR
WP.016560558.1kUNK/1-637              ---------------MYNWKKIFIVALLVSIMIYLEYEMDHTLVHASSYSKTTNSIIQKPTDPPKDKPIKVNVSGGGTFCYGPTFSGGESYIVIEQCWQMHVKNAR
WP.004782420.1kUNK/1-637              ---------------MYNWKKIFIVALLVSIMIYLEYEMDHTLVHASSYSKTTNSIIQKPTDPPKDKPIKVNVSGGGTFCYGPTFSGGESYIVIEQCWQMHVKNAR
WP.004777970.1kUNK/1-637              ---------------MYNWKKIFIVALLVSIMIYLEYEMDHTLVHASSYSKTTNSIIQKPTDPPKDKPIKVNVSGGGTFCYGPTFSGGESYIVIEQCWQMHVKNAR
WP.004762443.1kUNK/1-637              ---------------MYNWKKIFIVALLVSIMIYLEYEMDHTLVHASSYSKTTNSIIQKPTDPPKDKPIKVNVSGGGTFCYGPTFSGGESYIVIEQCWQMHVKNAR
WP.082293464.1kUNK/1-637              ---------------MYNWKKIFIVALLVSIMIYLEHEMDHTFIHASSSSKTTDSIIQKPTDPPKDKPIKVNVSGGGTFCYGPTFSGGESYIIIEQCWQMHVKNAR
WP.061526950.1kUNK/1-637              ---------------MYNWKKIFIVALLVSIMIYLEHEMDHTLVHASSSSKTTDSIIQKPTDPPKDKPIKVNVSGGGTFCYGPTFSGGESYIIIEQCWQMHVMNAR
WP.004776825.1kUNK/1-635              ---------------MRNWKKVLIVVLLVSIGLGFEYGMNPTPVDA-SSSQANYSIVQKPTDPPKDKPIKVNVSGGGTFCYGPTFSGGESYIIIEQCWEMHVKNAR
WP.025180417.1kUNK/1-640              ---------------MYNWKKIFIVVLLISIMIYLEYEMDHTFVHASSSSKTVDSIIQKPTDPPKDKPIKVNVSGGGTFCYGPTFSGGESYIVIEQCWQMHVKNAR
WP.004760055.1kUNK/1-637              ---------------MKRS-IVLVIVLLVFVAVSFEYIVNHTHIHA--SSKTVDSIIQKPTDSPKDKPIKVNVSGGGTFCYGPTFSGGESYIVIEQCWQMHVKNAR
WP.004750987.1kUNK/1-637              ---------------MKRS-IVLVIVLLVFVAVSFEYIVNHTHIHA--SSKTVDSIIQKPTDSPKDKPIKVNVSGGGTFCYGPTFSGGESYIVIEQCWQMHVKNAR
WP.004768516.1kUNK/1-637              ---------------MKRS-IVLVIVLLVFVAVSFEYIVNHTHIHA--SSKTVDSIIQKPTDSPKDKPIKVNVSGGGTFCYGPTFSGGESYIVIEQCWQMHVKNAR
WP.016560561.1kUNK/1-637              ---------------MKRS-IVLVIVLLVFVAVSFEYIVNHTHIHA--SSKTVDSIIQKPTDSPKDKPIKVNVSGGGTFCYGPTFSGGESYIVIEQCWQMHVKNAR
WP.004770409.1kUNK/1-637              ---------------MKRS-IVLVIVLLVFVAVSFEYIVNHTHIHA--SSKTVDSIIQKPTDSPKDKPIKVNVSGGGTFCYGPTFSGGESYIIIEQCWQMHVKNAR
WP.004780769.1kUNK/1-637              ---------------MKRS-IVLVIVLLVFVAVSFEYIVNHTHIHA--SSKTVDSIIQKPTDSPKDKPIKVNVSGGGTFCYGPTFSGGESYIVIEQCWQMHVKNAR
WP.004762584.1kUNK/1-637              ---------------MKRS-IVLVIVLLVFVAVSFEYIVNHTHIHA--SSKTVDSIIQKPTDSPKDKPIKVNVSGGGTFCYGPTFSGGESYIIIEQCWQMHVKNAR
WP.004758541.1kUNK/1-637              ---------------MKRS-IVLVIVLLVFVAVSFEYIVNHTHIHA--SSKTVDSIIQKPTDPPKDKPIKVNVSGGGTFCYGPTFSGGESYIVIEQCWQMHVKNAR
WP.016560869.1kUNK/1-638              ---------------MQNWKKIFIVVLLISIMIYLEYEMDHTLVHASSSSKTVDSIIQKPTDPPKDKPIKVNVSGGGTFCYGPTFSGGESYIVIEQCWQMHVMSAR
WP.004763090.1kUNK/1-638              ---------------MQNWKKIFIVVLLISIMIYLEYEMDHTLVHASSSSKIVDSIIQKPTDPPKDKPIKVIVHDGGKFCYGPTFSGGESYIVIEQCWQMHVMNAR
EMO77833.1kUNK_s200801925/1-638       ---------------MYNWKKIFIVVLLISIMIYLEYEMDHTLVHASSSSKTVDSIIQKPTDPPKDKPIKVNVSGGGTFCYGPTFSGGESYIVIEQCWQMHVMSAR
EKO52879.1kUNK_s200802841/1-638       ---------------MYNWKKIFIVVLLISIMIYLEYEMDHTFVHASSYSKTVDSIIQKPTDPPKDKPIKVNVSGGGTFCYGPTFSGGESYIVIEQCWQMHVMSAR
WP.061526938.1kUNK/1-640              ---------------MYNWKKIFIVALLVSIMIYLEHEMDHTLVHASSSLKTTDSIIQKPTDPPKDKPIKVNVSGGGTFCYGPTFSGGESYIIIEQCWQMHVMNAR
WP.016753526.1kUNK/1-639              ---------------MYNWKKIFIVVLLVSIMIYLAYEMDHTFVHA-AASKATSSIVQKPTDPPKDKPIKVIVHDGGKFCYGPTFSGGESYIIIEQCWQMHVMNAR
WP.004767507.1kUNK/1-639              ---------------MYNWKKIFIVVLLVSIMIYLAYEMDHTFVHA-AVSKATSSIVQKPTDPPKDKPIKVIVHDGGKFCYGPTFSGGESYIIIEQCWQMHVMNAR
WP.004763809.1kUNK/1-639              ---------------MYNWKKIFIVVLLVSIMIYLAYEMDHTFVHA-AASKATSSIVQKPTDPPKDKPIKVIVHDGGKFCYGPTFSGGESYIIIEQCWQMHVMNAR
WP.004761272.1kUNK/1-639              ---------------MYNWKKIFIVVLLVSIMIYLVYEMDHTFVHA-AVSKATSSIVQKPTDPPKDKPIKVIVHDGGKFCYGPTFSGGESYIIIEQCWQMHVMNAR
WP.004753356.1kUNK/1-639              ---------------MYNWKKIFIVVLLVSIMIYLAYEMDHTFVHA-AVSKATSSIVQKPTDPPKDKPIKVIVHDGGKFCYGPTFSGGESYIIIEQCWQMHVMNAR
EMO82366.1kUNK_s200801774/1-639       ---------------MYNWKKIFIVVLLVSIMIYLAYEMDHTFVHA-AVSKATSSIVQKPTDPPKDKPIKVIVHDGGKFCYGPTFSGGESYIIIEQCWQMHVMNAR
EMO76636.1kUNK_s200801925/1-630       ------------------------MVLLVSIMIYLAYEMDHTFVHA-AASKATSSIVQKPTDPPKDKPIKVIVHDGGKFCYGPTFSGGESYIIIEQCWQMHVMNAR
EMN06505.1kBIM_s1051/1-639            ---------------MYNWKKIFIVVLLVSIMIYLAYEMDHTFVHA-AASKATSSIVQKPTDPPKDKPIKVIVHDGGKFCYGPTFSGGESYIIIEQCWQMHVMNAR
WP.004782770.1kUNK/1-639              ---------------MYNWKKIFIVVLLVSIMIYLAYEMDHTLVHA-AASKATSSIVQKPTDPPKDKPIKVIVHDGGKFCYGPTFSGGESYIIIEQCWQMHVMNAR
PN5K11_PN5.26_AG15.kPN5-2/1-636       ---------------MRNWKKMFIVVLLVSIGVGFEYGMNPTPVDA-SSSQANYSIVQKPTDPPKDKPIQVVVSDGGTFCYGPTFSGGESYIIIEQCWEMHVKNAR
WP.004765912.1kUNK/1-637              ---------------MRNWKKMFIVVLLVSIGVGFEYGMNPTPIDA--SSKANYSIVQKPTDPPKDKPIKVNVSDGGTFCYGPIFSGGESYIIIEQCWERHVMNAR
PN5K1A_PN5.xxxx_AG15.kPN5/1-637       ---------------MRNWKKMFIVVLLVSIGVGFEYGMNPTPIDA--SSKANYSIVQKPTDPPKDKPIKVNVSDGGTFCYGPIFSGGESYIIIEQCWERHVMNAR
PN5K18_PN5.3138_AG15.kPN5/1-636       ---------------MRNWKKVFIVVLLVSIGVGFEYGMNPTPVDA-SSSQANYSIVQKPTDPPKDKPIQVVVSDGGKFCYGPIFSGGESYIIIEQCWERHVKNAR
PN5K1D_PN5.4761_AG15.kPN5/1-641       ---------------MRNWKKVFIVVLLVSIGVGFEYGMNPTPVDA-SSSQANYSIVQKPTDPPKDKPIQVVVSDGGKFCYGPIFSGGESYIIIEQCWERHVKNAR
PN5K12_PN5.738_AG15.kPN5-/1-538       ---------------MRNWKKVLIVVFLVSIGVGFEYGMNPTPVDA--SSKANYSIVQKPTDPPKDKPIKVNVSGGGTFCYGPTFSGGESYIIIEQCWEMHVKNAR
EMN03029.1kBIM_s1051/1-638            ---------------MYNWKKIFIVVLLISIMIYLEYEMDHTLVHASSSSKTVDSIIQKPTDPPKDKPIKVNVSGGGTFCYGPTFSGGESYIVIEQCWQMHVMSAR
EKR08500.1kVZI_s200702274/1-638       ---------------MYNWKKIFIVVLLISIMIYLEYEMDHTFVHASSSSKTVDSIIQKPTDPPKDKPIKVNVSGGGTFCYGPTFSGGESYIIIEQCWQMHVKNAR
WP.139363894.1kUNK/1-645              ---------------MYNWKKIFIVALLVSIMIYLEYEMDHTFIHASSSLKTTDSIIQKPTDPPKDKPIKVNVSGGGTFCYGPTFSGGENYIIIEQCWQMHVMNAR
KXZ29949.1kUNK_pAYB32.087/1-648       ---------------MYNWKKIFIVALLVSIMIYLEHEMDHTFIHASSYSKTTDSIIQKPTDPPKDKPIKVNVSGGGTFCYGPTFSGGENYIIIEQCWQMHVMNAR
WP.004783492.1kUNK/1-638              ---------------MYNWKKIFILVLLISIMIYLEYEMDHTFVHASSYSKTTDSIIQKPTDPPKDKPIQVVVHDGGKFCYGPIFSGGESYIIIEQCWQMHVMSAR
WP.057162238.1kUNK/1-638              ---------------MYNWKKIFIVVLLISIMIYLEYEMDHTLVHASSSSKTVDSIIQKPTDPPKDKPIKVNVSGGGTFCYGPTFSGGESYIVIEQCWQMHVMNAR
EPG51831.1kCRI_s3522CT/1-638          ---------------MYNWKKIFIVVLLISIMIYLEYEMDHTFVHASSSSKTVDSIIQKPTDPPKDKPIKVNVSGGGTFCYGPTFSGGESYIVIEQCWQMHVMNAR
EMO67081.1kUNK_s200803703/1-638       ---------------MYNWKKIFIVVLLVSIMIYLEYEMDHTLVHASSYSKTTDSIIQKPTDPPKDKPIKVNVSGGGTFCYGPTFSGGESYIVIEQCWQMHVMNAR
WP.004755380.1kUNK/1-638              ---------------MYNWKKIFIVALLVSIMIYLEYEMDHTLVHASSSSKTVDSIIQKPTDPPKDKPIKVNVSGGGTFCYGPTFSGGESYIVIEQCWQMHVKNAR
EKR10425.1kVZI_s200702274/1-638       ---------------MYNWKKIFIVVLLISIMIYLEYEMDHTFVHASSSSKTVDSIIQKPTDPPKDKPIKVNVSGGGTFCYGPTFSGGESYIIIEQCWQMHVKNAR
WP.004761023.1kUNK/1-638              ---------------MYNWKKIFIVVLLISIMIYLEYEMDHTFVHASSSSKTVDSIIQKPTDPPKDKPIKVNVSGGGTFCYGPTFSGGESYIIIEQCWQMHVKNAR
EJO71672.1kGSA_sRM52/1-638            ---------------MYNWKKIFIVVLLISIMIYLEYEMDHTFVHASSSSKTVDSIIQKPTDPPKDKPIKVNVSGGGTFCYGPTFSGGESYIIIEQCWQMHVKNAR
EMN27413.1kSNE_sRM1/1-638             ---------------MYNWKKIFIVVLLISIMIYLEYEMDHTLVHASSSSKTVDSIIQKPTDPPKDKPIKVNVSGGGTFCYGPTFSGGESYIVIEQCWQMHVMNAR
WP.016560936.1kUNK/1-635              ---------------MRNW-KVLILILLVSIVVGFEYGINYTLVHASSSSKTVDSIIQKPTDPPKDKPIKVNVSGGGTFCYGPTFSGGESYIIIEQCQQMHVMNAR
WP.004762307.1kUNK/1-635              ---------------MRNW-KVLILILLVSIVVGFEYGINYTLVHASSSSKTVDSIIQKPTDPPKDKPIKVNVSGGGTFCYGPTFSGGESYIIIEQCQQMHVMNAR
WP.004756854.1kUNK/1-635              ---------------MRNW-KVLILILLVSIVVGFEYGINYTLVHASSSSKTVDSIIQKPTDPPKDKPIKVNVSGGGTFCYGPTFSGGESYIIIEQCQQMHVMNAR
WP.053388497.1kUNK/1-635              ---------------MRNW-KVLILILLVSIVVGFEYGINYTFVHASSSSKTVDSIIQKPTDPPKDKPIKVNVSGGGTFCYGPTFSGGESYIIIEQCQQMHVMNAR
WP.004770274.1kUNK/1-635              ---------------MRNW-KVLILILLVSIVVGFEYGINYTLVHASSYSKTTDSIIQKPTDPPKDKPIKVNVSGGGTFCYGPTFSGGESYIIIEQCQQMHVMNAR
EMO75345.1kUNK_s200801925/1-635       ---------------MRNW-KVLILILLVSIVVGFEYGINYTLVHASSYSKTTDSIIQKPTDPPKDKPIKVNVSGGGTFCYGPTFSGGESYIIIEQCQQMHVMNAR
WP.025179280.1kUNK/1-640              ---------------MYNWKKMFIVVLLVSIVVGFEYGINYTFIHASSSSKTTGSVIQKPTDPPKDKPIKVNVSDGGTFCYGPTFSGGESYIIIEQCWEMHVKNAR
WP.004778812.1kUNK/1-636              ---------------MSNWKKMFIIVLLVSIGVGFEYGMDHTLVHA-SSSKTTGSIIQKPTDPPKDKPIKVNVSGGGTFCYGPTFSGGESYIIIEQCWEMHVKNAR
PN5K15_PN5.2001_AG15.kPN5/1-636       ---------------MSNWKKMFIIVLLVSIGVGFEYGMDHTLVHA-SSSKTTGSIIQKPTDPPKDKPIKVNVSGGGTFCYGPTFSGGESYIIIEQCWEMHVKNAR
WP.004779221.1kUNK/1-638              ---------------MYNWKKMFIVVLLVSIVVGFEYGINYTFIHASSSSKTTGSVIQKPTDPPKDKPIKVNVSDGGTFCYGPTFSGGESYIIIEQCWEMHVKNAR
WP.004778348.1kUNK/1-641              ---------------MRNWKKVFIVVLLVSIGVGFEYGMNPTPVDA-SSSQANYSIVQKPTDPPKDKPIQVVVSDGGKFCYGPIFSGGESYIIIEQCWERHVKNAR
PN5K13_PN5.1884_AG15.kPN5/1-639       ---------------MSNWKKMFIIVLLVSIGVGFEYGMNSTPVDA-SSSQANYSIVQKPTDPPKDKPIKVNVSGGGTFCYGPTFSGGESYIIIEQCWEMHVKNAR
PN5K17_PN5.3082_AG15.kPN5/1-635       ---------------MRNWKKVLIVVLLVSIGFGFEYGMNPTPVDA--SSKANYSIVQKPTDPPKDKPIKVNVSGGGTFCYGPTFSGGESYIIIEQCWEMHVKNAR
PN5K19_PN5.3140_AG15.kPN5/1-637       ---------------MRNWKKVLIVVLLVSIGLGFEYGMNSTPVDA-SSSQANYSIIQKPTDPPKDKPIKVNVSDGGTFCYGPTFSGGESYIIIEQCWEMHVKNAR
WP.194099470.1kUNK/1-638              ---------------MRKW-KVSLLVLFVFIRVDYSYGVNPAFIPI--PSGVTGSIVQKPTDPPKDKAIKVVIHDGVKFCYGPIFRGGESYIIVEQCSEQHVMNAR
WP.020765398.1mUNK/1-638              ---------------MRKW-KVSLLVLFVFIRVDYSYGVNPAFIPI--PSGVTGSIVQKPTDPPKDKAIKVVIHDGVKFCYGPIFRGGESYIIVEQCSEQHVMNAR
WP.004768037.1kUNK/1-638              ---------------MRKW-KVSLLVLFVFIRVDYSYGVNPAFTPI--PSGVTGSIVQKPTDPPKDKAIKVVIHDGVKFCYGPIFSGGESYIIVEQCSEQHVMNAR
WP.016751059.1kUNK/1-638              ---------------MRKW-KVSLLVLFVFIRVDYSYGVNPAFTPI--PSGVTGSIVQKPTDPPKDKAIKVVIHDGVKFCYGPIFSGGESYIIVEQCSEQHVMNAR
WP.004751295.1kUNK/1-638              ---------------MRKW-KVSLLVLFVFIRVDYSYGVNPAFTPI--PSGVTGSIVQKPTDPPKDKAIKVVIHDGVKFCYGPIFSGGESYIIVEQCSEQHVMNAR
WP.004763471.1kUNK/1-638              ---------------MRKW-KVSLLVLFVFIRVDYSYGVNPAFTPI--PSGVTGSIVQKPTDPPKDKAIKVVIHDGVKFCYGPIFSGGESYIIVEQCSEQHVMNAR
WP.016560802.1kUNK/1-638              ---------------MRKW-KVSLLVLFVFIRVDYSYGVNPAFTPI--PSGVTGSIVQKPTDPPKDKAIKVVIHDGVKFCYGPIFSGGESYIIVEQCSEQHVMNAR
WP.004781922.1kUNK/1-638              ---------------MRKW-KVSLLVLFVFIRVDYSYGVNPAFTPI--PSGVTGSIVQKPTDPPKDKAIKVVIHDGVKFCYGPIFSGGESYIIVEQCSEQHVMNAR
WP.020762537.1kUNK/1-641              ---------------MYNWKKMFIVVLLVSIVVGFEYGINYTFIHASSSSKTTGSVIQKPTDPPKDKPIKVNVSDGGTFCYGPTFSGGESYIIIEQCWEMHVKNAR
WP.004778411.1kUNK/1-638              ---------------MRKW-KVSLLVLFVFIRVDYSYGVNPAFIPI--SSGVTGSIVQKPTDPPKDKPIKVNVSGGGTFCYGPTFSGGESYIIIEQCWEMHVKNAR
WP.004765202.1kUNK/1-640              ---------------MSNWKKMFIIVLLVSIGVGFEYGMDHTLVHA-SSSKTTGSIIQKPTDPPKDKPIKVNVSGGGTFCYGPTFSGGESYIIIEQCWEMHVKNAR
PN5K16_PN5.2913_AG15.kPN5/1-638       ---------------MRKW-KVSLLVLFVFIRVDYSYGVNPAFIPI--SSGVTGSIVQKPTDPPKDKPIKVNVSGGGTFCYGPTFSGGESYIIIEQCWEMHVKNAR
WP.192504711.1iUNK/1-638              ---------------MRKW-KVSLLVLFVFIRADYSYGVNPAFTPI--PSSVTGSIVQKSTDSPKDKAIKVVIHDGRKFCYGPIFSGGESYIIVEQCSEQHVMNAR
WP.137170976.1iUNK/1-638              ---------------MRKW-KVSLLVLFVFIRADYSYGVNPAFTPI--PSSVTGSIVQKSTDPPKDKAIKVVIHDGRKFCYGPIFSGGESYIIVEQCLEQHVMNAR
WP.137108447.1iUNK/1-638              ---------------MRKW-KVSLLVLFVFIRADYSYGVNPAFTPI--PSSVTGSIVQKSTDPPKDKAIKVVIHDGRKFCYGPIFSGGESYIIVEQCLEQHVMNAR
WP.096693360.1iUNK/1-638              ---------------MRKW-KVSLLVLFVFIRVDYSYGVNPAFTPI--PSSVTGSIVQKSTDPPKDKVIKVVIHDGRKFCYGPIFSGGESYIIVEQCSEQHVMNAR
WP.061278339.1iUNK/1-638              ---------------MRKW-KVSLLVLFVFIRADYSYGVNPAFTPI--PSSVTGSIVQKSTDSPKDKAIKVVIHDGRKFCYGPIFSGGESYIIVEQCSEQHVMNAR
WP.061242781.1iUNK/1-638              ---------------MRKW-KVSLLVLFVFIRADYSYGVNPAFTPI--PSSVTGSIVQKSTDSPKDKAIKVVIHDGRKFCYGPIFSGGESYIIVEQCSEQHVMNAR
WP.061234719.1iUNK/1-638              ---------------MRKW-KVSLLVLFVFIRVDYSYGVNPAFTPI--PSSVTGSIVQKSTDPPKDKVIKVVIHDGRKFCYGPIFSGGESYIIVEQCSEQHVMNAR
WP.002183635.1iUNK/1-638              ---------------MRKW-KVSLLVLFVFIRADYSYGVNPAFTPI--PSSVTGSIVQKSTDSPKDKAIKVVIHDGRKFCYGPIFSGGESYIIVEQCSEQHVMNAR
WP.002101592.1iUNK/1-638              ---------------MRKW-KVSLLVLFVFIRVDYSYGVNPAFTPI--PSSVTGSIVQKSTDPPKDKVIKVVIHDGRKFCYGPIFSGGESYIIVEQCSEQHVMNAR
WP.001233616.1iUNK/1-638              ---------------MRKW-KVSLLVLFVFIRVDYSYGVNPAFTPI--SSSVTGSIVQKSTDPPKDKAIKVVIHDGRKFCYGPIFSGGESYIIVEQCSEQHVMNAR
WP.001233611.1iUNK/1-638              ---------------MRKW-KVSLLVLFVFIRVDYSYGVNPAFTPI--PSSVTGSIVQKSTDPPKDKAIKVVIHDGRKFCYGPIFSGGESYIIVEQCSEQHVMNAR
WP.001233608.1iUNK/1-638              ---------------MRKW-KVSLLVLFVFIRADYSYGVNPAFTPI--PSSVTGSIVQKSTDSPKDKAIKVVIHDGRKFCYGPIFSGGESYIIVEQCSEQHVMNAR
WP.001233601.1iUNK/1-638              ---------------MRKW-KVSLLVLFVFIRADYSYGVNPAFTPI--PSSVTGSIVQKSTDSPKDKAIKVVIHDGRKFCYGPIFSGGESYIIVEQCSEQHVMNAR
WP.001233600.1iUNK/1-638              ---------------MRKW-KVSLLVLFVFIRADYSYGVNPAFTPI--PSSVTGSIVQKSTDSPKDKAIKVVIHDGRKFCYGPIFSGGESYIIVEQCSEQHVMNAR
RT2Q17_RT2.1631_AG3.iRT2-/1-638       ---------------MRKW-KVSLLVLFVFIRADYSYGVNPAFTPI--PSSVTGSIVQKSTDPPKDKAIKVVIHDGRKFCYGPIFSGGESYIIVEQCLEQHVMNAR
MAE.iUPMMCNIIDLP_1628862/1-638        ---------------MRKW-KVSLLVLFVFIRVDYSYGVNPAFTPI--SSSVTGSIVQKSTDPPKDKAIKVVIHDGRKFCYGPIFSGGESYIIVEQCSEQHVMNAR
MAE.iUPMMCNIIDHP_1628864/1-638        ---------------MRKW-KVSLLVLFVFIRVDYSYGVNPAFTPI--SSSVTGSIVQKSTDPPKDKAIKVVIHDGRKFCYGPIFSGGESYIIVEQCSEQHVMNAR
KW2Q17_KW2.1631_AG3.iKW2-/1-638       ---------------MRKW-KVSLLVLFVFIRADYSYGVNPAFTPI--PSSVTGSIVQKSTDPPKDKAIKVVIHDGRKFCYGPIFSGGESYIIVEQCLEQHVMNAR
KW2Q17_KW2.1608_AG3.iKW2-/1-638       ---------------MRKW-KVSLLVLFVFIRADYSYGVNPAFTPI--PSSVTGSIVQKSTDPPKDKAIKVVIHDGRKFCYGPIFSGGESYIIVEQCLEQHVMNAR
KW1Q17_KW1.2516_AG3.iKW1-/1-638       ---------------MRKW-KVSLLVLFVFIRADYSYGVNPAFTPI--PSSVTGSIVQKSTDPPKDKAIKVVIHDGRKFCYGPIFSGGESYIIVEQCLEQHVMNAR
KAA1291929.1iGRA/1-638                ---------------MRKW-KVSLLVLFVFIRADYSYGVNPAFTPI--PSSVTGSIVQKSTDPPKDKAIKVVIHDGRKFCYGPIFSGGESYIIVEQCLEQHVMNAR
ICT.iLangkawi_1718672/1-638           ---------------MRKW-KVSLLVLFVFIRADYSYGVNPAFTPI--PSSVTGSIVQKSTDSPKDKAIKVVIHDGRKFCYGPIFSGGESYIIVEQCSEQHVMNAR
BAE.iS1548_2676815/1-638              ---------------MRKW-KVSLLVLFVFIRADYSYGVNPAFTPI--PSSVTGSIVQKSTDSPKDKAIKVVIHDGRKFCYGPIFSGGESYIIVEQCSEQHVMNAR
WP.020784150.1iUNK/1-638              ---------------MRKW-KVSLLVLFVFIRADYSYGVNPAFTPI--PSSVTGSIVQKSTDSPKDKAIKVVIHDGRKFCYGPIFSGGESYIIVEQCSEQHVMNAR
WP.017857966.1iUNK/1-638              ---------------MRKW-KVSLLVLFVFIRADYSYGVNPAFTPI--PSSVTGSIVQKSTDPPKDKAIKVVIHDGRKFCYGPIFSGGESYIIVEQCLEQHVMNAR
WP.001233598.1iUNK/1-638              ---------------MRKW-KVSLLVLFVFIRADYSYGVNPAFTPI--PSSVTGSIVQKSTDSPKDKAIKVVIHDGRKFCYGPIFSGGESYIIVEQCSEQHVMNAR
CLA.iS782_1810732/1-638               ---------------MRKW-KVSLLVLFVFIRADYSYGVNPAFTPI--PSSVTGSIVQKSTDSPKDKAIKVVIHDGRKFCYGPIFSGGESYIIVEQCSEQHVMNAR
AW3Q17_AW3.3122_AG10.iAW3/1-638       ---------------MRKW-KVSLLVLFVFIRADYSYGVNPAFTPI--PSSVTGSIVQKSTDSPKDKAIKVVIHDGRKFCYGPIFSGGESYIIVEQCSEQHVMNAR
```

```
AW3Q17_AW3.3122_AG10.iAW3/1-638 ---------------MRKW-KVSLLVLFVFIRADYSYGVNPAFTPI--PSSVTGSIVQKSTDSPKDKAIKVVIHDGRKFCYGPIFSGGESYIIVEQCSEQHVMNAR
AW1Q17_AW1.1516_AG3.iAW1-/1-638 ---------------MRKW-KVSLLVLFVFIRADYSYGVNPAFTPI--PSSVTGSIVQKSTDPPKDKAIKVVIHDGRKFCYGPIFSGGESYIIVEQCLEQHVMNAR
AP6Q17_AP6.2763_AG3.iAP6-/1-638 ---------------MRKW-KVSLLVLFVFIRADYSYGVNPAFTPI--PSSVTGSIVQKSTDSPKDKAIKVVIHDGRKFCYGPIFSGGESYIIVEQCSEQHVMNAR
AP5Q17_AP5.2698_AG10.iAP5/1-638 ---------------MRKW-KVSLLVLFVFIRADYSYGVNPAFTPI--PSSVTGSIVQKSTDSPKDKAIKVVIHDGRKFCYGPIFSGGESYIIVEQCSEQHVMNAR
EMN48597.1iUNK_sL1207/1-638 ---------------MRKW-KVSLLVLFVFIRADYSYGVNPAFTPI--PSSVTGSIVQKSTDSPKDKAIKVVIHDGRKFCYGPIFSGGESYIIVEQCSEQHVMNAR
WP.061270948.1iUNK/1-638 ---------------MRKW-KVSLLVLFVFIRADYSYGVNPAFTPI--PSSVTGSIVQKSTDSPKDKAIKVVIHDGRKFCYGPIFSGGESYIIVEQCSEQHVMNAR
WP.001233599.1iUNK/1-638 ---------------MRKW-KVSLLVLFVFIRADYSYGVNPAFTPI--PSSVTGSIVQKSTDSPKDKAIKVVIHDGRKFCYGPIFSGGESYIIVEQCSEQHVMNAR
WP.001233602.1iUNK/1-638 ---------------MRKW-KVSLLVLFVFIRADYSYGVNPAFTPI--PSSVTGSIVQKSTDSPKDKAIKVVIHDGRKFCYGPIFSGGESYIIVEQCSEQHVMNAR
WP.017854276.1iUNK/1-630 ---------------MRKW-KVSLLVLFVFIRADYSYGVNPAFTPI------PSSV----TDSPKDKAIKVVIHDGRKFCYGPIFSGGESYIIVEQCSEQHVMNAR
WP.001233615.1iUNK/1-638 ---------------MRKW-KVSLLVLFVFIRVDYSYGVNPAFTPI--PSSVTGSIVQKSTDPPKDKVIKVVIHDGRKFCYGPIFSGGESYIIVEQCSEQHVMNAR
WP.061270182.1iUNK/1-638 ---------------MRKW-KVSLLVLFVFIRVDYSYGVNPAFTPI--PSSVTGSIVQKSTDPPKDKAIKVVIHDGRKFCYGPIFSGGESYIIVEQCSEQHVMNAR
WP.061266475.1iUNK/1-638 ---------------MRKW-KVSLLVLFVFIRVDYSYGVNPAFTPI--PSSVTGSIVQKSTDPPKDKVIKVVIHDGRKFCYGPIFSGGESYIIVEQCSEQHVMNAR
WP.061245955.1iUNK/1-638 ---------------MRKW-KVSLLVLFVFIRVDYSYGVNPAFTPI--PSSVTGSIVQKSTDPPKDKVIKVVIHDGRKFCYGPIFSGGESYIIVEQCSEQHVMNAR
WP.061231395.1iUNK/1-638 ---------------MRKW-KVSLLVLFVFIRVDYSYGVNPAFTPI--PSSVTGSIVQKSTDPPKDKVIKVVIHDGRKFCYGPIFSGGESYIIVEQCSEQHVMNAR
WP.002107870.1iUNK/1-638 ---------------MRKW-KVSLLVLFVFIRVDYSYGVNPAFTPI--PSSVTGSIVQKSTDPPKDKVIKVVIHDGRKFCYGPIFSGGESYIIVEQCSEQHVMNAR
WP.001233614.1iUNK/1-638 ---------------MRKW-KVSLLVLFVFIRVDYSYGVNPAFTPI--PSSVTGSIVQKSTDPPKDKVIKVVIHDGRKFCYGPIFSGGESYIIVEQCSEQHVMNAR
WP.001233613.1iUNK/1-638 ---------------MRKW-KVSLLVLFVFIRVDYSYGVNPAFTPI--PSSVTGSIVQKSTDPPKDKVIKVVIHDGRKFCYGPIFSGGESYIIVEQCSEQHVMNAR
WP.001233603.1iUNK/1-638 ---------------MRKW-KVSLLVLFVFIRADYSYGVNPAFTPI--PSSVTGSIVQKSTDSPKDKAIKVVIHDGRKFCYGPIFSGGESYIIVEQCSEQHVMNAR
WP.001233597.1iUNK/1-638 ---------------MRKW-KVSLLVLFVFIRADYSYGVNPAFTPI--PSSVTGSIVQKSTDSPKDKAIKVVIHDGRKFCYGPIFSGGESYIIVEQCSEQHVMNAR
PN2Q17_PN2.2605_AG14.iPN2/1-638 ---------------MRKW-KVSLLVLFVFIRVDYSYGVNPAFTPI--PSSVTGSIVQKSTDPPKDKAIKVVIHDGRKFCYGPIFSGGESYIIVEQCSEQHVMNAR
LHI.iS56609_1746353/1-638 ---------------MRKW-KVSLLVLFVFIRVDYSYGVNPAFTPI--PSSVTGSIVQKSTDPPKDKVIKVVIHDGRKFCYGPIFSGGESYIIVEQCSEQHVMNAR
KG1Q18_KG1.2904_AG14.uKG1/1-638 ---------------MRKW-KVSLLVLFVFIRVDYSYGVNPAFTPI--PSSVTGSIVQKSTDPPKDKAIKVVIHDGRKFCYGPIFSGGESYIIVEQCSEQHVMNAR
WP.061244985.1iUNK/1-638 ---------------MRKW-KVSLLVLFVFIRVDYSYGVNPAFTPI--PSSVTGSIVQKSTDPPKDKVIKVVIHDGRKFCYGPIFSGGESYIIVEQCSEQHVMNAR
WP.001233612.1iUNK/1-638 ---------------MRKW-KVSLLVLFVFIRVDYSYGVNPAFTPI--PSSVTGSIVQKSTDPPKDKVIKVVIHDGRKFCYGPIFSGGESYIIVEQCSEQHVMNAR
WP.001233604.1iUNK/1-638 ---------------MRKW-KVSLLVLFVFIRADYSYGVNPAFTPI--PSSVTGSIVQKSTDSPKDKAIKVVIHDGRKFCYGPIFSGGESYIIVEQCSEQHVMNAR
WP.061270534.1iUNK/1-638 ---------------MRKW-KVSLLVLFVFIRVDYSYGVNPAFTPI--PSSVTGSIVQKSTDPPKDKVIKVVIHDGRKFCYGPIFSGGESYIIVEQCSEQHVMNAR
WP.061243789.1iUNK/1-638 ---------------MRKW-KVSLLVLFVFIRADYSYGVNPAFTPI--PSSVTGSIVQKSTDSPKDKAIKVVIHDGRKFCYGPIFSGGESYIIVEQCSEQHVMNAR
WP.017855561.1iUNK/1-638 ---------------MRKW-KVSLLVLFVFIRADYSYGVNPAFTPI--PSSVTGSIVQKSTDSPKDKAIKVVIHDGRKFCYGPIFSGGESYIIVEQCSEQHVMNAR
WP.011069716.1iUNK/1-638 ---------------MRKW-KVSLLVLFVFIRADYSYGVNPAFTPI--PSSVTGSIVQKSTDSPKDKAIKVVIHDGRKFCYGPIFSGGESYIIVEQCSEQHVMNAR
WP.002106570.1iUNK/1-638 ---------------MRKW-KVSLLVLFVFIRVDYSYGVNPAFTPI--PSSVTGSIVQKSTDSPKDKAIKVVIHDGRKFCYGPIFSGGESYIIVEQCSEQHVMNAR
WP.001233607.1iUNK/1-638 ---------------MRKW-KVSLLVLFVFIRADYSYGVNPAFTPI--PSSVTGSIVQKSTDSPKDKAIKVVIHDGRKFCYGPIFSGGESYIIVEQCSEQHVMNAR
WP.001233606.1iUNK/1-638 ---------------MRKW-KVSLLVLFVFIRADYSYGVNPAFTPI--PSSVTGSIVQKSTDSPKDKAIKVVIHDGRKFCYGPIFSGGESYIIVEQCSEQHVMNAR
WP.001233605.1iUNK/1-638 ---------------MRKW-KVSLLVLFVFIRADYSYGVNPAFTPI--PSSVTGSIVQKSTDSPKDKAIKVVIHDGRKFCYGPIFSGGESYIIVEQCSEQHVMNAR
UNK.iR7_1642019/1-638 ---------------MRKW-KVSLLVLFVFIRADYSYGVNPAFTPI--PSSVTGSIVQKSTDSPKDKAIKVVIHDGRKFCYGPIFSGGESYIIVEQCSEQHVMNAR
UNK.iR22_1652003/1-638 ---------------MRKW-KVSLLVLFVFIRADYSYGVNPAFTPI--PSSVTGSIVQKSTDSPKDKAIKVVIHDGRKFCYGPIFSGGESYIIVEQCSEQHVMNAR
UNK.iR21_1651853/1-638 ---------------MRKW-KVSLLVLFVFIRADYSYGVNPAFTPI--PSSVTGSIVQKSTDSPKDKAIKVVIHDGRKFCYGPIFSGGESYIIVEQCSEQHVMNAR
UNK.iR19_1651662/1-638 ---------------MRKW-KVSLLVLFVFIRADYSYGVNPAFTPI--PSSVTGSIVQKSTDSPKDKAIKVVIHDGRKFCYGPIFSGGESYIIVEQCSEQHVMNAR
UNK.iR17_1652494/1-638 ---------------MRKW-KVSLLVLFVFIRADYSYGVNPAFTPI--PSSVTGSIVQKSTDSPKDKAIKVVIHDGRKFCYGPIFSGGESYIIVEQCSEQHVMNAR
UNK.iR16_1642272/1-638 ---------------MRKW-KVSLLVLFVFIRADYSYGVNPAFTPI--PSSVTGSIVQKSTDSPKDKAIKVVIHDGRKFCYGPIFSGGESYIIVEQCSEQHVMNAR
UNK.iR13_1641854/1-638 ---------------MRKW-KVSLLVLFVFIRADYSYGVNPAFTPI--PSSVTGSIVQKSTDSPKDKAIKVVIHDGRKFCYGPIFSGGESYIIVEQCSEQHVMNAR
UNK.iR13L_1658926/1-638 ---------------MRKW-KVSLLVLFVFIRADYSYGVNPAFTPI--PSSVTGSIVQKSTDSPKDKAIKVVIHDGRKFCYGPIFSGGESYIIVEQCSEQHVMNAR
UNK.iR12_1647532/1-638 ---------------MRKW-KVSLLVLFVFIRADYSYGVNPAFTPI--PSSVTGSIVQKSTDSPKDKAIKVVIHDGRKFCYGPIFSGGESYIIVEQCSEQHVMNAR
UNK.iR11_1642961/1-638 ---------------MRKW-KVSLLVLFVFIRADYSYGVNPAFTPI--PSSVTGSIVQKSTDSPKDKAIKVVIHDGRKFCYGPIFSGGESYIIVEQCSEQHVMNAR
PD1Q15_PD1.2182_AG3.iPD1-/1-638 ---------------MRKW-KVSLLVLFVFIRVDYSYGVNPAFTPI--PSSVTGSIVQKSTDSPKDKAIKVVIHDGRKFCYGPIFSGGESYIIVEQCSEQHVMNAR
LAI.iS56601_2614706/1-638 ---------------MRKW-KVSLLVLFVFIRADYSYGVNPAFTPI--PSSVTGSIVQKSTDSPKDKAIKVVIHDGRKFCYGPIFSGGESYIIVEQCSEQHVMNAR
LAI.iIPAV_2612986/1-638 ---------------MRKW-KVSLLVLFVFIRADYSYGVNPAFTPI--PSSVTGSIVQKSTDSPKDKAIKVVIHDGRKFCYGPIFSGGESYIIVEQCSEQHVMNAR
KG2Q17_KG2.2644_AG3.uKG2-/1-638 ---------------MRKW-KVSLLVLFVFIRVDYSYGVNPAFTPI--PSSVTGSIVQKSTDSPKDKAIKVVIHDGRKFCYGPIFSGGESYIIVEQCSEQHVMNAR
ICT.iS898_1672311/1-638 ---------------MRKW-KVSLLVLFVFIRADYSYGVNPAFTPI--PSSVTGSIVQKSTDSPKDKAIKVVIHDGRKFCYGPIFSGGESYIIVEQCSEQHVMNAR
ICT.iPiscina_1668885/1-638 ---------------MRKW-KVSLLVLFDFIRADYSYGVNPAFTPI--PSSVTGSIVQKSTDSPKDKAIKVVIHDGRKFCYGPIFSGGESYIIVEQCSEQHVMNAR
HJO.iNorma_2666463/1-638 ---------------MRKW-KVSLLVLFVFIRADYSYGVNPAFTPI--PSSVTGSIVQKSTDSPKDKAIKVVIHDGRKFCYGPIFSGGESYIIVEQCSEQHVMNAR
HJO.iL53_2715159/1-638 ---------------MRKW-KVSLLVLFVFIRADYSYGVNPAFTPI--PSSVTGSIVQKSTDSPKDKAIKVVIHDGRKFCYGPIFSGGESYIIVEQCSEQHVMNAR
HJO.iHjtno_2683946/1-638 ---------------MRKW-KVSLLVLFVFIRADYSYGVNPAFTPI--PSSVTGSIVQKSTDSPKDKAIKVVIHDGRKFCYGPIFSGGESYIIVEQCSEQHVMNAR
EMM83676.1iUNK_s200600185/1-638 ---------------MRKW-KVSLLVLFVFIRADYSYGVNPAFTPI--PSSVTGSIVQKSTDSPKDKAIKVVIHDGRKFCYGPIFSGGESYIIVEQCSEQHVMNAR
COP.iSK1_1672234/1-638 ---------------MRKW-KVSLLVLFVFIRADYSYGVNPAFTPI--PSSVTGSIVQKSTDSPKDKAIKVVIHDGRKFCYGPIFSGGESYIIVEQCSEQHVMNAR
COP.iL1130_Q72SM1.cl1672/1-638 ---------------MRKW-KVSLLVLFVFIRADYSYGVNPAFTPI--PSSVTGSIVQKSTDSPKDKAIKVVIHDGRKFCYGPIFSGGESYIIVEQCSEQHVMNAR
COP.iFDAARGOS203_3962248/1-638 ---------------MRKW-KVSLLVLFVFIRADYSYGVNPAFTPI--PSSVTGSIVQKSTDSPKDKAIKVVIHDGRKFCYGPIFSGGESYIIVEQCSEQHVMNAR
CLA.iS611_2566653/1-638 ---------------MRKW-KVSLLVLFVFIRADYSYGVNPAFTPI--PSSVTGSIVQKSTDSPKDKAIKVVIHDGRKFCYGPIFSGGESYIIVEQCSEQHVMNAR
CLA.iS114_4033293/1-638 ---------------MRKW-KVSLLVLFVFIRADYSYGVNPAFTPI--PSSVTGSIVQKSTDSPKDKAIKVVIHDGRKFCYGPIFSGGESYIIVEQCSEQHVMNAR
CLA.iLJ178_2570862/1-638 ---------------MRKW-KVSLLVLFVFIRADYSYGVNPAFTPI--PSSVTGSIVQKSTDSPKDKAIKVVIHDGRKFCYGPIFSGGESYIIVEQCSEQHVMNAR
ASV09804.1iCLA_pB2G50.171/1-638 ---------------MRKW-KVSLLVLFVLIRADYSYGVNPAFTPI--PSSVTGSIVQKSTDSPKDKAIKVVIHDGRKFCYGPIFSGGESYIIVEQCSEQHVMNAR
APH41258.1iICT/1-638 ---------------MRKW-KVSLLVLFDFIRADYSYGVNPAFTPI--PSSVTGSIVQKSTDSPKDKAIKVVIHDGRKFCYGPIFSGGESYIIVEQCSEQHVMNAR
WP.017862192.1iUNK/1-638 ---------------MRKW-KVFLLVLFVFIRADYSYGVNPAFTPI--PSSVTGSIVQKSTDSPKDKAIKVVIHDGRKFCYGPIFSGGESYIIVEQCSEQHVMNAR
WP.001233592.1iUNK/1-638 ---------------MRKW-KVFLLVLFVFIRADYSYGVNPAFTPI--PSSVTGSIVQKSTDSPKDKAIKVVIHDGRKFCYGPIFSGGESYIIVEQCSEQHVMNAR
KWV28310.1iUNK_pLA702.100/1-645 --------mlqkERKMRKW-KVFLLVLFVFIRADYSYGVNPAFTPI--PSSVTGSIVQKSTDSPKDKAIKVVIHDGRKFCYGPIFSGGESYIIVEQCSEQHVMNAR
BVA.iPigK151_1730734/1-638 ---------------MRKW-KVFLLVLFVFIRADYSYGVNPAFTPI--PSSVTGSIVQKSTDSPKDKAIKVVIHDGRKFCYGPIFSGGESYIIVEQCSEQHVMNAR
PN3Q17_SLPN3.1718_AG3.iPN/1-638 ---------------MRKW-KVSLLVLFVFIRADYSYGVNPAFTPI--PSSVTGSIVQKSTDPPKDKAIKVVIHDGRKFCYGPIFSGGESYIIVEQCSEQHVMNAR
KAA1267482.1iWHE/1-638 ---------------MRKW-KVSLLVLFVFIRADYSYGVNPAFTPI--PSSVTGSIVQKSTDPPKDKAIKVVIHDGRKFCYGPIFSGGESYIIVEQCSEQHVMNAR
AP7Q17_AP7.1619_AG3.iAP7-/1-638 ---------------MRKW-KVSLLVLFVFIRADYSYGVNPAFTPI--PSSVTGSIVQKSTDPPKDKAIKVVIHDGRKFCYGPIFSGGESYIIVEQCSEQHVMNAR
AP3Q17_AP3.1718_AG10.iAP3/1-638 ---------------MRKW-KVSLLVLFVFIRADYSYGVNPAFTPI--PSSVTGSIVQKSTDPPKDKAIKVVIHDGRKFCYGPIFSGGESYIIVEQCSEQHVMNAR
AP1Q16_AP1.2160_AG3.iAP1-/1-638 ---------------MRKW-KVSLLVLFVFIRADYSYGVNPAFTPI--PSSVTGSIVQKSTDPPKDKAIKVVIHDGRKFCYGPIFSGGESYIIVEQCSEQHVMNAR
AG3Q17_U1.1718_AG3.iO7371/1-638 ---------------MRKW-KVSLLVLFVFIRADYSYGVNPAFTPI--PSSVTGSIVQKSTDPPKDKAIKVVIHDGRKFCYGPIFSGGESYIIVEQCSEQHVMNAR
WP.203368468.1iUNK/1-635 ---------------MRHS-IVFIVVLLVLVTTSFEYSINNIHIHA--SSNTSNSIIQKPTDQPKDKPIKIVIHDGKKFCYAPVFSGGESYIVIEQCWQMHVMNAR
WP.192503305.1iUNK/1-635 ---------------MRHS-IVFIIVLLVLVTTSFEYSINNIHIHA--SSNMSNSIIQKPTDQPKDKPIKIVIHDGKKFCYAPVFSGGESYIVIEQCWQMHVMNAR
WP.096637860.1iUNK/1-635 ---------------MRHS-IVFIVVLLVLVTTSFEYSINNIHIHA--SSNMSNSIIQKPTDQPKDKPIKIVIHDGKKFCYAPVFSGGESYIVIEQCWQMHVMNAR
WP.025180359.1iUNK/1-635 ---------------MRHS-IVFIIVLLVLVTTSFEYSINNIHIHA--SSNTSNSIIQKPTDQPKDKPIKIVIHDGKKFCYAPVFSGGESYIVIEQCWQMHVMNAR
WP.025177699.1iUNK/1-635 ---------------MRHS-IVFIVVLLVLVTTSFEYSINNIHIHA--SSNMSNSIIQKPTDQPKDKPIKIVIHDGKKFCYAPVFSGGESYIVIEQCWQMHVMNAR
WP.020781271.1iUNK/1-635 ---------------MRHS-IVFIIVLLVLVTTSFEYSINNIHIHA--SSNMSNSIIQKPTDQPKDKPIKIVIHDGKKFCYAPVFSGGESYIVIEQCWQMHVMNAR
WP.017862215.1iUNK/1-635 ---------------MRHS-IVFIIVLLVLVTTSFEYSINNIHIHA--SSNMSNSIIQKPTDQPKDKPIKIVIHDGKKFCYAPVFSGGESYIVIEQCWQMHVMNAR
WP.002118342.1iUNK/1-635 ---------------MRHS-IVFIVVLLVLVTTSFEYSINNIHIHA--SSNTSNSIIQKPTDQPKDKPIKIVIHDGKKFCYAPVFSGGESYIVIEQCWQMHVMNAR
WP.002097357.1iUNK/1-635 ---------------MRHS-IVFIIVLLVLVTTSFEYSINNIHIHA--SSNMSNSIIQKPTDQPKDKPIKIVIHDGKKFCYAPVFSGGESYIVIEQCWQMHVMNAR
WP.001216433.1iUNK/1-635 ---------------MRHS-IVFIVVLLVLVTTSFEYSINNIHIHA--SSNTSNSIIQKPTDQPKDKPIKIVIHDGKKFCYAPVFSGGESYIVIEQCWQMHVMNAR
PD1Q1B_PD1.4154_AG3.iPD1-/1-635 ---------------MRHS-IVFIVVLLVLVTTSFEYSINNIHIHA--SSNTSNSIIQKPTDQPKDKPIKIVIHDGKKFCYAPVFSGGESYIVIKQCWQMHVMNAR
KG2Q14_KG2.1661_AG3.uKG2/1-635 ---------------MRHS-IVFIVVLLVLVTTSFEYSINNIHIHA--SSNTSNSIIQKPTDQPKDKPIKIVIHDGKKFCYAPVFSGGESYIVIKQCWQMHVMNAR
ICT.iLangkawi_3513593/1-635 ---------------MRHS-IVFIIVLLVLVTTSFEYSINNIHIHA--SSNMSNSIIQKPTDQPKDKPIKIVIHDGKKFCYAPVFSGGESYIVIEQCWQMHVMNAR
HJO.iNorma_885592/1-635 ---------------MRHS-IVFIVVLLVLVTTSFEYSINNIHIHA--SSNMSNSIIQKPTDQPKDKPIKIVIHDGKKFCYAPVFSGGESYIVIEQCWQMHVMNAR
HJO.iL53_896000/1-635 ---------------MRHS-IVFIVVLLVLVTTSFEYSINNIHIHA--SSNMSNSIIQKPTDQPKDKPIKIVIHDGKKFCYAPVFSGGESYIVIEQCWQMHVMNAR
HJO.iHjtno_883288/1-635 ---------------MRHS-IVFIVVLLVLVTTSFEYSINNIHIHA--SSNMSNSIIQKPTDQPKDKPIKIVIHDGKKFCYAPVFSGGESYIVIEQCWQMHVMNAR
EMN71213.1iBAE_sUI08561/1-635 ---------------MRHS-IVFIIVLLVLVTTSFEYSINNIHIHA--SSNTSNSIIQKPTDQPKDKPIKIVIHDGKKFCYAPVFSGGESYIVIEQCWQMHVMNAR
EKN89682.1iUNK_s200200062/1-602 ---------------------------------------------------mSNSIIQKPTDQPKDKPIKIVIHDGKKFCYAPVFSGGESYIVIEQCWQMHVMNAR
BVA.iPigK151_3571695/1-635 ---------------MRHS-IVFIIVLLVLVTTSFEYSINNIHIHA--SSNMSNSIIQKPTDQPKDKPIKIVIHDGKKFCYAPVFSGGESYIVIEQCWQMHVMNAR
ALE38144.1iHJO_pG436.0931/1-602 ---------------m------------------------------------SNSIIQKPTDQPKDKPIKIVIHDGKKFCYAPVFSGGESYIVIEQCWQMHVMNAR
WP.001216436.1iUNK/1-635 ---------------MRHS-IVFIVVLLVLVTTSFEYSINNIHIHA--SSNTSNSIIQKPTDQPKDKPIKIVIHDGKKFCYAPVFSGGESYIVIEQCWQMHVMNAR
LHI.iS56609_3515502/1-635 ---------------MRHS-IVFIVVLLVLVTTSFEYSINNIHIHA--SSNTSNSIIQKPTDQPKDKPIKIVIHDGKKFCYAPVFSGGESYIVIEQCWQMHVMNAR
AW3X1A_AW3.3967_AG10.iAW3/1-373 ---------------MRHS-IVFIVVLLVLVTTSFEYSINNIHIHA--SSNTSNSIIQKPTDQPKDKPIKIVIHDGKKFCYAPVFSGGESYIVIEQCWQMHVMNAR
AP6X1A_AP6.3612_AG3.iAP6-/1-373 ---------------MRHS-IVFIVVLLVLVTTSFEYSINNIHIHA--SSNTSNSIIQKPTDQPKDKPIKIVIHDGKKFCYAPVFSGGESYIVIEQCWQMHVMNAR
AP5X1A_AP5.3534_AG10.iAP5/1-373 ---------------MRHS-IVFIVVLLVLVTTSFEYSINNIHIHA--SSNTSNSIIQKPTDQPKDKPIKIVIHDGKKFCYAPVFSGGESYIVIEQCWQMHVMNAR
WP.061230224.1iUNK/1-635 ---------------MRHS-IVFIVVLLVLVTTSFEYSINNIHIHA--SSNTSNSIIQKPTDQPKDKPIKIVIHDGKKFCYAPVFSGGESYIVIEQCWQMHVMNAR
WP.192505408.1iUNK/1-635 ---------------MRHS-IVFIVVLLVLVTTSFEYSINNIHIHA--SSNTSNSIIQKPTDQPKDKPIKIVIHDGKKFCYAPVFSGGESYIVIEQCWQMHVMNAR
CLA.iS782_3731820/1-635 ---------------MRHS-IVFIVVLLVLVTTSFEYSINNIHIHA--SSNTSNSIIQKPTDQPKDKPIKIVIHDGKKFCYAPVFSGGESYIVIEQCWQMHVMNAR
WP.002105372.1iUNK/1-635 ---------------MRHS-IVFIVVLLVLVTTSFEYSINNIHIHA--SSNTSNSIIQKPTDQPKDKPIKIVIHDGKKFCYAPVFSGGESYIVIEQCWQMHVMNAR
EMM97995.1iZNI_sLT2156/1-635 ---------------MRHS-IVFIVVLLVLVTTSFEYSINNIHIHA--SSNTSNSIIQKPTDQPKDKPIKIVIHDGKKFCYAPVFSGGESYIVIEQCWQMHVMNAR
WP.001216432.1iUNK/1-635 ---------------MRHS-IVFIVVLLVLVTTSFEYSINNIHIHA--SSNTSNSIIQKPTDQPKDKPIKIVIHDGKKFCYAPVFSGGESYIVIEQCWQMHVMNAR
BAE.iS1548_3525207/1-635 ---------------MRHS-IVFIVVLLVLVTTSFEYSINNIHIHA--SSNTSNSIIQKPTDQPKDKPIKIVIHDGKKFCYAPVFSGGESYIVIEQCWQMHVMNAR
WP.061250523.1iUNK/1-635 ---------------MRHS-IVFIVVLLVLVTTSFEYSINNIHIHA--SSNTSNSIIQKPTDQPKDKPIKIVIHDGKKFCYAPVFSGGESYIVIEQCWQMHVMNAR
WP.061244769.1iUNK/1-635 ---------------MRHS-IVFIVVLLVLVTTSFEYSINNIHIHA--SSNTSNSIIQKPTDQPKDKPIKIVIHDGKKFCYAPVFSGGESYIVIEQCWQMHVMNAR
WP.001216431.1iUNK/1-635 ---------------MRHS-IVFIVVLLVLVTTSFEYSINNIHIHA--SSNTSNSIIQKPTDQPKDKPIKIVIHDGKKFCYAPVFSGGESYIVIEQCWQMHVMNAR
```

WP.041160711.1iUNK/1-635	---------------MRHS-IVFIIVLLVLVTTSFEYSINNIHIHA--SSNMSNSIIQKPTDQPKDKPIKIVIHDGKKFCYAPVFSGGESYIVIEQCWQMHVMNAR
WP.137170882.1iUNK/1-635	---------------MRHS-IVFIVVLLVLVTTSFEYSINNIHIHA--SSNTSNSIIQKPTDQPKDKPIKIVIHDGKKFCYGPTFSGGESYIVIEQCWQMHVTNAR
WP.137119336.1iUNK/1-635	---------------MRHS-IVFIVVLLVLVTTSFEYSINNIHIHA--SSNTSNSIIQKPTDQPKDKPIKIVIHDGKKFCYGPTFSGGESYIVIEQCWQMHVTNAR
WP.137108606.1iUNK/1-635	---------------MRHS-IVFIVVLLVLVTTSFEYSINNIHIHA--SSNTSNSIIQKPTDQPKDKPIKIVIHDGKKFCYGPTFSGGESYIVIEQCWQMHVTNAR
PN3Q14_SLPN3.874_AG3.iPN3/1-635	---------------MRHS-IVFIVVLLVLVTTSFEYSINNIHIHA--SSNTSNSIIQKPTDQPKDKPIKIVIHDGKKFCYGPTFSGGESYIVIEQCWQMHVTNAR
KW2Q14_KW2.797_AG3.iKW2-1/1-635	---------------MRHS-IVFIVVLLVLVTTSFEYSINNIHIHA--SSNTSNSIIQKPTDQPKDKPIKIVIHDGKKFCYGPTFSGGESYIVIEQCWQMHVTNAR
KW2Q14_KW2.780_AG3.iKW2-1/1-635	---------------MRHS-IVFIVVLLVLVTTSFEYSINNIHIHA--SSNTSNSIIQKPTDQPKDKPIKIVIHDGKKFCYGPTFSGGESYIVIEQCWQMHVTNAR
KW1Q1A_KW1.3358_AG3.iKW1-/1-635	---------------MRHS-IVFIVVLLVLVTTSFEYSINNIHIHA--SSNTSNSIIQKPTDQPKDKPIKIVIHDGKKFCYGPTFSGGESYIVIEQCWQMHVTNAR
AW1Q14_AW1.653_AG3.iAW1-1/1-635	---------------MRHS-IVFIVVLLVLVTTSFEYSINNIHIHA--SSNTSNSIIQKPTDQPKDKPIKIVIHDGKKFCYGPTFSGGESYIVIEQCWQMHVTNAR
AP7Q14_AP7.781_AG3.iAP7-1/1-635	---------------MRHS-IVFIVVLLVLVTTSFEYSINNIHIHA--SSNTSNSIIQKPTDQPKDKPIKIVIHDGKKFCYGPTFSGGESYIVIEQCWQMHVTNAR
AP3Q14_AP3.874_AG10.iAP3-/1-635	---------------MRHS-IVFIVVLLVLVTTSFEYSINNIHIHA--SSNTSNSIIQKPTDQPKDKPIKIVIHDGKKFCYGPTFSGGESYIVIEQCWQMHVTNAR
AP1Q13_AP1.1310_AG3.iAP1-/1-635	---------------MRHS-IVFIVVLLVLVTTSFEYSINNIHIHA--SSNTSNSIIQKPTDQPKDKPIKIVIHDGKKFCYGPTFSGGESYIVIEQCWQMHVTNAR
AG3Q14_U1.874_AG3.iO73718/1-635	---------------MRHS-IVFIVVLLVLVTTSFEYSINNIHIHA--SSNTSNSIIQKPTDQPKDKPIKIVIHDGKKFCYGPTFSGGESYIVIEQCWQMHVTNAR
WP.025179757.1iUNK/1-635	---------------MRHS-IVFIIVLLVLVTTSFEYSINNIHIHA--SSNMSNSIIQKPTDQPKDKPIKIVIHDGKKFCYAPVFSGGESYIVIEQCWQMHVMNAR
WP.001216424.1iUNK/1-635	---------------MRHS-IVFIVVLLVLVTTSFEYSINNIHIHA--SSNTSNSIIQKPTDQPKDKPIKIVIHDGGKFCYGPVFSGGESYIIIEQCWQMHVMNAR
UNK.iR7_3404575/1-635	---------------MRHS-IVFIVVLLVLVTTSFEYSINNIHIHA--SSNTSNSIIQKPTDQPKDKPIKIVIHDGGKFCYGPVFSGGESYIIIEQCWQMHVMNAR
UNK.iR22_3414280/1-635	---------------MRHS-IVFIVVLLVLVTTSFEYSINNIHIHA--SSNTSNSIIQKPTDQPKDKPIKIVIHDGGKFCYGPVFSGGESYIIIEQCWQMHVMNAR
UNK.iR21_3415571/1-635	---------------MRHS-IVFIVVLLVLVTTSFEYSINNIHIHA--SSNTSNSIIQKPTDQPKDKPIKIVIHDGGKFCYGPVFSGGESYIIIEQCWQMHVMNAR
UNK.iR19_3414213/1-635	---------------MRHS-IVFIVVLLVLVTTSFEYSINNIHIHA--SSNTSNSIIQKPTDQPKDKPIKIVIHDGGKFCYGPVFSGGESYIIIEQCWQMHVMNAR
UNK.iR17_3414922/1-635	---------------MRHS-IVFIVVLLVLVTTSFEYSINNIHIHA--SSNTSNSIIQKPTDQPKDKPIKIVIHDGGKFCYGPVFSGGESYIIIEQCWQMHVMNAR
UNK.iR16_3405117/1-635	---------------MRHS-IVFIVVLLVLVTTSFEYSINNIHIHA--SSNTSNSIIQKPTDQPKDKPIKIVIHDGGKFCYGPVFSGGESYIIIEQCWQMHVMNAR
UNK.iR13_3405389/1-635	---------------MRHS-IVFIVVLLVLVTTSFEYSINNIHIHA--SSNTSNSIIQKPTDQPKDKPIKIVIHDGGKFCYGPVFSGGESYIIIEQCWQMHVMNAR
UNK.iR11_3405858/1-635	---------------MRHS-IVFIVVLLVLVTTSFEYSINNIHIHA--SSNTSNSIIQKPTDQPKDKPIKIVIHDGGKFCYGPVFSGGESYIIIEQCWQMHVMNAR
RT2Q14_RT2.788_AG3.iRT2-1/1-635	---------------MRHS-IVFIVVLLVLVTTSFEYSINNIHIHA--SSNTSNSIIQKPTDQPKDKPIKIVIHDGKKFCYGPTFSGGESYIVIEQCWQMHVTNAR
LAI.iS56601_779765/1-635	---------------MRHS-IVFIIVLLVLVTTSFEYSINNIHIHA--SSNMSNSIIQKPTDQPKDKPIKIVIHDGKKFCYAPVFSGGESYIVIEQCWQMHVMNAR
LAI.iIPAV_779157/1-635	---------------MRHS-IVFIIVLLVLVTTSFEYSINNIHIHA--SSNMSNSIIQKPTDQPKDKPIKIVIHDGKKFCYAPVFSGGESYIVIEQCWQMHVMNAR
ICT.iS898_3451851/1-635	---------------MRHS-IVFIVVLLVLVTTSFEYSINNIHIHA--SSNTSNSIIQKPTDQPKDKPIKIVIHDGGKFCYGPVFSGGESYIIIEQCWQMHVMNAR
ICT.iPiscina_3408322/1-635	---------------MRHS-IVFIVVLLVLVTTSFEYSINNIHIHA--SSNTSNSIIQKPTDQPKDKPIKIVIHDGGKFCYGPVFSGGESYIIIEQCWQMHVMNAR
EKR55926.1iUNK_sUI12758/1-602	---mSNSIIQKPTDQPKDKPIKIVIHDGKKFCYAPVFSGGESYIVIEQCWQMHVMNAR
COP.iSK1_3451842/1-635	---------------MRHS-IVFIVVLLVLVTTSFEYSINNIHIHA--SSNTSNSIIQKPTDQPKDKPIKIVIHDGGKFCYGPVFSGGESYIIIEQCWQMHVMNAR
COP.iL1130_Q72NJ0.cl3451/1-635	---------------MRHS-IVFIVVLLVLVTTSFEYSINNIHIHA--SSNTSNSIIQKPTDQPKDKPIKIVIHDGGKFCYGPVFSGGESYIIIEQCWQMHVMNAR
COP.iFDAARGOS203_1461397/1-635	---------------MRHS-IVFIVVLLVLVTTSFEYSINNIHIHA--SSNTSNSIIQKPTDQPKDKPIKIVIHDGGKFCYGPVFSGGESYIIIEQCWQMHVMNAR
BAE.iS1489_3559139/1-635	---------------MRHS-IVFIIVLLVLVTTSFEYSINNIHIHA--SSNMSNSIIQKPTDQPKDKPIKIVIHDGKKFCYAPVFSGGESYIVIEQCWQMHVMNAR
AAS71397.1iCOP_sL1130/1-639	-----------meaNMRHS-IVFIVVLLVLVTTSFEYSINNIHIHA--SSNTSNSIIQKPTDQPKDKPIKIVIHDGGKFCYGPVFSGGESYIIIEQCWQMHVMNAR
AAN47968.2iLAI_s56601/1-602	---mSNSIIQKPTDQPKDKPIKIVIHDGKKFCYAPVFSGGESYIVIEQCWQMHVMNAR
WP.017855280.1iUNK/1-635	---------------MRHS-IVFIVVLLVLVTTSFEYSINNIHIHA--SSNTSNSIIQKPTDQPKDKPIKIVIHDGGKFCYGPVFSGGESYIIIEQCWQMHVMNAR
UNK.iRCA_3869691/1-589	---------------MRHS-IVFIVVLLVLVTTSFEYSINNIHIHA--SSNTSNSIIQKPTDQPKDKPIKIVIHDGGKFCYGPVFSGGESYIIIEQCWQMHVMNAR
WP.017855335.1iUNK/1-634	---------------MRHS-IVFIVVLLVLVTTSFEYSINNIHIHA--SSNTSNSIIQKPTDQPKDKPIKIVIHDGGKFCYGPVFSGGESYIIIEQCWQMHVMNAR
WP.002156208.1iUNK/1-635	---------------MRHS-IVFIVVLLVLVTTSFEYSINNIHIHA--SSNTSNSIIQKPTDQPKDKPIKIVIHDGGKFCYGPVFSGGESYIIIEQCWQMHVMNAR
EMM81387.1iUNK_s200600185/1-635	---------------MRHS-IVFIVVLLVLVTTSFEYSINNIHIHA--SSNMSNSIIQKPTDQPKDKPIKIVIHDGKKFCYAPVFSGGESYIVIEQCWQMHVMNAR
WP.002125661.1iUNK/1-635	---------------MRHS-IVFIVVLLVLVTTSFEYSINNIHIHA--SSNTSNSIIQKPTDQPKDKPIKIVIHDGKKFCYAPVFSGGESYIVIEQCWQMHVMNAR
WP.002081486.1iUNK/1-635	---------------MRHS-IVFIIVLLVLVTTSFEYSINNIHIHA--SSNMSNSIIQKPTDQPKDKPIKIVIHDGKKFCYAPVFSGGESYIVIEQCWQMHVMNAR
EMM89333.1iDAN_sLT1649/1-602	---mSNSIIQKPTDQPKDKPIKIVIHDGKKFCYAPVFSGGESYIVIEQCWQMHVMNAR
WP.002183563.1iUNK/1-635	---------------MRHS-IVFIVVLLVLVTTSFEYSINNIHIHA--SSNTSNSIIQKPTDQPKDKPIKIVIHDGGKFCYGPVFSGGESYIIIEQCWQMHVMNAR
WP.001216426.1iUNK/1-635	---------------MRHS-IVFIVVLLVLVTTSFEYSINNIHIHA--SSNTSNSIIQKPTDQPKDKPIKIVIHDGGKFCYGPVFSGGESYIIIEQCWQMHVMNAR
PN2Q14_PN2.1751_AG14.iPN2/1-635	---------------MRHS-IVFIVVLLVLVTTSFEYSINNIHIHA--SSNTSNSIIQKPTDQPKDKPIKIVIHDGGKFCYGPVFSGGESYIIIEQCWQMHVMNAR
KG1Q1D_KG1.4744_AG14.uKG1/1-635	---------------MRHS-IVFIVVLLVLVTTSFEYSINNIHIHA--SSNTSNSIIQKPTDQPKDKPIKIVIHDGGKFCYGPVFSGGESYIIIEQCWQMHVMNAR
CLA.iS611_3427313/1-635	---------------MRHS-IVFIVVLLVLVTTSFEYSINNIHIHA--SSNTSNSIIQKPTDQPKDKPIKIVIHDGGKFCYGPVFSGGESYIIIEQCWQMHVMNAR
CLA.iS114_857715/1-635	---------------MRHS-IVFIVVLLVLVTTSFEYSINNIHIHA--SSNTSNSIIQKPTDQPKDKPIKIVIHDGGKFCYGPVFSGGESYIIIEQCWQMHVMNAR
CLA.iLJ178_3432200/1-635	---------------MRHS-IVFIVVLLVLVTTSFEYSINNIHIHA--SSNTSNSIIQKPTDQPKDKPIKIVIHDGGKFCYGPVFSGGESYIIIEQCWQMHVMNAR
EMN50810.1iUNK_sL1207/1-635	---------------MRHS-IVFIVVLLVLVTTSFEYSINNIHIHA--SSNTSNSIIQKPTDQPKDKPIKIVIHDGGKFCYGPVFSGGESYIIIEQCWQMHVMNAR
WP.017856587.1iUNK/1-635	---------------MRHS-IVFIVVLLVLVTTSFEYSINNIHIHA--SSNTSNSIIQKPTDQPKDKPIKIVIHDGGKFCYGPVFSGGESYIIIEQCWQMHVMNAR
WP.017857335.1iUNK/1-635	---------------MRHS-IVFIVVLLVLVTTSFEYSINNIHIHA--SSNTSNSIIQKPTDQPKDKPIKIVIHDGGKFCYGPVFSGGESYIIIEQCWQMHVMNAR
CLA.iRUFN_2965845/1-632	---------------MRHS-IVFIVVLLVLVTTSFEYSINNIHIHA--SSNTSNSIIQKPTDQPKDKPIKIVIHDGGKFCYGPVFSGGESYIIIEQCWQMHVMNAR
WP.137108578.1iUNK/1-638	---------------MCNWKKIFIVVLLVLIGFGFEYGMNPTPVDA--SSKIEYSVIQKPTDPPKDKPIKVIVSDGGKFCYGPNFSGGESYIIIEQCWQMHVTNAR
KW1Q18_KW1.3195_AG3.iKW1-/1-638	---------------MCNWKKIFIVVLLVLIGFGFEYGMNPTPVDA--SSKIEYSVIQKPTDPPKDKPIKVIVSDGGKFCYGPNFSGGESYIIIEQCWQMHVTNAR
WP.137108137.1iUNK/1-636	---------------MRNWKKMFMIVLLVLIGFGLEYGMNPTPVDA--SSKIEYSVIQKPTDPPKDKPIKIVIHDGGKFCYGPVFSGGESYIIIEQCWQMHVTNAR
RT2Q1A_RT2.3164_AG3.iRT2-/1-636	---------------MRNWKKMFMIVLLVLIGFGLEYGMNPTPVDA--SSKIEYSVIQKPTDPPKDKPIKIVIHDGGKFCYGPVFSGGESYIIIEQCWQMHVTNAR
PN3Q1A_SLPN3.3202_AG3.iPN/1-636	---------------MRNWKKMFMIVLLVLIGFGLEYGMNPTPVDA--SSKIEYSVIQKPTDPPKDKPIKIVIHDGGKFCYGPVFSGGESYIIIEQCWQMHVTNAR
PD1Q18_PD1.3693_AG3.iPD1-/1-636	---------------MRNWKKMFMIVLLVLIGFGLEYGMNPTPVDA--SSKIEYSVIQKPTDPPKDKPIKIVIHDGGKFCYGPVFSGGESYIIIEQCWQMHVTNAR
KW2Q1A_KW2.3128_AG3.iKW2-/1-636	---------------MRNWKKMFMIVLLVLIGFGLEYGMNPTPVDA--SSKIEYSVIQKPTDPPKDKPIKIVIHDGGKFCYGPVFSGGESYIIIEQCWQMHVTNAR
KW2Q1A_KW2.3110_AG3.iKW2-/1-636	---------------MRNWKKMFMIVLLVLIGFGLEYGMNPTPVDA--SSKIEYSVIQKPTDPPKDKPIKIVIHDGGKFCYGPVFSGGESYIIIEQCWQMHVTNAR
KW1Q14_KW1.1030_AG3.iKW1-/1-636	---------------MRNWKKMFMIVLLVLIGFGLEYGMNPTPVDA--SSKIEYSVIQKPTDPPKDKPIKIVIHDGGKFCYGPVFSGGESYIIIEQCWQMHVTNAR
KG2Q1A_KG2.4176_AG3.uKG2-/1-636	---------------MRNWKKMFMIVLLVLIGFGLEYGMNPTPVDA--SSKIEYSVIQKPTDPPKDKPIKIVIHDGGKFCYGPVFSGGESYIIIEQCWQMHVTNAR
AP7Q1A_AP7.3101_AG3.iAP7-/1-636	---------------MRNWKKMFMIVLLVLIGFGLEYGMNPTPVDA--SSKIEYSVIQKPTDPPKDKPIKIVIHDGGKFCYGPVFSGGESYIIIEQCWQMHVTNAR
AP3Q1A_AP3.3202_AG10.iAP3/1-636	---------------MRNWKKMFMIVLLVLIGFGLEYGMNPTPVDA--SSKIEYSVIQKPTDPPKDKPIKIVIHDGGKFCYGPVFSGGESYIIIEQCWQMHVTNAR
AP1Q19_AP1.3361_AG3.iAP1-/1-636	---------------MRNWKKMFMIVLLVLIGFGLEYGMNPTPVDA--SSKIEYSVIQKPTDPPKDKPIKIVIHDGGKFCYGPVFSGGESYIIIEQCWQMHVTNAR
AG3Q1A_U1.3202_AG3.iO7371/1-636	---------------MRNWKKMFMIVLLVLIGFGLEYGMNPTPVDA--SSKIEYSVIQKPTDPPKDKPIKIVIHDGGKFCYGPVFSGGESYIIIEQCWQMHVTNAR
WP.025176475.1iUNK/1-636	---------------MRNWKKVSMIVLLVLIGFGFEYGMNPTPVDA--SSKIEYLVIQKPTDPPKDQPIKVIVSGEGKFCYGPDFSGGESYIIIEQCWQMHVMNAR
WP.000336234.1iUNK/1-636	---------------MCNWKKIFIVVLLVLIGFGFEYGINPTPVDA--SSKIEYLVIQKPTDPPKDQPIKVIVSGEGKFCYGPDFSGGESYIIIEQCWQMHVMNAR
WP.001246821.1iUNK/1-636	---------------MRNWKKVSMIVLLVLIGFGFEYGMNPTPVDA--SSKIEYLVIQKPTDPPKDQPIKVIVSGEGKFCYGPDFSGGESYIIIEQCWQMHVMNAR
WP.192505542.1iUNK/1-636	---------------MRNWKKIFIVVLLVLIGFGLEYGMNPTPVDA--SSKIEYSVIQKPTDPPKDKPIKVIVSGEGKFCYGPDFSGGESYIIIEQCWQMHVMNAR
WP.192503327.1iUNK/1-636	---------------MRNWKKVFMIVLLVLIGFGLEYGINPTPVDA--SSKIEYSVIQKPTDPPKDKPIKVIVSGEGKFCYGPDFSGGESYIIIEQCWQMHVMNAR
WP.176484996.1iUNK/1-619	---------------mirg-----------------YVXNPTPVDA--SSKIEYSVIQKPTDPPKDKPIKVIVSGEGKFCYGPDFSGGESYIIIEQCWQMHVMNAR
WP.137119396.1iUNK/1-636	---------------MRNWKKVFMIVLLVLIGFGLEYGINPTPVDA--SSKIEYLVIQKPTDPPKDKPIKVIVSGEGKFCYGPDFSGGESYIIIEQCWQMHVMNAR
WP.105096134.1iUNK/1-636	---------------MCNWKKIFIVVLLVLIGFGFEYGINPTPVDA--SSKIEYLVIQKPTDPPKDQPIKVIVSGEGKFCYGPDFSGGESYIIIEQCWQMHVMNAR
WP.025185261.1iUNK/1-636	---------------MRNWKKVFMIVLLVLIGFGLEYGINPTPVDA--SSKIEYSVIQKPTDPPKDKPIKVIVSGEGKFCYGPDFSGGESYIIIEQCWQMHVMNAR
WP.025180336.1iUNK/1-636	---------------MRNWKKIFIVVLLVLIGFGLEYGMNPTPVDA--SSKIEYSVIQKPTDPPKDQPIKVIVSGEGKFCYGPDFSGGESYIIIEQCWQMHVMNAR
WP.025180297.1iUNK/1-638	---------------MYNWKKILIVVLLASIMVYLEYEMDHTLVHAASSSKTTNSIVQKPTDPPKDKPIKVNVSGGGTFCYGPNFSGGESYIIIEQCWQMHVMNAR
WP.002127783.1iUNK/1-636	---------------MRNWKKVFMIVLLVLIGFGLEYGINPTPVDA--SSKIEYSVIQKPTDPPKDKPIKVIVSGEGKFCYGPDFSGGESYIIIEQCWQMHVMNAR
WP.002121904.1iUNK/1-636	---------------MRNWKKVFMIVLLVLIGFGLEYGINPTPVDA--SSKIEYSVIQKPTDPPKDQPIKVIVSDGGKFCYGPDFSGGESYIIIEQCWQMHVMNAR
WP.001246824.1iUNK/1-636	---------------MRNWKKVSMIVLLVLIGFGLEYGINPTPVDA--SSKIEYSVIQKPTDPPKDKPIKVIVSGEGKFCYGPDFSGGESYIIIEQCWQMHVMNAR
WP.001246820.1iUNK/1-636	---------------MRNWKKVSMIVLLVLIGFGFEYGMNPTPVDA--SSKIEYLVIQKPTDPPKDQPIKVIVSGEGKFCYGPDFSGGESYIIIEQCWQMHVMNAR
WP.001246818.1iUNK/1-636	---------------MRNWKKVFMIVLLVLIGFGLEYGINPTPVDA--SSKIEYSVIQKPTDPPKDKPIKVIVSGEGKFCYGPDFSGGESYIIIEQCWQMHVMNAR
WP.000336233.1iUNK/1-636	---------------MCNWKKIFIVVLLVLIGFGFEYGINPTPVDA--SSKIEYLVIQKPTDPPKDQPIKVIVSGEGKFCYGPDFSGGESYIIIEQCWQMHVMNAR
WP.000280008.1iUNK/1-635	---------------MYKW-KVLTAFFLISIGSGFEYGVNHTHIHA--LSKIEYSVIQKPTDPPKDKPIKVIVSDGGKFCYGPNFSGGESYIIIEQCWQMHVMNAR
UNK.iRCA_1517495/1-636	---------------MRNWKKVSMIVLLVLIGFGFEYGINPTPVDA--SSKIEYLVIQKPTDPPKDQPIKVIVSGEGKFCYGPDFSGGESYIIIEQCWQMHVMNAR
PCE70240.1iUNK_pCFV95.186/1-618	---------------mrg------------------YVXNPTPVDA--SSKIEYSVIQKPTDPPKDKPIKVIVSGEGKFCYGPDFSGGESYIIIEQCWQMHVMNAR
LHI.iS56609_1089290/1-636	---------------MRNWKKVFMIVLLVLIGFGLEYGINPTPVDA--SSKIEYSVIQKPTDPPKDKPIKVIVSGEGKFCYGPDFSGGESYIIIEQCWQMHVMNAR
LAI.iS56601_3246739/1-636	---------------MRNWKKVFMIVLLVLIGFGLEYGINPTPVDA--SSKIEYSVIQKPTDPPKDKPIKVIVSGEGKFCYGPDFSGGESYIIIEQCWQMHVMNAR
LAI.iIPAV_3243838/1-636	---------------MRNWKKVFMIVLLVLIGFGLEYGINPTPVDA--SSKIEYSVIQKPTDPPKDKPIKVIVSGEGKFCYGPDFSGGESYIIIEQCWQMHVMNAR
ICT.iS898_1052237/1-636	---------------MRNWKKVSMIVLLVLIGFGFEYGMNPTPVDA--SSKIEYLVIQKPTDPPKDQPIKVIVSGEGKFCYGPDFSGGESYIIIEQCWQMHVMNAR
ICT.iPiscina_1060929/1-636	---------------MRNWKKVSMIVLLVLIGFGFEYGMNPTPVDA--SSKIEYLVIQKPTDPPKDQPIKVIVSGEGKFCYGPDFSGGESYIIIEQCWQMHVMNAR
ICT.iLangkawi_1108981/1-636	---------------MRNWKKVFMIVLLVLIGFGLEYGINPTPVDA--SSKIEYSVIQKPTDPPKDKPIKVIVSGEGKFCYGPDFSGGESYIIIEQCWQMHVMNAR
EMP05347.1iPES_s200701872/1-628	-----------------------MIVLLVLIGFGLEYGINPTPVDA--SSKIEYSVIQKPTDPPKDKPIKVIVSGEGKFCYGPDFSGGESYIIIEQCWQMHVMNAR
EMO07338.1iICT_sVerdunHP/1-628	-----------------------MIVLLVLIGFGFEYGMNPTPVDA--SSKIEYLVIQKPTDPPKDQPIKVIVSGEGKFCYGPDFSGGESYIIIEQCWQMHVMNAR
EMN67929.1iGSA_sUI08434/1-628	-----------------------MIVLLVLIGFGLEYGINPTPVDA--SSKIEYSVIQKPTDPPKDKPIKVIVSGEGKFCYGPDFSGGESYIIIEQCWQMHVMNAR
EMF70973.1iCLA_sLT1962/1-630	---------------------mFMIVLLVLIGFGLEYGINPTPVDA--SSKIEYSVIQKPTDPPKDKPIKVIVSGEGKFCYGPDFSGGESYIIIEQCWQMHVMNAR
EMF41663.1iLRA_sTE1992/1-630	---------------------mSMIVLLVLIGFGLEYGINPTPVDA--SSKIEYSVIQKPTDPPKDKPIKVIVSGEGKFCYGPDFSGGESYIIIEQCWQMHVMNAR
EKR18842.1iPES_s200600696/1-613	--------------------------------------MNPTPVDA--SSKIEYSVIQKPTDPPKDQPIKVIVSGEGKFCYGPDFSGGESYIIIEQCWQMHVMNAR
EKQ49790.1iUNK_s200200062/1-615	--------------------------------------MDHTLVHAASSSKTTNSIVQKPTDPPKDKPIKVNVSGGGTFCYGPNFSGGESYIIIEQCWQMHVMNAR
EKP22627.1iICT_sVerdunLP/1-613	--------------------------------------MNPTPVDA--SSKIEYLVIQKPTDPPKDQPIKVIVSGEGKFCYGPDFSGGESYIIIEQCWQMHVMNAR
COP.iSK1_1052259/1-636	---------------MRNWKKVSMIVLLVLIGFGFEYGMNPTPVDA--SSKIEYLVIQKPTDPPKDQPIKVIVSGEGKFCYGPDFSGGESYIIIEQCWQMHVMNAR
COP.iL1130_Q72TZ4.cl1052/1-636	---------------MRNWKKVSMIVLLVLIGFGFEYGMNPTPVDA--SSKIEYLVIQKPTDPPKDQPIKVIVSGEGKFCYGPDFSGGESYIIIEQCWQMHVMNAR
COP.iFDAARGOS203_3342350/1-636	---------------MRNWKKVSMIVLLVLIGFGFEYGMNPTPVDA--SSKIEYLVIQKPTDPPKDQPIKVIVSGEGKFCYGPDFSGGESYIIIEQCWQMHVMNAR
CLA.iS782_1025581/1-636	---------------MRNWKKIFIVVLLVLIGFGLEYGMNPTPVDA--SSKIEYSVIQKPTDPPKDKPIKVIVSGEGKFCYGPDFSGGESYIIIEQCWQMHVMNAR

BVA.iPigK151_3389157/1-636	---------------MRNWKKVSMIVLLVLIGFGLEYGINPTPVDA--SSKIEYSVIQKPTDPPKDKPIKVIVSGEGKFCYGPDFSGGESYIIIEQCWQMHVMNAR
AW3Q14_AW3.1483_AG10.iAW3/1-633	---------------MRNWKKVFMIVLLVLIGFGLEYGINPTPVDA--SSKIEYSVIQKPTDPPKDK---VIVSGEGKFCYGPDFSGGESYIIIEQCWQMHVMNAR
AW1Q1A_AW1.3141_AG3.iAW1-/1-636	---------------MRNWKKVFMIVLLVLIGFGLEYGINPTPVDA--SSKIEYLVIQKPTDPPKDKPIKVIVSGEGKFCYGPDFSGGESYIIIEQCWQMHVMNAR
ASP41721.1iUNK_pAMR47.071/1-636	---------------MRNWKKVSMIVLLVLIGFGFEYGINPTPVDA--SSKIEYLVIQKPTDPPKDQPIKVIVSGEGKFCYGPDFSGGESYIIIEQCWQMHVMNAR
AP6Q14_AP6.1189_AG3.iAP6-/1-636	---------------MRNWKKVFMIVLLVLIGFGLEYGINPTPVDA--SSKIEYSVIQKPTDPPKDKPIKVIVSGEGKFCYGPDFSGGESYIIIEQCWQMHVMNAR
AP5Q14_AP5.1135_AG10.iAP5/1-636	---------------MRNWKKVFMIVLLVLIGFGLEYGINPTPVDA--SSKIEYSVIQKPTDPPKDKPIKVIVSGEGKFCYGPDFSGGESYIIIEQCWQMHVMNAR
WP.082267517.1iUNK/1-630	---------------MRNWKKVFMIVLLVLIGFGLEYGINPTPVDA--SSKIEYSVIQKPTDPPKDKPIKVIVSGEGKFCYGPDFSGGESYIIIEQCWQMHVMNAR
WP.002101894.1iUNK/1-636	---------------MRNWKKIFIVVLLVLIGFGLEYGINPTPVDA--SSKIEYSVIQKPTDPPKDKPIKVIVSGEGKFCYGPDFSGGESYIIIEQCWQMHVMNAR
WP.082283173.1iUNK/1-636	---------------MRNWKKIFIVVLLVLIGFGLEYGMNPTPVDA--SSKIEYSVIQKPTDPPKDKPIKVIVSGEGKFCYGPDFSGGESYIIIEQCWQMHVMNAR
HJO.iL53_1091748/1-636	---------------MRNWKKIFIVVLLVLIGFGLEYGMNPTPVDA--SSKIEYSVIQKPTDPPKDKPIKVIVSGEGKFCYGPDFSGGESYIIIEQCWQMHVMNAR
WP.192502085.1iUNK/1-636	---------------MRNWKKVFMIVLLVLIGFGLEYGMNPTPVDA--SSKIEYLVIQKPTDPPKDQPIKVIVSGEGKFCYGPDFSGGESYIIIEQCWQMHVMNAR
BAE.iS1548_1111136/1-636	---------------MRNWKKVFMIVLLVLIGFGLEYGMNPTPVDA--SSKIEYLVIQKPTDPPKDQPIKVIVSGEGKFCYGPDFSGGESYIIIEQCWQMHVMNAR
WP.025177628.1iUNK/1-636	---------------MRNWKKIFIVVLLVLIGFGLEYGMNPTPVDA--SSKIEYSVIQKPTDPPKDKPIKVIVSDEGKFCYGPDFSGGESYIIIEQCWQMHVMNAR
EKO98983.1iUNK_sBrem329/1-613	--------------------------------------MNPTPVDA--SSKIEYSVIQKPTDPPKDKPIKVIVSDEGKFCYGPDFSGGESYIIIEQCWQMHVMNAR
WP.192486215.1iUNK/1-636	---------------MRNWKKIFIVVLLVLIGFGLEYGINPTPVDA--SSKIEYSVIQKPTDPPKDKPIKVIVSGEGKFCYGPDFSGGESYIIIEQCWQMHVMNAR
WP.017857166.1iUNK/1-635	---------------MRHS-IVFIVVLLVLVTTSFEYSINNIHIHA--SSNTSNSIIQKPTDQPKDKPIKIVIHDGGKFCYGPVFSGGESYIIIEQCWQMHVMNAR
WP.082284627.1iUNK/1-606	--m-EYLVIQKPTDPPKDKPIKVIVSGEGKFCYGPNFSGGESYIIIEQCWQMHVMNAR
WP.082279653.1iUNK/1-604	---------------m------------------------------------EYLVIQKPTDPPKDQPIKVIVSGEGKFCYGPDFSGGESYIIIEQCWQMHVMNAR
WP.203368306.1iUNK/1-638	---------------MCNWKKIFIVVLLVLIGFGFEYGINPTPVDA--SSKIEYLVIQKPTDPPKDKPIKVIVSGEGKFCYGPDFSGGESYIIIEQCWQMHVMNAR
WP.137170899.1iUNK/1-638	---------------MCNWKKIFIVVLLVLIGFGFEYGMNPTPVDA--SSKIEYLVIQKPTDPPKDKPIKVIVRGEGKFCYGPDFSGGESYIIIEQCWQMHVMNAR
WP.137119341.1iUNK/1-638	---------------MCNWKKIFIVVLLVLIGFGFEYGMNPTPVDA--SSKIEYLVIQKPTDPPKDKPIKVIVSGEGKFCYGPDFSGGESYIIIEQCWQMHVMNAR
RT2Q16_RT2.955_AG3.iRT2-1/1-638	---------------MCNWKKIFIVVLLVLIGFGFEYGMNPTPVDA--SSKIEYLVIQKPTDPPKDKPIKVIVSGEGKFCYGPDFSGGESYIIIEQCWQMHVMNAR
KW2Q16_KW2.960_AG3.iKW2-1/1-638	---------------MCNWKKIFIVVLLVLIGFGFEYGMNPTPVDA--SSKIEYLVIQKPTDPPKDKPIKVIVRGEGKFCYGPDFSGGESYIIIEQCWQMHVMNAR
KW2Q16_KW2.942_AG3.iKW2-1/1-638	---------------MCNWKKIFIVVLLVLIGFGFEYGMNPTPVDA--SSKIEYLVIQKPTDPPKDKPIKVIVRGEGKFCYGPDFSGGESYIIIEQCWQMHVMNAR
KAA1291412.1iGRA/1-638	---------------MCNWKKIFIVVLLVLIGFGFEYGMNPTPVDA--SSKIEYLVIQKPTDPPKDKPIKVIVRGEGKFCYGPDFSGGESYIIIEQCWQMHVMNAR
AW1Q16_AW1.821_AG3.iAW1-1/1-638	---------------MCNWKKIFIVVLLVLIGFGFEYGMNPTPVDA--SSKIEYLVIQKPTDPPKDKPIKVIVSGEGKFCYGPDFSGGESYIIIEQCWQMHVMNAR
WP.061245996.1iUNK/1-636	---------------MRNWKKVFIVILLVLIGFGFEYGMNPTPVDA--SSRIEYSVIQKPTDPPKDKPIKIVIHDGGRFCYGPVFSGDESYIIIEQCWQMHVMNAR
WP.002100667.1iUNK/1-636	---------------MRNWKKVFIVILLVLIGFGFEYGMNPTLVDA--SSRIEYSVIQKPTDPPKDKPIKIVIHDGGRFCYGPVFSGDESYIIIEQCWQMHVMNAR
PN2Q1A_PN2.4122_AG14.iPN2/1-636	---------------MRNWKKVFIVILLVLIGFGFEYGMNPTPVDA--SSRIEYSVIQKPTDPPKDKPIKIVIHDGGRFCYGPVFSGDESYIIIEQCWQMHVMNAR
KG1Q17_KG1.2197_AG14.uKG1/1-636	---------------MRNWKKVFIVILLVLIGFGFEYGMNPTPVDA--SSRIEYSVIQKPTDPPKDKPIKIVIHDGGRFCYGPVFSGDESYIIIEQCWQMHVMNAR
WP.141622011.1iUNK/1-638	---------------MCNWKKIFIVVLLVLIGFGFEYGINPTPVDA--SSKIEYLVIQKPTDPPKDQPIKVIVSGGGKFCYGPNFSGGESYIIIEQCWQMHVMNAR
WP.057141815.1iUNK/1-636	---------------MCNWKKIFIVVLLVLIGFGFEYGMNPTPVDA--SSKIEYSVIQKPTDPPKDKPIKVIVSDGGKFCYGPDFSGGESYIIIEQCWQMHVTNAR
WP.002116238.1iUNK/1-638	---------------MRNWKKVFIVILLVLIGFGFEYGMNPTPVDA--SSRIEYSVIQKPTDPPKDKPIKIVIHDGGRFCYGPVFSGDESYIIIEQCWQMHVMNAR
CLA.iS611_1037225/1-636	---------------MRNWKKVFIVILLVLIGFGFEYGMNPTPVDA--SSRIEYSVIQKPTDPPKDKPIKIVIHDGGRFCYGPVFSGDESYIIIEQCWQMHVMNAR
CLA.iLJ178_1042589/1-638	---------------MRNWKKVFIVILLVLIGFGFEYGMNPTPVDA--SSRIEYSVIQKPTDPPKDKPIKIVIHDGGRFCYGPVFSGDESYIIIEQCWQMHVMNAR
WP.061246153.1iUNK/1-638	---------------MCNWKKIFIVVLLVLIGFGFEYGMNPTPVDA--SSKIEYSVIQKPTDPPKDKPIKVIVSDGGKFCYGPDFSGGESYIIIEQCWQMHVTNAR
WP.002111049.1iUNK/1-638	---------------MRNWKKVFIVILLVLIGFGFEYGMNPTLVDA--SSRIEYSVIQKPTDPPKDKPIKIVIHDGGRFCYGPVFSGDESYIIIEQCWQMHVMNAR
WP.002107685.1iUNK/1-638	---------------MCNWKKIFIVVLLVLIGFGFEYGINPTPVDA--SSKIEYSVIQKPTDPPKDKPIKVIVSDGGKFCYGPDFSGGESYIIIEQCWQMHVTNAR
PN2Q16_PN2.1919_AG14.iPN2/1-638	---------------MCNWKKIFIVVLLVLIGFGFEYGMNPTPVDA--SSKIEYSVIQKPTDPPKDKPIKVIVSDGGKFCYGPDFSGGESYIIIEQCWQMHVTNAR
WP.001246823.1iUNK/1-636	---------------MRNWKKVSMIVLLVLIGFGFEYGMNPTPVDA--SSKIEYLVIQKPTDPPKDQPIKVIVSGEGKFCYGPDFSGGESYIIIEQCWQMHVMNAR
UNK.iR12_3411515/1-635	---------------MRHS-IVFIVVLLVLVTTSFEYSINNIHIHA--SSNTSNSIIQKPTDQPKDKPIKIVIHDGGKFCYGPVFSGGESYIIIEQCWQMHVMNAR
QHH72113.1iUNK/1-635	---------------MRHS-IVFIVVLLVLVTTSFEYSINNIHIHA--SSNTSNSIIQKPTDQPKDKPIKIVIHDGGKFCYGPVFSGGESYIIIEQCWQMHVMNAR
UNK.iR12_1037975/1-636	---------------MRNWKKVSMIVLLVLIGFGFEYGMNPTPVDA--SSKIEYLVIQKPTDPPKDQPIKVIVSGEGKFCYGPDFSGGESYIIIEQCWQMHVMNAR
QHH70335.1iUNK/1-636	---------------MRNWKKVSMIVLLVLIGFGFEYGMNPTPVDA--SSKIEYLVIQKPTDPPKDQPIKVIVSGEGKFCYGPDFSGGESYIIIEQCWQMHVMNAR
UNK.iR12_3266082/1-638	---------------MCNWKKIFIVVLLVLIGFGFEYGINPTPVDA--SSKIEYLVIQKPTDPPKDQPIKVIVSGEGKFCYGPDFSGGESYIIIEQCWQMHVMNAR
QHH71994.1iUNK/1-638	---------------MCNWKKIFIVVLLVLIGFGFEYGINPTPVDA--SSKIEYLVIQKPTDPPKDQPIKVIVSGEGKFCYGPDFSGGESYIIIEQCWQMHVMNAR
UNK.iR13L_1037023/1-636	---------------MRNWKKVSMIVLLVLIGFGFEYGMNPTPVDA--SSKIEYLVIQKPTDPPKDQPIKVIVSGEGKFCYGPDFSGGESYIIIEQCWQMHVMNAR
QHH27625.1iUNK/1-636	---------------MRNWKKVSMIVLLVLIGFGFEYGMNPTPVDA--SSKIEYLVIQKPTDPPKDQPIKVIVSGEGKFCYGPDFSGGESYIIIEQCWQMHVMNAR
WP.002132024.1iUNK/1-638	---------------MCNWKKIFIVVLLVLIGFGFEYGINPTPVDA--SSKIEYSVIQKPTDPPKDKPIKVIVSDGGKFCYGPDFSGGESYIIIEQCWQMHVTNAR
UNK.iR21_1045318/1-636	---------------MRNWKKVSMIVLLVLIGFGFEYGMNPTPVDA--SSKIEYLVIQKPTDPPKDQPIKVIVSGEGKFCYGPDFSGGESYIIIEQCWQMHVMNAR
QHH34907.1iUNK/1-636	---------------MRNWKKVSMIVLLVLIGFGFEYGMNPTPVDA--SSKIEYLVIQKPTDPPKDQPIKVIVSGEGKFCYGPDFSGGESYIIIEQCWQMHVMNAR
WP.060684618.1iUNK/1-638	---------------MCNWKKIFIVVLLGLIGFGFEYGINPTPVDA--SSKIEYLVIQKPTDPPKDQPIKVIVSGEGKFCYGPDFSGGESYIIIEQCWQMHVMNAR
WP.025180450.1iUNK/1-630	-----------------------MVILLVATITYLKYGIDHTHIHA--SSKIEYSVIQKPTDPPKDKPIKVIVSGEGKFCYGPDFSGGESYIIIEQCWQMHVMNAR
WP.004486793.1iUNK/1-638	---------------MCNWKKIFIVVLLVLIGFGFEYGINPTPVDA--SSKIEYLVIQKPTDPPKDQPIKVIVSGEGKFCYGPDFSGGESYIIIEQCWQMHVMNAR
WP.002188597.1iUNK/1-638	---------------MCNWKKIFIVVLLGLIGFGFEYGINPTPVDA--SSKIEYLVIQKPTDPPKDQPIKVIVSGEGKFCYGPDFSGGESYIIIEQCWQMHVMNAR
UNK.iR21_3268864/1-638	---------------MCNWKKIFIVVLLVLIGFGFEYGINPTPVDA--SSKIEYLVIQKPTDPPKDQPIKVIVSGEGKFCYGPDFSGGESYIIIEQCWQMHVMNAR
QHH36556.1iUNK/1-638	---------------MCNWKKIFIVVLLVLIGFGFEYGINPTPVDA--SSKIEYLVIQKPTDPPKDQPIKVIVSGEGKFCYGPDFSGGESYIIIEQCWQMHVMNAR
HJO.iNorma_3449794/1-638	---------------MCNWKKIFIVVLLGLIGFGFEYGINPTPVDA--SSKIEYLVIQKPTDPPKDQPIKVIVSGEGKFCYGPDFSGGESYIIIEQCWQMHVMNAR
HJO.iL53_3395534/1-638	---------------MCNWKKIFIVVLLGLIGFGFEYGINPTPVDA--SSKIEYLVIQKPTDPPKDQPIKVIVSGEGKFCYGPDFSGGESYIIIEQCWQMHVMNAR
HJO.iHjtno_1031476/1-638	---------------MCNWKKIFIVVLLGLIGFGFEYGINPTPVDA--SSKIEYLVIQKPTDPPKDQPIKVIVSGEGKFCYGPDFSGGESYIIIEQCWQMHVMNAR
EKQ36801.1iUNK_s200200062/1-629	------------------------mILLVATITYLKYGIDHTHIHA--SSKIEYSVIQKPTDPPKDKPIKVIVSGEGKFCYGPDFSGGESYIIIEQCWQMHVMNAR
EMN71304.1iBAE_sUI08561/1-601	--mVIQKPTDPPKDQPIKVIVSGEGKFCYGPDFSGGESYIIIEQCWQMHVMNAR
WP.025185475.1iUNK/1-636	---------------MRNWKKVFMIVLLVLIGFGFEYGINPTPVDA--SSKIEYLVIQKPTDPPKDQPIKVIVSGEGKFCYGPDFSGGESYIIIEQCWQMHVMNAR
EMN80843.1iGSA_sUI12764/1-628	-----------------------MIVLLVLIGFGFEYGINPTPVDA--SSKIEYLVIQKPTDPPKDQPIKVIVSGEGKFCYGPDFSGGESYIIIEQCWQMHVMNAR
WP.033109388.1iUNK/1-638	---------------MCNWKKMFIVVLLVLIGLGFEYGMNPTPVDA--SSKIEYLVIQKPTDPPKDKPIKVIVSGEGKFCYGPDFSGGESYIIIEQCWQMHVMNAR
MAE.iUPMMCNIIDLP_934932/1-638	---------------MCNWKKMFIVVLLVLIGLGFEYGMNPTPVDA--SSKIEYLVIQKPTDPPKDKPIKVIVSGEGKFCYGPDFSGGESYIIIEQCWQMHVMNAR
MAE.iUPMMCNIIDHP_934934/1-638	---------------MCNWKKMFIVVLLVLIGLGFEYGMNPTPVDA--SSKIEYLVIQKPTDPPKDKPIKVIVSGEGKFCYGPDFSGGESYIIIEQCWQMHVMNAR
WP.141607883.1iUNK/1-638	---------------MCNWKKIFIVVLLVLIGFGFEYGINPTPVDA--SSKIEYLVIQKPTDPPKDQPIKVIVSGGGKFCYGPNFSGGESYIIIEQCWQMHVMNAR
WP.000336240.1iUNK/1-638	---------------MCNWKKIFIVVLLVLIGFGFEYGINPTPVDA--SSKIEYLVIQKPTDPPKDQPIKVIVSGGGKFCYGPNFSGGESYIIIEQCWQMHVMNAR
WP.057141802.1iUNK/1-638	---------------MRNWKKVFIVILLVLIGFGFEYGMNPTPVDA--SSRIEYSVIQKPTDPPKDKPIKIVIHDGGRFCYGPVFSGDESYIIIEQCWQMHVMNAR
CLA.iS114_1007645/1-638	---------------MCNWKKIFIVVLLVLIGFGFEYGMNPTPVDA--SSKIEYSVIQKPTDPPKDKPIKVIVSDGGKFCYGPDFSGGESYIIIEQCWQMHVTNAR
ASV05489.1iCLA_pB2G47.047/1-638	---------------MCNWKKIFIVVLLVLIGFGFEYGMNPTPVDA--SSKIEYSVIQKPTDPPKDKPIKVIVSDGGKFCYGPDFSGGESYIIIEQCWQMHVTNAR
WP.025179754.1iUNK/1-638	---------------MCNWKKIFIVVLLVLIGFGFEYGMNPTPVDA--SSKIEYSVIQKPTDPPKDKPIKVIVSDGGKFCYGPDFSGGESYIIIEQCWQMHVMNAR
WP.002190917.1iUNK/1-638	---------------MCNWKKIFIVVLLVLIGFGFEYGMNPTPVDA--SSKIEYSVIQKPTDPPKDKPIKVIVSDGGKFCYGPDFSGGESYIIIEQCWQMHVTNAR
WP.002183819.1iUNK/1-638	---------------MCNWKKIFIVVLLVLIGFGFEYGINPTPVDA--SSKIEYSVIQKPTDPPKDKPIKVIVSDGGKFCYGPDFSGGESYIIIEQCWQMHVMNAR
WP.002116716.1iUNK/1-638	---------------MCNWKKIFIVVLLVLIGFGFEYGMNPTPVDA--SSKIEYSVIQKPTDPPKDKPIKVIVSDGGKFCYGPDFSGGESYIIIEQCWQMHVTNAR
EKR55690.1iUNK_sUI12758/1-615	--------------------------------------MNPTPVDA--SSKIEYSVIQKPTDPPKDKPIKVIVSDGGKFCYGPDFSGGESYIIIEQCWQMHVMNAR
CLA.iS611_3276038/1-638	---------------MCNWKKIFIVVLLVLIGFGFEYGMNPTPVDA--SSKIEYSVIQKPTDPPKDKPIKVIVSDGGKFCYGPDFSGGESYIIIEQCWQMHVTNAR
CLA.iLJ178_3281064/1-638	---------------MCNWKKIFIVVLLVLIGFGFEYGMNPTPVDA--SSKIEYSVIQKPTDPPKDKPIKVIVSDGGKFCYGPDFSGGESYIIIEQCWQMHVTNAR
CLA.iRUFN_1902939/1-638	---------------MCNWKKIFIVVLLVLIGFGFEYGMNPTPVDA--SSKIEYSVIQKPTDPPKDKPIKVIVSDGGKFCYGPDFSGGESYIIIEQCWQMHVTNAR
ASV08925.1iCLA_pB2G50.088/1-638	---------------MCNWKKIFIVVLLVLIGFGFEYGMNPTPVDA--SSKIEYSVIQKPTDPPKDKPIKVIVSDGGKFCYGPDFSGGESYIIIEQCWQMHVTNAR
WP.192504757.1iUNK/1-636	---------------MRNWKKIFIVVLLVLIGFGLEYGINPTPVDA--SSKIEYSVIQKPTDPPKDKPIKVIVSGEGKFCYGPDFSGGESYIIIEQCWQMHVMNAR
BAE.iS1489_3378996/1-636	---------------MRNWKKIFIVVLLVLIGFGLEYGINPTPVDA--SSKIEYSVIQKPTDPPKDKPIKVIVSGEGKFCYGPDFSGGESYIIIEQCWQMHVMNAR
WP.192504942.1iUNK/1-638	---------------MCNWKKIFIVVLLVLIGFGFEYGINPTPVDA--SSKIEYLVIQKPTDPPKDQPIKVIVSGEGKFCYGPDFSGGESYIIIEQCWQMHVMNAR
BAE.iS1489_1027633/1-638	---------------MCNWKKIFIVVLLVLIGFGFEYGINPTPVDA--SSKIEYLVIQKPTDPPKDQPIKVIVSGEGKFCYGPDFSGGESYIIIEQCWQMHVMNAR
WP.002971337.1iUNK/1-638	---------------MRNWKKVSMIVLLVLIGFGFEYGMNPTPVDA--SSKIEYLVIQKPTDPPKDQPIKVIVSGEGKFCYGPDFSGGESYIIIEQCWQMHVMNAR
WP.192505399.1iUNK/1-644	---------------MCNWKKIFIVVLLVLIGFGFEYGINPTPVDA--SSKIEYLVIQKPTDPPKDKPIKVIVSGEGKFCYGPDFSGGESYIIIEQCWQMHVMNAR
CLA.iS782_3583294/1-644	---------------MCNWKKIFIVVLLVLIGFGFEYGINPTPVDA--SSKIEYLVIQKPTDPPKDKPIKVIVSGEGKFCYGPDFSGGESYIIIEQCWQMHVMNAR
WP.175270787.1iUNK/1-636	---------------MCNWKKIFIVVLLVLIGFGFEYGINPTPVDA--SSKIEYLVIQKPTDPPKDQPIKVIVSGEGKFCYGPDFSGGESYIIIEQCWQMHVMNAR
WP.192503304.1iUNK/1-638	---------------MCNWKKIFIVVLLVLIGFGFEYGMNPTPVDA--SSKIEYLVIQKPTDPPKDQPIKVIVSGEGKFCYGPDFSGGESYIIIEQCWQMHVMNAR
WP.105096096.1iUNK/1-666	---------------MRNWKKVFMIVLLVLIGFGLEYGINPTPVDA--SSKIEYSVIQKPTDPPKDKPIKVIVSGEGKFCYGPDFSGGESYIIIEQCWQMHVMNAR
WP.082280021.1iUNK/1-604	---------------m------------------------------------EYLVIQKPTDPPKDQPIKVIVSGEGKFCYGPDFSGGESYIIIEQCWQMHVMNAR
WP.045192774.1iUNK/1-638	---------------MRNWKKVFMIVLLVLIGFGFEYGINPTPVDA--SSKIEYLVIQKPTDPPKDKPIKVIVSGEGKFCYGPDFSGGESYIIIEQCWQMHVMNAR
WP.025177410.1iUNK/1-638	---------------MCNWKKVFMIVLLVLIGFGLEYGINPTPVDA--SSKIEYSVIQKPTDPPKDKPIKVIVSGEGKFCYGPDFSGGESYIIIEQCWQMHVMNAR
WP.002145500.1iUNK/1-638	---------------MCNWKKIFIVVLLVLIGFGFEYGINPTPVDA--SSKIEYLVIQKPTDPPKDQPIKVIVSGEGKFCYGPDFSGGESYIIIEQCWQMHVMNAR
WP.002109520.1iUNK/1-638	---------------MRNRKKVTIVILLVATITYLKYGIDHTHIHA--SSKIEYSVIQKPTDPPKDKPIKVIVSDGGKFCYGPNFSGGESYIIIEQCWQMHVMNAR
WP.002109128.1iUNK/1-638	---------------MCNWKKIFIVVLLVLIGFGFEYGINPTPVDA--SSKIEYLVIQKPTDPPKDQPIKVIVSGGGKFCYGPDFSGGESYIIIEQCWQMHVMNAR
WP.002102383.1iUNK/1-638	---------------MCNWKKIFIVVLLVLIGFGFEYGMNPTPVDA--SSKIEYSVIQKPTDPPKDKPIKVIVSDGGKFCYGPDFSGGESYIIIEQCWQMHVTNAR
WP.002097609.1iUNK/1-640	---------------MCNWKKIFIVVLLVLIGFGFEYGINPTPVDA--SSKIEYLVIQKPTDPPKDKPIKVIVSGEGKFCYGPDFSGGESYIIIEQCWQMHVMNAR
WP.002081463.1iUNK/1-638	---------------MCNWKKIFIVVLLVLIGFGFEYGINPTPVDA--SSKIEYLVIQKPTDPPKDKPIKVIVSGEGKFCYGPDFSGGESYIIIEQCWQMHVMNAR
WP.002079522.1iUNK/1-638	---------------MCNWKKIFIVVLLVLIGFGFEYGMNPTPVDA--SSKIEYLVIQKPTDPPKDKPIKVIVSGEGKFCYGPDFSGGESYIIIEQCWQMHVMNAR
WP.000336243.1iUNK/1-638	---------------MCNWKKVFMIVLLVLIGFGLEYGINPTPVDA--SSKIEYSVIQKPTDPPKDKPIKVIVSGEGKFCYGPDFSGGESYIIIEQCWQMHVMNAR
WP.000336237.1iUNK/1-638	---------------MCNWKKIFIVVLLVLIGFGFEYGINPTPVDA--SSKIEYLVIQKPTDPPKDQPIKVIVSGEGKFCYGPDFSGGESYIIIEQCWQMHVMNAR
WP.000336236.1iUNK/1-638	---------------MCNWKKIFIVVLLVLIGFGFEYGINPTPVDA--SSKIEYLVIQKPTDPPKDQPIKVIVSGEGKFCYGPDFSGGESYIIIEQCWQMHVMNAR
PD1Q19_PD1.3934_AG3.iPD1-/1-638	---------------MCNWKKIFIVVLLVLIGFGFEYGINPTPVDA--SSKIEYLVIQKPTDPPKDKPIKVIVSGEGKFCYGPDFSGGESYIIIEQCWQMHVMNAR

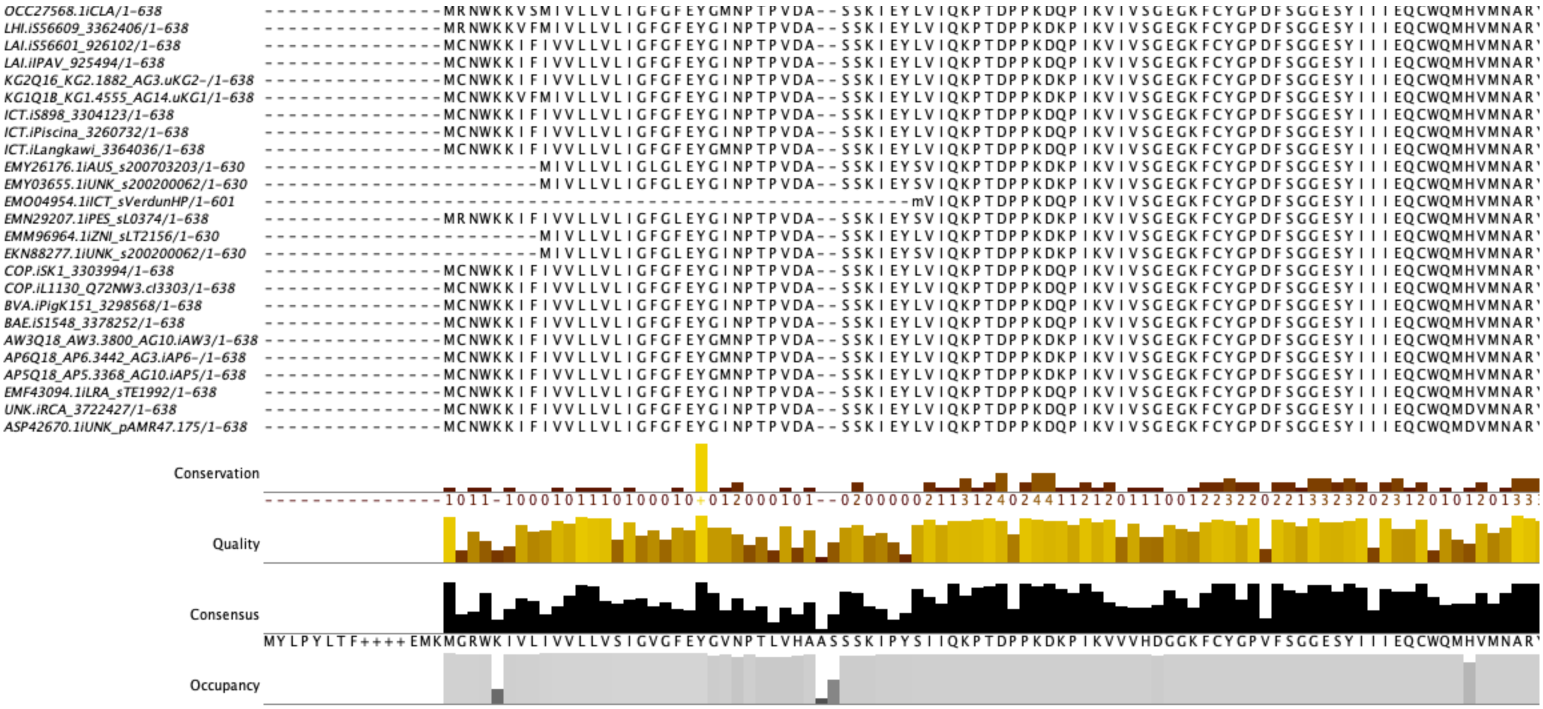
OCC27568.1iCLA/1-638
LHI.iS56609_3362406/1-638
LAI.iS56601_926102/1-638
LAI.iIPAV_925494/1-638
KG2Q16_KG2.1882_AG3.uKG2-/1-638
KG1Q18_KG1.4555_AG14.uKG1/1-638
ICT.iS898_3304123/1-638
ICT.iPiscina_3260732/1-638
ICT.iLangkawi_3364036/1-638
EMY26176.1iAUS_s200703203/1-630
EMY03655.1iUNK_s200200062/1-630
EMO04954.1iICT_sVerdunHP/1-601
EMN29207.1iPES_sL0374/1-638
EMM96964.1iZNI_sLT2156/1-630
EKN88277.1iUNK_s200200062/1-630
COP.iSK1_3303994/1-638
COP.iL1130_Q72NW3.cl3303/1-638
BVA.iPigK151_3298568/1-638
BAE.iS1548_3378252/1-638
AW3Q18_AW3.3800_AG10.iAW3/1-638
AP6Q18_AP6.3442_AG3.iAP6-/1-638
AP5Q18_AP5.3368_AG10.iAP5/1-638
EMF43094.1iLRA_sTE1992/1-638
UNK.iRCA_3722427/1-638
ASP42670.1iUNK_pAMR47.175/1-638
Conservation
Quality
Consensus
Occupancy
